# Portion, package or tableware size for changing selection and consumption of food, alcohol and tobacco

**DOI:** 10.1002/14651858.CD011045.pub2

**Published:** 2015-09-14

**Authors:** Gareth J Hollands, Ian Shemilt, Theresa M Marteau, Susan A Jebb, Hannah B Lewis, Yinghui Wei, Julian P T Higgins, David Ogilvie

**Affiliations:** University of CambridgeBehaviour and Health Research UnitForvie SiteRobinson WayCambridgeUKCB2 0SR; University College LondonEPPI‐Centre10 Woburn SquareLondonUKWC1H 0NR; University of OxfordNuffield Department of Primary Care Health SciencesRadcliffe Observatory QuarterWoodstock RoadOxfordOxfordshireUKOX2 6GG; MRC Human Nutrition ResearchElsie Widdowson Laboratory, 120 Fulbourn RoadCambridgeUKCB1 9NL; University of PlymouthCentre for Mathematical Sciences, School of Computing, Electronics and MathematicsPlymouthUK; University of BristolPopulation Health Sciences, Bristol Medical SchoolCanynge Hall39 Whatley RoadBristolUKBS8 2PS; University of CambridgeMRC Epidemiology UnitBox 285Cambridge Biomedical CampusCambridgeUKCB2 0QQ

**Keywords:** Adult, Child, Humans, Alcohol Drinking, Eating, Food Preferences, Smoking, Beverages, Beverages/statistics & numerical data, Cooking and Eating Utensils, Cooking and Eating Utensils/standards, Drinking Behavior, Portion Size, Portion Size/standards, Product Packaging, Product Packaging/standards, Randomized Controlled Trials as Topic

## Abstract

**Background:**

Overeating and harmful alcohol and tobacco use have been linked to the aetiology of various non‐communicable diseases, which are among the leading global causes of morbidity and premature mortality. As people are repeatedly exposed to varying sizes and shapes of food, alcohol and tobacco products in environments such as shops, restaurants, bars and homes, this has stimulated public health policy interest in product size and shape as potential targets for intervention.

**Objectives:**

1) To assess the effects of interventions involving exposure to different sizes or sets of physical dimensions of a portion, package, individual unit or item of tableware on unregulated selection or consumption of food, alcohol or tobacco products in adults and children.

2) To assess the extent to which these effects may be modified by study, intervention and participant characteristics.

**Search methods:**

We searched CENTRAL, MEDLINE, EMBASE, PsycINFO, eight other published or grey literature databases, trial registries and key websites up to November 2012, followed by citation searches and contacts with study authors. This original search identified eligible studies published up to July 2013, which are fully incorporated into the review. We conducted an updated search up to 30 January 2015 but further eligible studies are not yet fully incorporated due to their minimal potential to change the conclusions.

**Selection criteria:**

Randomised controlled trials with between‐subjects (parallel‐group) or within‐subjects (cross‐over) designs, conducted in laboratory or field settings, in adults or children. Eligible studies compared at least two groups of participants, each exposed to a different size or shape of a portion of a food (including non‐alcoholic beverages), alcohol or tobacco product, its package or individual unit size, or of an item of tableware used to consume it, and included a measure of unregulated selection or consumption of food, alcohol or tobacco.

**Data collection and analysis:**

We applied standard Cochrane methods to select eligible studies for inclusion and to collect data and assess risk of bias. We calculated study‐level effect sizes as standardised mean differences (SMDs) between comparison groups, measured as quantities selected or consumed. We combined these results using random‐effects meta‐analysis models to estimate summary effect sizes (SMDs with 95% confidence intervals (CIs)) for each outcome for size and shape comparisons. We rated the overall quality of evidence using the GRADE system. Finally, we used meta‐regression analysis to investigate statistical associations between summary effect sizes and variant study, intervention or participant characteristics.

**Main results:**

The current version of this review includes 72 studies, published between 1978 and July 2013, assessed as being at overall unclear or high risk of bias with respect to selection and consumption outcomes. Ninety‐six per cent of included studies (69/72) manipulated food products and 4% (3/72) manipulated cigarettes. No included studies manipulated alcohol products. Forty‐nine per cent (35/72) manipulated portion size, 14% (10/72) package size and 21% (15/72) tableware size or shape. More studies investigated effects among adults (76% (55/72)) than children and all studies were conducted in high‐income countries ‐ predominantly in the USA (81% (58/72)). Sources of funding were reported for the majority of studies, with no evidence of funding by agencies with possible commercial interests in their results.

A meta‐analysis of 86 independent comparisons from 58 studies (6603 participants) found a small to moderate effect of portion, package, individual unit or tableware size on consumption of food (SMD 0.38, 95% CI 0.29 to 0.46), providing moderate quality evidence that exposure to larger sizes increased quantities of food consumed among children (SMD 0.21, 95% CI 0.10 to 0.31) and adults (SMD 0.46, 95% CI 0.40 to 0.52). The size of this effect suggests that, if sustained reductions in exposure to larger‐sized food portions, packages and tableware could be achieved across the whole diet, this could reduce average daily energy consumed from food by between 144 and 228 kcal (8.5% to 13.5% from a baseline of 1689 kcal) among UK children and adults. A meta‐analysis of six independent comparisons from three studies (108 participants) found low quality evidence for no difference in the effect of cigarette length on consumption (SMD 0.25, 95% CI ‐0.14 to 0.65).

One included study (50 participants) estimated a large effect on consumption of exposure to differently shaped tableware (SMD 1.17, 95% CI 0.57 to 1.78), rated as very low quality evidence that exposure to shorter, wider bottles (versus taller, narrower bottles) increased quantities of water consumed by young adult participants.

A meta‐analysis of 13 independent comparisons from 10 studies (1164 participants) found a small to moderate effect of portion or tableware size on selection of food (SMD 0.42, 95% CI 0.24 to 0.59), rated as moderate quality evidence that exposure to larger sizes increased the quantities of food people selected for subsequent consumption. This effect was present among adults (SMD 0.55, 95% CI 0.35 to 0.75) but not children (SMD 0.14, 95% CI ‐0.06 to 0.34).

In addition, a meta‐analysis of three independent comparisons from three studies (232 participants) found a very large effect of exposure to differently shaped tableware on selection of non‐alcoholic beverages (SMD 1.47, 95% CI 0.52 to 2.43), rated as low quality evidence that exposure to shorter, wider (versus taller, narrower) glasses or bottles increased the quantities selected for subsequent consumption among adults (SMD 2.31, 95% CI 1.79 to 2.83) and children (SMD 1.03, 95% CI 0.41 to 1.65).

**Authors' conclusions:**

This review found that people consistently consume more food and drink when offered larger‐sized portions, packages or tableware than when offered smaller‐sized versions. This suggests that policies and practices that successfully reduce the size, availability and appeal of larger‐sized portions, packages, individual units and tableware can contribute to meaningful reductions in the quantities of food (including non‐alcoholic beverages) people select and consume in the immediate and short term. However, it is uncertain whether reducing portions at the smaller end of the size range can be as effective in reducing food consumption as reductions at the larger end of the range. We are unable to highlight clear implications for tobacco or alcohol policy due to identified gaps in the current evidence base.

## Plain language summary

**Portion, package or tableware size for changing selection and consumption of food, alcohol and tobacco**

**Review question**

We reviewed the evidence to establish by how much the amounts of food, alcohol or tobacco adults and children select or consume change in response to being presented with larger or smaller‐sized (or differently shaped) portions or packages of these products, or of items of tableware (such as plates or glasses) used to consume them.

**Study characteristics**

This review includes 72 randomised controlled trials (RCTs) published up to July 2013 that compared at least two groups of participants, each presented with a different size of a portion, package or item of tableware. Included studies measured the amounts of food, alcohol or tobacco selected and/or consumed by participants, typically over a period of one day or less. Almost all of the included studies investigated food, with only three tobacco studies and no alcohol studies found. Almost all assessed participants' responses to different sizes rather than different shapes. The average age of participants in the different studies ranged from three to 55 years, with more studies involving adults than children and most conducted in the USA. Sources of funding were reported for the majority of studies and there was no evidence of study funding by agencies with commercial interests in their results.

**Key findings and quality of evidence**

***Effects of size on consumption:*** We found evidence that people consistently ate more food or drank more non‐alcoholic drinks when offered larger‐sized portions, packages or items of tableware than when offered smaller‐sized versions. We estimate the size of this effect to be small to moderate among both children and adults. If an effect of this size were sustained across the whole diet it would be equivalent to around a 12% to 16% change in average daily energy intake from food among UK adults. We rated the overall quality of the evidence for this effect as moderate, due to concern about study limitations arising from incomplete or unclear reporting of methods and procedures. From three tobacco studies, we found no effect of longer compared with shorter cigarettes on the amounts of tobacco consumed. We rated the overall quality of evidence for this effect as low due to concerns about study limitations and not having enough evidence.

***Effects of shape on consumption:*** One study found that adults provided with shorter, wider bottles drank larger amounts of water from them, having already poured more, compared with those provided with taller, narrower bottles. However, we rated the quality of this evidence as very low, due to very serious concerns about study limitations and not having enough evidence (only one study with outcome data from 50 participants).

***Effects of size on selection:*** We further found that adults, but not children, consistently chose (selected) more food (including non‐alcoholic drinks) when offered larger‐sized portions, packages or items of tableware than when offered smaller‐sized versions. The estimated size of this effect was again small to moderate. We rated the overall quality of the evidence for this effect as moderate, due to concern about study limitations.

***Effects of shape on selection:*** Evidence from three studies suggested that adults and children provided with shorter, wider bottles or glasses selected increased quantities of non‐alcoholic beverages for subsequent consumption, compared with those provided with taller, narrower bottles or glasses. We rated the quality of this evidence as low, again due to concerns about study limitations and unexplained variation in effects between the three studies.

**Conclusions**

Overall, this review provides the most conclusive evidence to date that acting to reduce the size, availability and appeal of larger‐sized portions, packages and tableware has potential to reduce the quantities of food that people select and consume by meaningful amounts. However, it is uncertain whether reducing portions at the smaller end of the size range can be as effective in reducing food consumption as reductions at the larger end of the range. Our findings highlight the need for further research that aims to reduce uncertainties about these effects and address identified gaps in the evidence base, including not having enough evidence for longer‐term effects and the absence of evidence about alcohol products.

## Summary of findings

**Summary of findings for the main comparison:** Food: Larger versus smaller‐sized portions, packages or tableware for changing quantity consumed or selected

**Food: Larger versus smaller‐sized portions, packages or tableware for changing quantity consumed or selected**						
**Population:** children and adults **Settings:** high‐income countries, laboratory and field settings **Intervention:** larger‐sized portion, package, individual unit or item of tableware **Comparison**: smaller‐sized portion, package, individual unit or item of tableware						
**Outcomes**	Illustrative comparative risks* (95% CI)		**Relative effect (95% CI)**	**No of participants (studies)**	**Quality of the evidence (GRADE)**	**Comments**
	**Assumed risk**	**Corresponding risk**				
	**Smaller‐sized portion, package, individual unit or item of tableware**	**Larger‐sized portion, package, individual unit or item of tableware**				
Consumption	Mean daily energy intake from food among a representative sample of UK children and adults is 1689 kcal^3^	Mean daily energy intake from food would be 189 kcal (11.2%) higher with the intervention (144 to 228 kcal higher) among UK children and adults	Mean consumption in the intervention group was 0.38 standard deviations higher (0.29 higher to 0.46 higher)	6603 (86 independent comparisons)	⊕⊕⊕⊝ MODERATE ^1^	
‐ Consumption among children	Mean daily energy intake from food among a representative sample of UK children is 1651 kcal^3^	Mean daily energy intake from food would be 95 kcal (5.7%) higher with the intervention (45 to 140 kcal higher) among UK children	Mean consumption in the intervention group was 0.21 standard deviations higher (0.1 higher to 0.31 higher)	1421 (22 independent comparisons)	⊕⊕⊕⊝ MODERATE ^1^	
‐ Consumption among adults	Mean daily energy intake from food among a representative sample of UK adults is 1727 kcal^3^	Mean daily energy intake from food would be 247 kcal (14.3%) higher with the intervention (215 to 279 kcal higher) among UK adults	Mean consumption in the intervention group was 0.46 standard deviations higher (0.40 higher to 0.52 higher)	5182 (64 independent comparisons)	⊕⊕⊕⊝ MODERATE ^1^	
Selection without purchase	Mean daily energy intake from food among a representative sample of UK children and adults is 1689 kcal^3^	Mean daily energy intake from food would be 209 kcal (12.4%) higher with the intervention (119 to 293 kcal higher) among UK children and adults^4^	Mean selection without purchase in the intervention group was 0.42 standard deviations higher (0.24 higher to 0.59 higher)	1164 (13 independent comparisons)	⊕⊕⊕⊝ MODERATE ^1^	
‐ Selection without purchase among children	Mean daily energy intake from food among a representative sample of UK children is 1651 kcal^3^	Mean daily energy intake from food would be 63 kcal (3.8%) higher with the intervention (27 to 153 kcal higher) among UK children^4^	Mean selection without purchase in the intervention group was 0.14 standard deviations higher (0.06 lower to 0.34 higher)	382 (4 independent comparisons)	⊕⊕⊝⊝ LOW ^1,2^	
‐ Selection without purchase among adults	Mean daily energy intake from food among a representative sample of UK adults is 1727 kcal^3^	Mean daily energy intake from food would be 188 kcal (10.9%) higher with the intervention (188 to 403 kcal higher) among UK adults^4^	Mean selection without purchase in the intervention group was 0.55 standard deviations higher (0.35 higher to 0.75 higher)	782 (9 independent comparisons)	⊕⊕⊕⊝ MODERATE ^1^	
*The basis for the **assumed risk** (e.g. the median control group risk across studies) is provided in footnotes. The **corresponding risk** (and its 95% confidence interval) is based on the assumed risk in representative UK samples^3^ and the **relative effect** of the intervention (and its 95% CI). **CI:** confidence interval						
GRADE Working Group grades of evidence **High quality:** Further research is very unlikely to change our confidence in the estimate of effect. **Moderate quality:** Further research is likely to have an important impact on our confidence in the estimate of effect and may change the estimate. **Low quality:** Further research is very likely to have an important impact on our confidence in the estimate of effect and is likely to change the estimate. **Very low quality:** We are very uncertain about the estimate.						

^1^Rated down by one level for study limitations: we assessed risk of bias as unclear or high in all incorporated studies. ^2^Rated down by one level for imprecision: number of participants (effective sample size) incorporated into analysis is less than the number of patients generated by a conventional sample size calculation for a single adequately powered trial (optimal information size) and the confidence interval crosses zero. ^3^Estimates of means and standard deviations based on an unweighted analysis of data from the UK National Diet and Nutrition Survey, Years 1‐4 (National Centre for Social Research 2012) ‐ see Data synthesis. ^4^Illustration of equivalent absolute effect on daily energy intake from food assumes that all foods selected are consumed.

**Summary of findings 2:** Alcohol: Larger versus smaller‐sized portions, packages or tableware for changing quantity consumed or selected

**Alcohol: Larger versus smaller‐sized portions, packages or tableware for changing quantity consumed or selected**						
**Population:** children and adults **Settings:** high‐income countries, laboratory and field settings **Intervention:** larger‐sized portion, package, individual unit or item of tableware **Comparison:** smaller‐sized portion, package, individual unit or item of tableware						
**Outcomes**	Illustrative comparative risks* (95% CI)		**Relative effect (95% CI)**	**No of participants (studies)**	**Quality of the evidence (GRADE)**	**Comments**
	**Assumed risk**	**Corresponding risk**				
	**Smaller‐sized portion, package, individual unit or item of tableware**	**Larger‐sized portion, package, individual unit or item of tableware**				
Consumption	No evidence is available	‐	‐	(0 independent comparisons)	‐	‐
‐ Consumption among children	No evidence is available	‐	‐	(0 independent comparisons)	‐	‐
‐ Consumption among adults	No evidence is available	‐	‐	(0 independent comparisons)	‐	‐
Selection with or without purchase	No evidence is available	‐	‐	(0 independent comparisons)	‐	‐
‐ Selection with or without purchase among children	No evidence is available	‐	‐	(0 independent comparisons)	‐	‐
‐ Selection with or without purchase among adults	No evidence is available	‐	‐	(0 independent comparisons)	‐	‐
*The basis for the **assumed risk** (e.g. the median control group risk across studies) is provided in footnotes. The **corresponding risk** (and its 95% confidence interval) is based on the assumed risk in the comparison group and the **relative effect** of the intervention (and its 95% CI). **CI:** confidence interval						
GRADE Working Group grades of evidence **High quality:** Further research is very unlikely to change our confidence in the estimate of effect. **Moderate quality:** Further research is likely to have an important impact on our confidence in the estimate of effect and may change the estimate. **Low quality:** Further research is very likely to have an important impact on our confidence in the estimate of effect and is likely to change the estimate. **Very low quality:** We are very uncertain about the estimate.						

**Summary of findings 3:** Tobacco: Longer versus shorter cigarettes for changing quantity consumed or selected

**Tobacco: Longer versus shorter cigarettes for changing quantity consumed or selected**						
**Population:** children and adults **Settings:** high‐income countries, laboratory settings **Intervention:** longer cigarettes **Comparison:** shorter cigarettes						
**Outcomes**	Illustrative comparative risks* (95% CI)		**Relative effect (95% CI)**	**No of participants (studies)**	**Quality of the evidence (GRADE)**	**Comments**
	**Assumed risk**	**Corresponding risk**				
	**Shorter cigarettes **	**Longer cigarettes**				
Consumption	Mean number of cigarettes smoked per day among a representative sample of UK adults is 13	Mean number of cigarettes smoked per day would be 2 higher with the intervention (1 to 5 higher) among UK adults	Mean consumption in the intervention group was 0.25 standard deviations higher (0.14 lower to 0.65 higher)	108 (6 independent comparisons)	⊕⊕⊝⊝ LOW ^1,2^	‐
‐ Consumption among children	No evidence is available	‐	‐	(0 independent comparisons)	‐	‐
‐ Consumption among adults	Mean number of cigarettes smoked per day among a representative sample of UK adults is 13	Mean number of cigarettes smoked per day would be 2 higher with the intervention (1 to 5 higher) among UK adults	Mean consumption in the intervention group was 0.25 standard deviations higher (0.14 lower to 0.65 higher)	108 (6 independent comparisons)	⊕⊕⊝⊝ LOW ^1,2^	‐
Selection with or without purchase	No evidence is available	‐	‐	(0 independent comparisons)	‐	‐
‐ Selection with or without purchase among children	No evidence is available	‐	‐	(0 independent comparisons)	‐	‐
‐ Selection with or without purchase among adults	No evidence is available	‐	‐	(0 independent comparisons)	‐	‐
*The basis for the **assumed risk** (e.g. the median control group risk across studies) is provided in footnotes. The **corresponding risk** (and its 95% confidence interval) is based on the assumed risk in the comparison group and the **relative effect** of the intervention (and its 95% CI). **CI:** confidence interval						
GRADE Working Group grades of evidence **High quality:** Further research is very unlikely to change our confidence in the estimate of effect. **Moderate quality:** Further research is likely to have an important impact on our confidence in the estimate of effect and may change the estimate. **Low quality:** Further research is very likely to have an important impact on our confidence in the estimate of effect and is likely to change the estimate. **Very low quality:** We are very uncertain about the estimate.						

^1^Rated down by one level for study limitations: we assessed risk of bias as unclear or high in all incorporated studies. ^2^Rated down by one level for imprecision: number of participants (effective sample size) incorporated into analysis is less than the number of patients generated by a conventional sample size calculation for a single adequately powered trial (optimal information size) and confidence interval crosses zero. ^3^Estimates of means and standard deviations based on an unweighted analysis of data from the UK Opinions and Lifestyle Survey, 2012 (Office for National Statistics 2012) ‐ see Data synthesis.

**Summary of findings 4:** Food: Shorter, wider versus taller, narrower glasses or plastic bottles (shape) for changing quantity of non‐alcoholic beverages consumed or selected

**Shorter, wider versus taller, narrower glasses or plastic bottles (shape) for changing quantity of non‐alcoholic beverages consumed or selected**						
**Patient or population:** children and adults **Settings:** high‐income countries, field settings **Intervention:** shorter, wider glasses or plastic bottles **Comparison:** taller, narrower glasses or plastic bottles						
**Outcomes**	Illustrative comparative risks* (95% CI)		**Relative effect (95% CI)**	**No of participants (studies)**	**Quality of the evidence (GRADE)**	**Comments**
	**Assumed risk**	**Corresponding risk**				
	**Shorter, wider glasses or plastic bottles**	**Taller, narrower glasses or plastic bottles**				
Consumption	Mean quantity of energy‐containing non‐alcoholic beverages consumed in a single serve among a representative sample of UK adults is 245 grams^8^	Mean quantity of energy‐containing non‐alcoholic beverages consumed in a single serve would be 195 grams (79.6%) higher with the intervention (95 to 296 grams higher) among UK adults	Mean consumption in the intervention group was 1.17 standard deviations higher (0.57 higher to 1.78 higher)	50 (1 independent comparison)	⊕⊝⊝⊝ VERY LOW ^1,2^	‐
‐ Consumption among adults	Mean quantity of energy‐containing non‐alcoholic beverages consumed in a single serve among a representative sample of UK adults is 245 grams^8^	Mean quantity of energy‐containing non‐alcoholic beverages consumed in a single serve would be 195 grams (79.6%) higher with the intervention (95 to 296 grams higher) among UK adults	Mean consumption in the intervention group was 1.17 standard deviations higher (0.57 higher to 1.78 higher)	50 (1 independent comparison)	⊕⊝⊝⊝ VERY LOW ^1,2^	‐
‐ Consumption among children	No evidence is available	‐	‐	(0 independent comparisons)	‐	‐
Selection without purchase	Mean quantity of energy‐containing non‐alcoholic beverages consumed in a single serve among a representative sample of UK children and adults is 234 grams^8^	Mean quantity of energy‐containing non‐alcoholic beverages consumed in a single serve would be 242 grams (103.4%) higher with the intervention (86 to 400 grams higher) among UK children and adults^9^	Mean selection without purchase in the intervention group was 1.47 standard deviations higher (0.52 higher to 2.43 higher)	232 (3 independent comparisons)	⊕⊕⊝⊝ LOW ^3,4^	‐
‐ Selection without purchase among children	Mean quantity of energy‐containing non‐alcoholic beverages consumed in a single serve among a representative sample of UK children is 228 grams^8^	Mean quantity of energy‐containing non‐alcoholic beverages consumed in a single serve would be 377 grams (165.5%) higher with the intervention (292 to 462 grams higher) among UK children^9^	Mean selection without purchase in the intervention group was 2.31 standard deviations higher (1.79 higher to 2.83 higher)	96 (1 independent comparison)	⊕⊕⊝⊝ LOW ^5, 6^	‐
‐ Selection without purchase among adults	Mean quantity of energy‐containing non‐alcoholic beverages consumed in a single serve among a representative sample of UK adults is 245 grams^8^	Mean quantity of energy‐containing non‐alcoholic beverages consumed in a single serve would be 171 grams (70.1%) higher with the intervention (68 to 274 grams higher) among UK adults^9^	Mean selection without purchase in the intervention group was 1.03 standard deviations higher (0.41 higher to 1.65 higher)	136 (2 independent comparisons)	⊕⊕⊝⊝ LOW ^3,7^	‐
*The basis for the **assumed risk** (e.g. the median control group risk across studies) is provided in footnotes. The **corresponding risk** (and its 95% confidence interval) is based on the assumed risk in the comparison group and the **relative effect** of the intervention (and its 95% CI). **CI:** confidence interval						
GRADE Working Group grades of evidence **High quality:** Further research is very unlikely to change our confidence in the estimate of effect. **Moderate quality:** Further research is likely to have an important impact on our confidence in the estimate of effect and may change the estimate. **Low quality:** Further research is very likely to have an important impact on our confidence in the estimate of effect and is likely to change the estimate. **Very low quality:** We are very uncertain about the estimate.						

^1^Rated down two levels for study limitations: study assessed at high risk of bias with respect to the consumption outcome (see Characteristics of included studies 'Risk of bias' tables). ^2^Rated down one level for imprecision: number of participants (effective sample size) incorporated into analysis is less than the number of patients generated by a conventional sample size calculation for a single adequately powered trial (optimal information size) based on the lower limit of the confidence interval. ^3^Rated down one level for study limitations: studies assessed at unclear or high risk of bias with respect to the selection outcome (see Characteristics of included studies 'Risk of bias' tables). ^4^Rated down one level for inconsistency. I^2^ statistic from the random‐effects model shows that 90.1% of the total variance in study‐level estimates of this effect was due to statistical heterogeneity. ^5^Rated down one level for study limitations: study assessed at unclear risk of bias with respect to the selection outcome (see Characteristics of included studies 'Risk of bias' tables). ^6^Rated down one level for imprecision: single study. ^7^Rated down one level for inconsistency: point estimates are dissimilar and confidence intervals do not overlap. ^8^Estimates of means and standard deviations based on an unweighted analysis of data from the UK National Diet and Nutrition Survey, Years 1‐4 (National Centre for Social Research 2012) ‐ see Data synthesis. ^9^Illustration of equivalent absolute effect on quantity of energy‐containing non‐alcoholic beverages consumed in single serve assumes that all energy‐containing non‐alcoholic beverage selected in a single serve is consumed.

## Background

### Description of the condition

Non‐communicable diseases, principally cardiovascular diseases, diabetes, certain forms of cancer and chronic respiratory diseases, accounted for an estimated 62% of all deaths worldwide in 2012 (World Health Organization 2014a), and globally the proportion of years of life lost as a result of non‐communicable diseases increased from 38% in 2000 to 47% in 2012 (World Health Organization 2014b). Major risk factors for these conditions are in part determined by patterns of behaviour that are in principle modifiable, including consumption of food, alcohol and tobacco products (United Nations 2014). Identifying interventions that are effective in achieving sustained health behaviour change has therefore become one of the most important public health challenges of the 21^st^ century.

### Description of the intervention

It is increasingly recognised that the physical environments that surround us can exert considerable influences on our health behaviour and that altering these environments may provide a catalyst for behaviour change (Das 2012). In a recent scoping review, we described a class of interventions that involve altering the properties or placement of objects or stimuli within micro‐environments such as shops, restaurants, bars or homes, with the intention of changing health‐related behaviours (Hollands 2013a; Hollands 2013b).

The size of a portion or package is a modifiable property of food, alcohol and tobacco products that may influence their selection and consumption. In the case of food and alcohol products, the size or shape of an item of tableware used to consume such products may similarly influence their selection and consumption. Examples include the portion size of alcoholic beverages served in bars or of foods served in restaurants, at a buffet or in the home, such as portions of a dish served to restaurant customers (Diliberti 2004), the size or shape of plates or glasses used to serve products (Shah 2011), and the number or length of cigarettes in packets sold in shops (Russell 1980). In this context, the intervention involves manipulation of the size or physical dimensions of a food, alcohol or tobacco product, its packaging or the tableware used in its consumption. Comparisons of interest are between products, packages or items of tableware that differ only in terms of these properties.

### How the intervention might work

There are considerable influences on behaviour that are beyond individuals' deliberative control. Indeed, it has been suggested that most human behaviour occurs outside of awareness, cued by stimuli in environments and resulting in actions that may be largely unaccompanied by conscious reflection (Marteau 2012; Neal 2006). This proposition has led to increasing policy and research attention being placed on interventions with mechanisms of action that are less dependent on the conscious engagement of the recipients, including interventions that involve altering properties of objects or stimuli within the small‐scale environments that surround and cue behaviour (Hollands 2013a).

A number of mechanisms of action have been proposed to explain how the size of products may affect their consumption (Herman 2015; Steenhuis 2009). It has been suggested that as the amount of a product made available for consumption is increased, individuals will continue to perceive each increasing amount as an appropriate quantity to consume. This phenomenon may be explained by several mediating factors including personal and social norms about what constitutes a suitable amount of a product to consume. Such norms can be influenced by the amounts that are presented for consumption, and larger portions of food have become increasingly prevalent, making it increasingly unlikely that smaller portions are viewed as normal or appropriate for a single serving (Young 2002). There is also a tendency for individuals to engage most comfortably with a product as a single entity independent of its size. This 'unit bias' means that they are predisposed to consume the entirety of a product even as it changes size (Geier 2006). In addition, the way in which products are presented can influence their consumption. The presentation of food and alcohol products often entails the use of tableware, such as plates, glasses or cutlery. Not only does the size of tableware have the potential to directly influence the amount of a product available for consumption (Pratt 2012), but its physical dimensions can elicit various cognitive biases (Wansink 2005), which may influence perceptions of quantity and in turn determine levels of consumption. Similarly, sub‐dividing a fixed portion of a food into smaller pieces also affects perceptions of quantity (Scisco 2012). All of these mechanisms may also influence product selection (with or without purchasing), which is an important intermediate outcome in pathways to consumption.

Extant research involving the experimental manipulation of portion, package or tableware size has focused on food (including non‐alcoholic beverage) products to a much greater extent than tobacco products (Hollands 2013a). Whilst the causal mechanisms of underlying potential effects of such manipulations on selection or consumption of tobacco may be assumed to be broadly similar to food, smokers are known to titrate their received dose of nicotine to regulate the level in the body, with the potential to attenuate the effects of interventions to alter the size of tobacco products (Kozlowski 1986).

### Why it is important to do this review

A recent scoping review of evidence for the effects of choice architecture interventions identified a substantial number of randomised controlled trials that have investigated the effects of exposure to different portion, package or tableware sizes on selection and consumption behaviours (Hollands 2013a). The majority of these studies focused on food products, but because both tobacco and alcohol use also involve the selection and consumption of products, similar interventions may have the potential to change these behaviours via similar mechanisms. To our knowledge, evidence from these studies has yet to be synthesised using rigorous systematic review methods that include assessment of risk of bias and investigation of potential effect modifiers, nor to encompass alcohol and tobacco use. As such, we do not yet have reliable estimates of the effects of altering the sizes of portions, packages or tableware on product selection and consumption, nor of the influence of factors that may modify any such effects. Both are necessary to inform the selection and design of effective public health interventions.

Interventions that aim to reduce people's exposure to larger or smaller food portions, as opposed to those that involve providing information to encourage health behaviour change, may also have the potential to reduce health inequalities if they rely less on recipients' levels of literacy, numeracy and cognitive control, which have been found to be lower in population subgroups experiencing higher levels of social and material deprivation (Kutner 2006; Marteau 2012; Spears 2010; Williams 2003). Despite evidence that behaviours with the potential to undermine health are socially patterned (for example, that people in lower socioeconomic groups tend to consume less fruit and vegetables (Giskes 2010)), potential differences in behavioural responses to product sizing interventions between socioeconomic subgroups remain unclear. Also, to our knowledge (prior to conducting this review), no studies of the effects of product size had been conducted in low or middle‐income (LMIC) country populations (Hollands 2013a). This review therefore includes a focus on identifying evidence for differential effects of exposure to different sizes of these products between socioeconomic subgroups (and between studies conducted in LMIC and high‐income countries (HIC)), highlight any identified gaps in this aspect of the evidence base, and seek to draw implications for the potential of such interventions to affect health inequalities.

This systematic review is also timely given current interest in the topic within public health policy circles. There is evidence from the USA and Europe that portion sizes have been increasing since the 1970s (Young 2002; Young 2012). There have also been recent attempts to regulate the size of products in order to reduce consumption levels and improve public health, such as New York City Mayor Michael Bloomberg's proposed ban on the sale of sugary drinks larger than 16 oz (473 ml) (Gabbatt 2013). In the UK, there are recent examples of companies reducing the portion sizes of confectionery and sugary drinks as part of the Public Health Responsibility Deal in England. This systematic review can contribute to a better evidence‐based understanding of the potential impact of such policies.

## Objectives

1. To assess the effects of interventions involving exposure to different sizes or sets of physical dimensions of a portion, package, individual unit or item of tableware on unregulated (ad libitum) selection or consumption of food, alcohol or tobacco products in adults and children.
2. To assess the extent to which the effects of such interventions may be modified by:
  - study characteristics, such as target product type (food, alcohol, tobacco) or whether the target of the manipulation is a portion, package, individual unit or item of tableware;
  - intervention characteristics, such as magnitude of the difference in size; and
  - participant characteristics, such as age, gender or socioeconomic status (to facilitate an assessment of social differentiation in effects relevant to health equity).

## Methods

### Criteria for considering studies for this review

#### Types of studies

Randomised controlled trials with between‐subjects (parallel‐group) or within‐subjects (cross‐over) designs, conducted in laboratory or field settings. We excluded non‐randomised studies because our recent scoping review indicated that a sufficient number of eligible randomised controlled trials would be available to address our aim to synthesise evidence for intervention effects (Hollands 2013a). A key issue is that, compared with randomised controlled trials, non‐randomised studies rely on more stringent and sometimes non‐verifiable assumptions in order to confer confidence that, with successful implementation of the study design, the risk of systematic differences between comparison groups beyond the intervention of interest (i.e. confounding) is sufficiently low to permit valid inferences about causal effects.

#### Types of participants

Adults and children directly engaged with the manipulated products. We set no exclusion criteria in relation to demographic, socioeconomic or clinical characteristics or prognostic factors. We excluded studies involving non‐human participants (animal studies).

#### Types of interventions

Interventions eligible to be considered in this review were those that involved comparison of the effects of exposure to at least two sizes or sets of visible physical dimensions (that is volume, shape, height, width or depth) of either a portion of the same food (including non‐alcoholic beverages), alcohol or tobacco product, its package or individual unit size, or an item of tableware used to consume it. An eligible study could therefore include multiple eligible comparisons. For example, in a three‐arm between‐subjects study comparing the effects of exposure to a 200 g, 300 g or 400 g portion of pasta with sauce, eligible comparisons are: 200 g versus 300 g; 300 g versus 400 g; and 200 g versus 400 g (see also Data synthesis).

'Portion' refers to the overall amount (volume, weight or both) of a product that is presented for selection or consumption (for example, 200 g versus 300 g of pasta, 275 ml versus 440 ml of beer, or a packet of 10 versus 20 cigarettes). 'Package' refers to the different ways of packaging a specific portion, including that used for service, consumption or storage (for example, boxes, bags, cans or bottles). For example, the same portion of a food could be served within one large bag or multiple smaller bags. 'Individual unit' refers to the unit of a product that is presented within a given portion (for example, individual sweets or candies, biscuits or cookies, or cigarettes). 'Tableware' refers to crockery, cutlery or glassware used for serving or consuming food or drink (for example, plates, bowls, knives, forks, spoons or glasses). Packages and tableware as defined in this way have the capacity to limit or increase the portion or individual unit size of the consumed product and may therefore influence any corollary effects on selection and consumption.

We excluded the following:

- Interventions in which product size and/or shape may have been altered indirectly as a result of a higher‐level intervention but were not directly manipulated, to safeguard implementation fidelity (e.g. organisational‐level interventions to encourage the introduction of small‐scale environmental changes to alter product selection or consumption).
- Interventions in which the behavioural responses of participants (that is, selection or consumption levels or rates) were regulated by either explicit instructions to participants or some other action of the researcher (e.g. participants exposed to a product were given instructions on how much they should consume or a target rate of consumption). In such cases, selection or consumption of the manipulated product cannot be considered unregulated (ad libitum).
- Studies that compared packages, portions, individual units or tableware of different types or with different functions. For example, we excluded studies that made comparisons between different, differently sized eating utensils (e.g. straw versus spoon; chopsticks versus fork) whilst studies that made comparisons between different sizes of the same eating utensil were included (e.g. small spoon versus large spoon).
- Studies in which there were concurrent interventions unrelated to sizing that were intrinsically confounded with the comparison(s) of interest. For example, we excluded two‐arm studies in which one comparison group received a specified portion size and the other group received a smaller portion plus a concurrent nutritional labelling intervention.

#### Types of outcome measures

##### Primary outcomes

###### Behavioural endpoints

Eligible studies had to incorporate one or more measures of unregulated (ad libitum) consumption or selection (with or without purchasing) of food, alcohol or tobacco products. By unregulated, we refer to behaviour of participants that is not regulated by either explicit instructions or some other action of the researcher. Eligible studies may have measured consumption or selection in terms of quantities of manipulated products and/or quantities of non‐manipulated products. For example, a study investigating the effects of exposure to a large versus small portion of a pasta entrée, provided as part of a lunch meal, may have measured consumption in terms of energy intake from the entrée itself, or from a non‐manipulated vegetable side dish served with the entrée, or from the total lunch meal (that is, both manipulated and non‐manipulated components), or from all meals taken over the course of a whole day. Similarly, quantities consumed or selected may have been measured over a time period less than (immediate) or exceeding one day (longer‐term).

Our choice of eligible outcome constructs reflected a focus on the assessment of the effects of eligible interventions in terms of the types and amounts of food, alcohol and tobacco people consume, coupled with recognition that amount selected (with or without purchasing) is an important intermediate endpoint in pathways to consumption. We anticipated encountering a range of measures of these outcome constructs within included studies, and presented the following examples in the published protocol for this review.

####### 1. Consumption (intake) of a product

We assessed the amount of energy (e.g. calories), substances (e.g. carbon monoxide, alcohol, saturated fat), or products (e.g. food, drink or tobacco) consumed, measured in applicable natural units (e.g. kcals, kilojoules, grams). Objective measurement may involve calculating the amount of a product consumed by subtracting the amount remaining after consumption from the amount presented to the participant. Alternatively, it may involve direct observation of the individual by outcome assessors. Subjective measurement would involve participant self report.

####### 2. Selection of a product

a) Without purchase

b) With purchase

As per consumption, we assessed the amount of energy, substances or products selected for consumption, measured in applicable natural units. Depending on the study setting, a product may be selected with or without this act enjoining a purchase (that is, a transfer of money to the vendor).

###### Conceptual model

To supplement study eligibility criteria, we developed a provisional conceptual model that was published in the protocol for this review (Hollands 2014). This conceptual model was design‐oriented in the sense that its purpose was to help direct the review process by providing a simplified visual representation of the causal system of interest: the proposed causal pathway between eligible interventions and their outcomes (behavioural endpoints), and potential moderators of that relationship (effect modifiers) given that differential effects were plausible (Anderson 2011; Anderson 2013). We used the provisional conceptual model to inform the development of search strategies, data extraction forms and a provisional framework for the statistical analysis of outcome data collected from the eligible studies (see Search methods for identification of studies and Data collection and analysis). We iteratively revised the provisional conceptual model based on theory and evidence encountered in eligible studies during the course of the review process, and documented all revisions including the rationale for each revision and supporting evidence (see Data collection and analysis). We used the provisional and subsequent iterations of the conceptual model as a reference point for the design (in the protocol) and conduct (post‐protocol) of all stages of the systematic review up to and including data synthesis, and as a conceptual basis for explicit reporting of the methods and assumptions employed within the synthesis (Anderson 2013). In practice, iterative refinement of the conceptual model primarily involved incorporating further potential effect modifiers identified from theory and evidence presented in included study reports, which became candidates for consideration in the meta‐regression analysis (see Data collection and analysis). The final version of the conceptual model is shown Figure 1.

**1:**
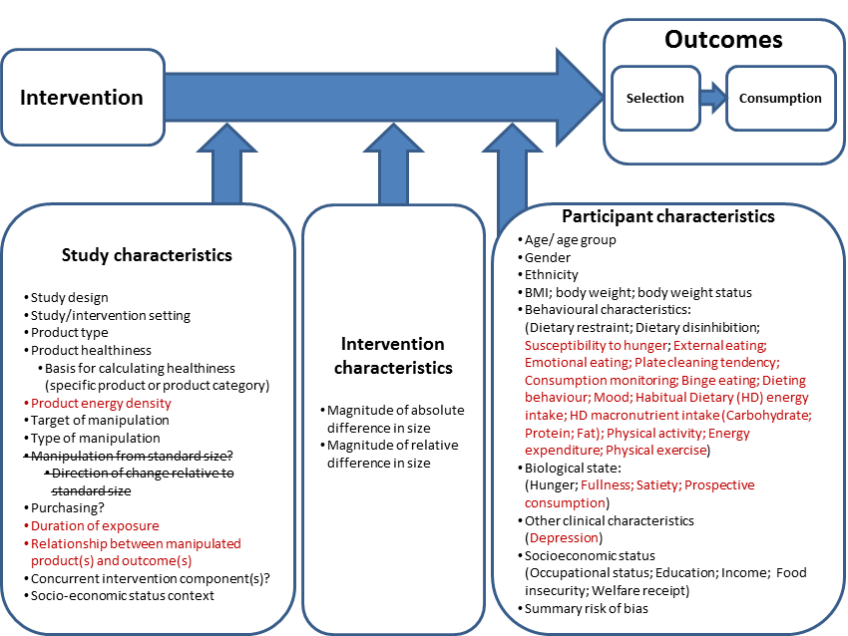
Final conceptual model. The 28 constructs included in the provisional conceptual model (Hollands 2014) and retained in this final version are shown in plain type. The 22 constructs added to this final conceptual model based on theory and evidence encountered during the review process are shown in red type. The 2 constructs included in the provisional conceptual model (Hollands 2014) but excluded from this final version are shown in strikethrough plain type. See Table 5 for a full record of the conceptual model development process.

Within the conceptual model (Figure 1) we distinguished between three sets of potential effect modifiers: study characteristics; intervention characteristics; and participant characteristics. Within our analytic framework for quantitative synthesis of outcome data collected from the included studies (see Data collection and analysis), potential effect‐modifying impacts of participant characteristics could in practice only be investigated based on between‐study comparisons, due to lack of reporting of results by participant subgroups within the included studies.

### Search methods for identification of studies

We initiated an original search, applying the methods described below in this section, in November 2012. We conducted an updated search, applying the same methods, prior to publication of the current version of the review, with a search date up to and including 30 January 2015. We have added eligible studies identified by the updated search (with subsequent title/abstract and full‐text screening) to Characteristics of studies awaiting classification, provisionally analysed them and will fully incorporate them into the review at the next update (see also Results of the search, Appendix 1 and Appendix 2).

#### Electronic searches

We conducted electronic searches for eligible studies within each of the following databases:

- Cochrane Central Register of Controlled Trials (CENTRAL 2015, Issue 1) (1992 to 30 January 2015);
- MEDLINE (OvidSP) (including MEDLINE In‐Process) (1946 to 30 January 2015);
- EMBASE (OvidSP) (1980 to 30 January 2015);
- PsycINFO (OvidSP) (1806 to 30 January 2015);
- Applied Social Sciences Index and Abstracts (ProQuest) (1987 to 30 January 2015);
- Food Science and Technology Abstracts (Web of Knowledge) (1969 to 22 November 2012);
- Science Citation Index Expanded (Web of Knowledge) (1900 to 30 January 2015);
- Social Sciences Citation Index (Web of Knowledge) (1956 to 30 January 2015);
- Trials Register of Promoting Health Interventions (EPPI Centre) (2004 to 30 January 2015).

We developed a MEDLINE search strategy by combining sets of controlled vocabulary and free‐text search terms based on the eligibility criteria described above (see Criteria for considering studies for this review). This was externally peer‐reviewed by an information retrieval specialist and Co‐convenor of the Cochrane Information Retrieval Methods Group and revised based on their peer‐review comments. We tested the MEDLINE search strategy for its sensitivity to retrieve a reference set of 48 records of reports of potentially eligible studies known to be indexed in MEDLINE that were identified by our preceding scoping review (Hollands 2013a). We adapted the final MEDLINE search strategy for use to search each of the other databases listed above based on close examination of database thesauri and scope notes if available. We imposed no restrictions for publication date, publication format or language and incorporated no study design filters. Full details of final search strategies for each database, along with search dates and yields (for both the original search and the updated search), are provided in Appendix 1.

#### Searching other resources

We conducted electronic searches of two grey literature resources using search strategies adapted from the final MEDLINE search strategy:

- Conference Proceedings Citation Index ‐ Science (Web of Knowledge) (1990 to 30 January 2015);
- Conference Proceedings Citation Index ‐ Social Science & Humanities (Web of Knowledge) (1990 to 30 January 2015);
- Open Grey ‐ www.opengrey.eu (1980 to 30 January 2015).

We also searched trial registers (ClinicalTrials.gov and the World Health Organization International Clinical Trials Registry Platform (ICTRP)) to identify registered trials, and the websites of the following key organisations in the area of health and nutrition:

- Centers for Disease Control and Prevention, USA;
- EU Platform for Action on Diet, Physical Activity and Health;
- International Obesity Task Force;
- Rudd Centre for Food Policy and Obesity, USA;
- UK Department of Health;
- World Health Organization.

In addition, we searched the reference lists of all eligible study reports that had been identified using the other search methods described above and undertook forward citation tracking (using Google Scholar and PubMed) to identify further eligible studies or study reports.

### Data collection and analysis

#### Selection of studies

We imported title‐abstract records retrieved by the electronic searches to EPPI Reviewer 4 (ER4) systematic review software (Thomas 2010). We identified, reviewed manually and removed duplicate records using ER4's automatic de‐duplication feature with the similarity threshold set initially to 0.85 and finally to 0.80 following satisfactory manual checks of incomplete duplicate groups. Two researchers working independently (GJH, IS) undertook duplicate screening of title‐abstract records. We coded title‐abstract records as 'provisionally eligible', 'excluded' or 'duplicate' by applying the eligibility criteria described above (see Criteria for considering studies for this review). Disagreements in the coding of title‐abstract records were identified and resolved by discussion to reach consensus between the two researchers (GJH, IS).

We obtained copies of corresponding full‐text study reports for all title‐abstract records coded as 'provisionally eligible'. Two researchers working independently (GJH, IS) undertook duplicate screening of full‐text study reports. We coded full‐text study reports as 'eligible' or 'excluded' by applying the eligibility criteria described above (see Criteria for considering studies for this review). Coding disagreements were again identified and resolved by discussion to reach consensus between the two researchers, with a third researcher (DO) acting as arbiter when needed. We recorded bibliographic details of study reports excluded at the full‐text screening stage, along with the primary reason for exclusion, in a Characteristics of excluded studies table. We identified and linked multiple full‐text reports of the same study. We also identified full‐text reports comprising multiple eligible studies. We documented the flow of records and studies through the systematic review process using a PRISMA flow diagram (Moher 2009).

#### Data extraction and management

We developed an electronic data extraction form based on the Cochrane Public Health Review Group's template (http://ph.cochrane.org/review‐authors). We piloted an initial draft form using a selection of 10 included studies and then amended this in consultation with other members of the review team. One researcher (GJH or IS) extracted data on characteristics of included studies, while two researchers working independently (GJH, IS) extracted outcome data in duplicate. We only collected outcome data relating to comparison groups eligible for consideration in this review, but Characteristics of included studies tables record details of all study arms (conditions). Discrepancies in extracted outcome data were identified and resolved by checking against the study report, discussion and consensus between two researchers (GJH, IS). We sought key data missing from reports of included studies by contacting study authors.

At the protocol stage, we intended to collect the data summarised immediately below in this section. This represented the core data set (comprising 28 pre‐specified moderator constructs for potential examination using meta‐regression analyses; see Data synthesis) that we could reasonably anticipate would need to be collected based on our study eligibility criteria (see Criteria for considering studies for this review) and provisional conceptual model (Hollands 2014).

##### Study characteristics

- Study design: between‐subjects design, within‐subjects design
- Study (intervention) setting: laboratory, field; for consumption at home or away from home
- Product type: food (including non‐alcoholic beverages), alcohol, tobacco
- Product healthiness: Food Standards Agency (FSA) score (Rayner 2005) at level of specific product or, if not possible, at level of product category
- Target of manipulation: portion, package, individual unit, tableware
- Type of manipulation: size (including volume) or shape
- Manipulation from a standard size: no or yes*
- If applicable, direction of the change relative to standard size: smaller or larger*
- If applicable, selection with purchasing or selection without purchasing
- Concurrent intervention components (e.g. nutritional labelling)
- Socioeconomic status context (low, high)

##### Intervention characteristics

- Magnitude of the absolute difference in size (e.g. difference in quantity): smaller size always coded as Intervention 1 and larger size as Intervention 2
- Magnitude of the relative difference in size (e.g. percentage difference in quantity): smaller size always coded as Intervention 1 and larger size as Intervention 2

##### Participant characteristics

- Age/age group
- Gender: male, female
- Ethnicity
- Body mass index (BMI); body weight; body weight status
- Behavioural characteristics (e.g. dietary restraint; susceptibility to hunger)
- Biological state (e.g. hunger)
- Other clinical characteristics (e.g. morbidities such as cardiovascular diseases, diabetes, psychiatric disorders)
- Socioeconomic status (e.g. occupational status; education; income; food insecurity; welfare receipt)
- Summary risk of bias

These participant characteristics cover several categories of social differentiation relevant to health equity, namely: age, ethnicity, gender, occupation, education, income and other proxy measures of socioeconomic status. The incorporation of study‐level data on these participant characteristics into our proposed meta‐regression analysis (see 'Data synthesis') was in part intended to enable us to interpret any differential effects through a health equity lens (Welch 2012) (see also Objectives 2c).

As anticipated, our conceptual model ‐ and consequently the core data set ‐ evolved as the review process progressed. First, we excluded a pair of potential effect modifiers (study characteristics) included in our *provisional* conceptual model that express studied portion size manipulations relative to a standard size (see asterisked characteristics '*' in the list of 'Study characteristics', above), since it was not judged feasible to define standard sizes based on information reported in included studies. Second, the process of collecting data from included studies identified 22 additional potential effect modifiers (moderator constructs) that were added to the conceptual model. These additional constructs were included in the current, published review version of the conceptual model (Figure 1) and are listed below:

##### Study characteristics

- Product energy density
- Duration of exposure
- Relationship between manipulated product(s) and outcome(s)

##### Intervention characteristics

None added.

##### Participant characteristics

- Behavioural characteristics (susceptibility to hunger; external eating; emotional eating; plate cleaning tendency; consumption monitoring; binge eating; dieting behaviour; mood; habitual dietary energy intake; habitual dietary macronutrient intake (carbohydrate; protein; fat); physical activity; energy expenditure; physical exercise)
- Biological state (fullness; satiety; prospective consumption)
- Other clinical characteristics (depression)

We coded 28 variables that measured these constructs from included studies (as well as coding 43 variables that measured constructs included in the initial conceptual model). The current, published review version of our conceptual model (Figure 1) therefore comprised 48 moderator constructs, with 72 corresponding variables, for potential examination using meta‐regression analyses. Table 5 traces this iterative conceptual model development process, documenting all revisions made between the protocol (Hollands 2014) and final versions (Figure 1), together with the rationale and supporting evidence for each revision.

**1:** Record of conceptual model development

**Construct**	**Variable description (type)**	**Category**	**Included in provisional conceptual model?**	**Included in final conceptual model?**	**Included study first encountered**	**Other included studies encountered**	**Rationale for inclusion in final conceptual model**	**Supporting evidence**	**Rationale for exclusion from final conceptual model**
Study design	Randomised controlled trial or cluster‐randomised controlled trial (Categorical, dichotomous)	Study characteristic	Yes	Yes	N/A	N/A	N/A	N/A	N/A
Study design	Between subjects or within‐subjects design (Categorical, dichotomous)	Study characteristic	Yes	Yes	N/A	N/A	N/A	N/A	N/A
Study/ intervention setting	Laboratory or field setting (Categorical, dichotomous)	Study characteristic	Yes	Yes	N/A	N/A	N/A	N/A	N/A
Study/ intervention setting	Selecting/consuming alone or selecting/consuming with others	Study characteristic	Yes	Yes	N/A	N/A	N/A	N/A	N/A
Product type	Food or tobacco (or alcohol ‐ no studies) (Categorical, dichotomous)	Study characteristic	Yes	Yes	N/A	N/A	N/A	N/A	N/A
Healthiness of manipulated product(s) (food products only)	FSA Nutrient Profile Score (Continuous)	Study characteristic	Yes	Yes	N/A	N/A	N/A	N/A	N/A
Basis for calculating healthiness of manipulated product(s) (food products only)	Specific product or product category (Categorical, dichotomous)	Study characteristic	Yes	Yes	N/A	N/A	N/A	N/A	N/A
Energy density of manipulated product(s) (food products only)	Energy density points from FSA Nutrient Profile model (Continuous)	Study characteristic	No	Yes	Devitt 2004 (study includes concurrent manipulation of energy density)	Kral 2004a, Fisher 2007b, Leahy 2008, Looney 2011, Rolls 2006b (studies include concurrent manipulation of energy density)	Evidence from previous studies that the energy density of food can exert independent and combined influences on energy intake suggests that this has the potential to modify any effects of larger portions, packages, individual units or tableware on the selection and consumption of food	Kral 2004a, Kral 2004b, Rolls 2009, Bell 1998, Rolls 1999, Rolls 2006b	N/A
Target of manipulation	Portion, package, individual unit, package with individual unit, or tableware (Categorical, nominal)	Study characteristic	Yes	Yes	N/A	N/A	N/A	N/A	N/A
Type of manipulation	Size (including volume) or shape manipulation (Categorical, dichotomous)	Study characteristic	Yes	Yes	N/A	N/A	N/A	N/A	Post‐hoc decision taken to conduct separate meta‐analyses for size and shape since comparisons of size were not judged conceptually comparable to comparisons of shape among the set of studies included in this review Therefore no longer conceptualised as a potential effect modifier
Manipulation from a standard size	No or yes (Categorical, dichotomous)	Study characteristic	Yes	No	N/A	N/A	N/A	N/A	In practice it was rarely possible to code this variable based on information in study reports, and not judged practicable to code with reference to data from external sources
If applicable, direction of the change relative to standard size	Smaller or larger (Categorical, dichotomous)	Study characteristic	Yes	No	N/A	N/A	N/A	N/A	In practice it was rarely possible to code this variable based on information in study reports, and not judged practicable to code with reference to data from external sources
Selection without purchasing or selection with purchasing	Selection without purchasing or selection with purchasing (Categorical, dichotomous)	Study characteristic	Yes	Yes	N/A	N/A	N/A	N/A	N/A
Duration of exposure to the intervention	≤ 1 day or > 1 day (Categorical, dichotomous)	Study characteristic	No	Yes	N/A – Added based on discussion of collected data between 2 review authors (GJH and IS, April 2014), which identified duration of exposure as a variant characteristic of included studies (in addition to timing of outcome measurement, which had been included in our provisional conceptual model)	N/A	Duration of exposure to larger portions, packages, individual units or tableware has the potential to modify any effects of such exposure on the selection and consumption of food	Rolls 2006a, Rolls 2007a	N/A
Relationship between manipulated product(s) and consumption/ selection outcomes (food products only)	The manipulated foods comprise all of those in the study and all are selected or consumed ad libitum (Dummy)	Study characteristic	No	Yes	N/A – Added based on discussion of collected data between 2 review authors (GJH and IS, April 2014), which identified duration of exposure as a variant characteristic of included studies	N/A	This relationship may have the potential to modify any effects of such exposure on the selection and consumption of food. This is because providing any additional foods for consumption beyond those manipulated may result in additional energy consumption in either or both conditions. Given potential ceiling effects on total consumption, this could modify any intervention effect	‐	N/A
Relationship between manipulated product(s) and consumption/ selection outcomes (food products only)	The manipulated foods are only a subset of all the foods in the study and there are other non‐manipulated foods that are compulsory to select or consume (Dummy)	Study characteristic	No	Yes	N/A – Added based on discussion of collected data between 2 review authors (GJH and IS, April 2014), which identified duration of exposure as a variant characteristic of included studies	N/A	This relationship may have the potential to modify any effects of such exposure on the selection and consumption of food. This is because providing compulsory additional foods beyond those manipulated would result in additional energy consumption in both conditions. Given potential ceiling effects on total consumption, this could attenuate any intervention effect	‐	N/A
Relationship between manipulated product(s) and consumption/ selection outcomes (food products only)	The manipulated foods are only a subset of all the foods in the study and there are other non‐manipulated foods in study that are selected or consumed ad libitum (Dummy)	Study characteristic	No	Yes	N/A – Added based on discussion of collected data between 2 review authors (GJH and IS, April 2014), which identified duration of exposure as a variant characteristic of included studies	N/A	This relationship may have the potential to modify any effects of such exposure on the selection and consumption of food. This is because providing additional foods to be consumed ad libitum beyond those manipulated may result in additional energy consumption in either or both conditions. Given potential ceiling effects on total consumption, this could modify any intervention effect	‐	N/A
Relationship between manipulated product(s) and consumption/ selection outcomes (food products only)	Outcome data maps directly onto the manipulated food(s) (as opposed to a wider set of foods, including but not limited to manipulated food(s)) (Dummy)	Study characteristic	No	Yes	N/A – Added based on discussion of collected data between 2 review authors (GJH and IS, April 2014), which identified duration of exposure as a variant characteristic of included studies	N/A	This relationship may have the potential to modify any effects of such exposure on the selection and consumption of food. This is because including any additional foods in outcome measurement beyond those manipulated may result in additional energy consumption being measured in either or both conditions	‐	N/A
Concurrent intervention component(s)	Absent or present (Categorical, dichotomous)	Study characteristic	Yes	Yes	N/A	N/A	N/A	N/A	N/A
Socio‐economic status context	Low deprivation or high deprivation (Categorical, dichotomous)	Study characteristic	Yes	Yes	N/A	N/A	N/A	N/A	N/A
Magnitude of the absolute difference in size	Difference between larger size and smaller size in grams (Continuous)	Intervention characteristic	Yes	Yes	N/A	N/A	N/A	N/A	N/A
Magnitude of the relative difference in size	Larger size expressed as a proportion (%) of smaller size (Continuous)	Intervention characteristic	Yes	Yes	N/A	N/A	N/A	N/A	N/A
Age	Average (mean) age in years among study completers (Continuous)	Participant characteristic	Yes	Yes	N/A	N/A	N/A	N/A	N/A
Gender	Proportion (%) of study completers who were female (Continuous)	Participant characteristic	Yes	Yes	N/A	N/A	N/A	N/A	N/A
Ethnicity	Proportion (%) of study completers of white ethnicity (Continuous)	Participant characteristic	Yes	Yes	N/A	N/A	N/A	N/A	N/A
Body mass index (BMI)	Average (mean) BMI among study completers (Continuous)	Participant characteristic	Yes	Yes	N/A	N/A	N/A	N/A	N/A
Body mass index (BMI)	Average (mean) BMI‐z score among study completers (Continuous)	Participant characteristic	Yes	Yes	N/A	N/A	N/A	N/A	N/A
Body weight	Average (mean) weight in kilograms among study completers (Continuous)	Participant characteristic	Yes	Yes	N/A	N/A	N/A	N/A	N/A
Body weight status	Average (mean) percentage (%) body fat among study completers (Continuous)	Participant characteristic	Yes	Yes	N/A	N/A	N/A	N/A	N/A
Body weight status	Proportion (%) of study completers who were overweight (Continuous)	Participant characteristic	Yes	Yes	N/A	N/A	N/A	N/A	N/A
Body weight status	Proportion (%) of study completers who were obese (Continuous)	Participant characteristic	Yes	Yes	N/A	N/A	N/A	N/A	N/A
Body weight status	Proportion (%) of study completers who were overweight or obese (Continuous)	Participant characteristic	Yes	Yes	N/A	N/A	N/A	N/A	N/A
Behavioural characteristics: dietary restraint	Average (mean) dietary restraint score among study completers ‐ Three Factor Eating Questionnaire (Stunkard 1985) (Continuous)	Participant characteristic	Yes	Yes	N/A	N/A	N/A	N/A	N/A
Behavioural characteristics: dietary restraint	Average (mean) dietary restraint score among study completers ‐ Dutch Eating Behaviour Questionnaire (Van Strien 1986) (Continuous)	Participant characteristic	Yes	Yes	N/A	N/A	N/A	N/A	N/A
Behavioural characteristics: dietary restraint	Average (mean) dietary restraint score among study completers ‐ Restraint Scale (Herman 1980) (Continuous)	Participant characteristic	Yes	Yes	N/A	N/A	N/A	N/A	N/A
Behavioural characteristics: dietary disinhibition	Average (mean) dietary disinhibition score among study completers ‐ Three Factor Eating Questionnaire (Stunkard 1985) (Continuous)	Participant characteristic	Yes	Yes	N/A	N/A	N/A	N/A	N/A
Behavioural characteristics: dietary disinhibition	Average (mean) dietary disinhibition score among study completers ‐ Dutch Eating Behaviour Questionnaire (Van Strien 1986) (Continuous)	Participant characteristic	Yes	Yes	N/A	N/A	N/A	N/A	N/A
Behavioural characteristics: external eating	Average (mean) external eating score among study completers ‐ Dutch Eating Behaviour Questionnaire (Van Strien 1986) (Continuous)	Participant characteristic	No	Yes	Hermans 2012	Kelly 2009, Kral 2004a	External eating (which measures the tendency to eat in response to external food‐related cues such as the sight, taste, and smell of attractive food) has the potential to modify any effects of larger portions, packages, individual units or tableware on the selection and consumption of food	Herman 2008, Burton 2007, Rodin 1981	N/A
Behavioural characteristics: emotional eating	Average (mean) emotional eating score among study completers ‐ Dutch Eating Behaviour Questionnaire (Van Strien 1986) (Continuous)	Participant characteristic	No	Yes	Kelly 2009	Kral 2004a	Emotional eating (which measures the tendency to eat in response to emotions such as anxiety, disappointment or boredom) has the potential to modify any effects of larger portions, packages, individual units or tableware on the selection and consumption of food	Van Strien 1986, Wallis 2009	N/A
Behavioural characteristics: susceptibility to hunger	Average (mean) hunger score among study completers – Three factor eating questionnaire (Stunkard 1985) (Continuous)	Participant characteristic	No	Yes	Flood 2006	Kral 2004a, Rolls 2002, Rolls 2004a, Rolls 2004b, Rolls 2006a, Rolls 2006b, Rolls 2007a, Rolls 2007b (S1), Rolls 2007b (S2), Rolls 2007b (S3), Rolls 2010a (E1), Rolls 2010b (E2)	Susceptibility to hunger (predisposition to feelings of hunger) has the potential to modify any effects of larger portions, packages, individual units or tableware on the selection and consumption of food	Provencher 2003, Lindroos 1997	N/A
Behavioural characteristics: plate cleaning tendency	Average (mean) plate cleaning tendency score among study completers ‐ 7‐point agreement scale anchored (‐3) strongly disagree and (+3) strongly agree (Marchiori 2012a, Wansink 2005e) (Continuous)	Participant characteristic	No	Yes	Marchiori 2012a	‐	Plate cleaning tendency (the tendency for a person to consume all the food presented to them) has the potential to modify any effects of larger portions, packages, individual units or tableware on the selection and consumption of food	Wansink 2005e	N/A
Behavioural characteristic: plate cleaning tendency	Behavioural characteristic ‐ Proportion (%) of adult study completers who often or always clean the plate (Continuous)	Participant characteristic	No	Yes	Rolls 2004a	‐	Plate cleaning tendency (the tendency for a person to consume all the food presented to them) has the potential to modify any effects of larger portions, packages, individual units or tableware on the selection and consumption of food	Wansink 2005e	N/A
Behavioural characteristic: plate cleaning tendency	Behavioural characteristic ‐ Proportion (%) of child study completers who often or always clean the plate (Continuous)	Participant characteristic	No	Yes	Rolls 2004a	‐	Plate cleaning tendency (the tendency for a person to consume all the food presented to them) has the potential to modify any effects of larger portions, packages, individual units or tableware on the selection and consumption of food	Wansink 2005e	N/A
Behavioural characteristics: consumption monitoring	Average (mean) consumption monitoring score among study completers ‐ 7‐point agreement scale anchored (‐3) strongly disagree and (+3) strongly agree (Continuous)	Participant characteristic	No	Yes	Marchiori 2012a	‐	Consumption monitoring (the tendency for a person to pay attention to and monitor the food they are consuming) has the potential to modify any effects of larger portions, packages, individual units or tableware on the selection and consumption of food	Polivy 1986	N/A
Behavioural characteristics: binge eating	Average (mean) binge eating score among study completers ‐ Eating Disorders Examination (Fairburn 1993) (Continuous)	Participant characteristic	No	Yes	Marchiori 2012a	‐	Binge eating (discrete episodes of eating during which the amount consumed is unusually large and there is a sense of loss of control over eating at the time) has the potential to modify any effects of larger portions, packages, individual units or tableware on the selection and consumption of food	Fairburn 1993	N/A
Behavioural characteristics: binge eating	Average (mean) binge eating score among study completers – Binge Eating Questionnaire (Gormally 1982) (Continuous)	Participant characteristic	No	Yes	Stroebele 2009	‐	Binge eating (discrete episodes of eating during which the amount consumed is unusually large and there is a sense of loss of control over eating at the time (Fairburn 1993)) has the potential to modify any effects of larger portions, packages, individual units or tableware on the selection and consumption of food	Fairburn 1993, Cooper 2003	N/A
Behavioural characteristics: dieting behaviour	Average (mean) dieting behavior score – Eating Attitude Test (EAT‐26) (Garner 1982) (Continuous)	Participant characteristic	No	Yes	Marchiori 2012a	Rolls 2002, Rolls 2004a, Rolls 2004b, Rolls 2007a	Dieting behaviour (behaviour that involves a person restricting themselves to smaller amounts or specific types of food either to lose weight or for medical reasons) has the potential to modify any effects of larger portions, packages, individual units or tableware on the selection and consumption of food	Van Strien 1986, Stunkard 1985	N/A
Behavioural characteristics: mood	Average (mean) mood score among study completers ‐ 7‐point agreement scale anchored (‐3) strongly disagree and (+3) strongly agree (Marchiori 2012a, Reinbach 2010) (Continuous)	Participant characteristic	No	Yes	Marchiori 2012a	‐	Mood has the potential to modify any effects of larger portions, packages, individual units or tableware on the selection and consumption of food	Gardner 2014	N/A
Behavioural characteristics: habitual dietary energy intake	Average (mean) dietary energy intake per diem among study completers in kcal (Continuous)	Participant characteristic	No	Yes	Ahn 2010	Ebbeling 2007	Baseline level of dietary energy intake has the potential to modify any effects of larger portions, packages, individual units or tableware on the selection and consumption of food	Fyfe 2010, Birch 1991	N/A
Behavioural characteristics: habitual dietary macronutrient intake, Carbohydrate	Average (mean) carbohydrate intake as a proportion (%) of daily energy intake among study completers (Continuous)	Participant characteristic	No	Yes	Ahn 2010	‐	Baseline levels of macronutrient intake have the potential to modify any effects of larger portions, packages, individual units or tableware on the selection and consumption of food	Beasley 2009, Monteleone 2003, Yeomans 2001, Rolls 1988	N/A
Behavioural characteristics: habitual dietary macronutrient intake, Protein	Average (mean) protein intake as a proportion (%) of daily energy intake among study completers (Continuous)	Participant characteristic	No	Yes	Ahn 2010	‐	Baseline levels of macronutrient intake have the potential to modify any effects of larger portions, packages, individual units or tableware on the selection and consumption of food	Beasley 2009, Rolls 1988	N/A
Behavioural characteristics: habitual dietary macronutrient intake, Fat	Average (mean) fat intake as a proportion (%) of daily energy intake among study completers (Continuous)	Participant characteristic	No	Yes	Ahn 2010	‐	Baseline levels of macronutrient intake have the potential to modify any effects of larger portions, packages, individual units or tableware on the selection and consumption of food	Beasley 2009, Brennan 2012, Monteleone 2003, Yeomans 2001, Rolls 1988	N/A
Behavioural characteristics: physical activity	Average (mean) daily total number of steps among study completers (Continuous)	Participant characteristic	No	Yes	Ahn 2010	‐	Baseline levels of physical activity have the potential to modify any effects of larger portions, packages, individual units or tableware on the selection and consumption of food	Martins 2007	N/A
Behavioural characteristics: habitual energy expenditure	Average (mean) daily energy expenditure among study completers in kcal (Continuous)	Participant characteristic	No	Yes	Rolls 2006a	Rolls 2006b, Rolls 2007a, Rolls 2007b (S1), Rolls 2007b (S2), Rolls 2007b (S3), Rolls 2010a (E1), Rolls 2010b (E2)	Baseline levels of energy expenditure have the potential to modify any effects of larger portions, packages, individual units or tableware on the selection and consumption of food	Martins 2007	N/A
Behavioural characteristics: habitual physical exercise	Average (mean) number of hours of physical exercise completed among study completers per week (Continuous)	Participant characteristic	No	Yes	Marchiori 2012c	‐	Baseline levels of physical exercise have the potential to modify any effects of larger portions, packages, individual units or tableware on the selection and consumption of food	Martins 2007	N/A
Biological state: hunger	Average (mean) hunger rating among study completers – 100 mm visual analogue scale (Continuous)	Participant characteristic	Yes	Yes	N/A	N/A	N/A	N/A	N/A
Biological state: hunger	Average (mean) hunger rating among study completers ‐ 3‐point rating scale (Continuous)	Participant characteristic	Yes	Yes	N/A	N/A	N/A	N/A	N/A
Biological state: hunger	Average (mean) hunger rating among study completers ‐ 7‐point rating scale (Continuous)	Participant characteristic	Yes	Yes	N/A	N/A	N/A	N/A	N/A
Biological state: appetitive state, Fullness	Average (mean) fullness rating among study completers – 100 mm visual analogue scale (Continuous)	Participant characteristic	No	Yes	Shah 2011	‐	Baseline levels of feelings of fullness (specific somatic sensation or perceived general state of fullness (Blundell 2010)) have the potential to modify any effects of larger portions, packages, individual units or tableware on the selection and consumption of food	Doucet 2008	N/A
Biological state: appetitive state, Satiety	Average (mean) satiety rating among study completers – 100 mm visual analogue scale (Continuous)	Participant characteristic	No	Yes	Shah 2011	‐	Baseline levels of feelings of satiety (specific somatic sensation or perceived general state of being satiated (Blundell 2010)) have the potential to modify any effects of larger portions, packages, individual units or tableware on the selection and consumption of food	Lemmens 2011	N/A
Biological state: appetitive state, Prospective consumption	Average (mean) prospective consumption rating among study completers – 100 mm visual analogue scale (Continuous)	Participant characteristic	No	Yes	Shah 2011	‐	Baseline levels of prospective consumption (how much participants felt they could eat now (Shah 2011)) have the potential to modify any effects of larger portions, packages, individual units or tableware on the selection and consumption of food	Doucet 2008	N/A
Other clinical characteristics: depression	Average (mean) depression score among study completers ‐ Zung Depression Inventory (Zung 1986) (Continuous)	Participant characteristic	No	Yes	Rolls 2002	Rolls 2004a, Rolls 2004b, Rolls 2007a	Baseline feelings of depression (or of affective, psychological or somatic symptoms associated with depression) have the potential to modify any effects of larger portions, packages, individual units or tableware on the selection and consumption	Grossniklaus 2010	N/A
Socioeconomic status: occupational status	Proportion (%) of study completers in employment (Continuous)	Participant characteristic	Yes	Yes	N/A	N/A	N/A	N/A	N/A
Socioeconomic status: occupational status	Proportion (%) of study completers with a parent or caregiver in employment (Continuous)	Participant characteristic	Yes	Yes	N/A	N/A	N/A	N/A	N/A
Socioeconomic status: education	Average (mean) number of years of education completed among study completers (Continuous)	Participant characteristic	Yes	Yes	N/A	N/A	N/A	N/A	N/A
Socioeconomic status: education	Proportion (%) of study completers who completed at least some further education (greater than high school, at least some college) (Continuous)	Participant characteristic	Yes	Yes	N/A	N/A	N/A	N/A	N/A
Socioeconomic status: education	Proportion (%) of study completers with a parent or caregiver who completed at least some further education (greater than high school, at least some college) (Continuous)	Participant characteristic	Yes	Yes	N/A	N/A	N/A	N/A	N/A
Socioeconomic status: education	Proportion (%) of study completers with a parent or caregiver who completed at least a 4‐year university degree (Continuous)	Participant characteristic	Yes	Yes	N/A	N/A	N/A	N/A	N/A
Socioeconomic status: income	Proportion (%) of study completers with an individual income > USD 50,000 (Continuous)	Participant characteristic	Yes	Yes	N/A	N/A	N/A	N/A	N/A
Socioeconomic status: income	Proportion (%) of study completers with a total family income > USD 50,000 (Continuous)	Participant characteristic	Yes	Yes	N/A	N/A	N/A	N/A	N/A
Other measures of socioeconomic status: food insecurity	Proportion (%) of study completers living in a food insecure household (Continuous)	Participant characteristic	Yes	Yes	N/A	N/A	N/A	N/A	N/A
Other measures of socioeconomic status: welfare receipt	Proportion (%) of study completers participating in the US National School Lunch Program (Continuous)	Participant characteristic	Yes	Yes	N/A	N/A	N/A	N/A	N/A
Other measures of socioeconomic status: welfare receipt	Proportion (%) of study completers participating in the US School Nutrition Assistance Program (Continuous)	Participant characteristic	Yes	Yes	N/A	N/A	N/A	N/A	N/A
Overall (summary) risk of bias	Low risk, unclear risk or high risk (Categorical, nominal)	Participant characteristic	Yes	Yes	N/A	N/A	N/A	N/A	N/A

##### Outcome data

As anticipated, eligible primary studies frequently included more than one measure of each target outcome construct, specifically: (a) more than one measure of selection for a given comparison, (b) more than one measure of consumption for a given comparison, or both. For each included study in which (a) or (b) applied, we extracted outcome data for use in meta‐analysis for the (a) primary selection or (b) primary consumption outcome(s) as (pre‐)specified by the study authors. If the study authors did not (pre‐)specify a single (primary) (a) selection or (b) consumption outcome, we applied the following criteria to select the (a) selection or (b) consumption measure for which outcome data would be extracted for use in meta‐analysis from a list of all available measures. We selected the measure of (a) selection or (b) consumption most proximal to health outcomes in the context of the specific intervention at hand. For example, if a study reported measures of both energy intake and the amount of food eaten (in grams), we selected energy intake as the measure of the target outcome construct most proximal to diet‐related health outcomes. We also selected the largest‐scale measure of the target outcome construct. For example, if a study manipulated the size of a portion of vegetable served as one component of a plated entrée, and measured the effects of a large versus a small vegetable portion size in terms of: (i) the amount of that vegetable consumed from the plated entrée, and (ii) the total amount of food consumed from the plated entrée, then we selected (ii) as the consumption outcome measure for which we extracted data. We made each selection in advance of data extraction, blinded to the outcome data. We recorded details of selection and consumption outcomes measures available in each included study and documented these in Characteristics of included studies.

For included studies that investigated a size manipulation, we always coded exposure to the larger of the two portions, packages, individual units or items of tableware as the intervention, whilst we always coded exposure to the smaller of the two as the comparator. For included studies that investigated a shape manipulation, we always coded exposure to the shorter, wider of the two items of tableware as the intervention, whilst we always coded exposure to the taller, narrower of the two as the comparator.

For all outcome data we collected information on: outcome variable type (in practice, this was invariably continuous); outcome variable definition; unit of measurement (natural units); specific metric (final values, change from baseline); method of aggregation (mean); timing of measurement (immediate (that is, ≤ 1 day) or longer‐term (that is, > 1 day)); and type of measure (objective, self report). For continuous outcomes, we extracted mean differences, or mean changes in final measurements from baseline measurements, for each comparison group along with associated standard deviations (or, if standard deviations were missing, standard errors, 95% confidence intervals or relevant t‐statistics, f‐statistics or exact P values that we used to calculate standard deviations); we also indicated whether a high or low value is favourable from a public health perspective. For included studies with factorial designs, we combined comparison groups so that any independent or interactive effects of the co‐occurring manipulation were averaged across the comparison groups of interest, in order to allow investigation of the independent effects of the size or shape manipulation.

#### Assessment of risk of bias in included studies

We assessed risk of bias in the included studies using the Cochrane 'Risk of bias' tool addressing eight specific domains, namely: random sequence generation and allocation concealment (selection bias); blinding of participants and personnel (performance bias); blinding of outcome assessors (detection bias); incomplete outcome data (attrition bias); selective outcome reporting (reporting bias); and baseline comparability of participant characteristics between groups and consistency in intervention delivery (other bias) (Higgins 2011b). The last domain refers to whether information and specific instructions provided to participants were standardised between conditions and whether participant (non‐)compliance with the study protocol was appropriately managed.

Two researchers working independently (GJH, IS) applied the Cochrane 'Risk of bias' tool to each included study. We recorded supporting information for judgements of risk of bias (high, low or unclear) in the form of verbatim text extracted from study reports, supplemented with reviewer comments. We identified and resolved discrepancies between the two researchers' judgements or supporting information by discussion to reach consensus. We derived a summary risk of bias judgement (high, low or unclear) for each specific outcome, for inclusion as a study‐level covariate in the final stage of the meta‐regression analysis (see Data synthesis). We also considered summary risk of bias in determining the strength of inferences drawn from the results of the data synthesis and in developing conclusions and recommendations concerning the design and conduct of future research. We derived the summary risk of bias judgement from the four domains judged to be most critical in this specific review, namely: random sequence generation (selection bias); allocation concealment (selection bias); blinding of participants and personnel (performance bias); and baseline comparability of participant characteristics between groups (other bias). It was derived using an algorithm suggested in Section 8.7 (Table 8.7a) of the *Cochrane Handbook for Systematic Reviews of Interventions* (Higgins 2011b). Specifically, if the judgement in at least one of these four domains was 'high risk of bias' then we determined summary risk of bias to be high. If no judgements of 'high' risk were made in these four domains, but the judgement in at least one of these domains was 'unclear risk of bias' then we determined the summary risk of bias to be unclear. We only judged summary risk of bias 'low' if judgements in all four of these domains were 'low risk of bias'.

#### Measures of treatment effect

We calculated the standardised mean difference (SMD) with 95% confidence intervals to express the size of the intervention effect in each study relative to the variability observed in that study. We classified included study results according to two categories of timing of outcome measurement: immediate outcomes (that is ≤ 1 day) versus longer‐term outcomes (that is > 1 day).

#### Unit of analysis issues

In the case of cluster‐randomised controlled trials, where an analysis was reported that accounted for the clustered study design, we estimated the effect on this basis. Where this was not possible and the information was not available from the authors, then we carried out an 'approximately correct' analysis according to current guidelines (Higgins 2011a). We imputed estimates of the intra‐cluster correlation (ICC) using estimates derived from similar studies included in the review. We also computed inflated standard errors for outcome data from cluster‐randomised controlled trials based on reported test statistics (f values, t values or P values) and used these data in all statistical analyses. Where test statistics were not available, we imputed inflated standard errors from unadjusted standard errors based on ratios of adjusted to unadjusted standard errors obtained from similar studies included in the review.

For included studies with a within‐subjects design, we calculated the standardised mean difference for continuous outcomes using the methods described in Section 16.4 of the *Cochrane Handbook for Systematic Reviews of Interventions* (Higgins 2011a). Similar to our approach for cluster‐randomised controlled trials, we sought to compute deflated standard errors for outcome data from studies with a within‐subjects design based on reported test statistics, or on ratios of inflated to unadjusted standard errors obtained from similar studies included in the review. However, in studies with a within‐subjects design, these ratios exceeded one, which is counter‐intuitive and suggests there was no statistical advantage in using within‐subjects designs in this area. We therefore reverted to use of unadjusted standard errors for studies with a within‐subjects design in all statistical analyses.

Final outcome values served as the primary unit of analysis. Only one included study reported outcome data using changes from baseline as the metric (Ahn 2010). For this study we computed final values based on reported data, supplemented with additional information supplied by the authors.

#### Dealing with missing data

Where data were missing due to participant dropout we conducted available case analyses and recorded any issues of missing data within the assessments conducted using the Cochrane 'Risk of bias' tool.

#### Assessment of heterogeneity

We assessed statistical heterogeneity in results by inspection of a graphical display of the estimated treatment effects from included studies along with their 95% confidence intervals, and by formal statistical tests of homogeneity (Chi^2^) and measures of inconsistency (I^2^) and heterogeneity (τ^2^).

#### Assessment of reporting biases

We drew funnel plots (plots of effect estimates versus the inverse of their standard errors) to inform assessment of reporting biases. We conducted statistical tests to formally investigate the degree of asymmetry using the method proposed by Egger et al (Egger 1997). We interpreted the results of statistical tests based on visual inspection of the funnel plots. Asymmetry of the funnel plot may indicate publication bias or other biases related to sample size, though it may also represent a true relationship between trial size and effect size.

#### Data synthesis

We described and summarised the findings of included studies to address the two stated objectives of the review. We provide a narrative synthesis describing the interventions, participants, study characteristics and effects of eligible interventions upon pre‐specified outcomes (see Criteria for considering studies for this review).

Our statistical analysis of the results of included studies used a series of random‐effects and fixed‐effect models to estimate summary effect sizes as SMDs with 95% confidence intervals. We determined the final configuration of our statistical analysis based on the final version conceptual model (Figure 1). We conducted the statistical analysis using STATA (StataCorp, College Station, TX, 2014) and it comprised the following stages:

Stage 1. A standard meta‐analysis to estimate summary effect sizes for all eligible interventions versus all comparators, using metan (Harris 2008).

Stage 2. A meta‐regression analysis with type of product (food, alcohol, tobacco) as a covariate.

Stage 3. A meta‐regression analysis with study characteristics as additional covariates.

Stage 4. A meta‐regression analysis with intervention characteristics as covariates. At the protocol stage, we considered the option of conducting multivariate analysis to deal with studies with multiple treatment arms in order for direct comparisons between each treatment arm and a control condition to be modelled, using mvmeta (White 2011). In practice, we did not judge this appropriate and we conducted all meta‐regression analyses using metareg (Harbord 2008).

Stage 5. A meta‐regression analysis with participant characteristics and 'Risk of bias' assessment as covariates.

We only incorporated outcome data from independent comparisons into the statistical analysis. For example, from an included study that measured energy consumed from a lunch meal in four groups of participants served with a 275 g, a 367 g, a 458 g or a 550 g sandwich (Rolls 2004a), available pairwise comparisons are: 275 g versus 367 g, 275 g versus 458 g, 275 g versus 550 g, 367 g versus 458 g, 367 g versus 550 g, and 458 g versus 550 g. However, since these comparisons are not independent from one another, only the incremental comparisons (which are independent) were incorporated: 275 g versus 367 g, 367 g versus 458 g, and 458 g versus 550 g. Our decision to incorporate only outcome data from incremental comparisons into the statistical analysis effectively assumes a linear 'dose‐response' relationship between portion size and consumption/selection for portions of the sizes investigated in included studies. This assumption was judged reasonable by topic expert members of the review team and it is also conservative in terms of its impact on estimates of summary effect sizes. Some groups of study participants feature in two incremental comparisons (e.g. the 367 g group features in both the 275 g versus 367 g comparison and the 367 g versus 458 g comparison), therefore we halved sample sizes for groups featuring in two incremental comparisons to adjust their weighting in the analysis for this non‐independence.

Preliminary examination of outcome data revealed substantive variation in effect sizes between comparisons identified from studies that manipulated portion, package, individual unit or tableware *size* and those identified from studies that manipulated tableware *shape*. We did not judge comparisons of size conceptually comparable to comparisons of shape among the set of studies included in this review: size comparisons consisted in larger versus smaller sizes (of a portion, package, individual unit or item of tableware), whilst shape comparisons consisted in shorter, wider versus taller, narrower glasses or bottles (tableware). We therefore took the post‐hoc decision to conduct separate meta‐analyses for size and shape respectively, for both consumption and selection outcomes. (This decision effectively removed the covariate that differentiated between size and shape manipulations from subsequent meta‐regression analyses ‐ see below and Table 5). Preliminary analyses also revealed substantive variation in effect sizes between those measured in children and those measured in adults (as well as variation in effect sizes between adults of different ages), and between comparisons involving food products and those involving tobacco products. We therefore estimated supplementary summary effect sizes for these subgroups to illustrate these variations in effects. In describing the effects of size and shape interventions on selection and consumption, our narrative synthesis is disaggregated as appropriate to reflect these variations and to incorporate supplementary effect sizes estimated to illustrate them (see Effects of interventions).

We used the following procedures for meta‐regression analyses. First, for each of the two outcomes (consumption and selection), we conducted a series of univariable analyses using random‐effects models to test for a statistical association between each covariate and the study‐level effect size (SMD). All variables identified in the final version of the conceptual model (see Table 5) were candidate covariates for univariable analyses. Blinded to data extracted for covariates from study reports by two researchers (GJH, IS), topic experts within the review team selected six baseline participant characteristics to be prioritised when contacting study authors to request data on potential effect modifiers that appeared to have been measured but were missing from study reports. This selection was based on what were expected to be the most important modifiers of the effects of the intervention, primarily based on topic experts' knowledge of theory and evidence for determinants of between‐person variation in levels of food and energy intake (since the majority of studies included in this review focused on food ‐ see Description of studies). The six selected covariates (variable type) were: age (continuous), gender (categorical), BMI (continuous), dietary restraint (continuous), dietary disinhibition (continuous) and hunger (continuous). All six had been pre‐specified in the original version of the conceptual model (Figure 1) and had been measured at baseline in at least one included study. We decided in advance of conducting univariable meta‐regression analyses that candidate covariates would be excluded if they had been measured in fewer than 10 independent comparisons feeding into an analysis (insufficient data) or if there was no variation in the value of the covariate between independent comparisons feeding into an analysis (absence of variation, which precluded estimation). Based on these exclusion criteria, we conducted two series of univariable meta‐regression analyses to investigate potential modifiers of the effects of larger versus smaller portions, packages, individual units or tableware on: (a) consumption of food and tobacco; and (b) on the selection (without purchase) of food. We did not conduct other planned series of univariable meta‐regression analyses due to insufficient data following application of the exclusion criteria outlined above.

Second, we estimated random‐effects models to identify the collections of study‐level covariates that best explained the between‐studies component of the variance in study‐level estimates of effect size. As with univariable analyses, it proved possible in practice to implement this analysis to investigate potential modifiers of the effects of larger versus smaller portions, packages, individual units or tableware on: (a) consumption of food and tobacco; and (b) on the selection (without purchase) of food. We did not conduct other planned second stage analyses due to insufficient data. We selected variables for inclusion in models using a stepwise forward selection procedure. We selected first the covariate which had the largest value of R^2^ (a measure of the proportion of the between‐studies component of the variance explained by the model) based on the results of the preceding series of univariable analyses. Next, we added each of the other covariates observed to be statistically associated with the study‐level effect size in the results of the preceding univariable analyses to the model in sequence (in an order corresponding to Stages 2 to 4 of the statistical analysis plan, outlined above in this section). Each covariate was retained in the final model if its incorporation contributed to an increase in the value of the R^2^ but was otherwise dropped from the model. Consequently, once this procedure was completed, the final model specification maximised the value of R^2^.

To facilitate interpretation of estimated effect sizes (Schünemann 2011), we re‐expressed a series of SMD values ranging between 0.1 and 2.5 in terms of selected metrics of food or tobacco selection/consumption. Baseline values (SMD = 0.0) reflect estimated average (mean) consumption levels among representative samples of UK adults or children and associated among‐participant variation (that is, the standard deviation). Two researchers (IS and HBL) estimated average (mean) food energy intake, non‐alcoholic beverage consumption and cigarette consumption (among smokers) using unweighted data from the UK National Diet and Nutrition Survey Years 1‐4, collected using 24‐hour dietary recall in a nationally representative UK population sample (National Centre for Social Research 2012). One researcher (IS) also estimated an alternative estimate of average cigarette consumption (among smokers) based on unweighted data from the UK Opinions and Lifestyle Survey 2012 (Office for National Statistics 2012). We used these data to re‐express SMD values in terms of the proportionate (%) and absolute changes from baseline values in terms of each selected metric and tabulated these data for illustrative purposes (see Effects of interventions). We also compared re‐expressed values among UK adults and children to those based on published estimates among equivalent US samples.

##### 'Summary of findings' table

We used the standard GRADE system to rate the quality of the respective bodies of evidence for (1) consumption and (2) selection (with or without purchasing) outcomes in terms of the extent of our confidence in (summary) estimates of effects. GRADE criteria for assessing quality of evidence encompass study limitations, inconsistency, imprecision, indirectness, publication bias and other considerations. We recorded the justifications underpinning these assessments. We present this information in a series of 'Summary of findings' tables developed using GRADEpro GDT (Brozek 2008), alongside a summary of the estimated intervention effect and details of the numbers of studies (independent comparisons) and participants that underpinned each estimate. Our decision to present a series of 'Summary of findings' tables rather than a single table reflects our decisions to conduct separate meta‐analyses for size and shape respectively (for both consumption and selection outcomes) and to present separate summary effect sizes for food products and tobacco products (see above in this section ‐ in both cases preliminary examination of outcome data had identified substantial variation in effect sizes between studies with these variant characteristics). Separate 'Summary of findings' tables are therefore presented to summarise evidence for the (differential) effects of exposure to larger‐sized portions, packages and tableware (by product ‐ food and tobacco) and exposure to differently shaped tableware (by product ‐ food only). Within each 'Summary of findings' table, findings are grouped by outcome (consumption and selection). In addition to presenting the overall summary effect size for each outcome, we also present disaggregated summary effect sizes for subgroups of studies involving children and adults respectively (again, due to identified variation in effect sizes between those measured in children and those measured in adults ‐ see above in this section).

#### Sensitivity analysis

We conducted a sensitivity analysis to explore the impact of outcome data imputed due to missing data. In practice, standard deviations were the only component of outcome data that needed to be imputed for some independent comparisons due to missing data. Therefore, this sensitivity analysis in practice involved re‐estimating fixed‐effect and random‐effects meta‐analyses (for both selection and consumption outcomes – all comparisons) using imputed values for standard deviations that were (1) double and (2) half those used in the 'base case' analyses reported in the Effects of interventions section. At the protocol stage, we had also planned to conduct a sensitivity analysis to explore the separate analysis of studies of food and tobacco products. In practice, we estimated supplementary summary effect sizes for these subgroups of studies (see Data synthesis), which was functionally equivalent to this planned sensitivity analysis.

## Results

### Description of studies

#### Results of the search

The flow of studies through the systematic review process is shown in Figure 2. Electronic database searches retrieved a total of 76,279 study records, including duplicates. Searches of other resources identified 23 additional study records not retrieved by electronic database searches, comprising 15 records identified by searching reference lists of eligible study reports or forward citation tracking and eight records identified within our preceding, broader scoping review (Hollands 2013a). Automatic and manual de‐duplication identified 24,624 duplicate records, which we discarded. Therefore, 51,655 unique records entered title/abstract screening. Of these, we excluded 51,472 records and obtained corresponding full‐text study reports for the remaining 183 records assessed as potentially eligible.

**2:**
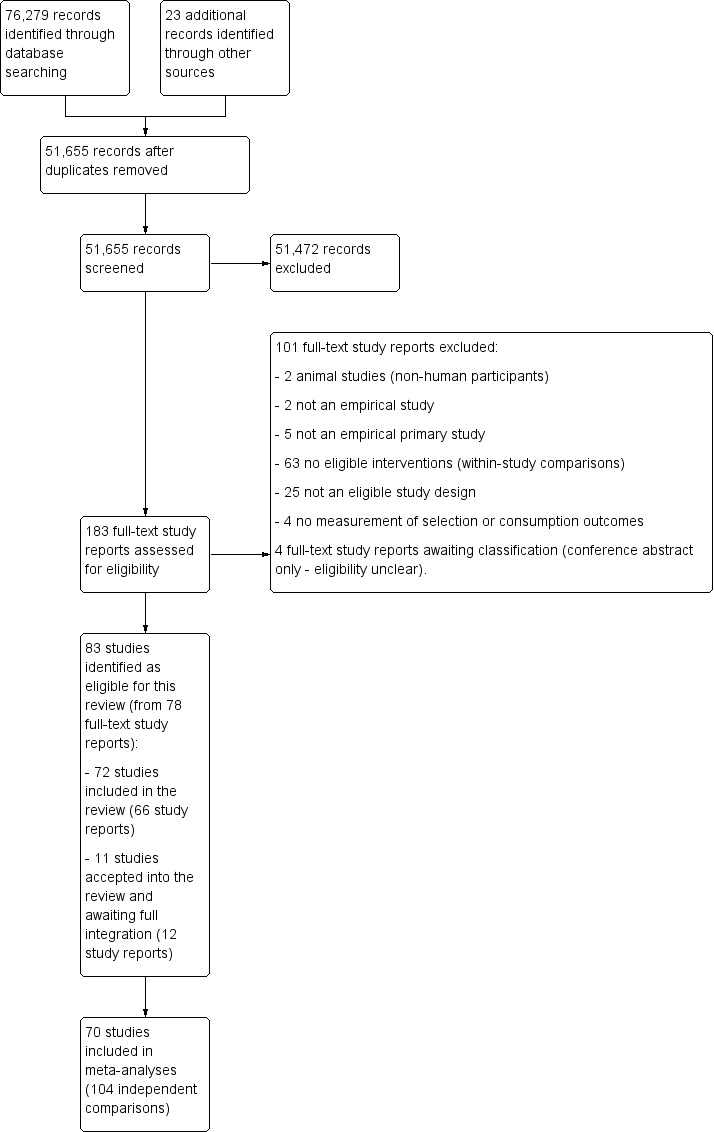
PRISMA study flow diagram.

We excluded 101 study reports based on full‐text screening. Primary reasons for exclusion are summarised in Figure 2 (PRISMA flow diagram) and in the Characteristics of excluded studies table. A further four full‐text study reports were conference abstracts with insufficient information to enable confident assessment of eligibility (Loney 2010, Martinez 2010, Schmidt 2013, Skov 2013). Brief details of these four studies are provided in Characteristics of studies awaiting classification tables. Therefore, following exclusions, identification and linking of multiple eligible study reports of the same study and identification of study reports comprising multiple eligible studies, we have identified a total of 83 studies as meeting the eligibility criteria for this review (from 78 full‐text study reports). The number of included studies exceeds the number of included study reports due to the comparative incidences of study reports that report multiple studies (i.e. two or more studies reported in the same publication) and studies reported in single or multiple study reports among studies/reports that we identified as meeting eligibility criteria for this review.

##### Eligible studies included in the review

Seventy‐two of the 83 eligible studies (66 study reports) were identified by the original search initiated in November 2012 (see Search methods for identification of studies). These 72 studies, published between 1978 and July 2013, are described in the Included studies section below (with further details of each study provided in Characteristics of included studies tables) and are recorded as 'studies included in the review' in Figure 2. All remaining sub‐sections of the Results section of the current version of this review (i.e. Included studies, Excluded studies, Risk of bias in included studies and Effects of interventions), as well as its Discussion and Authors' conclusions sections, are based *exclusively* on evidence collected from these 72 included studies. We sought to establish contact with authors of 36 of 72 included studies to request data missing from study reports (Argo 2012 (S5); Burger 2011; Cavanagh 2013; Coelho do Vale 2008 (S2); DiSantis 2013; Fisher 2013; Flood 2006; Goldstein 2006; Jeffery 2007; Kral 2004a; Kral 2010; Levitsky 2004; Marchiori 2012a; Marchiori 2012c; Mishra 2012 (S1); Mishra 2012 (S2); Rolls 2000; Rolls 2002; Rolls 2004a; Rolls 2004b; Rolls 2006a; Rolls 2007b (S1); Rolls 2007b (S3); Rolls 2010a (E1); Rolls 2010b (E2); Russell 1980; Scott 2008b (S2); Scott 2008c (S3); Scott 2008d (S4); Spill 2010; Spill 2011b; Wansink 1996a (S1); Wansink 2001; Wansink 2003 (S1); Wansink 2003 (S2); Wansink 2011a (S4)). We were able to establish contact with authors of 32 of these 36 studies (Burger 2011; Cavanagh 2013; Coelho do Vale 2008 (S2); DiSantis 2013; Fisher 2013; Flood 2006; Jeffery 2007; Kral 2004a; Kral 2010; Levitsky 2004; Marchiori 2012a; Marchiori 2012c; Rolls 2000; Rolls 2002; Rolls 2004a; Rolls 2004b; Rolls 2006a; Rolls 2007b (S1); Rolls 2007b (S3); Rolls 2010a (E1); Rolls 2010b (E2); Russell 1980; Scott 2008b (S2); Scott 2008c (S3); Scott 2008d (S4); Spill 2010; Spill 2011b; Wansink 1996a (S1); Wansink 2001; Wansink 2003 (S1); Wansink 2003 (S2); Wansink 2011a (S4)), of which 20 supplied the requested information (Burger 2011; Cavanagh 2013; Coelho do Vale 2008 (S2); DiSantis 2013; Flood 2006; Kral 2010; Levitsky 2004; Marchiori 2012a; Marchiori 2012c; Rolls 2000; Rolls 2002; Rolls 2004a; Rolls 2004b; Rolls 2006a; Rolls 2007b (S1); Rolls 2007b (S3); Rolls 2010a (E1); Rolls 2010b (E2); Spill 2010; Spill 2011b). Including data supplied by study authors, 70 of 72 included studies provided useable data for meta‐analyses (104 independent comparisons) ‐ the exceptions were the studies by Argo 2012 (S5) and Goldstein 2006.

##### Eligible studies accepted into the review and awaiting full integration

The other 11 of the 83 eligible studies (12 study reports) were identified by the updated search (30 January 2015) (Bajaj 2014; Haire 2014; Kral 2014; Marchiori 2014; Rolls 2014a; Smith 2013a; van Ittersum 2013; van Kleef 2014; Wansink 2013; Wansink 2014; Williams 2014). These 11 studies, published during 2013 and 2014, are described in Characteristics of studies awaiting classification tables and are recorded as 'studies accepted into the review and awaiting full integration' in Figure 2. As well as describing key characteristics of each of these 11 further eligible studies, the Characteristics of studies awaiting classification tables also include provisional study‐level effect sizes (SMDs and 95% CIs) computed based on useable data provisionally extracted from 12 corresponding study reports.

It was important to establish whether the full integration of these 11 eligible studies could change the interpretation of the results of this review, and hence its conclusions, as reported below in Results, Discussion and Authors' conclusions. We therefore conducted preliminary analyses to investigate this issue using outcome data that could provisionally be extracted from each of the 11 further eligible studies. These preliminary analyses are summarised in Appendix 2. Their results establish that there is minimal potential for full integration of these 11 studies to change the interpretation of the results of this review, and hence its conclusions, as reported below in Results, Discussion and Authors' conclusions. On this basis we took the pragmatic decision (in consultation with the Cochrane Public Health Review Group) to defer full integration of these 11 studies until the first major update of this review. Therefore, as highlighted above, all results and findings presented in the remainder of the main text of this review are based *exclusively* on evidence collected from the 72 included studies identified by the original search up to and including 20 November 2012.

#### Included studies

The majority of the 72 included studies were conducted in the USA (58 of 72), with five studies from Canada (Argo 2012 (S1); Argo 2012 (S2); Argo 2012 (S4); Argo 2012 (S5); Koh 2009), three from Belgium (Marchiori 2011; Marchiori 2012a; Marchiori 2012c), two from the Netherlands (Coelho do Vale 2008 (S2); Hermans 2012), two from the UK (Kelly 2009; Russell 1980), and one study each from Australia (Cavanagh 2013) and South Korea (Ahn 2010). We identified no eligible studies conducted in low‐ or middle‐income countries (LMICs). The majority of included studies were conducted in laboratory settings (50 of 72) and the others (22 of 72) were conducted in field settings ‐ predominantly restaurants or school or workplace cafeterias (Ahn 2010; Diliberti 2004; DiSantis 2013; Ebbeling 2007; Huss 2013; Jeffery 2007; Leahy 2008; Looney 2011; Marchiori 2012c; Mishra 2012 (S1); Raynor 2007; Raynor 2009; Russell 1980; Spill 2010; Spill 2011b; Stroebele 2009; Wansink 2001; Wansink 2003 (S1); Wansink 2003 (S2); Wansink 2005b; Wansink 2006; Wansink 2011b).

Study participants were adults (16 years or more) in 55 of 72 studies (predominantly younger adults aged 19 to 30 years), children in 16 studies (predominantly younger children aged three to six years) (DiSantis 2013; Ebbeling 2007; Fisher 2003; Fisher 2007b; Fisher 2007c; Fisher 2013; Huss 2013; Kral 2010; Leahy 2008; Looney 2011; Marchiori 2012c; Mathias 2012; Rolls 2000; Spill 2010; Spill 2011b; Wansink 2003 (S1)), and both adults and children in one study (Fisher 2007a). In the median study, participants' mean age was 22.2 years (Rolls 2002), ranging between 2.6 years (Fisher 2007c) and 55.2 years (Ahn 2010). Data on the sex of participants was available in 65 of 72 studies. The median study included 55% female participants, ranging from 0% to 100% female (interquartile range (IQR): 49 to 84). Seventy of 72 studies were conducted in low deprivation contexts, whilst the other two were conducted in high deprivation contexts (DiSantis 2013; Fisher 2007a).

In the median studies, participants' mean body mass indexes (BMIs) were 23.5 (Flood 2006; Raynor 2007) and, across all included studies, mean BMI ranged between 17.0 (Kral 2010) and 34.0 (Fisher 2007a). Mean dietary restraint score (Stunkard 1985) in the median studies was 5.8 (Flood 2006, Rolls 2006a), with a range of 4.3 (Raynor 2007) to 9.8 (Burger 2011), while mean dietary disinhibition score (Stunkard 1985) in the median studies was 4.3 (Rolls 2007b (S1); Rolls 2007b (S2)), with a range of 3.5 (Rolls 2002) to 5.3 (Burger 2011; Kral 2004a). Mean baseline hunger score (Stunkard 1985) in the median study was 4.5 (Flood 2006), with a range of 3.6 (Rolls 2007a) to 5.6 (Rolls 2004b). These results suggest that included studies examined effects in participants who were mainly unrestrained eaters (Stunkard 1985).

Sixty‐nine of 72 studies involved manipulations of food products, with the other three focused on tobacco (Jarvik 1978 (E1); Jarvik 1978 (E2); Russell 1980). No eligible studies of alcohol products were identified. The target of manipulation was the portion size in 35 of 72 studies (Burger 2011; Cavanagh 2013; Diliberti 2004; Fisher 2003; Fisher 2007a; Fisher 2007b; Fisher 2007c; Flood 2006; Goldstein 2006; Hermans 2012; Huss 2013; Jeffery 2007; Kelly 2009; Kral 2004a; Kral 2010; Leahy 2008; Levitsky 2004; Looney 2011; Mathias 2012; Rolls 2000; Rolls 2002; Rolls 2004a; Rolls 2004b; Rolls 2006a; Rolls 2006b; Rolls 2007a; Rolls 2010a (E1); Rolls 2010b (E2); Spill 2010; Spill 2011b; van Kleef 2013; Wansink 1996b (S2); Wansink 1996c (S4); Wansink 2001; Wansink 2005b). In 10 studies the target of manipulation was the package size (Argo 2012 (S1); Argo 2012 (S2); Argo 2012 (S4); Argo 2012 (S5); Coelho do Vale 2008 (S2); Ebbeling 2007; Raynor 2009; Stroebele 2009; Wansink 1996a (S1); Wansink 2011a (S4)), in six studies it was the size of individual units of a product (including in the three included tobacco studies, which all manipulated the length of cigarettes) (Devitt 2004; Jarvik 1978 (E1); Jarvik 1978 (E2); Marchiori 2011; Marchiori 2012c; Russell 1980), and in 15 studies it was the size or shape of tableware (Ahn 2010; DiSantis 2013; Koh 2009; Mishra 2012 (S1); Mishra 2012 (S2); Rolls 2007b (S1); Rolls 2007b (S2); Rolls 2007b (S3); Shah 2011; van Kleef 2012; Wansink 2003 (S1); Wansink 2003 (S2); Wansink 2005d; Wansink 2006; Wansink 2011b). One study incorporated separate manipulations of both portion size and tableware size (Fisher 2013), and two studies incorporated separate manipulations of both portion size and package size (Marchiori 2012a; Raynor 2007). Three studies incorporated concurrent manipulations of package size and individual unit size, applied simultaneously and were therefore inherently confounded (Scott 2008b (S2); Scott 2008c (S3); Scott 2008d (S4)).

Sixty‐nine of 72 studies manipulated size, whilst the other three manipulated shape (Wansink 2003 (S1); Wansink 2003 (S2); Wansink 2005d). Among studies that manipulated size, the larger of the two compared portions, packages, individual units or items of tableware was, on average (median) 167% (IQR: 140 to 200) of the size of the smaller version, and the mode was 200%. The larger of the two compared portions, packages, individual units or items of tableware was ≈200% of the size of the smaller version in one‐third of included food studies (independent comparisons) and fell between 120% and 159% in half of the included food studies, indicating a bimodal distribution. Absolute sizes investigated in included food studies also tended to be large compared with reference portion sizes (defined here as the size that is recommended to be consumed, or that is customarily consumed, in a single eating occasion, by one or more schemes for communicating portion size messages to consumers (Lewis 2012)) derived from a published report on typical portion sizes in the UK in 2002 (Food Standards Agency 2002). For example, the pairs of portion, package or individual unit sizes compared within included food studies both exceeded the reference portion size in 81% (34 of 42) of those independent comparisons for which these data were available and applicable (42 of 86), whilst only 5% (2 of 42) compared a (larger) portion that was ≈100% of the reference portion size with a (smaller) portion that was < 100% of the reference portion size (Food Standards Agency 2002). Reference portion sizes could not be coded for approximately half of the pairs of food product sizes compared within included studies (44 of 86) due to them manipulating tableware (for example, DiSantis 2013), or multiple products simultaneously (for example, Kelly 2009), or due to missing data.

Further details on characteristics of interventions and comparators are provided in Characteristics of included studies.

Consumption outcomes only were reported in 59 of 72 included studies (Ahn 2010; Argo 2012 (S1); Argo 2012 (S2); Argo 2012 (S4); Argo 2012 (S5); Burger 2011; Cavanagh 2013; Coelho do Vale 2008 (S2); Devitt 2004; Diliberti 2004; Ebbeling 2007; Fisher 2007a; Fisher 2007b; Fisher 2007c; Flood 2006; Goldstein 2006; Hermans 2012; Huss 2013; Jarvik 1978 (E1); Jarvik 1978 (E2); Jeffery 2007; Kelly 2009; Kral 2004a; Kral 2010; Leahy 2008; Levitsky 2004; Looney 2011; Marchiori 2011; Marchiori 2012a; Marchiori 2012c; Mathias 2012; Mishra 2012 (S1); Mishra 2012 (S2); Raynor 2007; Raynor 2009; Rolls 2000; Rolls 2002; Rolls 2004a; Rolls 2004b; Rolls 2006a; Rolls 2006b; Rolls 2007a; Rolls 2007b (S1); Rolls 2007b (S2); Rolls 2007b (S3); Rolls 2010a (E1); Rolls 2010b (E2); Russell 1980; Scott 2008b (S2); Scott 2008c (S3); Scott 2008d (S4); Shah 2011; Spill 2010; Spill 2011b; Stroebele 2009; van Kleef 2013; Wansink 2001; Wansink 2005b; Wansink 2011b). Selection outcomes only were reported in seven other studies (Wansink 1996a (S1); Wansink 1996b (S2); Wansink 1996c (S4); Wansink 2003 (S1); Wansink 2003 (S2); Wansink 2006; Wansink 2011a (S4)), whilst both selection and consumption outcomes were reported in six other studies (DiSantis 2013; Fisher 2003; Fisher 2013; Koh 2009; van Kleef 2012; Wansink 2005d). Outcomes were measured objectively rather than by participant self report in almost all included studies with two exceptions (Ahn 2010; Jeffery 2007), and were typically measured over a period of one day or less (60 of 72 studies). Those studies that measured outcomes over a period exceeding one day were Ahn 2010, Fisher 2013, Huss 2013, Jeffery 2007, Kelly 2009, Raynor 2007, Raynor 2009, Rolls 2006a, Rolls 2006b, Rolls 2007a, Russell 1980 and Stroebele 2009.

In line with the eligibility criteria, all 72 included studies were randomised controlled trials (see Types of studies). Thirty‐eight had a within‐subjects (cross‐over) design (Burger 2011; Devitt 2004; DiSantis 2013; Ebbeling 2007; Fisher 2003; Fisher 2007a; Fisher 2007b; Fisher 2007c; Fisher 2013; Flood 2006; Huss 2013; Jarvik 1978 (E1); Jarvik 1978 (E2); Jeffery 2007; Kelly 2009; Kral 2004a; Kral 2010; Leahy 2008; Levitsky 2004; Looney 2011; Mathias 2012; Rolls 2000; Rolls 2002; Rolls 2004a; Rolls 2004b; Rolls 2006a; Rolls 2006b; Rolls 2007a; Rolls 2007b (S1); Rolls 2007b (S2); Rolls 2007b (S3); Rolls 2010a (E1); Rolls 2010b (E2); Russell 1980; Shah 2011; Spill 2010; Spill 2011b; Stroebele 2009), and the remaining 34 had a between‐subjects (parallel‐group) design (Ahn 2010; Argo 2012 (S1); Argo 2012 (S2); Argo 2012 (S4); Argo 2012 (S5); Cavanagh 2013; Coelho do Vale 2008 (S2); Diliberti 2004; Goldstein 2006; Hermans 2012; Koh 2009; Marchiori 2011; Marchiori 2012a; Marchiori 2012c; Mishra 2012 (S1); Mishra 2012 (S2); Raynor 2007; Raynor 2009; Scott 2008b (S2); Scott 2008c (S3); Scott 2008d (S4); van Kleef 2012; van Kleef 2013; Wansink 1996a (S1); Wansink 1996b (S2); Wansink 1996c (S4); Wansink 2001; Wansink 2003 (S1); Wansink 2003 (S2); Wansink 2005b; Wansink 2005d; Wansink 2006; Wansink 2011b; Wansink 2011a (S4)). There was no evidence of funding of included studies by agencies that may have commercial interests in their results.

#### Excluded studies

We excluded 81 of 149 study reports identified by the original search from this review at the full‐text screening stage. We further excluded 20 of 34 study reports identified by the updated search at the full‐text screening stage. Details of the combined total of 101 excluded study reports (of 183 screened in full‐text) are provided in Characteristics of excluded studies, along with the primary reason for exclusion in each case (in two cases ‐ Just 2014 and Scisco 2012 ‐ the excluded study report comprised two ineligible studies (denoted as S1 and S2 in Characteristics of excluded studies tables), both excluded).

The most common reasons for exclusion were the lack of an eligible intervention, and the lack of an eligible study design. Illustrative examples of studies with no eligible intervention include Attwood 2012, in which participants were instructed to drink all of the product presented to them, rather than the quantity that they freely chose to drink. Bohnert 2011 examined the effects of using a specially designed plate (which gave visual indications of suggested portion size) versus a plain plate. There was no difference in the size or shape of the different plates, and the only difference was in its surface design, therefore there was no eligible intervention.

Illustrative examples of studies with an ineligible study design include Leidy 2010, in which participants were not randomly assigned between the two portion size conditions. The comparison was between two different experiments, as confirmed by correspondence with the senior author. Freedman 2010 again did not randomly assign participants, but instead appeared to report a study with a case series or uncontrolled longitudinal design.

### Risk of bias in included studies

Following the procedures outlined in Assessment of risk of bias in included studies, we made a summary 'Risk of bias' assessment for each outcome. We classified seven studies from the 65 that measured consumption as at overall high risk of bias with respect to this outcome (Ahn 2010; Diliberti 2004; Goldstein 2006; Huss 2013; Mishra 2012 (S1); Raynor 2009; Wansink 2005d), with the remaining 58 studies classified as at overall unclear risk of bias. We classified nine of the 13 studies that measured selection (without purchase) as at overall unclear risk of bias with respect to this outcome (DiSantis 2013; Fisher 2003; Fisher 2013; Koh 2009; van Kleef 2012; Wansink 2003 (S1); Wansink 2003 (S2); Wansink 2006; Wansink 2011a (S4)), with four at high risk of bias (Wansink 1996a (S1); Wansink 1996b (S2); Wansink 1996c (S4); Wansink 2005d).

Decisions regarding individual domains within the Cochrane 'Risk of bias' tool are summarised below. Figure 3 summarises risk of bias judgements across included studies and full details of review authors' judgements and support for judgements are provided for each study in 'Risk of bias' tables in Characteristics of included studies.

**3:**
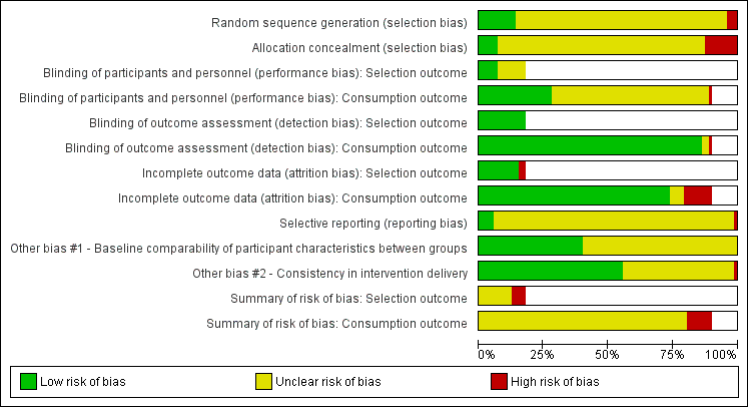
'Risk of bias' graph: review authors' judgements about each risk of bias item presented as percentages across all eligible studies (N = 83. 'Risk of bias' assessments completed for 72 eligible studies included in the review. White spaces in the bars of this graph denote the respective proportions of the 72 included studies that did not measure (i) selection or (ii) consumption outcomes. See also Results of the search and Figure 2).

#### Allocation

We judged the risk of allocation bias due to the procedures used to generate a randomised sequence of assignments to be unclear in 59 of 72 studies because insufficient information was provided about these procedures to permit a judgement of low or high risk. We judged the risk of bias from this source to be low in 10 studies (Ahn 2010; Ebbeling 2007; Looney 2011; Raynor 2009, Russell 1980; Spill 2010; Wansink 1996a (S1); Wansink 1996b (S2); Wansink 1996c (S4); Wansink 2005d) and high in the remaining three studies (Goldstein 2006; Huss 2013; Mishra 2012 (S1)).

We judged risk of bias due to procedures used to conceal the allocation sequence from those involved in the enrolment and assignment of participants to be unclear in 58 studies, again due to insufficient information to permit a judgement of low or high risk. We judged risk of bias from this source to be low in five studies (DiSantis 2013; Ebbeling 2007; Huss 2013; Mathias 2012; Wansink 2011b), and high in the other nine studies (Ahn 2010; Diliberti 2004; Goldstein 2006; Mishra 2012 (S1); Raynor 2009; Wansink 1996a (S1); Wansink 1996b (S2); Wansink 1996c (S4); Wansink 2005d).

#### Blinding

##### Blinding of participants and personnel

Among the 13 studies that reported selection outcomes, we judged risk of bias to be unclear in this domain due to insufficient information in eight studies (DiSantis 2013; Fisher 2003; Fisher 2013; Wansink 2003 (S1); Wansink 2003 (S2); Wansink 2005d; Wansink 2006; Wansink 2011a (S4)), and low in the remaining five studies (Koh 2009; van Kleef 2012; Wansink 1996a (S1); Wansink 1996b (S2); Wansink 1996c (S4)).

Among the 65 studies that reported consumption outcomes, we judged risk of bias to be high in this domain in one study (Ahn 2010), low in 20 studies (Argo 2012 (S1); Argo 2012 (S2); Argo 2012 (S4); Argo 2012 (S5); Cavanagh 2013; Coelho do Vale 2008 (S2); Goldstein 2006; Hermans 2012; Koh 2009; Marchiori 2011; Marchiori 2012a; Marchiori 2012c; Raynor 2007; Raynor 2009; Scott 2008b (S2); Scott 2008c (S3); Scott 2008d (S4); van Kleef 2012; van Kleef 2013; Wansink 2011b), and unclear due to insufficient information in the remaining 44 studies.

##### Blinding of outcome assessment

We judged all 13 studies that reported selection outcomes to be at low risk of bias in this domain (DiSantis 2013; Fisher 2003; Fisher 2013; Koh 2009; van Kleef 2012; Wansink 1996a (S1); Wansink 1996b (S2); Wansink 1996c (S4); Wansink 2003 (S1); Wansink 2003 (S2); Wansink 2005d; Wansink 2006; Wansink 2011a (S4)).

Among the 65 studies that reported consumption outcomes, we judged the risk of bias to be high in this domain in one study (Ahn 2010). In this study, we regarded it possible that the outcome measurement may have been influenced by a lack of blinding, because participants were instructed to keep dietary records of their own intake. We judged two other studies to be at unclear risk of bias due to insufficient information (Jeffery 2007; Stroebele 2009). We judged the remaining 62 studies to be at low risk of bias.

#### Incomplete outcome data

Among the 13 studies that reported selection outcomes, we judged two to be at high risk of bias for this domain (Fisher 2003; Fisher 2013), with the remaining 11 studies judged to be at low risk of bias. Of the 65 studies that reported consumption outcomes, we judged eight to be at high risk of bias (Coelho do Vale 2008 (S2); Fisher 2003; Fisher 2007c; Fisher 2013; Leahy 2008; Looney 2011; Marchiori 2011; Mathias 2012), with four studies assessed as at unclear risk of bias (Mishra 2012 (S1); Mishra 2012 (S2); Rolls 2007a; Russell 1980). We judged the remaining 53 studies as at low risk of bias. We judged studies to be at high risk of bias for this domain if > 10% of participants' data had been excluded from the analysis due to low (or zero) levels of selection or consumption, or due to being outliers.

#### Selective reporting

We judged 67 of 72 studies to be at unclear risk of bias in this domain. This was determined by searching for record(s) containing details of the study protocol in online trial registries (ClinicalTrials.gov and the WHO International Clinical Trials Registry Platform (ICTRP)) and finding no corresponding records. As such, there was insufficient information to permit judgement of 'low risk' or 'high risk'. We assessed this domain to be at low risk of bias in four studies for which records were found and the comparison of the trial registry entries and published studies confirmed no selective outcome reporting (Ebbeling 2007; Fisher 2007b; Looney 2011; Raynor 2009). We classified one study as being at high risk of bias due to a discrepancy between the trial registry entry and the published study regarding the specified primary outcomes (Raynor 2007).

#### Other potential sources of bias

We assessed two additional potential sources of bias that we had pre‐specified as potentially important for this review: baseline comparability of participant characteristics between groups and consistency in intervention delivery.

Regarding baseline comparability of participant characteristics between groups, we judged 29 studies to be at low risk of bias (Ahn 2010; Burger 2011; Cavanagh 2013; Ebbeling 2007; Fisher 2003; Fisher 2007a; Fisher 2007c; Fisher 2013; Hermans 2012; Huss 2013; Jeffery 2007; Kelly 2009; Koh 2009; Kral 2010; Levitsky 2004; Looney 2011; Marchiori 2011; Marchiori 2012a; Marchiori 2012c; Raynor 2007; Raynor 2009; Rolls 2010a (E1); Rolls 2010b (E2); Russell 1980; Stroebele 2009; van Kleef 2012; van Kleef 2013; Wansink 2005b; Wansink 2011b). We assessed studies as being at low risk of bias in this domain if there were no differences in terms of baseline characteristics between comparison groups (study arms in the case of between‐subjects designs and condition orders in the case of within‐subjects designs), or where any observed differences in characteristics had been controlled for in the statistical analysis, or were judged by the review team to be unlikely to impact on key outcomes. We judged risk of bias to be high in this domain in the other 43 studies.

Regarding consistency in intervention delivery, we judged one study to be at high risk of bias because the bowl that was being manipulated was placed in a different location and at a different distance from participants in each comparison group (van Kleef 2012). We judged risk of bias unclear in this domain in 31 studies (Burger 2011; Devitt 2004; DiSantis 2013; Ebbeling 2007; Fisher 2003; Fisher 2007b; Fisher 2007c; Fisher 2013; Hermans 2012; Huss 2013; Koh 2009; Kral 2004a; Kral 2010; Levitsky 2004; Looney 2011; Mathias 2012; Mishra 2012 (S2); Raynor 2009; Rolls 2006a; Rolls 2006b; Rolls 2007a; Rolls 2007b (S1); Rolls 2007b (S2); Rolls 2007b (S3); Scott 2008b (S2); Scott 2008c (S3); Scott 2008d (S4); Shah 2011; Spill 2010; Spill 2011b; Stroebele 2009). We judged the remaining 40 studies to be at low risk of bias in this domain since information and instructions appeared to be standardised between comparison groups.

### Effects of interventions

See: Table 1; Table 2; Table 3; Table 4

This section presents the results of our statistical analyses of outcome data collected from included studies. Results of meta‐analyses are presented as standardised mean differences (SMDs) with 95% confidence intervals (CIs). A rule of thumb for interpreting these effect sizes (SMDs) is as follows: 0.2 represents a small effect, 0.5 a moderate effect and 0.8 a large effect (Cohen 1988; Schünemann 2011).

However, it is perhaps more intuitive to interpret SMDs once they have been re‐expressed using a familiar metric (Schünemann 2011). Figure 4 is intended as an illustrative guide to help readers interpret the estimated effect sizes (SMDs) presented below in this section. Figure 4 re‐expresses a series of SMD values ranging between 0.1 and 2.5 in terms of selected measures of food or tobacco selection/consumption (for example, 'Equivalent change in average daily energy intake from food (kcal) selected or consumed' in the first column). Baseline values (SMD = 0.0) reflect estimated average (mean) consumption levels among representative samples of UK adults or children (see Data synthesis). For example, mean (standard deviation (SD)) daily energy intake from food among UK adults is estimated to be 1727 (± 537) kcal (National Centre for Social Research 2012). Each column of Figure 4 re‐expresses SMD values in terms of proportionate (%) and absolute changes from baseline values (reflecting observed among‐participant variation in consumption‐levels within each corresponding UK sample). For example, a SMD of 0.4 can be re‐expressed as equivalent to a 12.4% (215 kcal) increase in average daily energy intake from food, or a 27.2% (67 g) increase in the average single‐serve quantity of energy‐containing non‐alcoholic beverage, or a three to four cigarette increase in the average daily number of cigarettes, selected or consumed by UK adults.

**4:**
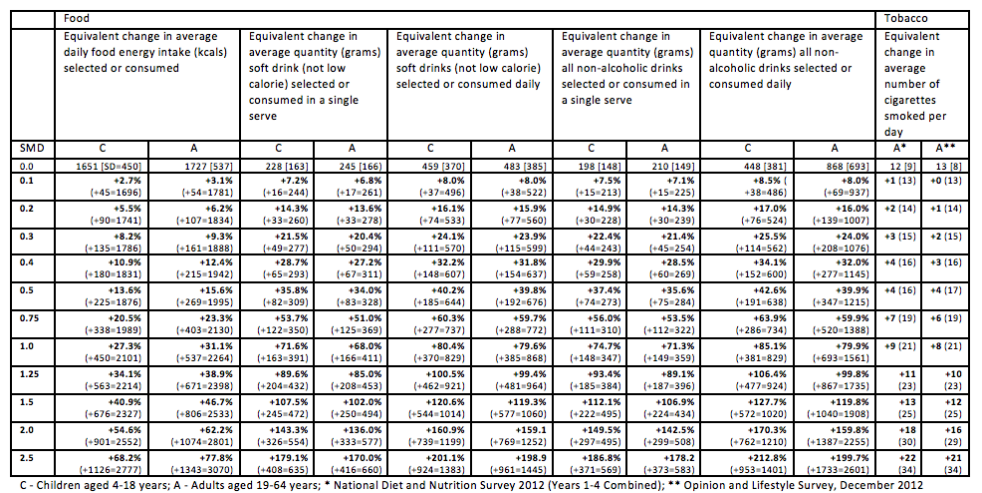
Effect sizes re‐expressed using familiar metrics

It is important to use Figure 4 judiciously. First, end users of this review should consider the extent to which average (mean) baseline values and SDs reflect consumption patterns in their own country or region. For example, at 1727 (± 537) kcal, estimated mean (SD) daily energy intake from food among UK adults is slightly lower than among US adults with a smaller standard deviation (1834 ± 1013 kcal ‐ Drewnowski 2013). As such, if SMDs were re‐expressed based on data for US adults, proportionate (%) and absolute changes from baseline values would be larger than among UK adults (that is, a SMD of 0.4 would be re‐expressed as equivalent to a 22.1% (405 kcal) increase in average daily energy intake from food among US adults). Likewise, at 459 ± 370 g, estimated mean (SD) daily consumption of energy‐containing non‐alcoholic beverages among UK children is lower than daily sugar‐sweetened beverage (SSB) consumption among US children, with a smaller standard deviation (551 ± 1257 g ‐ Wang 2009). As such, if SMDs were re‐expressed based on US children's data, proportionate (%) and absolute changes from baseline values would again be larger than among UK children (that is, a SMD of 0.2 would be re‐expressed as equivalent to a 45.7% (251 g) increase in average daily SSB consumption among US children). Moreover, the inclusion of Figure 4 for illustrative purposes does not restrict the applicability of the results of this review to the UK population, nor is it intended to generalise the results to the UK population.

Second, none of the metrics shown in Figure 4 were actually measured as outcomes in the studies that were incorporated into meta‐analyses presented in this section (and we are not aware of any representative observational studies that include estimates of among‐participant variation in any of the specific measures of consumption/selection that *were* actually used to assess outcomes in these studies). Re‐expressing SMDs estimated using meta‐analyses as equivalent changes in *other* metrics therefore makes an implicit assumption that our estimates of effect size are directly transferable to these other metrics. For example, it assumes that the estimated size of the effect of (larger) size on consumption of food ‐ typically measured in included studies of food products as the quantity of food or energy consumed from a single meal (or single course within a meal) ‐ would produce the same size of effect on a person's energy intake over the course of a whole day. It is therefore important to recognise that, whilst Figure 4 offers illustrations to help guide interpretation of effect sizes estimated using meta‐analyses, it also extrapolates beyond the scope of the outcome data and source studies incorporated into those analyses.

#### 1. Consumption

Ninety‐seven comparisons identified from 64 eligible studies assessed the effect of exposure to different sizes or shapes of portions, packages, individual units or tableware on consumption of food or tobacco by exposed participants.

##### 1.1 Effect of larger size on consumption

We conducted a meta‐analysis to investigate the effect of exposure to larger size on unregulated consumption. Based on characteristics of the studies it incorporated, this meta‐analysis effectively investigated the effect of exposure to larger portions, packages, individual units or tableware on participants' unregulated consumption of food or tobacco. Usable outcome data were available for 92 independent comparisons, involving 6711 participants, identified from 61 eligible food or tobacco studies (Ahn 2010; Argo 2012 (S1); Argo 2012 (S2); Argo 2012 (S4); Burger 2011; Cavanagh 2013; Coelho do Vale 2008 (S2); Devitt 2004; Diliberti 2004; DiSantis 2013; Ebbeling 2007; Fisher 2003; Fisher 2007a; Fisher 2007b; Fisher 2007c; Flood 2006; Hermans 2012; Huss 2013; Jarvik 1978 (E1); Jarvik 1978 (E2); Jeffery 2007; Kelly 2009; Koh 2009; Kral 2004a; Kral 2010; Leahy 2008; Levitsky 2004; Looney 2011; Marchiori 2011; Marchiori 2012a; Marchiori 2012c; Mathias 2012; Mishra 2012 (S1); Mishra 2012 (S2); Raynor 2007; Raynor 2009; Rolls 2000; Rolls 2002; Rolls 2004a; Rolls 2004b; Rolls 2006a; Rolls 2006b; Rolls 2007a; Rolls 2007b (S1); Rolls 2007b (S2); Rolls 2007b (S3); Rolls 2010a (E1); Rolls 2010b (E2); Russell 1980; Scott 2008b (S2); Scott 2008c (S3); Scott 2008d (S4); Shah 2011; Spill 2010; Spill 2011b; Stroebele 2009; van Kleef 2012; van Kleef 2013; Wansink 2001; Wansink 2005b; Wansink 2011b).

Random‐effects meta‐analysis showed a summary mean effect size (SMD) of 0.37 (95% CI 0.29 to 0.45, P value < 0.001), suggesting that exposure to larger‐sized portions, packages, individual units or tableware increased the quantities of food or tobacco people consumed and that the relative effect size was small to moderate (Figure 5). This result was consistent between random‐effects and fixed‐effect models with the fixed‐effect model generating a SMD of 0.40 (95% CI 0.35 to 0.45). The I^2^ statistic shows that 58.4% of the total variance in study‐level estimates of this effect was due to statistical heterogeneity (variation in true effect sizes across studies) rather than sampling error (chance). This represents substantial heterogeneity. A 95% interval for prediction of an effect in a new study similar to the included studies ranges from SMD ‐0.21 to SMD 0.96, reflecting effects ranging from a moderate reduction to a large increase in consumption. An Egger test for funnel plot asymmetry did not identify evidence consistent with the presence of publication bias (P value = 0.20) (Figure 6).

**5:**
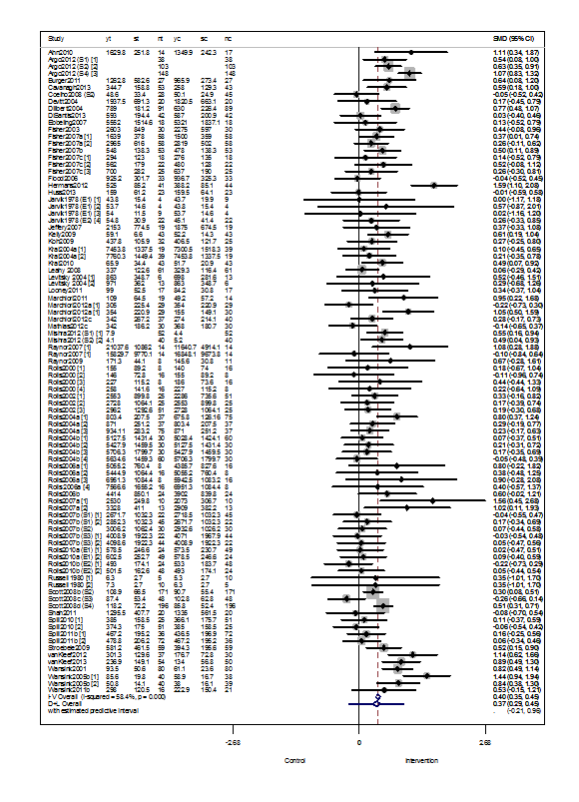
Forest plot of the standardised mean difference in unregulated consumption of food or tobacco between participants exposed to larger (intervention) versus smaller (control) sized portions, packages, individual units and/or tableware

**6:**
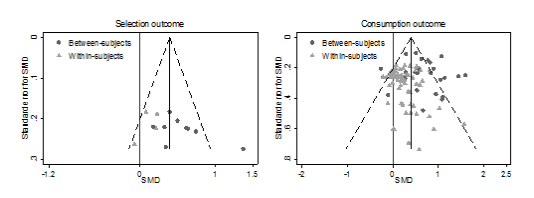
**Assessing publication bias**. Funnel plots including all studies reporting the selection outcome (left) and consumption outcome (right) do not show asymmetry (Egger test P value = 0.20 and P value = 0.18 respectively)

The results of a sensitivity analysis, in which standard deviations imputed for five independent comparisons (five studies: Argo 2012 (S1); Argo 2012 (S2); Argo 2012 (S4); Mishra 2012 (S1); Mishra 2012 (S2)) were (1) doubled and (2) halved (see Sensitivity analysis), indicated that the interpretation of the results of this meta‐analysis is not influenced by changes in the values of imputed standard deviations. Summary mean effect sizes (SMDs) estimated for this sensitivity analysis using random‐effects models were (1) 0.36 (95% CI 0.28 to 0.44, P value < 0.001) and (2) 0.37 (95% CI 0.29 to 0.46, P value < 0.001), respectively. Corresponding summary mean effect sizes (SMDs) from fixed‐effect models were (1) 0.37 (95% CI 0.32 to 0.42) and (2) 0.50 (95% CI 0.45 to 0.54).

###### Potential modifiers of the effect of larger size on consumption

We conducted a series of meta‐regression analyses to investigate the extent to which this substantial heterogeneity could be explained by study‐level covariates. Of 71 candidate study‐level covariates, 40 were excluded due to either insufficient data (< 10 included studies) or were not estimable due to the absence of variability in data values between studies. Univariable meta‐regression analysis results for the 31 remaining study‐level covariates are presented in Appendix 3. We observed six of these covariates to be associated with the effect of larger‐sized portions, packages, individual units or tableware on the quantities of food or tobacco people consume. Below, we report results from each stage of our meta‐regression analyses (as described in the Data synthesis section) and for each stage highlight any variables that we observed to be associated with the intervention effect. We also report on any variables that the review team pre‐specified as potential effect modifiers, but which were not observed in our univariable meta‐regression analyses to be associated with the intervention effect.

####### Type of product (food, alcohol, tobacco)

- Meta‐regression analysis did not find evidence that the effect of larger‐sized portions, packages, individual units or tableware on consumption differed by the type of product studied (i.e. between food and tobacco products ‐ there were no outcome data for alcohol products). However, based on overall low quality evidence from tobacco studies comprising 108 total participants (effective sample size), exposure to longer versus shorter cigarettes was not found to influence the quantity consumed (SMD 0.25, 95% CI ‐0.14 to 0.65) in tobacco studies, while moderate quality evidence for a small to moderate effect of exposure to larger versus smaller‐sized portions, packages or tableware was found among food studies (SMD 0.38, 95% CI 0.29 to 0.46) based on data collected from 6603 total participants (effective sample size).

####### Study characteristics

- Effect sizes were smaller in studies with a within‐subjects design than in those with a between‐subjects design. Specifically, increases in the amount of food or tobacco consumed by participants exposed to larger‐sized portions, packages, individual units or tableware were, on average, 0.40 units smaller (95% CI ‐0.55 to ‐0.25) in studies with a within‐subjects design than in those with a between‐subjects design. Effect sizes for each of these subgroups are presented in Figure 7, showing that exposure to larger sizes increased consumption among participants in both within‐subjects and between‐subjects studies.
- Effect sizes were larger in studies of less healthy food products. Specifically, each 10‐point increase in Food Standards Agency (FSA) nutrient profile score corresponded to a 0.06 unit increase (95% CI 0.04 to 0.22) in the amount of additional food consumed as a result of exposure to larger sizes.
- Effect sizes were larger in studies of more energy‐dense food products. Specifically, each one‐point increase in energy density score (a component of the FSA nutrient profile score) corresponded to a 0.04 unit increase (95% CI 0.00 to 0.08) in the amount of additional food consumed as a result of exposure to larger sizes.
- Effect sizes were larger in studies of food products in which the manipulated food(s) comprised all of those available in the study and all were consumed ad libitum than in the other studies of food products. Specifically, increases in the amount of food consumed as a result of exposure to larger sizes were, on average, 0.22 units larger (95% CI 0.02 to 0.41) in studies of food products in which the manipulated food(s) comprised all of those in the study and all were consumed ad libitum than in studies of food products that did not have these characteristics.
- Effect sizes were larger in studies of food products in which outcome data mapped directly onto the manipulated food(s), as opposed to a wider set of foods including, but not limited to, the manipulated food(s). Specifically, increases in the amount of food consumed as a result of exposure to larger sizes were, on average, 0.32 units larger (95% CI 0.16 to 0.48) in studies of food products in which outcome data mapped directly onto the manipulated food(s) than in studies of food products in which outcome data mapped to a wider set of foods including, but not limited to, the manipulated food(s).
- Meta‐regression analysis did not find evidence that the size of the effect of larger size on consumption was associated with the target of the manipulation (i.e. whether this was a portion, package, individual unit or tableware). Effect sizes for each of these subgroups are presented in Figure 7. While no evidence was found for an effect of exposure to larger‐sized packages and individual units on consumption within the 'package with individual unit' subgroup, this analysis was likely underpowered. We found evidence for this effect in all other subgroups (see Figure 7).

**7:**
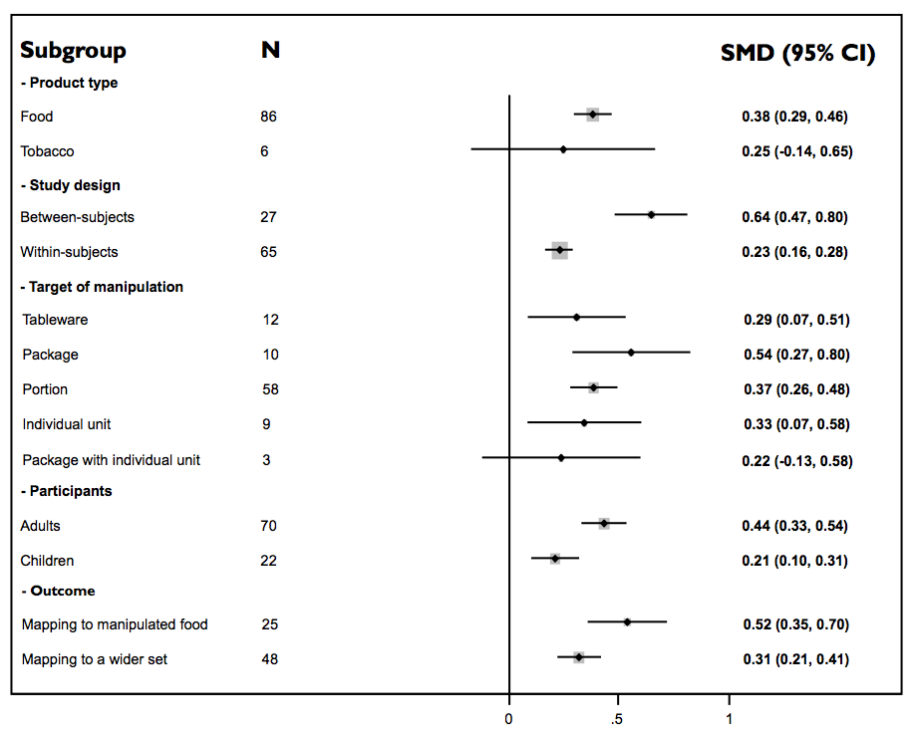
Summary effect sizes (standardised mean differences) in subgroups of studies (consumption outcome)

####### Intervention characteristics

- In meta‐regression analysis, we observed neither the absolute nor the relative difference in size between the two portions, packages, individual units or items of tableware being compared to be associated with the effect of larger size on consumption. This pre‐planned analysis explored the relationship between relative difference in size and the effect of larger size on consumption using a linear regression that (as can be inferred from the null result) showed no convincing evidence of a linear relationship. On visual examination of the relationship, however, a pattern was apparent, with a bimodal distribution of the variable that captures the relative difference in size (that is, the variable that expresses the larger size as a proportion of the smaller size within each independent pairwise comparison ‐ see also Included studies). We therefore undertook a post‐hoc analysis in order to characterise this relationship among studies of food products (that is, limited to independent pairwise comparisons between food portion, package, individual unit or tableware sizes). Specifically, we conducted a meta‐analysis to investigate the effect of larger size on consumption among two subgroups of studies (independent comparisons) clustered around each mode of the identified bimodal distribution (see also Included studies): (1) those in which the larger‐sized portion, package, individual unit of food or item of tableware was in the range between 120% and 160% of the smaller size; and (2) those in which the larger‐sized portion, package or individual unit of food was ≈200% of the smaller size. This analysis therefore excluded outliers (that is, excluding nine independent comparisons in which the larger‐sized portion, package, individual unit of food or item of tableware was > 202% of the smaller size, from Coelho do Vale 2008 (S2), Devitt 2004, Marchiori 2012a, Raynor 2007, Raynor 2009, Shah 2011, van Kleef 2013 and Wansink 2011b ‐ range 243% to 2607%). Summary effect sizes (SMDs), estimated using random‐effects models for each subgroup, were: (1) 0.25 (95% CI 0.15 to 0.35), I^2^ = 22% (based on 39 independent comparisons, 2415 participants); and (2) 0.50 (95% CI 0.31 to 0.69), I^2^ = 66% (based on 25 independent comparisons, 1414 participants).

####### Participant characteristics

- Effect sizes were larger in studies comprising older participants. Specifically, each 10‐year increase in the mean age of participants corresponded, on average, to a 0.09 unit increase (95% CI 0.00 to 0.18) in the incremental amount of food or tobacco consumed as a result of exposure to larger sizes. This result is set in the context of overall moderate quality evidence that the effect of exposure to larger size on consumption of food was present among both children (SMD 0.21, 95% CI 0.10 to 0.31 ‐ moderate quality evidence ‐ 1421 participants) and adults (SMD 0.46, 95% CI 0.40 to 0.52 ‐ moderate quality evidence ‐ 5182 participants) ‐ see Figure 7 and Table 1. We also identified variation in this effect size between studies comprising adult participants of different ages.
- We did not observe the following participant characteristics to be associated with the effect of larger size on consumption: gender, BMI, hunger, dietary restraint and dietary disinhibition.

####### Final regression model

A meta‐regression model was estimated to identify the collection of study‐level covariates that best explained the between‐studies component of the total variance in estimates of the effect of larger sizes on consumption. The final random‐effects model explained 91% of the between‐studies variance in effect sizes for the consumption outcome (R^2^ = 90.77%, P value = 0.001), leaving 9% unexplained. This model incorporated the following five covariates, each of which had been identified as a potential modifier of the effect of larger sizes on consumption based on observed associations in univariable meta‐regression analyses: study design (within‐subjects or between‐subjects); FSA 'nutrient profile score'; FSA 'energy density score'; participants' mean age; and a variable differentiating studies of food products in which the manipulated food(s) comprised all of those available in the study and all were consumed ad libitum from other food studies. The variable differentiating food studies, in which outcome data mapped directly onto the manipulated food(s) as opposed to a wider set of foods, was excluded from the final model for two reasons: first, its addition did not increase the adjusted R^2^ and second, due to its collinearity with the study design covariate (within‐subjects or between‐subjects). Not all of the five incorporated covariates were independently predictive of effect size (consumption) in the final model. Figure 8 comprises three bubble plots that show associations between study‐level effect sizes (effect of larger size on consumption) and each of the three continuous variables identified as potential effect modifiers: FSA 'nutrient profile score'; FSA 'energy density score'; and participants' mean age.

**8:**
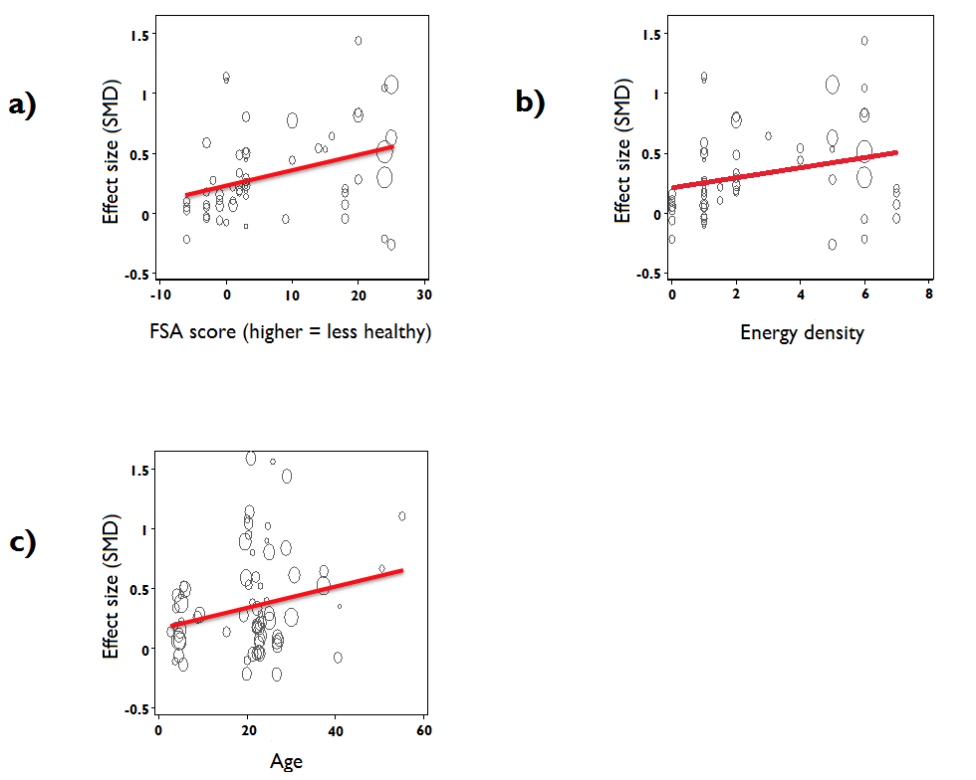
**Bubble plots**. Fitted meta‐regression lines showing associations between study‐level effect sizes for consumption and study characteristics (continuous variables) identified as effect modifiers: a) FSA score; b) energy density; c) age.

##### 1.2. Effect of shape on consumption

One food study involving 50 adult participants investigated the effect of shape on unregulated consumption (Wansink 2005d). This study investigated the effect of being provided with shorter, wider (versus taller, narrower) empty clear plastic bottles on the quantities of water selected and consumed one hour after vigorous physical activity in a sample of US Army and Marine Reserve Officer's Training Corps students. It reported an effect size (SMD) of 1.17 (95% CI 0.57 to 1.78), assessed as very low quality evidence for a large effect of shorter, wider bottles on quantities of water consumed, given that participants provided with shorter, wider bottles had more water available for consumption than those provided with taller, narrower bottles due to having selected (poured) more in the first place (see *Potential modifiers of the effect of shape on selection without purchase*, below).

###### Potential modifiers of the effect of shape on consumption

Investigation of potential modifiers of the effect of shape on consumption was not possible as only one study (comprising one comparison) investigated this effect (Wansink 2005d).

#### 2. Selection

Seventeen comparisons identified from 14 eligible studies assessed the effect of exposure to different sizes or shapes of portions, packages or tableware on quantities of food selected for consumption by exposed participants. No studies investigated this effect in relation to alcohol or tobacco products. None of the 17 comparisons involved purchasing of the food selected for consumption (that is, all measured unregulated selection without purchase).

##### 2.1. Effect of larger size on selection without purchase

We conducted a meta‐analysis to investigate the effect of exposure to larger size on unregulated selection without purchase. Based on characteristics of the studies it incorporated, this meta‐analysis effectively investigated the effect of exposure to larger‐sized portions or tableware on participants' unregulated selection without purchase of food. Usable outcome data were available for 13 comparisons, involving 1164 participants, identified from 10 eligible food studies that we assessed as being at unclear or high risk of bias (DiSantis 2013; Fisher 2003; Fisher 2013; Koh 2009; van Kleef 2012; Wansink 1996a (S1); Wansink 1996b (S2); Wansink 1996c (S4); Wansink 2006; Wansink 2011a (S4).

Random effects meta‐analysis showed a mean summary effect size (SMD) of 0.42 (95% CI 0.24 to 0.59, P value = 0.011), providing overall moderate quality evidence that exposure to larger‐sized portions, packages, individual units or tableware increased the quantities of food people selected for consumption and that the relative effect size was on average small to moderate (Figure 9). This result was consistent between random‐effects and fixed‐effect models, with the fixed‐effect model generating a SMD of 0.40 (95% CI 0.28 to 0.52). The I^2^ statistic indicated that 53.5% of the total variance in study‐level estimates of this effect was due to statistical heterogeneity (substantial heterogeneity). A 95% interval for prediction of an effect in a new study similar to the included studies ranges from SMD ‐0.14 to SMD 0.97, reflecting effects ranging from a small reduction to a large increase in quantity of food selected. An Egger test for funnel plot asymmetry did not identify evidence consistent with the presence of publication bias (P value = 0.18) (Figure 6).

**9:**
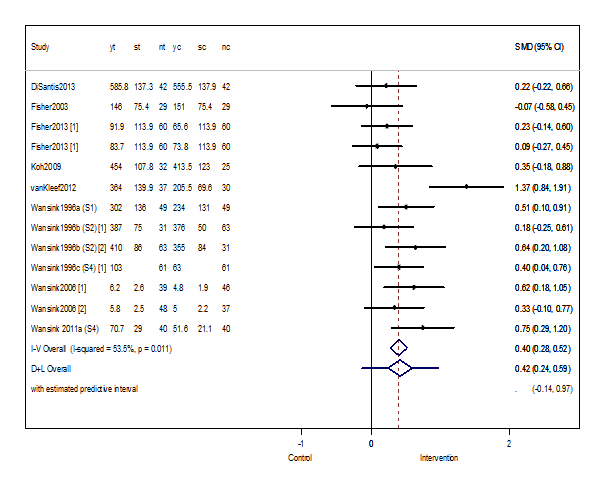
Forest plot of the standardised mean difference in unregulated selection (without purchase) of food between participants exposed to larger (intervention) versus smaller (control) sized portions, packages and/or tableware

The results of a sensitivity analysis, in which standard deviations imputed for one independent comparison (one study: Wansink 1996c (S4)) were (1) doubled and (2) halved (see Sensitivity analysis), indicated that the interpretation of the results of this meta‐analysis is robust to changes in the value of the imputed standard deviation. Summary mean effect sizes (SMDs) estimated for this sensitivity analysis using random‐effects models were (1) 0.42 (95% CI 0.23 to 0.60, P value < 0.001) and (2) 0.41 (95% CI 0.25 to 0.58, P value < 0.001) respectively. Corresponding summary mean effect sizes (SMDs) from fixed‐effect models were (1) 0.42 (95% CI 0.28 to 0.52) and (2) 0.40 (95% 0.30 to 0.50).

###### Potential modifiers of the effect of larger size on selection without purchase

We conducted a series of meta‐regression analyses to investigate the extent to which this substantial heterogeneity in effect sizes could be explained by study‐level covariates. These analyses were limited by low statistical power. Most of the 71 candidate study‐level covariates were excluded due to either insufficient data (< 10 included studies) or were not estimable due to the absence of variability in data values between studies. A full set of results of these univariable meta‐regression analyses is provided in Appendix 4. Of 15 study‐level covariates investigated in these analyses, we observed two to be associated with the effect of larger‐sized portions, packages and/or tableware on the quantities of food participants selected for consumption. Below, we report results from each stage of our meta‐regression analyses (as described in the Data synthesis section) and for each stage highlight any variables that we observed to be associated with the intervention effect. We also report on any variables that the review team pre‐specified as potential effect modifiers, but which were not observed in our univariable meta‐regression analyses to be associated with the intervention effect.

####### Type of product (food, alcohol, tobacco)

- This was excluded due to absence of variation in product type between included comparisons: all comparisons related to food products.

####### Study characteristics

- Effect sizes were smaller in studies with a within‐subjects design than in those with a between‐subjects design. Specifically, increases in the quantities of food selected as a result of exposure to larger‐sized portions or tableware were, on average, ‐0.41 units smaller (95% CI ‐0.76 to ‐0.06) among studies with a within‐subjects design than among those with a between‐subjects design. Effect sizes for each of these subgroups presented in Figure 10 further indicate that exposure to larger sizes was observed to be associated with increased selection of food among participants in between‐subjects studies but not among participants in within‐subjects studies.
- Effect sizes were larger in studies of food products in which outcome data mapped directly onto the manipulated food(s), as opposed to a wider set of foods including (but not limited to) the manipulated food(s). Specifically, increases in the quantities of food selected as a result of exposure to larger sizes were, on average, 0.41 units larger (95% CI 0.06 to 0.76) in the former subgroup than in the latter.
- Meta‐regression analysis did not find evidence that the size of the effect of larger size on selection of food was associated with the target of the manipulation (i.e. whether this was a portion or an item of tableware). Effect sizes for each of these subgroups are presented in Figure 10, which shows that evidence for this effect was found in both studies manipulating portion size (SMD 0.30, 95% CI 0.09 to 0.50) and those manipulating tableware size (SMD 0.51, 95% CI 0.21 to 0.81).

**10:**
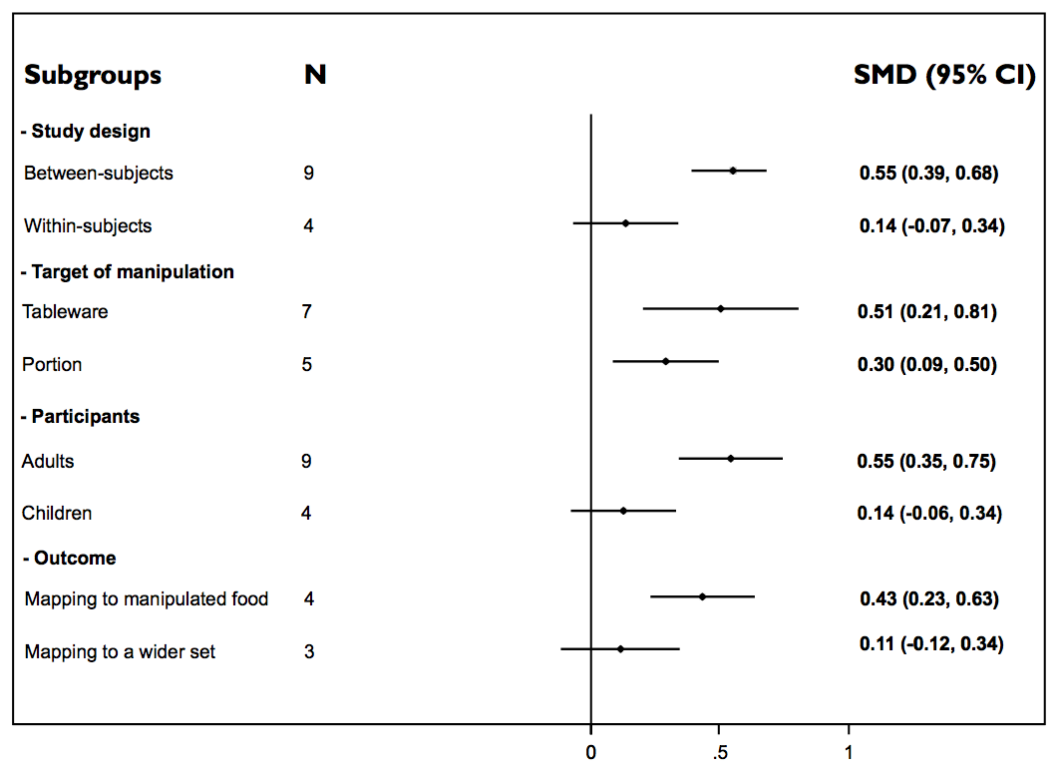
Summary effect sizes (standardised mean differences) in subgroups of studies (selection outcome)

####### Intervention characteristics

- In meta‐regression analysis, we did not observe the relative difference in size between the two portions or items of tableware being compared to be associated with the effect of larger size on selection without purchase. The potential association between this effect and absolute difference in size could not be investigated due to insufficient data.

####### Participant characteristics

- Potential associations between the effect of larger size on selection and the following participant characteristics could not be investigated using meta‐regression analysis due to insufficient data: age, BMI, hunger, dietary restraint and dietary disinhibition. We observed no association between this effect and participants' gender. The results of an illustrative analysis presented in Figure 10 indicate that the effect of exposure to larger size on selection of food was present among adults (SMD 0.55, 95% CI 0.35 to 0.75 ‐ moderate quality evidence ‐ 782 participants) but not among children (SMD 0.14, 95% CI ‐0.06 to 0.34 ‐ low quality evidence ‐ 382 participants) ‐ see also Table 1.

####### Final regression model

Variation in study design (within‐subjects versus between‐subjects) alone explained 79% of the statistical heterogeneity observed in the effect of (larger) size on selection of food (R^2^ = 79.46%), leaving 21% unexplained. The covariate of outcome data mapping directly onto the manipulated food(s) also explained 79% of this statistical heterogeneity (R^2^ = 78.77%), leaving 21% unexplained. A meta‐regression model containing both of these covariates identified as potential effect modifiers could not be estimated due to perfect collinearity. As such the independent effect modifying influences of these two covariates cannot be disentangled. There are at least two plausible complementary explanations for the result that variation in study design explained a large proportion of this statistical heterogeneity. First, all those studies included in the meta‐analysis of the effect of larger size on selection that had a within‐subjects design measured this effect in children, whilst all those with a between‐subjects design measured it in adults. As highlighted above, the results presented in Figure 10 provide an indication that the effect of exposure to larger‐sized portions or items of tableware on quantities of food selected was found in studies of adults but not in studies of children. Second, all source studies included in this meta‐analysis that had a within‐subjects design were conducted by teams from one research centre, as (largely) were source studies that had a between‐subjects design.

##### 2.2. Effect of shape on selection without purchase

We conducted a meta‐analysis to investigate the effect of shape on unregulated selection. Given the characteristics of studies included in this meta‐analysis, it effectively investigated the effect of being provided with shorter, wider empty glasses or plastic bottles on participants' unregulated selection (without purchase) of fruit juices or water in a single, self serve setting. Usable outcome data for this meta‐analysis were available for three comparisons, involving 232 participants, identified from three eligible food studies assessed as being at unclear or high risk of bias (Wansink 2003 (S1); Wansink 2003 (S2); Wansink 2005d).

Random‐effects meta‐analysis showed a mean summary effect size (SMD) of 1.47 with wide confidence intervals (95% CI 0.52 to 2.43). This result provides overall low quality evidence that exposure to shorter, wider glasses or plastic bottles increased the quantities of fruit juices or water people selected for consumption and that the relative size of this effect was very large (Figure 11). This result was consistent between random‐effects and fixed‐effect models with the fixed‐effect model generating a SMD of 1.39 (95% CI 1.10 to 1.69). Although 95% confidence intervals were wide, the lower bound of 0.52 based on the random‐effects model still represents a moderate effect size. The I^2^ statistic from the random‐effects model shows that 90.1% of the total variance in study‐level estimates of this effect was due to statistical heterogeneity (considerable heterogeneity).

**11:**
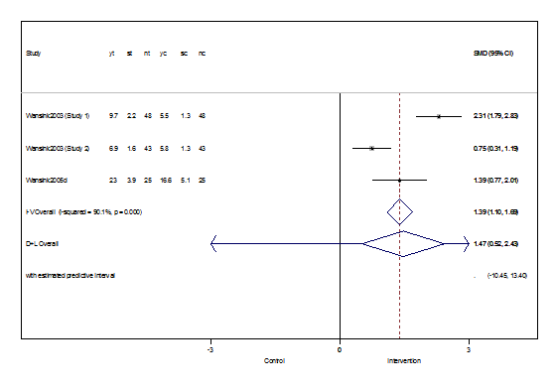
Forest plot of the standardised mean difference in unregulated selection without purchase of fruit juices or water between participants exposed to shorter, wider (intervention) versus taller, narrower (control) empty glasses or plastic bottles

###### Potential modifiers of the effect of shape on selection without purchase

We conducted no meta‐regression analyses to investigate the extent to which this statistical heterogeneity could be explained by study‐level covariates, due to insufficient data. However, it is likely that the considerable between‐studies variance in estimates of this effect may be attributable to the influence of variations between the three source studies providing data incorporated into this meta‐analysis in terms of their participants, interventions, comparisons and settings. Although Wansink 2003 (S1) and Wansink 2003 (S2) both investigated the effect of being provided with shorter, wider (versus taller, narrower) empty glasses on quantities of fruit juices selected by participants from a cafeteria line for consumption at breakfast, the former investigated this effect in a sample of adolescents (aged 12 to 17 years) attending a six‐week health and fitness camp who were motivated as a group to lose weight as well as trained to monitor how much they consumed, whilst the latter investigated the effect in a convenience sample of adults attending a weekend camp on jazz improvisation. The third source study, Wansink 2005d, investigated the effect of being provided with shorter, wider (versus taller, narrower) empty clear plastic bottles on the quantities of water selected for consumption one hour after vigorous physical activity in a sample of US Army and Marine Reserve Officer's Training Corps students. The study conducted in children, Wansink 2003 (S1), comprised 96 participants and found a SMD of 2.31 (95% CI 1.79 to 2.83 ‐ low quality evidence), whilst the estimated summary effect size in the subgroup of two studies conducted in adults, Wansink 2003 (S2) and Wansink 2005d, comprising 136 participants, was SMD 1.03 (95% CI 0.41 to 1.65 ‐ low quality evidence).

## Discussion

### Summary of main results

#### Main effects of size and shape on consumption and selection

##### Size

A clear finding of this review is that people exposed to larger‐sized portions, packages, individual units or tableware consistently consumed larger quantities of food compared with those exposed to smaller sizes. We rated the overall quality of evidence for a small to moderate effect of portion, package, individual unit or tableware size on food consumption among both children and adults as moderate. This quality rating confers confidence that the true effect is likely to be close to the estimated effect size (that is, small to moderate), but leaves open the possibility that it may be substantially different.

If sustained across the whole diet, the summary effect size attributable to these differences in product size would be equivalent to an absolute change in average daily energy intake from food (that is, energy intake from food and non‐alcoholic beverages, but excluding energy intake from alcoholic beverages and dietary supplements) of 215 to 279 kcal among UK adults (a 12% to 16% change from a baseline of 1727 kcal per day ‐ see Figure 4) (National Centre for Social Research 2012). Sustained reductions in daily energy intake from food of this size would have the potential to make meaningful contributions to the prevention and treatment of major risk factors for non‐communicable diseases. For example, 10‐year weight gain between 1999 and 2009 among adults in England (that is, 9 kg at the 90th percentile) has been estimated to be equivalent to extra energy intake of around 24 kcal per day over the same period (Department of Health 2011). Any sustained reductions in daily energy intake exceeding this level are therefore likely to be effective in helping to prevent further weight gain in the population (Department of Health 2011). In relation to the treatment of obesity, the UK National Institute for Health and Care Excellence recommends that adults should lose no more than 0.5 to 1 kg (1 to 2 lb) a week (NICE 2014). This rate of weight loss equates to an energy deficit of 500 to 1000 kcal per day. Although this target energy deficit is some way beyond the effect sizes that could feasibly be achieved by interventions to reduce portion size alone (based on our summary estimate of this effect among studies included in the review), our result suggests that interventions of this kind could meaningfully contribute to helping patients achieve such a target *if their effects were sustained*. Whilst these illustrations highlight the promise of interventions to reduce exposure to larger portion sizes, it is important to highlight that the sustainability of effects remains to be established, since studies included in this review were limited to the investigation of one‐off or repeated exposures over short time periods (see also Implications for practice and Implications for research). Moreover, very few studies included in this review investigated effects among samples of participants motivated to lose weight, further limiting inferences that can be drawn with respect to obesity treatment.

We also found overall moderate quality evidence for a small to moderate effect of portion or tableware size on food *selection* among adults. Adults consistently selected larger quantities of food for consumption when exposed to larger sizes (compared with exposure to smaller sizes). This result is consistent with the role of food selection as an important intermediate endpoint in pathways to consumption. If we assumed that all food selected for consumption were consumed and that this effect size were sustained over time (noting again that we found no evidence for sustainability of effects), it would be equivalent to an absolute change in average daily energy intake from food of 188 to 403 kcal among UK adults (an 11% to 23% change from a baseline of 1727 kcal per day ‐ see Figure 4) (National Centre for Social Research 2012). Whilst we did not find an effect of portion or tableware size on food selection among children, this result was based on overall low quality evidence from a small number of studies (independent comparisons), which confers limited confidence in our estimate of this effect (that is, the true effect among children may be substantially different from our estimate).

We did not find evidence for an effect of individual unit size on consumption of tobacco, based on a meta‐analysis of data collected from studies that investigated exposure to longer versus shorter cigarettes among adult smokers. However, this finding was again based on overall low quality evidence from a small number of older studies. We did not identify any eligible studies that investigated the effects of exposure to differently sized cigarette packs (for example, packs of 20 cigarettes versus packs of 10 cigarettes). Nor did we identify any eligible studies that investigated the effects of exposure to differently sized alcoholic beverage products (or tableware, such as glasses, used to consume such products).

##### Shape

This review found overall very low quality evidence from a single included study for a large effect of exposure to shorter, wider (versus taller, narrower) plastic bottles on the quantities of water participants consumed in a single‐serve context (Wansink 2005d). In this study, participants provided with shorter, wider bottles had more water available for consumption in the first place (due to having already selected more by pouring more into their bottles from a 10 gallon container) than participants provided with taller, narrower bottles. The 'very low quality' rating means that we have little confidence in the estimate of this effect (that is, the true effect is likely to be substantially different from our estimate).

We also found overall low quality evidence for a large to very large effect of exposure to shorter, wider (versus taller, narrower) glasses or plastic bottles on the quantities of fruit juice or water participants *selected* for consumption in a single‐serve context. If the effect size we estimated were transferable to energy‐containing non‐alcoholic beverages (Figure 4), it would be equivalent to an absolute change of 292 to 462 grams in the average quantity of these beverages selected in a single‐serve context among UK children (a 128% to 203% change from a baseline of 228 grams per serve) or 68 to 274 grams among UK adults (a 28% to 112% change from a baseline of 245 grams per serve) respectively (National Centre for Social Research 2012). We rated the quality of evidence as low with respect to our estimates of this effect, which again confers limited confidence in their accuracy. The findings are, however, consistent with long‐established psychological theory and evidence concerning the perceptual biases associated with exposure to differently shaped receptacles (Piaget 1969). While it seems unlikely that interventions that successfully reduced exposure to shorter, wider drinking receptacles (or conversely, increased exposure to taller, narrower versions) could in practice achieve sustained reductions in self served quantities of energy‐containing non‐alcoholic beverages (or increases in self served quantities of healthier alternatives) of this magnitude, this awaits study.

##### Moderators of main effects

As reflected in the discussion of main effects, our results indicated that the effects of portion, package, individual unit or tableware size may be modified by the age of those exposed to such manipulations. Whilst there was evidence that children and young people exposed to larger sizes still consumed more food, the size of this effect was found to be larger among adults, also increasing (albeit by very small incremental amounts) with the age of those exposed. These results suggest that intervening to reduce exposure to larger sizes of portions, packages, individual units or tableware may be more effective in influencing food consumption among adults than among children. This finding appears consistent with suggestions in the literature that as people age, external cues to consumption play an increasingly important role in the regulation of energy intake relative to internal cues, such as hunger and satiety (Ello‐Martin 2005)*.* This phenomenon has been observed in children, but we are not aware of any current evidence for whether this process continues over the adult life course.

It is noteworthy that, with the exception of age, no evidence was found in this review to support claims that the effects of exposure to different portion, package, individual unit or tableware sizes vary between men and women, between individuals with a different body mass index, or between those with different baseline levels of dietary restraint, dietary disinhibition or hunger (that is, those participant characteristics identified in advance as most likely to modify effects). With respect to gender and body mass index, we note that these findings differ from those suggested by the results of another recent review of food portion size effects (Zlatevska 2014). In relation to gender and amounts consumed, Zlatevska and colleagues found that female participants responded less to a doubling of portion size than did male participants (Zlatevska 2014). In relation to body mass index and amounts consumed, they found that overweight participants responded *less* to a doubling of portion size than did non‐overweight participants (Zlatevska 2014) ‐ a result which the authors highlight was unexpected since it challenges previous research suggesting that overweight people may be less sensitive to satiation and more sensitive to external cues than those who are not overweight (Wansink 2007b).

We were unable to examine effect moderation by study participants' socioeconomic status in this review due to the infrequency of reporting of such measures across included studies (this was one component of analysis intended to inform assessment of social differentiation in effects relevant to health equity ‐ seeObjectives and further, related discussion in Overall completeness and applicability of evidence). Socioeconomic status therefore remains an important potential moderator of the effects of sizing interventions that deserves closer attention in future research (see Implications for research).

We did, however, find evidence that this effect of size on consumption may be moderated by the type of food, specifically characterised by the healthiness and energy density of the manipulated food(s), with larger effects found in studies that manipulated less healthy products and in those that manipulated more energy‐dense products (albeit by very small incremental amounts) (see Implications for practice for further discussion of these tentative findings).

We found little evidence consistent with the proposal that the observed effects of size on consumption or selection may differ depending on whether it is the size of a portion, package, individual unit or item of tableware size that is altered. This finding indicates that interventions that successfully reduce exposure to larger sizes can be effective across a range of targets for manipulation.

However, we did identify some evidence to indicate that between‐study variation in the effect of larger size on food consumption may be attributable in part to between‐study differences in the relative size of the two portions, packages, individual units or items of tableware being compared. Although this finding is based on the results of a post‐hoc subgroup analysis (see Effects of interventions), we note that the results are consistent with our prior assumptions that the dose‐response relationship between portion size and consumption or selection would be linear at many of the sizes investigated (see Data synthesis), but that at extremes a non‐linear relationship*c*ould be expected due to a ceiling effect: external cues, such as social norms or perceptual biases that indicate a given amount of a product is appropriate, will eventually give way to internal cues to stop consuming, such as satiety. A recent analysis that plotted the absolute portion size served to each group of participants among included studies against the average (mean) amount of food they consumed from that portion also found a relationship of this kind (Zlatevska 2014). We reiterate (as stated in Included studies) that absolute sizes investigated in included food studies tended to be large compared with reference portion sizes, derived from a published report on typical portion sizes in the UK in 2002 (Food Standards Agency 2002). Knowledge of how the sizes of portions, packages and tableware investigated among included studies compare with reference portion sizes for those foods in different settings was not fully elucidated by this review due to the limited scope and availability of data (from included studies and external sources) to fully address it. However, this remains a critical issue for determining the policy implications of our findings concerning the effects of larger size on selection and consumption (see further commentary on this issue in Overall completeness and applicability of evidence and Implications for practice).

Meta‐regression analyses identified two further variables as potential moderators of the main effects of size on both consumption and selection, both methodological variables. The first variable delimits studies with a within‐subjects design and those with a between‐subjects design (effect sizes were larger in between‐subjects studies). We cannot fully explain this result. It may be an artefact of the different methods used to measure effects in between‐subjects and within‐subjects designs respectively: there are two independent groups in the former but only one group (with repeated measures for each participant) in the latter. Alternatively, the result may be due to factors related to the choice of design, including other methods and procedures applied by research centres using different study designs. The second variable distinguishes studies of food products in which the manipulated food(s) comprised all of those available in the study from all other studies (effect sizes were larger in the former studies). Providing additional foods for study participants to consume beyond those that were manipulated may result in additional energy consumption in either or both comparison groups, with the potential to modify the effect of larger sizes due to the same ceiling effect described above.

It is important to avoid over‐interpretation of the results of the meta‐regression analyses we conducted due to their observational nature, limited statistical power and multiple tests, which meant heightened probability of type I (obtaining a false positive result) and type II (obtaining a false negative result) errors. These results should therefore be viewed primarily as generating hypotheses about potential effect modifiers that will need to be investigated in further studies, with patterns of results replicated, before more confident inferences can be drawn.

### Overall completeness and applicability of evidence

The evidence synthesised in this review was collected from 72 included studies that featured 107 eligible independent comparisons between two different sizes or shapes of portions, packages, individual units or tableware used to consume food products (69 of 72 included studies), or between two different sizes (lengths) of individual units of tobacco products (cigarettes) (3 of 72 included studies). The effective sample sizes feeding into meta‐analyses of outcome data collected from included food studies typically exceeded numbers generated by a conventional sample size calculation for a single adequately powered trial (that is, the optimal information size), which strengthens confidence that these studies were sufficient to enable us to address our first objective to assess the effects of eligible interventions on unregulated selection or consumption *of food products* in adults and children. Moreover, included food studies encompassed a range of participants in terms of their age, gender and other trait or state characteristics, a range of specific manipulations (for example, various types of foods), and a variety of eating or drinking contexts (encompassing both laboratory and naturalistic field settings). This confers a degree of confidence that our findings concerning food are likely to be widely applicable. It was also possible to exploit variations between included studies to investigate and attempt to explain observed variations in effects, addressing the second objective of this review to assess potential effect modifiers. This allowed us to report observed associations that, if confirmed by further research, may prove useful in configuring and targeting sizing interventions for maximum effectiveness (see Implications for practice).

Eligible studies typically investigated exposures that were one‐off or, if repeated, were repeated over relatively short time periods, and participants' selection and consumption responses were typically measured over correspondingly immediate or short time periods. In addition, the laboratory and naturalistic field settings in which participants were exposed and had their selection and consumption responses measured were often highly controlled by the researchers. These findings highlight the current lack of evidence to establish whether meaningful changes in the quantities of food people consume can be sustained over the longer term in response to prolonged or repeated exposures, under free‐living conditions.

In terms of intervention characteristics, the distribution of evidence for effects on selection and consumption of food was skewed towards pairwise comparisons in which the difference in relative size of the portions, packages, individual units or tableware was large. In addition, the absolute sizes investigated in food studies tended to be large. Therefore, while included food studies did cover a range of absolute and relative sizes, further studies focusing on smaller incremental changes at the smaller end of the portion size continuum are needed to strengthen the evidence base in this respect.

As highlighted above (see Summary of main results), knowledge of how the absolute sizes of food portions and packages investigated among studies included in this review compare with reference portion sizes for those specific foods (defined here as the size that is recommended to be consumed, or that is customarily consumed, in a single eating occasion, by one or more schemes for communicating portion size messages to consumers (Lewis 2012)) is critical to the interpretation of the results of this review. However, this relationship is both complex and dynamic. Alongside variation between specific food products within each scheme, there is also variation between reference portion sizes for comparable products between schemes and jurisdictions (for example, recommended amounts may be defined by food manufacturers, food retailers, government agencies or non‐governmental organisations, and may provide general advice or weight‐loss advice (Institute of Grocery Distribution 2008; Lewis 2012)). Schemes that provide reference portion size information based on amounts customarily consumed are also typically based on analysis of dietary intake within a defined population, which will also vary between population subgroups and over time; estimates from some schemes still in current use may therefore diverge from current dietary intakes due to their age (for example, the US Food and Drug Administration's Reference Amounts Customarily Consumed are largely based on data published in 1993 (USFDA 2014)). It is therefore important to highlight that our discussion of potential policy actions that would be consistent with the evidence in this review concerning the effects of size on consumption of food (see Implications for practice, below) is necessarily tempered by consideration of where this body of evidence may be located on the 'absolute size continuum'. Our observation that the absolute sizes investigated in food studies tended to be large is based primarily on comparison with external data, derived from ranges of typical dietary intakes (amounts customarily consumed in a single eating occasion), that were published in 2002 (Food Standards Agency 2002), which may not be transferable to the present day or other settings. The key message is that we urge caution in extrapolating the results of this review beyond the range of relative size differences between, and/or the absolute sizes of, portions, packages and tableware sizes investigated among included studies.

Specifically, the limited body of evidence identified for the consumption effects of exposure to different portion, package and tableware sizes at the smaller end of the size continuum means that we cannot be certain whether reducing portions at the smaller end of the size range can be as effective in reducing food consumption as reductions at the larger end of the range. There may also be some potential for unintended effects of exposure to small portions. Exposure to smaller portions than those typically encountered could sometimes lead to increased consumption. One possibility is that people may avoid selecting or consuming larger portions of products they perceive as unhealthy, but allow themselves to indulge when those products are presented in small sizes, thereby shifting from no consumption to some. The potential for unintended compensatory effects (that is, compensating for smaller portions by eating more later in the day), whilst not evident from individual studies we have encountered (Jeffery 2007; Kral 2004a; Lewis 2015; Vermeer 2011), is another related issue that deserves close attention.

We judged few participant samples in included food studies to be characterised by high levels of material or social deprivation; few studies measured participants' socioeconomic status and no studies reported effects disaggregated by socioeconomic subgroup. Moreover, evidence for effects on selection and consumption of food was derived mainly from studies conducted in US samples, with no included studies conducted in low or middle‐income countries (LMICs). These factors largely precluded any assessment of social differentiation in effects relevant to health equity (with the exception of gender ‐ see Effects of interventions, 'Potential modifiers of the effect of larger size on consumption') (see alsoObjectives). We have no reasons to expect that cognitive biases proposed as mechanisms by which exposure to these interventions may influence food selection and consumption (for example, 'unit bias') will differ substantively between people living in high‐income countries (HICs) and those living in LMICs (see How the intervention might work). However, people living in HICs *are* likely to have different personal and social (descriptive and injunctive) norms about what constitutes a suitable amount of food to consume than those living in LMICs and such factors have been proposed to influence the effects of exposure to larger sizes on food selection and consumption. A range of other social, cultural, economic and contextual differences surrounding diet‐related behaviours between people living in HICs and LMICs may also plausibly modify these effects. For these reasons, the predominance of US evidence may limit the applicability of findings of this review to LMICs (and also to other HICs) to some extent.

This review identified three studies that investigated the effects of exposure to longer versus shorter cigarettes on tobacco consumption (Jarvik 1978 (E1); Jarvik 1978 (E2); Russell 1980). We did not identify any tobacco studies investigating the effects of exposure to different sizes (or shapes) of cigarette packs, which may be an alternative target for interventions to reduce exposure to single cigarettes or packs containing smaller than standard numbers of cigarettes. Applicability of the evidence derived from the three included tobacco studies we did find, published in 1978 and 1980, may be limited by its age. The small effective sample size (six independent comparisons, 108 participants) contributing to our meta‐analysis from these studies further weakens confidence that they provided sufficient evidence to allow us to address the first objective of this review with respect to tobacco products. The true effect of exposure to longer versus shorter cigarettes on tobacco consumption is likely to be substantially different from our summary estimate. Results based on evidence from tobacco studies should therefore be interpreted with caution.

The most notable gap in this evidence base, however, was the absence of any randomised controlled trials investigating effects on unregulated selection or consumption of alcoholic beverage products. This finding is in keeping with the small proportion of studies on alcohol, compared with food products, which we found in a large scoping review of interventions that involve altering the properties or placement of objects or stimuli within small‐scale micro‐environments to change health behaviour, of which 'sizing interventions' was just one type (Hollands 2013a; Hollands 2013b). One possible reason for the current dearth of studies on alcohol is that this reflects the focus of recent alcohol policies on reducing consumption in harmful and hazardous drinkers through individual‐level interventions (Kaner 2009). Interventions that target price can reduce consumption of alcohol across populations (Holmes 2014; Wagenaar 2009), but such interventions are generally unacceptable to industry, politicians and the general public (Diepeveen 2013). More recent evidence regarding the harmful effects on population health of alcohol consumption at moderate levels (Rehm 2015) may extend the research focus to include interventions in micro‐environments such as those pertaining to size.

### Quality of the evidence

Ratings of the overall quality of evidence incorporated into this review ranged between moderate and very low, which leaves open the possibility that our estimates of intervention effects differ substantially from true effects. Confidence in estimates of effects was diminished by serious concerns about study limitations, which were primarily raised by unclear and incomplete reporting of study methods and procedures by authors of included studies. Indeed, we identified limitations in study reporting and/or conduct with respect to each of the domains judged most critical to 'Risk of bias' assessment in this review: random sequence generation (selection bias); allocation concealment (selection bias); blinding of participants and personnel (performance bias); and baseline comparability of participant characteristics between groups (other bias). Given the nature of the included studies, we could not identify any obvious reason to prevent the straightforward implementation of unbiased methods and procedures for random sequence generation and allocation concealment. The use of within‐subjects designs precluded the blinding of participants in over half of the included studies, but we did not judge lack of blinding to place studies at high risk of bias in this domain due to a general lack of evidence for the presence and potential influence of carry‐over effects among included studies. We did not consider blinding of personnel (that is, intervention providers) to be a relevant consideration in assessing risk of bias in included studies because personnel were not judged instrumental in delivery of the intervention. Finally, while it may not always be practical to test such differences in applied field settings, in many instances baseline comparability of participant characteristics between comparison groups can and should be examined.

We identified few concerns regarding inconsistency in study results, since in general large amounts of unexplained inconsistency did not remain following planned investigations of potential effect modifiers using meta‐regression analyses. There were no serious concerns about the directness of the assembled evidence either, since it was all derived from studies that directly compared the interventions in which we were interested, in groups of eligible participants, and incorporated direct (and typically objective) measures of unregulated selection or consumption.

We had no serious concerns about imprecision in relation to our estimates of the effects of exposure to larger (versus smaller) portion, package, individual unit or tableware size on unregulated selection or consumption of food, since (as noted above) effective sample sizes comfortably exceeded the numbers generated by conventional sample size calculations for single adequately powered trials (optimal information sizes). However, we did have serious concerns about imprecision in relation to our estimates of the effect of exposure to longer (versus shorter) cigarettes on consumption of tobacco, and of the effect of exposure to shorter, wider (versus taller, narrower) glasses or plastic bottles on consumption of non‐alcoholic beverages, based on consideration of both threshold optimal information sizes and confidence intervals.

### Potential biases in the review process

Whilst it is possible that we may have failed to identify every study eligible for inclusion in this review, we took several steps to minimise this risk, including our use of highly sensitive search strategies and backward and forward citation searches. We therefore consider it improbable that we have failed to identify sufficient relevant evidence to substantively alter our conclusions. The scope, scale and complexity of this review and its analysis meant that we took the pragmatic decision (in consultation with the Cochrane Public Health Review Group) to defer full integration of 11 further eligible studies identified by the updated search (30 January 2015) (Bajaj 2014; Haire 2014; Kral 2014; Marchiori 2014; Rolls 2014a; Smith 2013a; van Ittersum 2013; van Kleef 2014; Wansink 2013; Wansink 2014; Williams 2014), until the first major update of this review. However, the results of *preliminary* analyses of outcome data that could *provisionally* be extracted from each of these 11 further eligible studies (see Appendix 2) establish that there is minimal potential for the full integration of these studies to change the interpretation of the results of this review, and hence its conclusions, as currently reported in the Results, Discussion and Authors' conclusions.

### Agreements and disagreements with other studies or reviews

In a review of the effects of portion sizing published in 2014, Zlatevska and colleagues found that increasing portion size led to a small to moderate increase in consumption, reporting an effect size of *d* = 0.45 (Zlatevska 2014). This point estimate was similar to those we found in the current review and within its 95% confidence intervals. Results of moderator analyses conducted in Zlatevska and colleagues' review were again broadly consistent with our results. First, Zlatevska and colleagues similarly reported that the intervention effect was greater in adults than in children. Second, consistent with our findings regarding moderation by healthiness and by energy density of food, they reported a larger effect for snack foods (which are typically less healthy and more energy‐dense) than non‐snack foods. Contrary to the results of our analysis, however, they reported finding a larger effect among men than among women and a smaller effect among overweight participants than among participants who were not overweight. Discrepancies between the results of these analyses are expected since they used different data sets as a consequence of differences in their respective eligibility criteria, procedures and analytic methods. Although criteria for considering studies in Zlatevska and colleagues' review were broadly similar to those applied in this review, the former focused exclusively on food, did not appear to exclude studies in which participants' consumption was regulated by either explicit instructions or some other action of the researcher, and additionally included studies that measured intended but not actual consumption. Zlatevska and colleagues' review did not include coverage of evidence for the effects of package, individual unit or tableware size on consumption and did not investigate food selection as an outcome. Indeed, we are not aware of any relevant, previously published reviews that investigate either the effects of exposure to food packages or to individual food units of varying size (and only one that investigates dishware size ‐ see below in this section), nor that investigate food selection as an outcome.

We are aware of only one other systematic review, published in 2013 (Small 2013), which ‐ like ours and Zlatevska and colleagues' reviews (Zlatevska 2014) ‐ encompassed evidence for the effects of exposure to food portions of varying size on energy intake among well and normally developing children. Small and colleagues aggregated evidence from six eligible primary studies ‐ all randomised controlled trials that are fully incorporated into our review (Fisher 2003; Fisher 2007a; Fisher 2007b; Fisher 2007c; Rolls 2000; Spill 2010) ‐ using a narrative synthesis and reported a similar finding: that larger served portions resulted in greater daily energy intake among participants (Small 2013).

In a review of the effect of dishware size on consumption of food published in 2014, Robinson and colleagues reported results consistent with no effect of dishware size on consumption (standardised mean difference (SMD) ‐0.18, 95% confidence interval (Cl) ‐0.35 to 0.00, P value = 0.05) ‐ although we note that the authors reported "a small effect that was not statistically significant", with exposure to larger dishware leading to greater consumption (Robinson 2014). Although this review again differed from ours with respect to its inclusion criteria (for example, non‐randomised studies were eligible and targets of the manipulation were restricted to bowl size or plate only), its estimate of this effect overlaps considerably with our corresponding estimate for the effect of tableware size on consumption (see Figure 7).

## Authors' conclusions

**Implications for practice**

Due to limitations in the scope, quality and quantity of relevant research evidence that is currently available (including in the case of alcohol, a complete absence of evidence), the key implications of this review for public health policy and practice, set out below, concern food. We are unable to highlight any clear implications for alcohol or tobacco policy. In addition, all of the currently available evidence derives from studies conducted in high‐income countries (HICs) (predominantly in the USA), with no evidence from studies conducted in low and middle‐income countries (LMICs). The applicability of our findings to public health decision‐making in LMICs therefore remains uncertain. Moreover, we found insufficient evidence to indicate whether portion size effects may vary in HICs between people according to their socioeconomic status or levels of social or material deprivation. As such, it is unknown whether and how interventions that reduce, or moderate the effects of, exposure to larger‐sized portions, packages, individual units and tableware would impact on existing inequalities between socioeconomic groups in health‐related behaviours or corollary health outcomes.

The principal finding of this review is that people consistently consume more food and drink when offered larger‐sized portions, packages or tableware than when offered smaller‐sized versions. This suggests that policies and practices that successfully reduce, or moderate the effects of, exposure to larger‐sized portions, packages, individual units and tableware – in and outside the home – can contribute to meaningful reductions in the quantities of food and non‐alcoholic beverages people select and consume in the immediate and short term. Actions to halt, reverse or mitigate the effects of recent trends towards larger portions (Young 2002; Young 2012) may therefore be justified on public health grounds. The portion sizes investigated in included food studies were typically at the larger end of the absolute size continuum, therefore the evidence in this review confers confidence that reducing the sizes of portions and packages that are large in absolute terms can achieve effects of the magnitude estimated. However, the evidence in this review neither convincingly supports, nor undermines, claims that making sizes smaller than have become typical or standard can be expected to have similarly meaningful impacts on food selection or consumption. In response to these findings, possible intervention strategies targeting the physical environment (in public sector and/or commercial sector settings) include: regulatory and legislative frameworks, or voluntary agreements with the food industry, which result in alterations in portion size (Bryden 2013; Hsiao 2013); reducing default serving sizes of energy‐dense foods and drinks where these are large in absolute terms, or providing smaller crockery, cutlery and glasses for use in their consumption; and various 'choice architecture' interventions in micro‐environments such as restaurants or supermarkets (Hollands 2013a). Examples of the latter may include, for example, reducing the availability of larger portion, package and tableware sizes; placement of larger portion sizes further away from purchasers; or demarcation of single portion sizes in packaging through wrapping or a visual cue.

Other potential intervention strategies targeting the economic environment include eliminating pricing practices whereby larger portion and package sizes cost less in relative (and sometimes absolute) monetary terms than smaller sizes and thus offer more value for money to consumers (Steenhuis 2009) and restricting price promotions on larger‐sized packages. There is limited and equivocal evidence for the effectiveness of interventions that do not seek to directly alter the availability or cost of larger sizes, but instead aim to educate people about appropriate portion sizes ‐ for example, by providing information about the portion size effect or the number of portions in a serving (Cavanagh 2013; Spanos 2015; Versluis 2015). This does not, however, rule out a potential role for social marketing campaigns to raise awareness and engender public acceptability of the public health case for interventions to reduce or moderate the effects of exposure to larger‐sized portions of food and drink. Such approaches may help to create the social and political conditions necessary to enable effective interventions to be implemented. The design of interventions targeting physical or economic environments, or aiming to educate or otherwise create enabling social, cultural and political conditions for effective intervention of this kind, will need to remain sensitive to local cultural and socioeconomic circumstances in different implementation settings (Huang 2015; Rychetnik 2002).

With the exception of directly controlling the sizes of the foods people consume, assessment of the effectiveness of possible intervention strategies was beyond the scope of this review. However, findings from relevant published evidence syntheses present a mixed picture. For example, a recent economic analysis ranked interventions comprising reductions in portion size of foods and beverages in various contexts highest, among a portfolio of evaluated policy levers, for reducing the population health burden of obesity (McKinsey Global Institute 2014). However, the portion size component of this economic analysis, based on a smaller, overlapping set of studies compared to the current review, assumed that the same sizes of effects estimated in source studies (which measured consumption effects over immediate or short time periods in response to one‐off or short‐term exposures) will be sustained and cumulative over people's lifetimes in response to repeated exposures (Corrine Sawyers, personal communication 2015). In addition, a 2009 review of interventions aiming to address the negative influences of portion size effects on consumption that formed part of the evidence base used in this economic analysis found few studies, and these showed mixed effects (Steenhuis 2009) (see also Implications for research).

This review suggested that the effect of larger size on consumption may be robust to variation between interventions in terms of several of their key characteristics and those of their participants. For example, we did not find evidence that the intervention effect varied substantively between men and women, nor by people's body mass index, susceptibility to hunger, or tendency to consciously control their eating behaviour. These findings are essentially observational, should be interpreted with caution and would need to be confirmed by future studies before they can be distilled into clear policy implications. However, if confirmed, these null findings would add credence to the claim that people are susceptible to environmental influences on food consumption that operate independently of individual characteristics that are often portrayed as the main drivers of over‐consumption; and indicate the potential for effective interventions targeting portion, package and tableware size to reduce consumption among a broad range of people. Other tentative findings suggested that such interventions may be particularly effective in reducing consumption among adults and that reductions in exposure to larger portion sizes of less healthy and of more energy‐dense foods ‐ those foods whose over‐consumption is most damaging to health ‐ might usefully be the principal target for policy action. We cannot readily explain these results but note that they replicate those of another recent review of food portion size effects (Zlatevska 2014). It may be that people have reduced ability to regulate their consumption of less healthy and more energy‐dense foods in response to external cues ‐ either due to these properties or other associated properties (for example, palatability) ‐ thereby increasing the potential for size to influence quantity consumed. However, studies included in this review that experimentally manipulated both size and energy density variables did not find interaction effects consistent with this proposal (Devitt 2004; Rolls 2006b; Rolls 2010a (E1); Rolls 2010b (E2)).

Irrespective of uncertainty regarding the mechanism of this moderation, these findings would be encouraging from a public health perspective if replicated by further research for two reasons. First, they highlight the possibility that the largest reductions in consumption might be achieved by reducing exposure to larger sizes of those products for which a reduction is likely to be most beneficial for health. Second, they are consistent with the proposal that a 'portion size effect' is still present when people are exposed to larger sizes of healthier and less energy‐dense foods, suggesting that interventions that successfully *increase* people's exposure to larger portion sizes of healthier, low energy‐dense foods such as vegetables may still be an effective strategy for increasing consumption of these foods (Rolls 2014b).

Whilst this review found evidence of moderate overall quality indicating that people select and consume more food when exposed to larger‐sized portions, packages, individual units and tableware, it is important to highlight that these findings were derived from studies that typically investigated exposures that were one‐off, or if repeated at all, were repeated over relatively short time periods, often under highly controlled experimental conditions. The longer‐term sustainability of the effects of prolonged or repeated exposures, and effects under free‐living conditions, therefore remain to be established. This underscores that the long‐term effectiveness of interventions introduced with the aim of reducing people's exposure to larger portion, package and tableware sizes is currently unknown (worldwide) and will be subject to all the challenges and complexities of achieving effective and sustained implementation at scale.

One such complexity is the actual and perceived monetary costs (prices) of food products, which have been proposed to modify the effects of portion or package size on food consumption (Steenhuis 2009). Evidence to inform understanding of potential interactions between product size and cost appears to be lacking (that is, no studies eligible for inclusion in this review investigated such interactions). Another is that scaling up interventions of this kind (that is, increasing their geographic coverage and scope with the corollary potential to influence the behaviour of large numbers of people in a wider range of eating and drinking contexts) would involve their introduction into a complex food environment populated by a multitude of available food products other than those having their sizes directly or indirectly altered. For example, in homes, shops and restaurants people have access to additional quantities of a wide variety of foods. The potential for compensatory consumption of other foods is not elucidated by this review.

A further set of challenges to implementing policies to reduce exposure to larger‐sized portions of food and non‐alcoholic beverages is provided by the commercial and legal contexts in which these products are sold. The likely strength of resistance among food and beverage industry representatives was evident in an unsuccessful attempt in New York to cap the portion sizes of sugar‐sweetened beverages sold in restaurants and other venues serving food (Gabbatt 2013; Grynbaum 2012). However, policies of this kind appear to be more acceptable among the general public (Diepeveen 2013; Petrescu under review), which raises the possibility of pursuing alternative strategies such as engaging civil and other organisations at local, national and international levels to advocate for reconfiguration of systems of production and consumption (Freudenberg 2014; Jackson 2009; Skidelsky 2013).

In summary, this review provides the most conclusive evidence to date that people consistently consume more food and drink when offered larger‐sized portions, packages or tableware than when offered smaller‐sized versions. This suggests that policies and practices that reduce, or moderate the effects of, exposure to larger sizes can contribute to meaningful reductions in the quantities of food and non‐alcoholic beverages people select and consume. This may justify actions to reduce the size, availability and appeal of food portion, package and tableware sizes that are large in absolute terms. However, it is uncertain whether reducing portions at the smaller end of the size range can be as effective in reducing food consumption as reductions at the larger end of the range. We are unable to highlight clear implications for tobacco or alcohol policy due to identified gaps and limitations in the current evidence base.

**Implications for research**

The implications for research set out below are based on gaps and uncertainties identified by reviewing the current evidence base, which (as highlighted above ‐ see Implications for practice) derives exclusively from studies conducted in HICs. Although it is feasible that the implications may also be applicable to research in LMICs, the lack of experience of conducting studies of this kind in LMICs leaves open the possibility that LMIC‐specific research issues may emerge if such experience accumulates.

This review found no evidence from randomised controlled trials for the effects of altering size or shape on selection or consumption of alcoholic beverages and identified only five eligible studies that included a focus on non‐alcoholic beverages. More evidence for intervention effects on unregulated selection and consumption is needed with respect to both of these product categories to inform the design of interventions to reduce their consumption and ameliorate associated impacts on health inequalities. The social patterning of harmful alcohol use and its health consequences is well documented (Fone 2013), whilst sugar‐sweetened beverage consumption, which represents the largest source of added sugar in UK and US diets (Tedstone 2014; Welsh 2011), is also socially patterned, with heavy consumption being more likely among adults and children from lower socioeconomic status backgrounds (Han 2013). Furthermore, few eligible tobacco studies were identified and those we did find compared the effects of exposure to longer versus shorter cigarettes, the most recent published in 1980 (Russell 1980). We found no studies of other conceivable tobacco product size or shape manipulations, such as cigarette packs sized to contain different numbers of cigarettes. This is notable given the European Union decision (Tobacco Products Directive: European Union 2014) to ban smaller cigarette packs containing fewer than 20 cigarettes from 2016. This decision was based on factors related to both harmonisation of trade and public health, including implementation of the WHO Framework Convention on Tobacco Control (WHO FCTC), which entered into force in 2005 (World Health Organization 2003). Article 16 of the WHO FCTC prohibits the sale of cigarettes individually or in small packets on the basis that this increases their affordability to children, which aligns with evidence indicating that price is an important factor in determining smoking initiation among children and young people (Godfrey 2009; NICE 2008; Pierce 2012). As such, most of the evidence incorporated into this review relates to the effect of exposure to larger versus smaller‐sized portions, packages, individual units and tableware on the selection and consumption of food (including non‐alcoholic beverages, although as noted above, these were underrepresented). However, several of the implications for research that we highlight below in relation to food studies may be transferable for consideration in the development of future research on alcohol and tobacco products.

The body of evidence in this review clearly indicates a potential role for interventions that successfully reduce exposure to larger portion, package or tableware sizes, or mitigate the effects of such exposure, to help change people's food, energy and nutrient intake. As noted above (see Implications for practice) the range of possible intervention strategies includes regulatory and legislative frameworks that mandate alterations in size, voluntary agreements with industry, choice architecture interventions, interventions targeting price, and educational and social marketing interventions (all of which fell outside the scope of this systematic review). Whilst we are not currently aware of any systematic reviews that have aimed to assess the effectiveness of such interventions, a traditional literature review of interventions designed to address the negative influence of portion size on energy intake, published in 2009, identified only five relevant primary studies (all conducted in HIC settings) investigating different specific interventions involving: provision of nutritional information on product labelling; nutritional labelling with price promotion; and restrictions placed on customers' purchasing of larger portions (Steenhuis 2009).

These observations point to the need for further research in two specific areas. First, further new primary studies of the effects of exposure to larger versus smaller‐sized portions, packages, individual units and tableware on selection and consumption of food (that is, studies meeting the eligibility criteria for this review) are needed. Second, a systematic review of evidence for the effectiveness of interventions to reduce exposure to larger sizes, or to mitigate the effects of exposure to larger sizes (that is, studies outside the scope of this review), may be needed, possibly followed by further, new primary studies of such interventions and policies. Critically, in order to generate evidence for effectiveness and the sustainability of effects, future primary studies in both of these identified areas of research should evaluate people's selection and consumption responses over longer time periods in 'real world' environments (such as homes, shops and restaurants) and under free‐living conditions as far as possible (that is, with minimal research‐imposed constraints on target behaviours and environments). This may mean, for example, studying interventions implemented within otherwise unaltered restaurant or shop environments in which participants are able to freely select and consume from a typically wide range of products and over a number of weeks or months. Moreover, the studies need to be designed to contribute to summary estimates of corollary impacts on health inequalities. This would not only ensure that policies found to be effective do not cause "intervention generated inequalities" (Lorenc 2013), but would also increase understanding of their potential to reduce inequalities arising from excessive consumption of less healthy products by more socially and materially deprived people, such as those with low levels of education or income. None of the included studies assessed (or indeed were powered to assess) the moderation of intervention effects by socioeconomic status, or potential interactions between product size and cost in influencing selection *with* purchasing.

With respect to the first specific area in which research is needed, further new primary studies of intervention effects on selection and consumption of food could feed into an updated synthesis that would have the potential to increase our confidence in summary estimates of these effect sizes and reduce associated uncertainty. This would have the potential to strengthen our qualified finding that portion, package, individual unit and tableware size represent promising targets for public health intervention to change the quantities of food, energy and nutrients people select consume. Any such studies should include further investigation of the tentative findings of this review in relation to potential effect modifiers.

There is also considerable scope for any such further studies to help fill gaps in the current evidence base that we have identified in this review. As well as the critical need to generate evidence for the effectiveness of prolonged or repeated exposures over longer time periods and with minimal research‐imposed constraints on behaviour, this could usefully include investigations of effects in a wider range of participant subgroups, such as adolescents and older adults. New primary studies could also expand the current evidence base by investigating effects in a wider set of field settings than were represented among studies included in this review, which were predominantly conducted in restaurants or in school or workplace cafeterias. Given that most food and drink is purchased in shops for consumption in the home (DEFRA 2013; Harnack 2000; Smith 2013b), research to examine intervention effects in these contexts is especially needed.

Critically, any further primary studies of this kind should also feature smaller absolute sizes, and smaller magnitudes of size difference between the compared portions, packages, individual units or items of tableware. More evidence from studies presenting participants with smaller absolute sizes is needed to confer a higher degree of confidence than can be derived from the body of evidence in this review that reducing sizes to amounts smaller than have become typical or standard has the potential to be an effective intervention strategy (see Overall completeness and applicability of evidence and Implications for practice).

With respect to the second specific area in which research is needed, it would be useful ‐ especially given the age of Steenhuis and colleagues' traditional literature review of interventions to address negative influences of portion sizing (Steenhuis 2009) ‐ to conduct a preliminary scoping exercise to ascertain whether sufficient primary studies of various possible interventions to reduce, or mitigate the effects of, exposure to larger food sizes have been conducted to warrant a new systematic review. If not, new primary studies of the effectiveness of a broader range of possible interventions than were identified in the earlier review (Steenhuis 2009) should be undertaken, encompassing regulatory, non‐regulatory and pricing strategies (highlighted above in this section). The appropriate balance between the two areas of primary research we have highlighted will depend in part on the extent to which overall moderate quality evidence for a small to moderate effect of size on consumption is regarded as a sufficient basis for policy action to mitigate the undesirable consequences of such effects.

Finally, the evidence base for the effects of these kinds of interventions would be substantively improved by better‐conducted and reported primary studies. In the process of conducting this review we encountered some egregious examples of study reporting ‐ such as reports lacking basic descriptive statistics for outcome data, or key details of study methods and procedures ‐ and unwillingness or inability of some study authors to provide additional data missing from study reports. This may be attributable in part to the age of some of the included studies and the slow diffusion of study reporting guidelines that have become established in medical research into the psychology and nutrition literatures (Grant 2013; Mayo‐Wilson 2013). Primary researchers should ensure that their study reporting complies with CONSORT‐SPI – a forthcoming extension of the Consolidated Standards of Reporting Trials (CONSORT) Statement, which has specifically been developed for randomised controlled trials of social and psychological interventions (Montgomery 2013) – and that it includes descriptions of interventions (exposures) sufficiently detailed to allow their replication (Hoffmann 2014). To maximise the optimal use and reuse of primary research, new study authors and those of existing studies will ideally ultimately provide open access to their complete, anonymised individual participant‐level data sets in machine‐readable format. In principle it would be possible to synthesise these data using individual participant data meta‐analysis methods (Stewart 2011), with the potential to reduce current levels of uncertainty concerning main effects and effect modifiers, and to generate findings with much sharper implications for policy concerning portion, package and tableware size interventions.

In summary, this review highlights the potential value of further research to establish sizes of effects of exposure to differently sized alcoholic beverage products. Further research may also be conducted to reduce uncertainty about the sizes of effects of exposure to differently sized portions and packages of food and (in particular) non‐alcoholic beverages, and of tableware used in their consumption, especially with regards to smaller absolute sizes and magnitudes of difference in relative sizes, and the sustainability of such effects, in 'real world' environments. Finally, effect sizes of interventions to reduce, or mitigate the effects of, exposure to larger‐sized food portions, packages and tableware, need to be established. Such interventions encompass a range of potential strategies, including changes to physical and economic environments designed to reduce the size, availability and/or appeal of larger food portions.

## Feedback

### Portion package or tableware size for changing selection and consumption of food alcohol and tobacco, 17 September 2015

#### Summary

The most significant patient‐important outcomes of this important study are reported in an incomplete and nationally biased fashion.

Abstract and Plain Language Summary are both UK‐biased, at expense of US population apparently most in need of reducing portion sizes.

1. Both Abstract and Plain Language Summary note majority of studies were done on US adults.

1a. Abstract:

"More studies investigated effects among adults (76% (55/72)) than children and all studies were conducted in high‐income countries ‐ predominantly in the USA (81% (58/72))."

1b. Plain Language Summary:

"The average age of participants in the different studies ranged from three to 55 years, with more studies involving adults than children and most conducted in the USA."

2. Both note size of effect, if sustained, could lead to patient‐important outcome of significant caloric reduction.

2a. Abstract:

"The size of this effect suggests that, if sustained reductions in exposure to larger‐sized food portions, packages and tableware could be achieved across the whole diet, this could reduce average daily energy consumed from food by between 144 and 228 kcal (8.5% to 13.5% from a baseline of 1689 kcal) among UK children and adults."

2b. Plain Language Summary:

"If an effect of this size were sustained across the whole diet it would be equivalent to around a 12% to 16% change in average daily energy intake from food among UK adults."

Again, no mention of US, comprising 81% of the RCTs, even though the patient‐important outcome of the projected sustained effect for the US population is almost *double* that for the reported UK population.

Compare:

"The data indicate that people consistently consume more food and drink when offered larger‐sized portions, packages, or tableware than when offered smaller‐sized versions. This finding suggests that, if sustained reductions in exposure to large sizes could be achieved across the whole diet, this could reduce average daily energy consumed from food by 10% to 17% among adults in the UK (equivalent of up to 290 kcals per day) or by 18% to 30% among US adults (equivalent of up to 547 kcals per day). The researchers did not find that the size of this effect varied substantively between men and women, or by people’s body mass index, susceptibility to hunger, or tendency to consciously control their eating behaviour."

Source?

"Media release from the University of Cambridge and Cochrane"

September 15, 2015

http://www.cochrane.org/news/portion‐package‐or‐tableware‐size‐changing‐selection‐and‐consumption‐food‐alcohol‐and‐tobacco

As a Wikipedia editor I rely on both the Abstract and the Plain Language Summary to help me in summarizing, in my own words, Cochrane reviews and other original research. (I also search for reliable secondary sources that critique same.) I do not generally cite press releases, no matter how well written.

I hope this communication oversight may be corrected in the near future.

Regards,

Paul S. Wilson

("Paulscrawl" on Wikipedia)

P.S.

I have already cited the review on two Wikipedia articles (content &/or location will no doubt be changed by myself or other editors; just a start for today):

Portion size

https://en.wikipedia.org/wiki/Portion_size

Weight management

https://en.wikipedia.org/wiki/Weight_management

I have modified the conflict of interest statement below to declare my interests:

I certify that I have no affiliations with or involvement in any organization or entity with a financial interest in the subject matter of my feedback.

I have been granted a Cochrane Library account (partner access donation) through the Wikipedia Library.

#### Reply

We thank Paul S. Wilson for the feedback submitted and value his contribution made on Wikipedia.

Feedback by readers provides the opportunity to improve the preparation and usefulness of our public health reviews. After consideration according to policy, It was the decision of the editors that the feedback will be used by the review authors to improve the clarity in the future update of the review. The authors have provided the following response

We thank Paul Wilson for this feedback and commend the valuable work done by editors like Paul to ensure health‐related Wikipedia articles incorporate reliable, up‐to‐date evidence for the effects of interventions, including evidence from Cochrane reviews.

The extracts cited in Paul’s feedback re‐express a summary effect size – namely, our summary estimate of the size of the effect of exposure to larger (versus smaller) sized portions, packages, or tableware on quantities of food or non‐alcoholic drinks consumed among included studies of adult participants – using a more familiar metric than units of standard deviation (standardised difference in means, hereafter ‘SMD’), in order to illustrate, and thereby facilitate, its interpretation. The summary effect size in this specific case was SMD 0.46, 95% CI 0.40 to 0.52. In accordance with guidance in The *Cochrane Handbook for Systematic Reviews of Interventions* (Chapter 12, Section 12.6.4), our objective was to re‐express this summary effect size in terms of the equivalent (absolute and relative) change in daily energy intake from food among population representative samples of adults.

Evidence from Cochrane reviews is intended for use to inform decision‐making internationally and in this context we saw no compelling evidence or rationale to choose one country over another for example illustrations (especially given our findings suggested the ‘portion size effect’ is consistent across a range of contexts, settings and populations). Origins of the evidence in the review (predominantly from US studies) were one consideration; another was generalizability of the example to other countries (and, from this perspective, high levels of food and drink consumption in the USA could be seen as representing ‘outlier’ values). It was also beyond the resources available to be allocated to developing illustrations for use to re‐express summary effect sizes among population representative samples from all countries that could use the findings of this review to inform decisions. As such, the series of judgements that led to the focus on UK data in order to illustrate this (and other) summary effect sizes for patient important outcomes were made on pragmatic grounds; balancing the aim of maximising fidelity between the illustrations and the evidence in the review, with the availability of data and resources to perform supplementary, secondary analyses of population representative datasets that would be needed in many cases.

In principle, we agree that it would be useful to present US (and other country‐level) illustrations of effect sizes in the published full review. When completing the first major update of this review, we will therefore update the ‘Discussion > Summary of main results section of the review’ to include the equivalent change in average daily energy intake from food among US adults, alongside the corresponding UK illustration.

More generally, we also plan to revisit the scope of illustrations to re‐express this summary effect sizes in planning for the first major update of this review, once again taking into account the balance between the added value and incremental costs of conducting the required secondary analyses of key datasets.

#### Contributors

Baker P, Hollands GJ, Shemilt I, Marteau TM, Jebb SA, Lewis HB, Wei Y, Higgins JPT, Ogilvie D

### Portion, package or tableware size for changing selection and consumption of food, alcohol and tobacco, 12 March 2017

#### Summary

I read an article in the Conversation yesterday which contradicts the findings of this review in relation to size of tableware. https://theconversation.com/do‐smaller‐plates‐make‐you‐eat‐less‐no‐74181. This is an extremely high profile and influential review and I wonder if policy makers will use it to implement measures to reduce tableware size alongside portion and packaging sizes without good evidence. Smaller tableware may even increase consumption. Portion size and tableware size intervention studies have been conflated in this review and I wonder if that has muddied the waters unnecessarily. The way these interventions might work (or not) is complex and different depending on whether it is portion size or tableware size you are manipulating.

The author of the article in the Conversation also highlights the serious question nmark over the work of Brian Wansink (https://www.theguardian.com/science/head‐quarters/2017/mar/02/fresh‐concerns‐raised‐over‐academic‐conduct‐of‐major‐us‐nutrition‐and‐behaviour‐lab) who is either the first or second author of more than ten of the included studies in this review. I didn't find a risk of bias table for the 72 individual studies...was there one?

I do not have any affiliation with or involvement in any organisation with a financial interest in the subject matter of my comment.

#### Reply

Thank you for your comments.

First, we are aware of this article in ‘The Conversation’ by Eric Robinson and the published meta‐analysis (Robinson et al, 2014) that is presented as supporting evidence for its central claim that “smaller plates may not reduce how much people eat”. We discuss the findings of this earlier meta‐analysis in our Cochrane review (see ‘Agreements and disagreements with other studies or reviews’) and highlight some differences in its methods. While you are correct that our pre‐specified primary analysis of the effect of larger (versus smaller) size on consumption of food combined outcome data from studies that investigated portion, package and tableware size, we also conducted a pre‐specified analysis to investigate potential differences in this effect between subgroups of included studies targeting portion, package or tableware size. The latter subgroup analysis did not find evidence for a difference in effect sizes between these subgroups. Moreover, we also presented a figure (Figure 7) to illustrate estimated effect sizes specific to each of these subgroups. Figure 7 shows that our estimate of the SMD (95% CI) among studies of tableware size was 0.29 (0.07, 0.51), which considerably overlaps with the corresponding estimate in the Robinson et al review: SMD ‐0.18 (0.00, ‐0.35), which given the differing direction of effect is equivalent to SMD 0.18 (‐0.00, 0.35). Notably, *both* of these point estimate effect sizes are consistent with a finding of ‘increased consumption’ among participants exposed to larger sized tableware; in the latter case, the lower bound of the 95% confidence interval is *also* consistent with ‘no effect of larger size on amounts consumed’, while in our review, the lower bound remains consistent with ‘increased consumption’ among groups exposed to larger sizes).

Therefore, contrary to claims in the article in The Conversation, we maintain that actions to reduce tableware size *are* compatible with current *cumulative* research evidence, as represented by the relevant summary effect sizes estimated in *both* of these reviews. However, critically, the overall GRADE rating applicable to our estimate of the ‘tableware‐specific’ effect size (see Figure 7) is ‘moderate’ (rated down by one level due to our concerns about study limitations ‐ risk of bias), which means that *further research is likely to have an important impact on our confidence in the estimate of effect and may change the estimate*. This finding explicitly leaves open the possibility that our estimate of the (summary) effect size could change when this Cochrane review is updated to integrate further outcome data from recent, new primary studies (including ‐ but likely not limited to, given that systematic searches across multiple databases will be run – those mentioned in in ‘The Conversation’ article). We recognise that this epistemic uncertainty – which in this case concerns both the size and direction of the ‘true’ effect (as well as potential effect modifiers) – may engender caution among policy makers who may be considering introducing measures to reduce tableware size. We further acknowledge that, while our current review finding suggests that use of larger sized tableware increases consumption, it is not yet known by how much tableware size can be reduced without leading to compensatory behaviour (for example, re‐filling a small plate), which could cause an overall increase in the amounts of food people consume. At the time of publication of the current version of our review, further research studies (such as Robinson et al’s subsequent 2016 study on dishware size) were needed to address this more specific question. Finally, we also note that, given that current evidence is predominantly laboratory‐based, unless policy makers do implement measures to reduce tableware size in real‐world settings and evaluate the impacts, we will never generate the new evidence required to resolve the uncertainty about the effectiveness of this approach as a public health intervention.

Second, we share the alarm you express concerning the recent, widespread coverage of apparent discrepancies and statistical errors identified in published reports of Brian Wansink’s research studies. We highlighted in our Cochrane review that Wansink was unable or unwilling, upon request, to provide us with key items of numerical data that were missing from, or unclear in, published reports of included studies for which he is the corresponding author (see Appendix 2 and Characteristics of Included Studies tables). However, whilst a recent blog has highlighted statistical errors in two of Wansink’s studies included in this Cochrane review (link to: http://www.timvanderzee.com/the‐wansink‐dossier‐an‐overview/), the identified errors do not relate to any data analysed in our review, and neither of the two studies contributed outcome data to our meta‐analysis investigating the effect of larger (versus smaller) size on food consumption. With specific regards to Figure 7, Wansink is a co‐author of one included study that investigated the effect of larger (versus smaller) tableware size on consumption (van Kleef 2012). However, no statistical errors or discrepancies have to date been identified in the latter study, and the result of the corresponding meta‐analysis, and its interpretation, are insensitive to the inclusion/exclusion of this study’s outcome data.

In the event that any study included in our Cochrane review is retracted, or statistical errors are identified in their numerical outcome data, we will reconsider whether to integrate that study’s data into updated meta‐analyses conducted as part of any future update of this Cochrane review. While, in our judgment, all of Wansink’s studies that are currently included in our review are at overall high or unclear risk of bias, the same is true of all 72 studies included this review, so Wansink’s studies are not unique in this respect. According to our published protocol for this Cochrane review, had there been studies judged at overall low risk of bias with respect to either outcome (that is, ‘selection’ or ‘consumption’), we would have included the study‐level risk of bias judgment as a covariate in the final stage of our planned meta‐regression analyses (Hollands 2014). In practice, since no included studies were judged to be at overall low risk of bias with respect to either outcome, the potential association between this covariate and estimated effect sizes could not be investigated as planned. However, study‐level judgments concerning risk‐of‐bias did explicitly feed into GRADE ratings assigned to each estimate of effect. This meant that confidence in (summary) estimates of effect was invariably rated down one level for serious concerns about study limitations (risk of bias), which (at best) led to an overall GRADE rating of ‘moderate’; meaning (as above) that *further research is likely to have an important impact on our confidence in the estimate of effect and may change the estimate*.

Finally, we did generate a table showing risk of bias judgments (by risk of bias domain and outcome) for each of the 72 individual studies included in the current version of this Cochrane review. However, this figure was excluded from the current published PDF version of the full review at the request of editors, because it could not legibly be printed using extant Cochrane publication software. In the current published version, risk of bias judgments (by risk of bias domain and outcome) are instead presented for each of the 72 individual studies in Characteristics of Included Studies tables (along with information supporting each judgment), and are summarised in Figure3.

In conclusion, our review currently provides the most robust estimate of the effect size of portion, package and tableware size on selection and consumption, not undermined by the concerns raised in this comment.

#### Contributors

Commenter ‐ Caroline Struthers,Education and training manager, EQUATOR Network

Responder (on behalf of the author team) ‐ Gareth Hollands, Behaviour and Health Research Unit, Institute of Public Health, University of Cambridge School of Clinical Medicine

## What's new

**Table:** 

**Date**	**Event**	**Description**
9 November 2018	Amended	Notes added to Wansink 2005e reference and description in Characteristics of Excluded Studies table indicating that this excluded study (due to ineligible study design) is an article that has since been retracted by JAMA (September 2018).
11 October 2018	Amended	Published note added in response to recent retraction of several studies by Brian Wansink
31 March 2017	Feedback has been incorporated	Feedback and authors' response added

## History

Protocol first published: Issue 4, 2014 Review first published: Issue 9, 2015

**Table:** 

**Date**	**Event**	**Description**
6 March 2017	Amended	Footnote 4 and 6 corrected in SOF table 4
29 October 2015	Feedback has been incorporated	Feedback submitted and responded to by authors

## Notes

*From the author team, 10 October, 2018, in response to recent retraction of several studies by Brian Wansink*

On the 19^th^ September 2018, JAMA, JAMA Internal Medicine and JAMA Pediatrics retracted six articles on which Brian Wansink (John Dyson Professor of Marketing at Cornell University), was an author (https://media.jamanetwork.com/news‐item/jama‐network‐retracts‐6‐articles‐that‐included‐dr‐brian‐wansink‐as‐author/). Given seven previous retractions, this means that 13 of his articles have been retracted as of 10^th^ October 2018 (http://retractiondatabase.org/RetractionSearch.aspx#?auth%3dWansink). The retracted articles are listed at the end of this note.

None of the 13 retracted articles authored by Wansink were included in this Cochrane review (one was excluded due to ineligible study design). The results and conclusions of the review are therefore not affected.

Other articles on which Wansink is an author, and which have not been retracted, were included in this review. It includes 72 studies, of which 13 studies were authored by Wansink.

The effects reported in this review are uncertain, attributable in part to evidence that is at significant risk of bias with, at best, GRADE ratings of ‘moderate’ (meaning that further research is likely to have an important impact on our confidence in estimated effects). These retractions do, however, introduce additional uncertainty regarding the veracity of other studies Wansink has authored, including those contributing to this review. Should any study included in this review be retracted, we will withdraw that study’s data from updated meta‐analyses conducted as part of future updates of this Cochrane review.

*Gareth Hollands and Theresa Marteau, on behalf of the author team*

Retracted studies (as of 10th October 2018)

Wansink B, Tal A, Shimizu M (2012). First foods most: after 18‐hour fast, people drawn to starches first and vegetables last. Arch Intern Med. 172(12): 961‐963.

Tal A, Wansink B (2013). Fattening fasting: hungry grocery shoppers buy more calories, not more food. JAMA Intern Med. 173(12): 1146‐1148.

Tal A, Zuckerman S, Wansink B (2014). Watch what you eat: action‐related television content increases food intake. JAMA Intern Med. 174(11): 1842‐1843.

Wansink B, Cheney MM (2005). Super Bowls: serving bowl size and food consumption. JAMA. 293(14): 1727‐1728.

Wansink B, Payne C, Werle C (2008). Consequences of belonging to the “clean plate club”. Arch Pediatr Adolesc Med. 162(10): 994‐995.

Hanks AS, Just DR, Wansink B (2013). Preordering school lunch encourages better food choices by children. JAMA Pediatr. 167(7): 673‐674.

Vuorinen A‐L, Strahilevitz MA, Wansink B, Safer DL (2017). Shifts in the Enjoyment of Healthy and Unhealthy Behaviors Affect Short‐ and Long‐Term Postbariatric Weight Loss. Bariatric Surgical Practice and Patient Care. 12(1): 35–42.

Wansink B, Just DR, Payne CR, Klinger MZ (2012). Attractive names sustain increased vegetable intake in schools. Prev Med. 55(4):330‐332.

Wansink B, Westgren R (2003). Profiling taste‐motivated segments. Appetite. 41(3): 323‐7.

Sigirci O, Rockmore M, Wansink B (2016). How Traumatic Violence Permanently Changes Shopping Behavior. *Front. Psychol.* 7:1298.

Sigirci O, Wansink B (2015). Low prices and high regret: how pricing influences regret at all‐you‐can‐eat buffets. BMC Nutrition 1:36.

Wansink B, Park S‐B (2002). Sensory Suggestiveness and Labeling: Do Soy Labels Bias Taste? Journal of Sensory Studies. 17(5): 483‐491.

Wansink B, Just DR, Payne CR (2012). Can Branding Improve School Lunches? Arch Pediatr Adolesc Med. 166(10): 967‐968.

## Appendices

### Appendix 1. Search strategies, search dates and yields

**Cochrane Central Register of Controlled Trials (CENTRAL) in *The Cochrane Library,* 1992 to 30 January 2015**

**Original search executed: 20 November 2012; Retrieved: 3192 records**

**Updated search executed: 30 January 2015; Retrieved 1269 records**

drink* OR drunk* OR alcohol* OR beverage* OR beer* OR lager* OR wine* OR cider* OR alcopop* OR alco‐pop* OR spirit OR spirits OR liquor* OR liquer* OR liqueur* OR whisky OR whiskey OR whiskies OR whiskeys OR schnapps OR brandy OR brandies OR gin OR gins OR rum OR rums OR tequila* OR vodka* OR cocktail* OR cigar* OR smoke OR smokes OR smoking OR smoker OR smokers OR smoked OR tobacco* OR nutri* OR calori* OR food* OR eat OR eats OR eaten OR eating OR ate OR meal* OR snack*

AND

siz* OR dimension* OR capacit* OR volume* OR shap* OR height* OR width* OR length* OR depth* OR divide*

AND

portion* OR serving* OR product* OR packag* OR packet* OR unit* OR tableware OR drinkware OR dinnerware OR crockery OR plate* OR platter* OR tureen* OR tajine* OR tagine* OR bowl* OR charger* OR cup* OR saucer* OR glass OR glasses OR mug OR mugs OR beaker* OR pitcher* OR jug* OR decanter* OR receptacle* OR container* OR dish* OR pot OR pots OR cutlery OR flatware OR utensil* OR knife OR *knife OR knives OR fork* OR spoon* OR *spoon OR tongs OR ladle* OR chopstick* OR box* OR bag* OR can* OR carton* OR bottle* OR straw*

NOT

rat OR rats OR mouse OR mice OR murine OR rodent OR rodents OR hamster OR hamsters OR pig OR pigs OR porcine OR rabbit OR rabbits OR animal OR animals OR dog OR dogs OR cat OR cats OR cow OR cows OR bovine OR sheep OR ovine OR monkey OR monkeys

**MEDLINE (OvidSP ‐ including MEDLINE In‐Process), 1946 to November Week 1 2012**

**Original search executed: 13 November 2012; Retrieved: 17,085 records**

**Updated search executed: 30 January 2015; Retrieved 4205 records**

1 exp Beverages/ 87429

2 exp Drinking Behavior/ 52972

3 exp Alcohol Drinking/ 47670

4 exp Food Industry/ 91946

5 exp Alcohol‐Related Disorders/ 92856

6 (drink$ or drunk$ or alcohol$ or beverage$1 or beer$1 or lager$1 or wine$1 or cider$1 or alcopop$1 or alco‐pop$1 or spirit or spirits or liquor$1 or liquer$1 or liqueur$1 or whisky or whiskey or whiskies or whiskeys or schnapps or brandy or brandies or gin or gins or rum or rums or tequila$1 or vodka$1 or cocktail$1).ti,ab. 286166

7 exp Tobacco/ 23931

8 exp Smoking/ 113243

9 exp "Tobacco Use Disorder"/ 7270

10 (cigar$ or smoke or smokes or smoking or smoker or smokers or smoked or tobacco$).ti,ab. 196390

11 exp Diet/ 178322

12 exp Food Industry/ 91946

13 exp Food/ 985939

14 exp Food Habits/ 18591

15 exp Food Preferences/ 8909

16 exp Eating/ 55571

17 exp Feeding Behavior/ 111521

18 exp Eating Disorders/ 20715

19 (nutri$ or calori$ or food$ or eat or eats or eaten or eating or ate or meal$ or snack$ or drink$ or drunk$ or beverage$1).ti,ab. 583819

20 exp Food Packaging/ 4321

21 exp Food Storage/ 249

22 exp Cooking/ and Eating Utensils/ 104

23 exp Product Packaging/ 15467

24 ((siz$ or dimension$ or capacit$ or volume$ or shap$ or height$ or width$ or length$ or depth$ or divide$) adj4 (portion$ or serving$ or product$ or packag$ or packet$ or unit$ or cigar$ or food$ or drink$ or alcohol$ or tableware or drinkware or dinnerware or crockery or plate$1 or platter$1 or tureen$1 or tajine$1 or tagine$1 or bowl$1 or charger$1 or cup$1 or saucer$1 or glass or glasses or mug or mugs or beaker$1 or pitcher$1 or jug$1 or decanter$1 or receptacle$1 or container$1 or dish$ or pot or pots or cutlery or flatware or utensil$1 or knife or $knife or knives or fork$1 or spoon$ or $spoon or tongs or ladle$1 or chopstick$1 or box$ or bag$ or can$ or carton$1 or bottle$ or straw$1)).ti,ab. 94119

25 or/1‐6 465421

26 or/7‐10 229371

27 or/11‐19 1554173

28 or/20‐24 109600

29 25 and 28 10916

30 26 and 28 2480

31 27 and 28 18704

32 or/29‐31 22530

33 animals/ 5087545

34 (rat or rats or mouse or mice or murine or rodent or rodents or hamster or hamsters or pig or pigs or porcine or rabbit or rabbits or animal or animals or dog or dogs or cat or cats or cow or cows or bovine or sheep or ovine or monkey or monkeys).ti,ab.

3089377

35 or/33‐34 5362242

36 humans/ and animals/ 1372372

37 35 not 36 3989870

38 32 not 37 17590

39 (editorial or case reports or in vitro).pt. 2288418

40 38 not 39 17085

**EMBASE (OvidSP), 1980 to 30 January 2015**

**Original search executed: 14 November 2012; Retrieved: 22,308 records**

**Updated search executed: 30 January 2015; Retrieved 6922 records**

1 exp beverage/ 121492

2 exp Drinking Behavior/ 32744

3 exp alcohol consumption/ 61917

4 exp food industry/ 18653

5 exp alcohol abuse/ 19149

6 (drink$ or drunk$ or alcohol$ or beverage$1 or beer$1 or lager$1 or wine$1 or cider$1 or alcopop$1 or alco‐pop$1 or spirit or spirits or liquor$1 or liquer$1 or liqueur$1 or whisky or whiskey or whiskies or whiskeys or schnapps or brandy or brandies or gin or gins or rum or rums or tequila$1 or vodka$1 or cocktail$1).ti,ab. 380427

7 exp tobacco/ 28053

8 exp smoking/ 154998

9 exp tobacco dependence/ 11151

10 (cigar$ or smoke or smokes or smoking or smoker or smokers or smoked or tobacco$).ti,ab. 247027

11 exp diet/ 174704

12 exp food industry/ 18653

13 exp food/ 566656

14 exp food habits/ 103715

15 exp food preferences/ 8309

16 exp eating/ 19350

17 exp feeding behavior/ 103715

18 exp eating disorder/ 32352

19 (nutri$ or calori$ or food$ or eat or eats or eaten or eating or ate or meal$ or snack$ or drink$ or drunk$ or beverage$1).ti,ab. 737112

20 exp food packaging/ 5102

21 exp food storage/ 3444

22 exp kitchen/ 1553

23 exp packaging/ 16183

24 ((siz$ or dimension$ or capacit$ or volume$ or shap$ or height$ or width$ or length$ or depth$ or divide$) adj4 (portion$ or serving$ or product$ or packag$ or packet$ or unit$ or cigar$ or food$ or drink$ or alcohol$ or tableware or drinkware or dinnerware or crockery or plate$1 or platter$1 or tureen$1 or tajine$1 or tagine$1 or bowl$1 or charger$1 or cup$1 or saucer$1 or glass or glasses or mug or mugs or beaker$1 or pitcher$1 or jug$1 or decanter$1 or receptacle$1 or container$1 or dish$ or pot or pots or cutlery or flatware or utensil$1 or knife or $knife or knives or fork$1 or spoon$ or $spoon or tongs or ladle$1 or chopstick$1 or box$ or bag$ or can$ or carton$1 or bottle$ or straw$1)).ti,ab. 120594

25 or/1‐6 494774

26 or/7‐10 290348

27 or/11‐19 1272638

28 or/20‐24 140907

29 25 and 28 9711

30 26 and 28 3061

31 27 and 28 22322

32 or/29‐31 27278

33 animals/ 1800693

34 (rat or rats or mouse or mice or murine or rodent or rodents or hamster or hamsters or pig or pigs or porcine or rabbit or rabbits or animal or animals or dog or dogs or cat or cats or cow or cows or bovine or sheep or ovine or monkey or monkeys).ti,ab.

3381652

35 or/33‐34 4408920

36 humans/ and animals/ 454714

37 35 not 36 3954206

38 32 not 37 22488

39 (editorial or case reports or in vitro).pt. 415728

40 38 not 39 22308

**PsycINFO (OvidSP), 1806 to 30 January 2015**

**Original search executed: 14 November 2012; Retrieved: 4099 records**

**Updated search executed: 30 January 2015; Retrieved 1079 records**

1 exp Alcoholic Beverage/ 1884

2 exp "Beverages (Nonalcoholic)"/ 772

3 exp Drinking Behavior/ 54223

4 exp Alcohol Drinking Patterns/ 49383

5 exp Alcohol Abuse/ 36125

6 (drink$ or drunk$ or alcohol$ or beverage$1 or beer$1 or lager$1 or wine$1 or cider$1 or alcopop$1 or alco‐pop$1 or spirit or spirits or liquor$1 or liquer$1 or liqueur$1 or whisky or whiskey or whiskies or whiskeys or schnapps or brandy or brandies or gin or gins or rum or rums or tequila$1 or vodka$1 or cocktail$1).ti,ab. 111663

7 exp Tobacco Smoking/ 20293

8 (cigar$ or smoke or smokes or smoking or smoker or smokers or smoked or tobacco$).ti,ab. 38912

9 exp diets/ 8007

10 exp eating behavior/ 11578

11 exp food/ 8002

12 exp food intake/ 11118

13 exp food preferences/ 3193

14 exp eating/ 11578

15 exp feeding behavior/ 8236

16 exp eating disorder/ 21015

17 (nutri$ or calori$ or food$ or eat or eats or eaten or eating or ate or meal$ or snack$ or drink$ or drunk$ or beverage$1).ti,ab. 123754

18 ((siz$ or dimension$ or capacit$ or volume$ or shap$ or height$ or width$ or length$ or depth$ or divide$) adj6 (portion$ or serving$ or product$ or packag$ or packet$ or unit$ or cigar$ or food$ or drink$ or alcohol$ or tableware or drinkware or dinnerware or crockery or plate$1 or platter$1 or tureen$1 or tajine$1 or tagine$1 or bowl$1 or charger$1 or cup$1 or saucer$1 or glass or glasses or mug or mugs or beaker$1 or pitcher$1 or jug$1 or decanter$1 or receptacle$1 or container$1 or dish$ or pot or pots or cutlery or flatware or utensil$1 or knife or $knife or knives or fork$1 or spoon$ or $spoon or tongs or ladle$1 or chopstick$1 or box$ or bag$ or can$ or carton$1 or bottle$ or straw$1)).ti,ab. 24137

19 or/1‐6 115188

20 or/7‐8 39235

21 or/9‐17 139533

22 18 and 19 3224

23 18 and 20 503

24 18 and 21 4019

25 or/22‐24 5627

26 limit 25 to human 4099

**Applied Social Sciences Index and Abstracts (ProQuest), 1987 to 30 January 2015**

**Original search executed: 20 November 2012; Retrieved: 949 records**

**Updated search executed: 30 January 2015; Retrieved 178 records**

all(drink* OR drunk* OR alcohol* OR beverage[*1] OR beer[*1] OR lager[*1] OR wine[*1] OR cider[*1] OR alcopop[*1] OR alco‐pop[*1] OR spirit OR spirits OR liquor[*1] OR liquer[*1] OR liqueur[*1] OR whisky OR whiskey OR whiskies OR whiskeys OR schnapps OR brandy OR brandies OR gin OR gins OR rum OR rums OR tequila[*1] OR vodka[*1] OR cocktail[*1] OR cigar* OR smoke OR smokes OR smoking OR smoker OR smokers OR smoked OR tobacco* OR nutri* OR calori* OR food* OR eat OR eats OR eaten OR eating OR ate OR meal* OR snack*)

AND

all((siz* OR dimension* OR capacit* OR volume* OR shap* OR height* OR width* OR length* OR depth* OR divide*) NEAR/6 (portion* OR serving* OR product* OR packag* OR packet* OR unit* OR cigar* OR food* OR drink* OR alcohol* OR tableware OR drinkware OR dinnerware OR crockery OR plate[*1] OR platter[*1] OR tureen[*1] OR tajine[*1] OR tagine[*1] OR bowl[*1] OR charger[*1] OR cup[*1] OR saucer[*1] OR glass OR glasses OR mug OR mugs OR beaker[*1] OR pitcher[*1] OR jug[*1] OR decanter[*1] OR receptacle[*1] OR container[*1] OR dish* OR pot OR pots OR cutlery OR flatware OR utensil[*1] OR knife OR *knife OR knives OR fork[*1] OR spoon* OR *spoon OR tongs OR ladle[*1] OR chopstick[*1] OR box* OR bag* OR can* OR carton[*1] OR bottle* OR straw[*1]))

NOT

all(rat OR rats OR mouse OR mice OR murine OR rodent OR rodents OR hamster OR hamsters OR pig OR pigs OR porcine OR rabbit OR rabbits OR animal OR animals OR dog OR dogs OR cat OR cats OR cow OR cows OR bovine OR sheep OR ovine OR monkey OR monkeys)

**Food Science and Technology Abstracts (Web of Knowledge), 1969 to 22 November 2012**

**Original search executed: 20 November 2012; Retrieved: 6437 records**

Topic=(drink* OR drunk* OR alcohol* OR beverage* OR beer* OR lager* OR wine* OR cider* OR alcopop* OR alco‐pop* OR spirit OR spirits OR liquor* OR liquer* OR liqueur* OR whisky OR whiskey OR whiskies OR whiskeys OR schnapps OR brandy OR brandies OR gin OR gins OR rum OR rums OR tequila* OR vodka* OR cocktail* OR cigar* OR smoke OR smokes OR smoking OR smoker OR smokers OR smoked OR tobacco* OR nutri* OR calori* OR food* OR eat OR eats OR eaten OR eating OR ate OR meal* OR snack*) AND Topic=((siz* OR dimension* OR capacit* OR volume* OR shap* OR height* OR width* OR length* OR depth* OR divide*) NEAR/6 (portion* OR serving* OR product* OR packag* OR packet* OR unit* OR cigar* OR food* OR drink* OR alcohol* OR tableware OR drinkware OR dinnerware OR crockery OR plate* OR platter* OR tureen* OR tajine* OR tagine* OR bowl* OR charger* OR cup* OR saucer* OR glass OR glasses OR mug OR mugs OR beaker* OR pitcher* OR jug* OR decanter* OR receptacle* OR container* OR dish* OR pot OR pots OR cutlery OR flatware OR utensil* OR knife OR *knife OR knives OR fork* OR spoon* OR *spoon OR tongs OR ladle* OR chopstick* OR box* OR bag* OR can* OR carton* OR bottle* OR straw*)) NOT Topic=(rat or rats or mouse or mice or murine or rodent or rodents or hamster or hamsters or pig or pigs or porcine or rabbit or rabbits or animal or animals or dog or dogs or cat or cats or cow or cows or bovine or sheep or ovine or monkey or monkeys)

Refined by: [excluding] Document Types=( PATENT OR REVIEW OR LEGISLATION OR BOOK ) AND [excluding] Research Areas=( PHYSICS OR BIOTECHNOLOGY APPLIED MICROBIOLOGY OR CHEMISTRY OR TOXICOLOGY ) AND [excluding] Descriptors=( FREEZING OR OXIDATION OR DRYING OR FOOD FACTORIES OR TEMP OR PHENOLS OR MOISTURE CONTENT OR STARCH OR ANTIOXIDATIVE ACTIVITY OR ANALYTICAL TECHNIQUES OR DISEASES OR STERILIZATION OR MODELLING OR TEMPERATURE OR PARTICLES OR MICROORGANISMS OR FLAVOUR OR PROCESSING THERMAL OR FOOD SAFETY OR EXTRUSION OR HEATING )

**We also ran a supplementary search for the FSTA index term ‘portion sizes’. Executed: 20 November 2012; Retrieved: 72 records**

Descriptors=(portion sizes)

Refined by: [excluding] Document Types=( REVIEW ) AND [excluding] FSTA Section=( PATENTS )

**Web of Knowledge (Science Citation Index Expanded, 1900 to 30 January 2015 Social Sciences Citation Index, 1956 to 30 January 2015; Conference Proceedings Citation Index ‐ Science, 1990 to 30 January 2015; Conference Proceedings Citation Index ‐ Social Science & Humanities, 1990 to 30 January 2015)**

**Original search executed: 20 November 2012; Retrieved: 5298 records**

**Updated search executed: 30 January 2015; Retrieved 2194 records**

Topic=(drink* OR drunk* OR alcohol* OR beverage* OR beer* OR lager* OR wine* OR cider* OR alcopop* OR alco‐pop* OR spirit OR spirits OR liquor* OR liquer* OR liqueur* OR whisky OR whiskey OR whiskies OR whiskeys OR schnapps OR brandy OR brandies OR gin OR gins OR rum OR rums OR tequila* OR vodka* OR cocktail* OR cigar* OR smoke OR smokes OR smoking OR smoker OR smokers OR smoked OR tobacco* OR nutri* OR calori* OR food* OR eat OR eats OR eaten OR eating OR ate OR meal* OR snack*) AND Topic=((siz* OR dimension* OR capacit* OR volume* OR shap* OR height* OR width* OR length* OR depth* OR divide*) NEAR/6 (portion* OR serving* OR product* OR packag* OR packet* OR unit* OR cigar* OR food* OR drink* OR alcohol* OR tableware OR drinkware OR dinnerware OR crockery OR plate* OR platter* OR tureen* OR tajine* OR tagine* OR bowl* OR charger* OR cup* OR saucer* OR glass OR glasses OR mug OR mugs OR beaker* OR pitcher* OR jug* OR decanter* OR receptacle* OR container* OR dish* OR pot OR pots OR cutlery OR flatware OR utensil* OR knife OR *knife OR knives OR fork* OR spoon* OR *spoon OR tongs OR ladle* OR chopstick* OR box* OR bag* OR can* OR carton* OR bottle* OR straw*)) NOT Topic=(rat OR rats OR mouse OR mice OR murine OR rodent OR rodents OR hamster OR hamsters OR pig OR pigs OR porcine OR rabbit OR rabbits OR animal OR animals OR dog OR dogs OR cat OR cats OR cow OR cows OR bovine OR sheep OR ovine OR monkey OR monkeys)

Refined by: [excluding] Web of Science Categories=( ECOLOGY OR ENTOMOLOGY OR CLINICAL NEUROLOGY OR ORNITHOLOGY OR MATERIALS SCIENCE CERAMICS OR MARINE FRESHWATER BIOLOGY OR SOIL SCIENCE OR PEDIATRICS OR CHEMISTRY PHYSICAL OR EVOLUTIONARY BIOLOGY OR AGRICULTURAL ENGINEERING OR ENERGY FUELS OR DENTISTRY ORAL SURGERY MEDICINE OR ENVIRONMENTAL SCIENCES OR LIMNOLOGY OR CELL BIOLOGY OR PHYSICS ATOMIC MOLECULAR CHEMICAL OR BIOPHYSICS OR ENGINEERING CHEMICAL OR ENGINEERING ELECTRICAL ELECTRONIC OR PHYSICS MULTIDISCIPLINARY OR MATERIALS SCIENCE MULTIDISCIPLINARY OR SURGERY OR MECHANICS OR OCEANOGRAPHY OR FORESTRY OR CARDIAC CARDIOVASCULAR SYSTEMS OR GASTROENTEROLOGY HEPATOLOGY OR PERIPHERAL VASCULAR DISEASE OR ZOOLOGY OR GEOSCIENCES MULTIDISCIPLINARY OR METEOROLOGY ATMOSPHERIC SCIENCES OR BIOTECHNOLOGY APPLIED MICROBIOLOGY OR PHYSICS CONDENSED MATTER OR CHEMISTRY INORGANIC NUCLEAR OR POLYMER SCIENCE OR ELECTROCHEMISTRY OR FISHERIES OR TOXICOLOGY OR CHEMISTRY MULTIDISCIPLINARY OR NEUROSCIENCES OR VETERINARY SCIENCES OR PLANT SCIENCES OR PSYCHOLOGY CLINICAL OR SPORT SCIENCES OR CHEMISTRY APPLIED OR GENETICS HEREDITY OR ENGINEERING CIVIL OR CHEMISTRY ANALYTICAL OR BIOCHEMISTRY MOLECULAR BIOLOGY OR THERMODYNAMICS OR COMPUTER SCIENCE INTERDISCIPLINARY APPLICATIONS OR PSYCHIATRY OR OPTICS OR ENGINEERING BIOMEDICAL OR AGRONOMY OR AGRICULTURE DAIRY ANIMAL SCIENCE OR BUSINESS OR ONCOLOGY OR BIOCHEMICAL RESEARCH METHODS OR PHARMACOLOGY PHARMACY OR NANOSCIENCE NANOTECHNOLOGY OR ANTHROPOLOGY OR AGRICULTURE MULTIDISCIPLINARY OR METALLURGY METALLURGICAL ENGINEERING OR MANAGEMENT OR WATER RESOURCES OR ECONOMICS OR SPECTROSCOPY OR PHYSIOLOGY OR NUCLEAR SCIENCE TECHNOLOGY OR MICROBIOLOGY OR RESPIRATORY SYSTEM OR CRITICAL CARE MEDICINE OR BIOLOGY OR INSTRUMENTS INSTRUMENTATION OR AGRICULTURAL ECONOMICS POLICY OR ENGINEERING ENVIRONMENTAL OR RADIOLOGY NUCLEAR MEDICINE MEDICAL IMAGING OR CRYSTALLOGRAPHY OR BIODIVERSITY CONSERVATION OR ENGINEERING MANUFACTURING OR HORTICULTURE OR ENGINEERING MECHANICAL OR OPERATIONS RESEARCH MANAGEMENT SCIENCE OR PHYSICS APPLIED OR CHEMISTRY ORGANIC OR IMMUNOLOGY OR ENDOCRINOLOGY METABOLISM ) AND [excluding] Web of Science Categories=( EDUCATION EDUCATIONAL RESEARCH OR MEDICAL INFORMATICS OR WOMEN S STUDIES OR ASTRONOMY ASTROPHYSICS OR COMMUNICATION OR STATISTICS PROBABILITY OR COMPUTER SCIENCE INFORMATION SYSTEMS OR COMPUTER SCIENCE THEORY METHODS OR CRIMINOLOGY PENOLOGY OR ENVIRONMENTAL STUDIES OR MATHEMATICAL COMPUTATIONAL BIOLOGY OR HEMATOLOGY OR TROPICAL MEDICINE OR PHYSICS MATHEMATICAL OR VIROLOGY OR GERONTOLOGY OR CHEMISTRY MEDICINAL OR MEDICINE LEGAL OR PSYCHOLOGY DEVELOPMENTAL OR UROLOGY NEPHROLOGY OR SOCIAL ISSUES OR IMAGING SCIENCE PHOTOGRAPHIC TECHNOLOGY OR OBSTETRICS GYNECOLOGY OR TRANSPORTATION OR LAW OR GEOCHEMISTRY GEOPHYSICS OR DERMATOLOGY OR MINERALOGY OR PHYSICS FLUIDS PLASMAS OR PHYSICS NUCLEAR OR GERIATRICS GERONTOLOGY OR ERGONOMICS OR SOCIAL SCIENCES MATHEMATICAL METHODS OR OPHTHALMOLOGY OR HOSPITALITY LEISURE SPORT TOURISM OR NURSING OR SOCIAL WORK OR FAMILY STUDIES OR EDUCATION SCIENTIFIC DISCIPLINES OR ANESTHESIOLOGY OR EMERGENCY MEDICINE OR MATERIALS SCIENCE PAPER WOOD OR GEOLOGY OR INFORMATION SCIENCE LIBRARY SCIENCE OR PARASITOLOGY OR POLITICAL SCIENCE OR PALEONTOLOGY OR MATHEMATICS INTERDISCIPLINARY APPLICATIONS OR ORTHOPEDICS OR RHEUMATOLOGY OR SOCIOLOGY OR REHABILITATION OR DEMOGRAPHY OR REPRODUCTIVE BIOLOGY OR MICROSCOPY OR ANATOMY MORPHOLOGY OR TELECOMMUNICATIONS OR OTORHINOLARYNGOLOGY OR ENGINEERING INDUSTRIAL OR AUTOMATION CONTROL SYSTEMS OR PHYSICS PARTICLES FIELDS OR MATHEMATICS OR DEVELOPMENTAL BIOLOGY OR PATHOLOGY OR ENGINEERING MULTIDISCIPLINARY OR INTEGRATIVE COMPLEMENTARY MEDICINE OR INFECTIOUS DISEASES OR PRIMARY HEALTH CARE OR ROBOTICS OR MATHEMATICS APPLIED OR MATERIALS SCIENCE TEXTILES OR URBAN STUDIES OR GEOGRAPHY OR MYCOLOGY OR INTERNATIONAL RELATIONS OR MEDICAL LABORATORY TECHNOLOGY OR COMPUTER SCIENCE SOFTWARE ENGINEERING OR MINING MINERAL PROCESSING OR COMPUTER SCIENCE ARTIFICIAL INTELLIGENCE OR MATERIALS SCIENCE COMPOSITES OR REMOTE SENSING OR PLANNING DEVELOPMENT ) AND [excluding] Web of Science Categories=( ACOUSTICS OR ENGINEERING MARINE OR MATERIALS SCIENCE CHARACTERIZATION TESTING OR ETHICS OR HISTORY OR HUMANITIES MULTIDISCIPLINARY OR INDUSTRIAL RELATIONS LABOR OR PSYCHOLOGY EDUCATIONAL OR MATERIALS SCIENCE BIOMATERIALS OR ALLERGY OR MEDICAL ETHICS OR MATERIALS SCIENCE COATINGS FILMS OR PHILOSOPHY OR CONSTRUCTION BUILDING TECHNOLOGY OR PSYCHOLOGY MATHEMATICAL OR AREA STUDIES OR PUBLIC ADMINISTRATION OR AUDIOLOGY SPEECH LANGUAGE PATHOLOGY OR TRANSPLANTATION OR COMPUTER SCIENCE HARDWARE ARCHITECTURE OR TRANSPORTATION SCIENCE TECHNOLOGY OR ENGINEERING GEOLOGICAL OR BUSINESS FINANCE OR ENGINEERING PETROLEUM OR CULTURAL STUDIES OR ETHNIC STUDIES OR ENGINEERING OCEAN OR GEOGRAPHY PHYSICAL OR HISTORY OF SOCIAL SCIENCES OR RELIGION OR HISTORY PHILOSOPHY OF SCIENCE OR ANDROLOGY OR MUSIC OR ENGINEERING AEROSPACE OR ARCHAEOLOGY OR NEUROIMAGING )

**Trials Register of Promoting Health Interventions (EPPI Centre), 2004 to 30 January 2015**

**Original search executed: 23 November 2012; Retrieved: 477 records**

**Updated search executed: 30 January 2015; Retrieved 167 records**

110 Focus of the report: alcohol OR healthy eating OR tobacco

111 Type(s) of intervention: environmental modification

112 110 AND 111

113 Freetext (item record) "unit*"

114 Freetext (item record) "portion*"

115 Freetext (item record) "serving*"

116 Freetext (item record) "product*"

117 Freetext (item record) "packag*"

118 Freetext (item record) "packet*"

119 Freetext (item record) "tableware"

120 Freetext (item record) "drinkware"

121 Freetext (item record) "dinnerware"

122 Freetext (item record) "crockery"

123 Freetext (item record) "plate*"

124 Freetext (item record) "platter*"

125 Freetext (item record) "tureen*"

126 Freetext (item record) "tajine*"

127 Freetext (item record) "tagine*"

128 Freetext (item record) "bowl*"

129 Freetext (item record) "charger*"

130 Freetext (item record) "cup*"

131 Freetext (item record) "saucer*"

132 Freetext (item record) "glass"

133 Freetext (item record) "glasses"

134 Freetext (item record) "mug"

135 Freetext (item record) "mugs"

136 Freetext (item record) "beaker*"

137 Freetext (item record) "pitcher*"

138 Freetext (item record) "jug*"

139 Freetext (item record) "decanter*"

140 Freetext (item record) "receptacle*"

141 Freetext (item record) "container*"

142 Freetext (item record) "dish*"

143 Freetext (item record) "pot"

144 Freetext (item record) "pots"

145 Freetext (item record) "cutlery"

146 Freetext (item record) "flatware"

147 Freetext (item record) "utensil*"

148 Freetext (item record) "knife"

149 Freetext (item record) "*knife"

150 Freetext (item record) "knives"

151 Freetext (item record) "fork"

152 Freetext (item record) "fork*"

153 Freetext (item record) "spoon*"

154 Freetext (item record) "*spoon"

155 Freetext (item record) "tongs"

156 Freetext (item record) "ladle*"

157 Freetext (item record) "chopstick*"

158 Freetext (item record) "box*"

159 Freetext (item record) "bag*"

160 Freetext (item record) "cans"

161 Freetext (item record) "carton*"

162 Freetext (item record) "bottle*"

163 Freetext (item record) "straw*"

164 113 OR 114 OR 115 OR 116 OR 117 OR 118 OR 119 OR 120 OR 121 OR 122 OR 123 OR 124 OR 125 OR 126 OR 127 OR 128 OR 129 OR 130 OR 131 OR 132 OR 133 OR 134 OR 135 OR 136 OR 137 OR 138 OR 139 OR 140 OR 141 OR 142 OR 143 OR 144 OR 145 OR 146 OR 147 OR 148 OR 149 OR 150 OR 151 OR 152 OR 153 OR 154 OR 155 OR 156 OR 157 OR 158 OR 159 OR 160 OR 161 OR 162 OR 163

165 Freetext (item record) "drink*"

166 Freetext (item record) "drunk*"

167 Freetext (item record) "alcohol*"

168 Freetext (item record) "beverage*"

169 Freetext (item record) "beer*"

170 Freetext (item record) "lager*"

171 Freetext (item record) "wine*"

172 Freetext (item record) "cider*"

173 Freetext (item record) "alcopop*"

174 Freetext (item record) "alco‐pop*"

175 Freetext (item record) "spirit"

176 Freetext (item record) "spirits"

177 Freetext (item record) "liquor*"

178 Freetext (item record) "liquer*"

179 Freetext (item record) "liqueur*"

180 Freetext (item record) "whisk*"

181 Freetext (item record) "schnapps"

182 Freetext (item record) "brandy"

183 Freetext (item record) "brandies"

184 Freetext (item record) "gin"

185 Freetext (item record) "gins"

186 Freetext (item record) "rum"

187 Freetext (item record) "rums"

188 Freetext (item record) "tequila*"

189 Freetext (item record) "vodka*"

190 Freetext (item record) "cocktail*"

191 Freetext (item record) "cigar*"

192 Freetext (item record) "smoke"

193 Freetext (item record) "smokes"

194 Freetext (item record) "smoking"

195 Freetext (item record) "smoker"

196 Freetext (item record) "smokers"

197 Freetext (item record) "smoked"

198 Freetext (item record) "tobacco*"

199 Freetext (item record) "nutri*"

200 Freetext (item record) "calori*"

201 Freetext (item record) "food*"

202 Freetext (item record) "eat"

203 Freetext (item record) "eats"

204 Freetext (item record) "eaten"

205 Freetext (item record) "eating"

206 Freetext (item record) "ate"

207 Freetext (item record) "meal"

208 Freetext (item record) "meal*"

209 Freetext (item record) "snack*"

210 165 OR 166 OR 167 OR 168 OR 169 OR 170 OR 171 OR 172 OR 173 OR 174 OR 175 OR 176 OR 177 OR 178 OR 179 OR 180 OR 181 OR 182 OR 183 OR 184 OR 185 OR 186 OR 187 OR 188 OR 189 OR 190 OR 191 OR 192 OR 193 OR 194 OR 195 OR 196 OR 197 OR 198 OR 199 OR 200 OR 201 OR 202 OR 203 OR 204 OR 205 OR 206 OR 207 OR 208 OR 209

211 164 AND 210

212 112 OR 211

213 114 OR 115 OR 116 OR 117 OR 118 OR 119 OR 120 OR 121 OR 122 OR 123 OR 124 OR 125 OR 126 OR 127 OR 128 OR 129 OR 130 OR 131 OR 132 OR 133 OR 134 OR 135 OR 136 OR 137 OR 138 OR 139 OR 140 OR 141 OR 142 OR 143 OR 144 OR 145 OR 146 OR 147 OR 148 OR 149 OR 150 OR 151 OR 152 OR 153 OR 154 OR 155 OR 156 OR 157 OR 158 OR 159 OR 160 OR 161 OR 162 OR 163

**Open Grey (www.opengrey.eu), 1980 to 30 January 2015**

**Search executed: 30 January 2015; Retrieved 367 records**

(drink* OR drunk* OR alcohol* OR beverage* OR beer* OR lager* OR wine* OR cider* OR alcopop* OR alco‐pop* OR spirit OR spirits OR liquor* OR liquer* OR liqueur* OR whisky OR whiskey OR whiskies OR whiskeys OR schnapps OR brandy OR brandies OR gin OR gins OR rum OR rums OR tequila* OR vodka* OR cocktail* OR cigar* OR smoke OR smokes OR smoking OR smoker OR smokers OR smoked OR tobacco* OR nutri* OR calori* OR food* OR eat OR eats OR eaten OR eating OR ate OR meal* OR snack*) AND ((siz* OR dimension* OR capacit* OR volume* OR shap* OR height* OR width* OR length* OR depth* OR divide*) NEAR/6 (portion* OR serving* OR product* OR packag* OR packet* OR unit* OR cigar* OR food* OR drink* OR alcohol* OR tableware OR drinkware OR dinnerware OR crockery OR plate* OR platter* OR tureen* OR tajine* OR tagine* OR bowl* OR charger* OR cup* OR saucer* OR glass OR glasses OR mug OR mugs OR beaker* OR pitcher* OR jug* OR decanter* OR receptacle* OR container* OR dish* OR pot OR pots OR cutlery OR flatware OR utensil* OR knife OR *knife OR knives OR fork* OR spoon* OR *spoon OR tongs OR ladle* OR chopstick* OR box* OR bag* OR can* OR carton* OR bottle* OR straw*))

### Appendix 2. Preliminary analyses of minimum data extracted from 11 eligible studies identified by the updated search

**Introduction**

The updated search conducted up to 30 January 2015 identified 11 further eligible studies published during 2013 and 2014 (see also Search methods for identification of studies, Results of the search and Appendix 1). Key characteristics of each of these 11 eligible studies (Bajaj 2014; Haire 2014; Kral 2014; Marchiori 2014; Rolls 2014a; Smith 2013a; van Ittersum 2013; van Kleef 2014; Wansink 2013; Wansink 2014; Williams 2014) are described in Characteristics of studies awaiting classification (the information in Characteristics of studies awaiting classification is based on the minimum data set that we provisionally extracted from the 12 corresponding study reports ‐ see below in this section).

All 11 further eligible studies have been accepted into the review and currently await full integration, which is scheduled for the first major update. At that stage we will: collect the maximum data set for each study (comprising > 1000 variables) from the 12 corresponding study reports (including supplementary coding based on external data sources and contacts with study authors to request data that are not available in study reports); conduct 'Risk of bias' assessments; update meta‐analyses; update meta‐regression analyses; update GRADE assessments; and make corollary updates to the Results, Discussion and Authors' conclusions sections of the review, including 'Summary of findings' tables (see also Data collection and analysis).

However, in advance of their full integration into this review, it was important to establish whether the pending full integration of these 11 eligible studies has any potential to change the interpretation of the results of this review, and hence its conclusions, as these are currently reported in the Results, Discussion and Authors' conclusions. These sections are currently based *exclusively* on evidence collected from the 72 included studies identified by the original search and published between 1978 and July 2013 (see also Search methods for identification of studies, Results of the search and Figure 2).

We therefore conducted preliminary statistical analyses to investigate this issue based on outcome data that could provisionally be extracted from each of the 11 further eligible studies (i.e. in advance of contacting study authors, with one exception ‐ see 'Potential impact of studies with no useable data', below).

**Procedure**

We provisionally extracted useable outcome data with respect to each eligible independent within‐study comparison identified in these 11 studies (Bajaj 2014; Haire 2014; Kral 2014; Marchiori 2014; Rolls 2014a; Smith 2013a; van Ittersum 2013; van Kleef 2014; Wansink 2013; Wansink 2014; Williams 2014). We then provisionally computed study‐level effect sizes for each eligible independent within‐study comparison as the standardised difference in means (SMD) and its standard error, with respect to consumption and selection outcomes (as applicable). We then integrated provisional study‐level effect sizes that could be computed from these 11 studies with those previously computed from 70 of 72 studies included studies identified by the original search, using random‐effects meta‐analysis (i.e. we applied the same procedures described in Data collection and analysis to provisionally update meta‐analyses). Finally, we assessed the potential for full integration of these 11 studies to change current quality of evidence ratings with respect to provisionally updated estimates of summary effect sizes using the GRADE system (see Data synthesis).

**Results**

We identified a total of 17 eligible independent within‐study comparisons (i.e. measurement of at least one of our specified outcomes) in the 11 further eligible studies (Bajaj 2014; Haire 2014; Kral 2014; Marchiori 2014; Rolls 2014a; Smith 2013a; van Ittersum 2013; van Kleef 2014; Wansink 2013; Wansink 2014; Williams 2014):

- 16 comparisons assessed the effect of larger versus smaller‐sized portions, packages or tableware on **consumption** of food; and
- six comparisons assessed the effect of larger versus smaller‐sized portions, packages or tableware on **selection** of food.

This established that full integration of these 11 studies could only influence the results of two meta‐analyses (and related findings), which investigated:

- the effect of exposure to larger versus smaller‐sized portions, packages or tableware on quantities of food **consumed** (Table 1); and
- the effect of exposure to larger versus smaller‐sized portions, packages or tableware on quantities of food **selected** (see Table 1).

Table A2.1 shows effect sizes provisionally computed for each eligible independent within‐study comparison identified in the 11 studies used in these preliminary analyses. For the consumption outcome, we extracted useable data with respect to 14 of 16 independent comparisons (nine of 11 studies). No useable consumption outcome data could be extracted from van Ittersum 2013. This was a paired study and the corresponding study report does not provide sufficient information (notably, the correlation coefficient) to enable estimation of the correct standard deviation or SMD based on reported F‐statistics. In addition, no useable consumption outcome data could be extracted from Wansink 2013 due to unclear reporting of results from the relevant intention‐to‐treat (ITT) analysis. For the selection outcome, we extracted useable data with respect to four of six independent comparisons (four of six studies). No useable selection outcome data could be extracted from van Ittersum 2013 or Wansink 2013 for the same reasons given above.

**Table A2.1 Study‐level effect sizes**

**Table:**

	**Consumption**			**Selection**		
**Comparison**	**SMD (95% CI)**	**SE**	**Interpretation**	**SMD (95% CI)**	**SE**	**Interpretation**
Bajaj 2014	0.23 (0.01 to 0.45)	0.11	Larger size increased consumption	Not measured	‐	‐
Haire 2014	0.23 (‐0.26 to 0.72)	0.25	No difference	Not measured	‐	‐
Kral 2014 [1]	0.43 (‐0.05 to 0.91)	0.25	No difference	Not measured		
Kral 2014 [2]	‐0.02 (‐0.50 to 0.46)	0.24	No difference	Not measured	‐	‐
Marchiori 2014	0.81 (0.42 to 1.20)	0.20	Larger size increased consumption	Not measured	‐	‐
Rolls 2014a [1]	‐0.32 (‐0.85 to 0.21)	0.27	No difference	‐0.35 (‐0.88 to 0.18)	0.27	No difference
Rolls 2014a [2]	‐0.35 (‐0.97 to 0.27)	0.32	No difference	‐0.36 (‐0.98 to 0.26)	0.32	No difference
Rolls 2014a [3]	‐0.15 (‐0.68 to 0.38)	0.27	No difference	‐0.32 (‐0.86 to 0.22)	0.28	No difference
Smith 2013a [1]	‐0.96 (‐1.33 to ‐0.59)	0.19	Larger size reduced consumption	Not measured	‐	‐
Smith 2013a [2]	1.04 (0.67 to 1.41)	0.19	Larger size increased consumption	Not measured	‐	‐
Smith 2013a [3]	0.67 (0.27 to 1.07)	0.20	Larger size increased consumption	Not measured	‐	‐
Smith 2013a [4]	0.61 (0.22 to 1.00)	0.20	Larger size increased consumption	Not measured	‐	‐
van Ittersum 2013	No useable data	‐	‐	No useable data	‐	‐
van Kleef 2014	0.48 (0.17 to 0.79)	0.16	Larger size increased consumption	Not measured	‐	‐
Wansink 2013	No useable data	‐	‐	No useable data	‐	‐
Wansink 2014	Not measured	‐	‐	1.41 (0.88 to 1.94)	0.27	Larger size increased selection
Williams 2014	0.46 (0.05 to 0.87)	0.21	Larger size increased consumption	Not measured	‐	‐

The first row of Table A2.2 (below) reproduces the result of the meta‐analysis that we conducted to investigate (1) the effect of exposure to larger versus smaller‐sized portions, packages or tableware on quantities of food *consumed* (see also Table 1)*.* This meta‐analysis was based on outcome data from a total of 6603 participants (86 independent comparisons). The second row of Table A2.2 shows the *provisional* result from a *preliminary* meta‐analysis that integrates outcome data from an additional 1591 participants (15 independent comparisons); a combined total N of 9785 participants (101 independent comparisons).

**Table A2.2. Effect of exposure to larger versus smaller‐sized portions, packages or tableware on quantities of food consumed**

**Table:**

**Independent comparisons (N)**	**Total participants (N)**	**SMD**	**95% CI lower bound**	**95% CI upper bound**	**I^2^**
86	6603	0.38	0.29	0.46	61%
100	9748	0.35	0.27	0.44	68%

The first row of Table A2.3 reproduces the result of the meta‐analysis that was conducted to investigate (2) the effect of exposure to larger versus smaller‐sized portions, packages or tableware on quantities of food *selected* (see also Table 1). This meta‐analysis was based on outcome data from a total of 1164 participants (13 independent comparisons). The second row of Table A2.3 shows the *provisional* result from a *preliminary* meta‐analysis that integrates outcome data from an additional 194 participants (four independent comparisons); a combined total N of 1358 participants (17 independent comparisons).

**Table A2.3. Effect of exposure to larger versus smaller‐sized portions, packages or tableware on quantities of food selected**

**Table:**

**Independent comparisons (N)**	**Total participants (N)**	**SMD**	**95% CI lower bound**	**95% CI upper bound**	**I^2^**
13	1164	0.42	0.24	0.59	54%
17	1358	0.36	0.15	0.57	73%

As shown in Tables A2.2 and A2.3, point estimates and 95% confidence intervals from random‐effects models are similar between the current and provisionally updated results of these meta‐analyses. Critically, provisionally updated results remain consistent with the current findings of this review (see Discussion and Authors' conclusions) that exposure to larger versus smaller‐sized portions, packages or tableware increased both quantities of food consumed and quantities of food selected for consumption, and that the sizes of these effects were small to moderate in relative terms.

Table A2.4 summarises the results of our quality of evidence ratings with respect to current and provisionally updated estimates of the summary effect size for (1) the effect of exposure to larger versus smaller sized portions, packages or tableware on quantities of food *consumed*.

**Table A2.4 Review of quality of evidence ratings: consumption**

**Table:**

	**Independent comparisons (N)**	**Total participants (N)**	**Risk of bias**	**Inconsistency**	**Indirectness**	**Imprecision**	**Other considerations**	**Overall quality rating**
Current	86	6603	Serious limitations	Not serious	Not serious	Not serious	None	Moderate
Provisionally updated	100	9748	Serious limitations	Not serious	Not serious	Not serious	None	Moderate

With respect to **risk of bias**, we already rated current evidence (86 independent comparisons) down by one level (i.e. serious limitations) due to all study‐level estimates of this effect having been judged to be at 'unclear or high risk of bias'. Therefore, even in the extreme hypothetical scenarios that all further eligible studies are in due course judged to be either at 'low' or 'unclear' or 'high' risk of bias with respect to their study‐level estimates of this effect, integration of these assessments (with respect to 16 further independent comparisons) cannot change the current rating (i.e. serious limitations).

With respect to **inconsistency**, we did not rate down current evidence (86 independent comparisons) based on our judgement that large inconsistency (heterogeneity) in study results did not remain after exploration of a priori hypotheses that might explain heterogeneity (i.e. potential effect modifiers) using meta‐regression analysis (see Data synthesis). Whilst the full integration of data concerning potential effect modifiers yet to be collected from further eligible studies (independent comparisons) into updated meta‐regression analyses will inevitably influence the detailed results of those analyses, we judge that the likelihood of the current rating (i.e. 'Not serious') could change as a consequence is minimal.

With respect to **indirectness**, we did not rate down current evidence (86 independent comparisons) based on our judgement that all included studies (within‐study comparisons) assessed interventions, comparators and outcomes that met eligibility criteria for this review in participant samples that also met eligibility criteria, and were all direct head‐to‐head comparisons. As such, there were no differences between the populations, interventions or outcomes measured among included studies and those under consideration in the current review. The same is also true of the 10 of 11 further eligible studies accepted into the review and currently awaiting full integration that measured the consumption outcome (see Characteristics of studies awaiting classification). Therefore, full integration of these further eligible studies cannot change the current rating (i.e. 'Not serious').

With respect to **imprecision**, we did not rate down current evidence (86 independent comparisons) based on examination of the upper and lower bounds of 95% confidence intervals associated with the estimated summary effect size, coupled with the consideration that the number of participants (effective sample size) incorporated into this meta‐analysis exceeded the number of participants generated by a conventional sample size calculation for a single adequately powered trial (optimal information size). Since full integration of further eligible studies will increase the number of participants (effective sample size) incorporated into an updated version of this meta‐analysis, this cannot change the current rating (i.e. 'Not serious').

With respect to **other considerations**, we judged that there were 'None' associated with current evidence (86 independent comparisons) on the basis that none of the primary reasons suggested by the GRADE system for rating up quality of evidence (Guyatt 2011) were applicable in this case. Based on provisional results of the relevant preliminary analysis reported above (see Table A2.2), we judge the likelihood that the current rating (i.e. 'None') could change as a consequence of full integration of data from 10 of 11 further eligible studies that measured the consumption outcome is minimal.

In summary, our review of quality of evidence ratings establishes that full integration of 10 further eligible studies accepted into the review and currently awaiting full integration that measured the consumption outcome cannot change the **overall quality of evidence** rating with respect to the provisionally updated estimate of the summary effect size for (1) the effect of exposure to larger versus smaller‐sized portions, packages or tableware on quantities of food *consumed*.

Table A2.5 summarises the results of our quality of evidence ratings with respect to current and provisionally updated estimates of the summary effect size for (2) the effect of exposure to larger versus smaller‐sized portions, packages or tableware on quantities of food *selected*.

**Table A2.5 Review of quality of evidence ratings: selection**

**Table:**

	**Independent comparisons (N)**	**Total participants (N)**	**Risk of bias**	**Inconsistency**	**Indirectness**	**Imprecision**	**Other considerations**	**Overall quality rating**
Current	13	1164	Serious limitations	Not serious	Not serious	Not serious	None	Moderate
Provisionally updated	17	1358	Serious limitations	Not serious	Not serious	Not serious	None	Moderate

Identical considerations to those described above in the case of the effect on consumption apply here with respect to ratings of risk of bias, inconsistency, indirectness, imprecision and other considerations that collectively determine confidence in estimates of the effect of exposure to larger versus smaller size on food *selection*. In summary, this review of quality of evidence ratings establishes that full integration of six further eligible studies accepted into the review and currently awaiting full integration that measured the selection outcome cannot change the **overall quality of evidence** rating with respect to the provisionally updated estimate of the summary effect size for the effect of exposure to larger versus smaller‐sized portions, packages or tableware on quantities of food *selected*.

**Potential impact of studies with no useable data**

As stated above no useable data could be extracted from the Wansink 2013 study with respect to either the consumption or the selection outcome due to unclear reporting of results from the relevant intention‐to‐treat (ITT) analysis. As noted in Characteristics of studies awaiting classification the Wansink 2013 study was a between‐subjects cluster‐randomised controlled trial that included investigation of the effects of 'exposure to whole apples available for purchase in the school lunchroom' (larger individual unit size), versus 'exposure to apples sliced into six symmetric pieces available for purchase in the school lunchroom' (smaller individual unit size). The study randomised six middle schools (clusters) comprising a total of 2150 participants (students) to these two comparison groups: 'whole apple schools' (larger individual unit size) and 'sliced apple schools' (smaller individual unit size).

Outcomes in this study included measures of both selection and consumption that are eligible for inclusion in meta‐analyses (1) and (2) respectively. The selection outcome appears to have been measured as the numbers of students who purchased (and did not purchase) an apple on study days in 'whole apple schools' and 'sliced apple schools' respectively. Based on these data it would in principle be possible to construct a 2 x 2 table in order to compute a log odds ratio and its standard error, which could then be converted into a useable SMD and its SE using the formula provided in Section 9.4.6 of the *Cochrane Handbook for Systematic Reviews of Interventions* (Deeks 2011). However, in order to apply this procedure we would first need confirmation from study authors of the following data, which are currently unclear in the corresponding study report (Wansink 2013): the numbers of participants in schools randomised to each comparison group (i.e. 'whole apple schools' and 'sliced apple schools'); and the numbers of participants who purchased and did not purchase an apple on study days in 'whole apple schools' and 'sliced apple schools' respectively. The consumption outcome appears to have been measured as the amount of apple consumed in grams per student on study days in 'whole apple schools' and 'sliced apple schools' respectively. However, in order to compute a SMD and its standard error based on these data, we need both the standard deviations and denominators (i.e. numbers of participants in 'whole apple schools' and 'sliced apple schools') associated with reported mean gram amounts of consumption in 'whole apple schools' and 'sliced apple schools' respectively. These numerical data are (respectively) not reported and ambiguous in the corresponding study report (in the latter case it is also unclear whether or not the denominators reflect the randomised allocation).

Since Wansink 2013 was a large study (with an effective sample size of 4300 participants), we sought these numerical results by contacting the corresponding author, but *to date of publication of this review* we have received a response but not the necessary data. This is consistent with previous contacts with the author that we initiated to request numerical results that are missing from, or unclear in, published reports of several of their other 11 studies already included in this review (Wansink 1996a (S1); Wansink 1996b (S2); Wansink 1996c (S4); Wansink 2001; Wansink 2003 (S1); Wansink 2003 (S2); Wansink 2005b; Wansink 2005d; Wansink 2006; Wansink 2011a (S4); Wansink 2011b). Whilst we have received responses to our previous contacts, the author was unable or unwilling to provide the requested data. As such, no useable outcome data have to date been collected from the Wansink 2013 that could be incorporated into the preliminary analyses presented above.

Therefore, whilst the potential impact of integrating data from Wansink 2013 into further updated meta‐analyses of (1) and (2) the effects of exposure to larger versus smaller‐sized portions, packages or tableware on quantities of food *consumed* and *selected* may be substantive, this cannot currently be established with any confidence and we judge the likelihood of obtaining useable data from the study authors to be low. To illustrate, with respect to the selection outcome, if we assumed that: (a) there were equal numbers of participants in schools randomised to each comparison group, (b) the denominator reported in Wansink 2013, Table 1, Row 1 ("*n=*334") was the 'total number of apples purchased' on study days in 'whole apple schools' and 'sliced apple schools' combined; and (c) the figures 6% and 10% in Wansink 2013 Table 1, Row 1 reflect the *relative* numbers of apples purchased on study days in 'whole apple schools' and 'sliced apple schools' respectively – then it would be possible to estimate a SMD and its standard error using the procedure described above as SMD ‐0.31 (SE 0.0647226) (were the latter estimate integrated into meta‐analysis (2), the summary effect size would be SMD 0.01 (95% CI ‐0.01 to 0.16)). However, it is important to highlight assumptions (a), (b) and (c) have not been verified and are likely to be incorrect, and moreover that this estimate of the study level SMD and its standard error are sensitive to variation in these assumptions. With respect to the consumption outcome, it was not judged credible to make assumptions needed to enable provisional estimation of a SMD and its standard error, due to the level of ambiguity in the reporting of these outcome data and the lack of scope for imputing data from similar studies in this specific case. On the latter point, Wansink 2013 has distinctive characteristics that differentiate it from the other studies included and accepted for inclusion in this review. For example, this is the only eligible study identified to date which included a measure of the effect on purchasing (i.e. selection with purchase) and that this is the only cluster‐randomised trial identified to date that includes a measure of selection (with or without purchase). Based on these considerations, we may propose to produce further updates of meta‐analyses (1) and (2) for the first major update of this review both *without* outcome data from Wansink 2013 (primary analyses) and *with* outcome data from Wansink 2013 (sensitivity analysis), subject to being able to obtain useable data from the study authors.

The second study with no useable data was van Ittersum 2013. Since this was a small study (effective sample size of 36), we judge that full integration of outcome data from this study into meta‐analyses (1) and (2) will have no substantive impact on current estimates of summary effect sizes.

**Conclusions**

The results of the preliminary analyses reported here in Appendix 2 (see also Characteristics of studies awaiting classification) establish that there is minimal potential for full integration 11 further eligible studies identified by the updated search to change the interpretation of the results of this review, and hence its conclusions, as these are currently reported in the Results, Discussion and Authors' conclusions. This conclusion is based on the following key findings:

- Interpretation of the result of an updated meta‐analysis of (1) the effect of exposure to larger versus smaller‐sized portions, packages or tableware on quantities of food *consumed* will not change: there will still be overall moderate quality evidence that larger portion, package and tableware size increased consumption of food, with a small to moderate effect size.
- Interpretation of the result of an updated meta‐analysis of (2) the effect of exposure to larger versus smaller sized portions, packages or tableware on quantities of food *selected* will not change: there will still be overall moderate quality evidence that larger portion, package and tableware size increased selection of food, with a small to moderate effect size.
- Overall quality of evidence ratings cannot change with respect to updated summary estimates of (1) and (2) the effects of exposure to larger versus smaller sized portions, packages or tableware on quantities of food *consumed* and *selected*.

Finally (as described above), we plan to fully integrate these 11 further eligible studies (Bajaj 2014; Haire 2014; Kral 2014; Marchiori 2014; Rolls 2014a; Smith 2013a; van Ittersum 2013; van Kleef 2014; Wansink 2013; Wansink 2014; Williams 2014) into this review as part of the process of conducting its first major update.

### Appendix 3. Full results of meta‐regression analyses conducted to investigate modifiers of the effect of larger size on consumption

**Table:**

**Variable name**	**num**	**incl_excl**	**coef**	**coef1**	**coef2**	**coef3**	**coef4**	**coef5**
Sel_Pur	4	Only one category	NA	NA	NA	NA	NA	NA
Prod_Type	92	Not significant	NA	‐0.13[‐0.65,0.38]	NA	NA	NA	NA
Soc_Setting	92	Not significant	NA	‐0.30[‐0.64,0.05]	‐0.14 [‐0.50,0.21]	‐0.30 [‐0.97,0.37]	NA	NA
FSA_Meth	57	Not significant	0.02 [‐0.21,0.24]	NA	NA	NA	NA	NA
FSA_Score	57	Included	0.01 [0.00,0.02]	NA	NA	NA	NA	NA
En_Density	57	Included	0.04 [‐0.00,0.08]	NA	NA	NA	NA	NA
Manip_Target	92	Not significant	NA	0.21 [‐0.22,0.64]	‐0.11 [‐0.62,0.40]	0.04 [‐0.33,0.40]	‐0.04 [‐0.46,0.37]	NA
Manip_Type	92	Only one category	NA	NA	NA	NA	NA	NA
Dur_Exposure	92	Not significant	0.23 [‐0.02,0.48]	NA	NA	NA	NA	NA
Conc_Int	92	Not significant	‐0.22 [‐0.54,0.09]	NA	NA	NA	NA	NA
SES_Context	92	Not significant	NA	0.15[‐0.27,0.57]	NA	NA	NA	NA
F_O_1	73	Included	0.22 [0.02,0.41]	NA	NA	NA	NA	NA
F_O_2	73	Not significant	‐0.12 [‐0.38,0.15]	NA	NA	NA	NA	NA
F_O_3	73	Not significant	‐0.13 [‐0.32,0.05]	NA	NA	NA	NA	NA
F_O_4	86	Included	0.32 [0.16,0.48]	NA	NA	NA	NA	NA
Size_Abs	52	Not significant	0.00 [‐0.00,0.00]	NA	NA	NA	NA	NA
Size_Rel	80	Not significant	‐0.00 [‐0.00,0.00]	NA	NA	NA	NA	NA
Age_Mean	74	Included	0.01 [‐0.00,0.02]	NA	NA	NA	NA	NA
Female_Percent	86	Not significant	0.00 [‐0.00,0.01]	NA	NA	NA	NA	NA
Eth_White_Percent	21	Not significant	0.00 [‐0.00,0.00]	NA	NA	NA	NA	NA
BMI_Mean	52	Not significant	‐0.01 [‐0.05,0.04]	NA	NA	NA	NA	NA
BMIz_Mean	5	Insufficient data	NA	NA	NA	NA	NA	NA
BodFat_Mean	2	Insufficient data	NA	NA	NA	NA	NA	NA
Weight_Mean	41	Not significant	0.00 [‐0.00,0.01]	NA	NA	NA	NA	NA
Overweight_Percent	19	Not significant	0.00 [‐0.01,0.01]	NA	NA	NA	NA	NA
Obese_Percent	10	Not significant	0.01 [‐0.02,0.05]	NA	NA	NA	NA	NA
Overweight_Obese_Percent	6	Insufficient data	NA	NA	NA	NA	NA	NA
Restraint_1_Mean	32	Not significant	0.01 [‐0.09,0.10]	NA	NA	NA	NA	NA
Restraint_2_Mean	4	Insufficient data	NA	NA	NA	NA	NA	NA
Restraint_3_Mean	3	Insufficient data	NA	NA	NA	NA	NA	NA
Disinhib_1_Mean	29	Not significant	‐0.05 [‐0.27,0.17]	NA	NA	NA	NA	NA
Disinhib_2_Mean	1	Insufficient data	NA	NA	NA	NA	NA	NA
ExEat_Mean	4	Insufficient data	NA	NA	NA	NA	NA	NA
EmEat_Mean	3	Insufficient data	NA	NA	NA	NA	NA	NA
PClean_Mean	2	Insufficient data	NA	NA	NA	NA	NA	NA
PClean_Ad_Percent	3	Insufficient data	NA	NA	NA	NA	NA	NA
PClean_Ch_Percent	3	Insufficient data	NA	NA	NA	NA	NA	NA
ConsMon_Mean	2	Insufficient data	NA	NA	NA	NA	NA	NA
Binge_1_Mean	2	Insufficient data	NA	NA	NA	NA	NA	NA
Binge_2_Mean	1	Insufficient data	NA	NA	NA	NA	NA	NA
Diet_Mean	14	Not significant	‐0.07[‐0.15,0.01]	NA	NA	NA	NA	NA
Mood_Mean	2	Insufficient data	NA	NA	NA	NA	NA	NA
EnInt_Mean	2	Insufficient data	NA	NA	NA	NA	NA	NA
Carb_Mean	1	Insufficient data	NA	NA	NA	NA	NA	NA
Prot_Mean	1	Insufficient data	NA	NA	NA	NA	NA	NA
Fat_Mean	1	Insufficient data	NA	NA	NA	NA	NA	NA
Step_Mean	1	Insufficient data	NA	NA	NA	NA	NA	NA
EnExp_Mean	16	Not significant	‐0.00[‐0.00,0.00]	NA	NA	NA	NA	NA
Exerc_Mean	1	Insufficient data	NA	NA	NA	NA	NA	NA
Hunger_1_Mean	29	Not significant	‐0.13[‐0.33,0.07]	NA	NA	NA	NA	NA
Hunger_2_Mean	8	Insufficient data	NA	NA	NA	NA	NA	NA
Hunger_3_Mean	1	Insufficient data	NA	NA	NA	NA	NA	NA
Hunger_4_Mean	1	Insufficient data	NA	NA	NA	NA	NA	NA
Fullness_Mean	1	Insufficient data	NA	NA	NA	NA	NA	NA
Sat_Mean	1	Insufficient data	NA	NA	NA	NA	NA	NA
ProsCon_Mean	1	Insufficient data	NA	NA	NA	NA	NA	NA
Depress_Mean	12	Not significant	‐0.22[‐0.50,0.07]	NA	NA	NA	NA	NA
Employ_Percent	2	Insufficient data	NA	NA	NA	NA	NA	NA
Par_Employ_Percent	7	Insufficient data	NA	NA	NA	NA	NA	NA
EduYears_Mean	1	Insufficient data	NA	NA	NA	NA	NA	NA
EduHigh_Percent	2	Insufficient data	NA	NA	NA	NA	NA	NA
Par_EduHigh_Percent	8	Insufficient data	NA	NA	NA	NA	NA	NA
Par_EduDeg_Percent	5	Insufficient data	NA	NA	NA	NA	NA	NA
Inc50_Percent	1	Insufficient data	NA	NA	NA	NA	NA	NA
FamInc50_Percent	5	Insufficient data	NA	NA	NA	NA	NA	NA
Insec_Percent	3	Insufficient data	NA	NA	NA	NA	NA	NA
NSLP_Percent	1	Insufficient data	NA	NA	NA	NA	NA	NA
SNAP_Percent	0	Insufficient data	NA	NA	NA	NA	NA	NA
ROBSum_Sel	92	Not significant	NA	‐0.10[‐0.47,0.27]	NA	NA	NA	NA
ROBSum_Con	92	Not significant	NA	‐0.24[‐0.61,0.13]	NA	NA	NA	NA
design1	92	Not significant	‐0.14 [‐0.38,0.09]	NA	NA	NA	NA	NA
design2	92	Included	‐0.40 [‐0.55,‐0.25]	NA	NA	NA	NA	NA
design3	92	Not significant	0.07 [‐0.13,0.26]	NA	NA	NA	NA	NA

### Appendix 4. Full results of meta‐regression analyses conducted to investigate modifiers of the effect of larger size on selection

**Table:**

**Variable name**	**num**	**incl_excl**	**coef**	**coef1**	**coef2**	**coef3**	**coef4**	**coef5**
Sel_Pur	13	Only one category	NA	NA	NA	NA	NA	NA
Prod_Type	13	Only one category	NA	NA	NA	NA	NA	NA
Soc_Setting	13	Not significant	NA	0.15 [‐0.27,0.58]	NA	NA	NA	NA
FSA_Meth	11	Not significant	‐0.49 [‐1.14,0.16]	NA	NA	NA	NA	NA
FSA_Score	11	Not significant	‐0.01 [‐0.06,0.04]	NA	NA	NA	NA	NA
En_Density	11	Not significant	‐0.02 [‐0.23,0.19]	NA	NA	NA	NA	NA
Manip_Target	13	Not significant	NA	0.22 [‐0.63,1.07]	0.21 [‐0.25,0.68]	NA	NA	NA
Manip_Type	13	Only one category	NA	NA	NA	NA	NA	NA
Dur_Exposure	13	Not significant	‐0.51 [‐1.33,0.31]	NA	NA	NA	NA	NA
Conc_Int	13	Not significant	‐0.22 [‐1.03,0.60]	NA	NA	NA	NA	NA
SES_Context	13	Not significant	NA	0.22 [‐0.60,1.03]	NA	NA	NA	NA
F_O_1	7	Insufficient data	NA	NA	NA	NA	NA	NA
F_O_2	7	Insufficient data	NA	NA	NA	NA	NA	NA
F_O_3	7	Insufficient data	NA	NA	NA	NA	NA	NA
F_O_4	13	Included	0.41 [0.06,0.76]	NA	NA	NA	NA	NA
Size_Abs	4	Insufficient data	NA	NA	NA	NA	NA	NA
Size_Rel	11	Not significant	‐0.00 [‐0.02,0.01]	NA	NA	NA	NA	NA
Age_Mean	6	Insufficient data	NA	NA	NA	NA	NA	NA
Female_Percent	13	Not significant	0.00 [‐0.01,0.01]	NA	NA	NA	NA	NA
Eth_White_Percent	4	Insufficient data	NA	NA	NA	NA	NA	NA
BMI_Mean	2	Insufficient data	NA	NA	NA	NA	NA	NA
BMIz_Mean	2	Insufficient data	NA	NA	NA	NA	NA	NA
BodFat_Mean	0	Insufficient data	NA	NA	NA	NA	NA	NA
Weight_Mean	0	Insufficient data	NA	NA	NA	NA	NA	NA
Overweight_Percent	0	Insufficient data	NA	NA	NA	NA	NA	NA
Obese_Percent	0	Insufficient data	NA	NA	NA	NA	NA	NA
Overweight_Obese_Percent	1	Insufficient data	NA	NA	NA	NA	NA	NA
Restraint_1_Mean	0	Insufficient data	NA	NA	NA	NA	NA	NA
Restraint_2_Mean	0	Insufficient data	NA	NA	NA	NA	NA	NA
Restraint_3_Mean	1	Insufficient data	NA	NA	NA	NA	NA	NA
Disinhib_1_Mean	0	Insufficient data	NA	NA	NA	NA	NA	NA
Disinhib_2_Mean	0	Insufficient data	NA	NA	NA	NA	NA	NA
ExEat_Mean	0	Insufficient data	NA	NA	NA	NA	NA	NA
EmEat_Mean	0	Insufficient data	NA	NA	NA	NA	NA	NA
PClean_Mean	0	Insufficient data	NA	NA	NA	NA	NA	NA
PClean_Ad_Percent	0	Insufficient data	NA	NA	NA	NA	NA	NA
PClean_Ch_Percent	0	Insufficient data	NA	NA	NA	NA	NA	NA
ConsMon_Mean	0	Insufficient data	NA	NA	NA	NA	NA	NA
Binge_1_Mean	0	Insufficient data	NA	NA	NA	NA	NA	NA
Binge_2_Mean	0	Insufficient data	NA	NA	NA	NA	NA	NA
Diet_Mean	0	Insufficient data	NA	NA	NA	NA	NA	NA
Mood_Mean	0	Insufficient data	NA	NA	NA	NA	NA	NA
EnInt_Mean	0	Insufficient data	NA	NA	NA	NA	NA	NA
Carb_Mean	0	Insufficient data	NA	NA	NA	NA	NA	NA
Prot_Mean	0	Insufficient data	NA	NA	NA	NA	NA	NA
Fat_Mean	0	Insufficient data	NA	NA	NA	NA	NA	NA
Step_Mean	0	Insufficient data	NA	NA	NA	NA	NA	NA
EnExp_Mean	0	Insufficient data	NA	NA	NA	NA	NA	NA
Exerc_Mean	0	Insufficient data	NA	NA	NA	NA	NA	NA
Hunger_1_Mean	0	Insufficient data	NA	NA	NA	NA	NA	NA
Hunger_2_Mean	0	Insufficient data	NA	NA	NA	NA	NA	NA
Hunger_3_Mean	0	Insufficient data	NA	NA	NA	NA	NA	NA
Hunger_4_Mean	0	Insufficient data	NA	NA	NA	NA	NA	NA
Fullness_Mean	0	Insufficient data	NA	NA	NA	NA	NA	NA
Sat_Mean	0	Insufficient data	NA	NA	NA	NA	NA	NA
ProsCon_Mean	0	Insufficient data	NA	NA	NA	NA	NA	NA
Depress_Mean	0	Insufficient data	NA	NA	NA	NA	NA	NA
Employ_Percent	0	Insufficient data	NA	NA	NA	NA	NA	NA
Par_Employ_Percent	4	Insufficient data	NA	NA	NA	NA	NA	NA
EduYears_Mean	0	Insufficient data	NA	NA	NA	NA	NA	NA
EduHigh_Percent	0	Insufficient data	NA	NA	NA	NA	NA	NA
Par_EduHigh_Percent	4	Insufficient data	NA	NA	NA	NA	NA	NA
Par_EduDeg_Percent	0	Insufficient data	NA	NA	NA	NA	NA	NA
Inc50_Percent	1	Insufficient data	NA	NA	NA	NA	NA	NA
FamInc50_Percent	0	Insufficient data	NA	NA	NA	NA	NA	NA
Insec_Percent	1	Insufficient data	NA	NA	NA	NA	NA	NA
NSLP_Percent	1	Insufficient data	NA	NA	NA	NA	NA	NA
SNAP_Percent	2	Insufficient data	NA	NA	NA	NA	NA	NA
ROBSum_Sel	13	Not significant	NA	0.02 [‐0.45,0.49]	NA	NA	NA	NA
ROBSum_Con	13	Not significant	NA	0.15 [‐0.27,0.58]	NA	NA	NA	NA
design1	13	Not significant	‐0.32 [‐0.76,0.12]	NA	NA	NA	NA	NA
design2	13	Included	‐0.41 [‐0.76,‐0.06]	NA	NA	NA	NA	NA
design3	13	Not significant	0.08 [‐0.39,0.56]	NA	NA	NA	NA	NA

## Characteristics of studies

### Characteristics of included studies [ordered by study ID]

**Table:** Ahn 2010

Methods	Study design: between‐subjects randomised controlled trial	
Participants	Setting: field setting, hospital diabetes outpatient clinic Geographical region: Eulji, South Korea Number of enrolled participants: 42 adults Number (%) of enrolled participants completing the study: 42 (100%) Study completers ‐ mean age (SD): 55.2 (7.1) Study completers ‐ sex: female only Study completers ‐ mean BMI kg/m^2^ (SD): 27.8 (4.0) Specific social or cultural characteristics: none Socio‐economic status context: low deprivation Inclusion criteria: female; aged between 20 and 70 years; diagnosed with type 2 diabetes mellitus according to the diagnostic standards established by the American Diabetes Association in 1997; BMI ≥ 23 kg/m2; HbA1c levels between 6.0% and 10.0% Exclusion criteria: current treatment with insulin or thiazolidinedione medications; consumes > 1 alcoholic beverage per day; eats away from home more than twice per week; special diet (e.g. vegetarian); unable to exercise; indigestion; anorexia; gestational diabetes; malignant tumour(s); cardiovascular disease; consumed body weight loss drugs in the last 3 months; difficult to follow; refused investigation	
Interventions	Manipulated product type: food Manipulation: tableware size (rice bowl) Duration of exposure to intervention: > 1 day Social setting: consuming alone and with others Study arms: small size rice bowl (200 mL bowl) with 5 to 10 minutes individual diet education, an information leaflet corresponding to prescribed energy intake and a pedometer; regular size rice bowl (380 mL bowl) with 5 to 10 minutes individual diet education, an information leaflet corresponding to prescribed energy intake and a pedometer; dietary education based on the diabetic dietary guideline of the Korean Diabetes Association Number of comparisons analysed: 1 Comparisons analysed: Intervention 1: small size rice bowl (200 mL bowl); *versus* Intervention 2: regular size rice bowl (380 mL bowl) Concurrent intervention components: yes. 5 to 10 minutes individual diet education, an information leaflet corresponding to prescribed energy intake and a pedometer ‐ provided to both Intervention 1 and Intervention 2 groups	
Outcomes	Outcomes reported in study: change in total daily energy intake (kcal); change in daily carbohydrate intake (grams); change in daily protein intake (grams); change in daily fat intake (grams); change in daily fibre intake (grams); change in daily cholesterol intake (mg); change in daily sodium intake (mg); change in daily carbohydrate intake, % of energy intake (%); change in daily protein intake, % of energy intake (%); change in daily fat intake, % of energy intake (%) Selection outcome analysed: N/A Measurement of selection outcome: N/A Timing of selection outcome measurement: N/A Consumption outcome analysed: total daily energy intake (kcal) Measurement of consumption outcome: self report Timing of consumption outcome measurement: longer‐term (> 1 day)	
Funding source	Not reported	
Notes	—	
***Risk of bias***		
**Bias**	**Authors' judgement**	**Support for judgement**
Random sequence generation (selection bias)	Low risk	Quote: "After the subjects enrolled, they were divided into small rice bowl group, regular rice bowl group, or control group, with the random number table."
Allocation concealment (selection bias)	High risk	Quote: "After the subjects enrolled, they were divided into small rice bowl group, regular rice bowl group, or control group, with the random number table." Comment: explicitly unconcealed procedure and investigators enrolling participants could possibly foresee assignments and thus introduce risk of selection bias
Blinding of participants and personnel (performance bias) Consumption outcome	High risk	Quote: "[Participants] were informed about the purpose and procedures involved in this study and all agreed to participate." Comment: no blinding of study participants nor study personnel and it is possible that the outcome may be influenced by lack of blinding of study participants
Blinding of outcome assessment (detection bias) Consumption outcome	High risk	Quote: "To determine food energy intake and nutrient intake, the rice bowl groups kept dietary records 3 days per week (2 weekdays and 1 weekend day) and reported to us a minimum of once every two weeks." Comment: no blinding of outcome assessment and it is possible that the outcome measurement may be influenced by lack of blinding
Incomplete outcome data (attrition bias) Consumption outcome	Low risk	Comment: no missing outcome data for consumption outcome
Selective reporting (reporting bias)	Unclear risk	Comment: search for record(s) containing details of study protocol conducted in ClinicalTrials.gov and the WHO International Clinical Trials Registry Platform (ICTRP). No records found. Insufficient information to permit judgement of 'low risk' or 'high risk'
Other bias #1 ‐ Baseline comparability of participant characteristics between groups	Low risk	Comment: study uses a between‐subjects design. No differences between comparison groups in terms of measured baseline participant characteristics
Other bias #2 ‐ Consistency in intervention delivery	Low risk	Quote: "...the subjects [in both the small rice bowl group and the large rice bowl group] were supplied with leaflet corresponding to prescribed energy and were educated on tips for putting rice into the bowl and taking side dishes, within 5‐10 minutes individual education... They were asked to use the bowl for every meal and carbohydrate sources such as bread, rice cake, potato, sweet potato were limited through the leaflet. Noodle could substitute for rice but any specific amount for that was not suggested. Fruit intake was shown as the amount per day through the leaflet. For fish, meat and vegetables, the subjects were educated with pictures of diet fitting each food exchanges unit and were asked to practice it but that was not emphasized intensively at each visit. The picture of diet of fish, meat and vegetables were included in the leaflet by focusing on foods frequently found in the preliminary survey... To assess compliance of use of rice bowl, the subjects were asked to record whether they used the provided bowls during breakfast, lunch, or dinner. During biweekly visits, subjects were instructed to bring their compliance reports and rice bowl usage compliance was calculated as a percentage. During each visit, the reported values were averaged and overall compliance was calculated as: compliance of use of rice bowl (%) = frequency of using bowls/number of total meals × 100...Between the small and regular rice bowl groups, there was no significant difference in frequency of usage." Comment: information and instructions to participants appear to have been standardised between the compared study conditions. Participants' compliance with the protocol for rice bowl usage was monitored and study authors state there was no difference between comparison groups in level of compliance.
Summary of risk of bias Consumption outcome	High risk	High risk

**Table:** Argo 2012 (S1)

Methods	Study design: between‐subjects randomised controlled trial	
Participants	Setting: laboratory setting Geographical region: Canada Number of enrolled participants: 76 female undergraduate students Number (%) of enrolled participants completing the study: 76 (100%) Study completers ‐ mean age (SD): not reported Study completers ‐ sex: female only Study completers ‐ mean BMI kg/m^2^ (SD): not reported (neither BMI nor other body weight or body weight status) Specific social or cultural characteristics: undergraduate students Socio‐economic status context: low deprivation Inclusion criteria: female; undergraduate student Exclusion criteria: none reported	
Interventions	Manipulated product type: food Manipulation: package size (gumdrops) Duration of exposure to intervention: ≤ 1 day Social setting: consuming alone Study arms: small‐package‐present (bowl containing 5 small, opaque packages each containing 4 gumdrops), low appearance self esteem; small‐package‐present (bowl containing 5 small, opaque packages each containing 4 gumdrops), high appearance self esteem; small‐package‐absent (bowl containing 20 loose, unpackaged gumdrops), low appearance self esteem; small‐package‐absent (bowl containing 20 loose, unpackaged gumdrops), high appearance self esteem Number of comparisons analysed: 1 Comparisons analysed: intervention 1: small‐package‐present (bowl containing 5 small, opaque packages each containing 4 gumdrops); *versus* Intervention 2: small‐package‐absent (bowl containing 20 loose, unpackaged gumdrops) Concurrent intervention components: no	
Outcomes	Outcomes reported in study: amount of gumdrops consumed (grams) Selection outcome analysed: N/A Measurement of selection outcome: N/A Timing of selection outcome measurement: N/A Consumption outcome analysed: amount of gumdrops consumed (grams) Measurement of consumption outcome: objective Timing of consumption outcome measurement: immediate (≤ 1 day)	
Funding source	Social Sciences and Humanities Research Council of Canada	
Notes	Outcome data for low appearance self esteem and high appearance self esteem participant subgroups collapsed and analysed together (one comparison)	
***Risk of bias***		
**Bias**	**Authors' judgement**	**Support for judgement**
Random sequence generation (selection bias)	Unclear risk	Comment: method of sequence generation is not described. Insufficient information about the sequence generation process to permit judgement of 'low risk' or 'high risk'
Allocation concealment (selection bias)	Unclear risk	Comment: method of concealment is not described. Insufficient information to permit judgement of 'low risk' or 'high risk'
Blinding of participants and personnel (performance bias) Consumption outcome	Low risk	Quote: "Participants completed the experiment individually and were seated in a cubicle facing away from a female experimenter. Each participant was told that we were interested in evaluations of a variety of products and that they would be asked to sample one of the products while completing a questionnaire... Finally, participants completed an open‐ended suspicion probe assessing what they thought was the purpose of the research. Responses indicated that participants were not cognizant of the hypotheses in this or any of the other studies." Comment: blinding of study participants attempted and unlikely that the blinding could have been broken. Very unlikely that key study personnel were blinded, but the review authors judge that the outcome is not likely to be influenced by lack of blinding of key study personnel
Blinding of outcome assessment (detection bias) Consumption outcome	Low risk	Comment: no blinding of outcome assessment, but the review authors judge that the outcome measurement is not likely to be influenced by lack of blinding
Incomplete outcome data (attrition bias) Consumption outcome	Low risk	Comment: no missing outcome data for consumption outcome
Selective reporting (reporting bias)	Unclear risk	Comment: search for record(s) containing details of study protocol conducted in ClinicalTrials.gov and the WHO International Clinical Trials Registry Platform (ICTRP). No records found. Insufficient information to permit judgement of 'low risk' or 'high risk'
Other bias #1 ‐ Baseline comparability of participant characteristics between groups	Unclear risk	Comment: study uses a between‐subjects design. Insufficient information to permit judgement of 'low risk' or 'high risk'
Other bias #2 ‐ Consistency in intervention delivery	Low risk	Quote: "Each participant was told that we were interested in evaluations of a variety of products and that they would be asked to sample one of the products while completing a questionnaire...Participants completed the experiment individually and were seated in a cubicle facing away from a female experimenter." Comment: information provided to participants appears to have been standardised between the compared study conditions. No specific instructions were provided to participants and therefore monitoring of participants' compliance with instructions is not applicable
Summary of risk of bias Consumption outcome	Unclear risk	Unclear risk

**Table:** Argo 2012 (S2)

Methods	Study design: between‐subjects randomised controlled trial	
Participants	Setting: laboratory setting Geographical region: Canada Number of enrolled participants: 207 undergraduate students Number (%) of enrolled participants completing the study: 207 (100%) Study completers ‐ mean age (SD): not reported Study completers ‐ sex: male (61%) and female (59%) Study completers ‐ mean BMI kg/m^2^ (SD): not reported (neither BMI nor other body weight or body weight status) Specific social or cultural characteristics: undergraduate students Socio‐economic status context: low deprivation Inclusion criteria: undergraduate student Exclusion criteria: none reported	
Interventions	Manipulated product type: food Manipulation: package size (candy‐coated chocolates) Duration of exposure to intervention: ≤1 day Social setting: consuming alone Study arms: small‐packages, product visible (8 x small transparent packages ‐ not reported how many chocolates in each package), low appearance self esteem; small‐packages, product visible (8 x small transparent packages ‐ not reported how many chocolates in each package), high appearance self esteem; small‐packages, product not visible (8 x small opaque packages ‐ not reported how many chocolates in each package), low appearance self esteem; small‐packages, product not visible (8 x small opaque packages ‐ not reported how many chocolates in each package), high appearance self esteem; large‐packages, product visible, (2 x large transparent packages ‐ not reported how many chocolates in each package), low appearance self esteem; large‐packages, product visible (2 x large transparent packages ‐ not reported how many chocolates in each package), high appearance self esteem; large‐packages, product not visible (2 x large opaque packages ‐ not reported how many chocolates in each package), low appearance self esteem; large‐packages, product not visible (2 x large opaque packages ‐ not reported how many chocolates in each package), high appearance self esteem. Number of comparisons analysed: 1 Comparisons analysed: Intervention 1:‐ small‐packages (8 x small transparent or opaque packages ‐ not reported how many chocolates in each package); *versus* Intervention 2:‐ large‐packages (2 x large transparent or opaque packages ‐ not reported how many chocolates in each package) Concurrent intervention components: no	
Outcomes	Outcomes reported in study: amount of candy‐coated chocolates consumed (grams) Selection outcome analysed: N/A Measurement of selection outcome: N/A Timing of selection outcome measurement: N/A Consumption outcome analysed: amount of candy‐coated chocolates consumed (grams) Measurement of consumption outcome: objective Timing of consumption outcome measurement: immediate (≤ 1 day)	
Funding source	Social Sciences and Humanities Research Council of Canada	
Notes	Outcome data for transparent and opaque package and low appearance self esteem and high appearance self esteem participant subgroups collapsed and analysed together (one comparison)	
***Risk of bias***		
**Bias**	**Authors' judgement**	**Support for judgement**
Random sequence generation (selection bias)	Unclear risk	Comment: method of sequence generation is not described. Insufficient information about the sequence generation process to permit judgement of 'low risk' or 'high risk'
Allocation concealment (selection bias)	Unclear risk	Comment: method of concealment is not described. Insufficient information to permit judgement of 'low risk' or 'high risk'
Blinding of participants and personnel (performance bias) Consumption outcome	Low risk	Quote: "Participants completed the experiment individually and were seated in a cubicle facing away from a female experimenter. Each participant was told that we were interested in evaluations of a variety of products and that they would be asked to sample one of the products while completing a questionnaire... Finally, participants completed an open‐ended suspicion probe assessing what they thought was the purpose of the research. Responses indicated that participants were not cognizant of the hypotheses in this or any of the other studies." Comment: blinding of study participants attempted and unlikely that the blinding could have been broken. Very unlikely that key study personnel were blinded, but the review authors judge that the outcome is not likely to be influenced by lack of blinding of key study personnel
Blinding of outcome assessment (detection bias) Consumption outcome	Low risk	Comment: no blinding of outcome assessment, but the review authors judge that the outcome measurement is not likely to be influenced by lack of blinding
Incomplete outcome data (attrition bias) Consumption outcome	Low risk	Comment: no missing outcome data for consumption outcome
Selective reporting (reporting bias)	Unclear risk	Comment: search for record(s) containing details of study protocol conducted in ClinicalTrials.gov and the WHO International Clinical Trials Registry Platform (ICTRP). No records found. Insufficient information to permit judgement of 'low risk' or 'high risk'
Other bias #1 ‐ Baseline comparability of participant characteristics between groups	Unclear risk	Comment: study uses a between‐subjects design. Insufficient information to permit judgement of 'low risk' or 'high risk'
Other bias #2 ‐ Consistency in intervention delivery	Low risk	Quote: "We used a procedure similar to that described in Study 1 [S2], with the following modifications. First, we measured ASE in an earlier session, and later we linked ASE scores to participants’ responses in the focal session. In addition, we extend the generalizability of our previous findings in two ways. First, we examine a different type of product (candy‐coated chocolates). Second, instead of using a package‐absent control, we used a large‐package control condition." Comment: information provided to participants appears to have been standardised between the compared study conditions. No specific instructions were provided to participants and therefore monitoring of participants' compliance with instructions is not applicable
Summary of risk of bias Consumption outcome	Unclear risk	Unclear risk

**Table:** Argo 2012 (S4)

Methods	Study design: between‐subjects randomised controlled trial	
Participants	Setting: laboratory setting Geographical region: Canada Number of enrolled participants: 297 female undergraduate students Number (%) of enrolled participants completing the study: 297 (100%) Study completers ‐ mean age (SD): not reported Study completers ‐ sex: female only Study completers ‐ mean BMI kg/m^2^ (SD): not reported (neither BMI nor other body weight or body weight status) Specific social or cultural characteristics: undergraduate students Socio‐economic status context: low deprivation Inclusion criteria: female; undergraduate student Exclusion criteria: none reported	
Interventions	Manipulated product type: food Manipulation: package size (candy‐coated chocolates) Duration of exposure to intervention: ≤ 1 day Social setting: consuming alone Study arms: small‐package‐present (8 x small, opaque packages ‐ 11 chocolates in each package), communicated caloric content absent, low appearance self esteem; small‐package‐present (8 x small, opaque packages ‐ 11 chocolates in each package), communicated caloric content absent, high appearance self esteem; small‐package‐present (8 x small, opaque packages ‐ 11 chocolates in each package), communicated caloric content low, low appearance self esteem; small‐package‐present (8 x small, opaque packages ‐ 11 chocolates in each package), communicated caloric content low, high appearance self esteem; small‐package‐present (8 x small, opaque packages ‐ 11 chocolates in each package), communicated caloric content high, low appearance self esteem; small‐package‐present (8 x small, opaque packages ‐ 11 chocolates in each package), communicated caloric content high, high appearance self esteem; small‐package‐absent (88 x loose, unpackaged chocolates), communicated caloric content absent, low appearance self esteem; small‐package‐ absent (88 x loose, unpackaged chocolates), communicated caloric content absent, high appearance self esteem; small‐package‐absent (88 x loose, unpackaged chocolates), communicated caloric content low, low appearance self esteem; small‐package‐absent (88 x loose, unpackaged chocolates), communicated caloric content low, high appearance self esteem; small‐package‐absent (88 x loose, unpackaged chocolates), communicated caloric content high, low appearance self esteem; small‐package‐absent (88 x loose, unpackaged chocolates), communicated caloric content high, high appearance self esteem Number of comparisons analysed: 1 Comparisons analysed: Intervention 1:‐ small‐package‐present (8 x small, opaque packages ‐ 11 chocolates in each package); *versus* Intervention 2:‐ large‐packages (88 x loose, unpackaged chocolates) Concurrent intervention components: no	
Outcomes	Outcomes reported in study: amount of candy‐coated chocolates consumed (grams) Selection outcome analysed: N/A Measurement of selection outcome: N/A Timing of selection outcome measurement: N/A Consumption outcome analysed: amount of candy‐coated chocolates consumed (grams) Measurement of consumption outcome: objective Timing of consumption outcome measurement: immediate (≤ 1 day)	
Funding source	Social Sciences and Humanities Research Council of Canada	
Notes	Outcome data for communicated caloric content low and communicated caloric content high, and low appearance self esteem and high appearance self esteem participant subgroups collapsed and analysed together (one comparison)	
***Risk of bias***		
**Bias**	**Authors' judgement**	**Support for judgement**
Random sequence generation (selection bias)	Unclear risk	Comment: method of sequence generation is not described. Insufficient information about the sequence generation process to permit judgement of 'low risk' or 'high risk'
Allocation concealment (selection bias)	Unclear risk	Comment: method of concealment is not described. Insufficient information to permit judgement of 'low risk' or 'high risk'
Blinding of participants and personnel (performance bias) Consumption outcome	Low risk	Quote: "Participants completed the experiment individually and were seated in a cubicle facing away from a female experimenter. Each participant was told that we were interested in evaluations of a variety of products and that they would be asked to sample one of the products while completing a questionnaire... Finally, participants completed an open‐ended suspicion probe assessing what they thought was the purpose of the research. Responses indicated that participants were not cognizant of the hypotheses in this or any of the other studies." Comment: blinding of study participants attempted and unlikely that the blinding could have been broken. Very unlikely that key study personnel were blinded, but the review authors judge that the outcome is not likely to be influenced by lack of blinding of key study personnel
Blinding of outcome assessment (detection bias) Consumption outcome	Low risk	Comment: no blinding of outcome assessment, but the review authors judge that the outcome measurement is not likely to be influenced by lack of blinding
Incomplete outcome data (attrition bias) Consumption outcome	Low risk	Comment: no missing outcome data for consumption outcome
Selective reporting (reporting bias)	Unclear risk	Comment: search for record(s) containing details of study protocol conducted in ClinicalTrials.gov and the WHO International Clinical Trials Registry Platform (ICTRP). No records found. Insufficient information to permit judgement of 'low risk' or 'high risk'
Other bias #1 ‐ Baseline comparability of participant characteristics between groups	Unclear risk	Comment: study uses a between‐subjects design. Insufficient information to permit judgement of 'low risk' or 'high risk'
Other bias #2 ‐ Consistency in intervention delivery	Low risk	Quote: "We used the same general procedure and cover story as described in Study 1, with a few notable changes. First, we measured ASE in an earlier session and subsequently linked ASE scores to participants’ responses in the focal session. In the session itself, participants were first given either eight small packages of candy‐coated chocolates or a bowl of loose product (with the same quantity). In addition, before receiving the product, participants were provided with caloric information regarding the candy. In the high‐calorie condition, they were told that 11 candies contained 150 calories, in the low‐calorie condition they were informed that 11 candies contained 50 calories, and in the information‐absent condition they were not provided with any caloric information." Comment: information provided to participants appears to have been standardised between the compared study conditions. No specific instructions were provided to participants and therefore monitoring of participants' compliance with instructions is not applicable
Summary of risk of bias Consumption outcome	Unclear risk	Unclear risk

**Table:** Argo 2012 (S5)

Methods	Study design: between‐subjects randomised controlled trial	
Participants	Setting: laboratory setting Geographical region: Canada Number of enrolled participants: 105 female undergraduate students Number (%) of enrolled participants completing the study: 105 (100%) Study completers ‐ mean age (SD): not reported Study completers ‐ sex: female only Study completers ‐ mean BMI kg/m^2^ (SD): not reported (neither BMI nor other body weight or body weight status) Specific social or cultural characteristics: undergraduate students Socio‐economic status context: low deprivation Inclusion criteria: female; undergraduate student Exclusion criteria: none reported	
Interventions	Manipulated product type: food Manipulation: package size (candy‐coated chocolates) Duration of exposure to intervention: ≤ 1 day Social setting: consuming alone Study arms: small‐package‐present (8 x small, opaque packages ‐ 11 chocolates in each package), cognitive load low, low appearance self esteem; small‐package‐present (8 x small, opaque packages ‐ 11 chocolates in each package), cognitive load low, high appearance self esteem; small‐package‐present (8 x small, opaque packages ‐ 11 chocolates in each package), cognitive load high, low appearance self esteem; small‐package‐present (8 x small, opaque packages ‐ 11 chocolates in each package), cognitive load high, high appearance self esteem; small‐package‐absent (88 x loose, unpackaged chocolates), cognitive load low, low appearance self esteem; small‐package‐absent (88 x loose, unpackaged chocolates), cognitive load low, high appearance self esteem; small‐package‐absent (88 x loose, unpackaged chocolates), cognitive load high, low appearance self esteem; small‐package‐absent (88 x loose, unpackaged chocolates), cognitive load high, high appearance self esteem Number of comparisons analysed: 0 Comparisons analysed: N/A – no usable outcome data Concurrent intervention components: no	
Outcomes	Outcomes reported in study: amount of candy‐coated chocolates consumed (grams) Selection outcome analysed: N/A Measurement of selection outcome: N/A Timing of selection outcome measurement: N/A Consumption outcome analysed: N/A – no usable outcome data Measurement of consumption outcome: N/A – no usable outcome data Timing of consumption outcome measurement: N/A – no usable outcome data	
Funding source	Social Sciences and Humanities Research Council of Canada	
Notes	No usable outcome data in published study report. Attempts made to contact study authors (Jennifer Argo and Katherine White) via e‐mail, but no contact established	
***Risk of bias***		
**Bias**	**Authors' judgement**	**Support for judgement**
Random sequence generation (selection bias)	Unclear risk	Comment: method of sequence generation is not described. Insufficient information about the sequence generation process to permit judgement of 'low risk' or 'high risk'
Allocation concealment (selection bias)	Unclear risk	Comment: method of concealment is not described. Insufficient information to permit judgement of 'low risk' or 'high risk'
Blinding of participants and personnel (performance bias) Consumption outcome	Low risk	Quote: "Participants completed the experiment individually and were seated in a cubicle facing away from a female experimenter. Each participant was told that we were interested in evaluations of a variety of products and that they would be asked to sample one of the products while completing a questionnaire... Finally, participants completed an open‐ended suspicion probe assessing what they thought was the purpose of the research. Responses indicated that participants were not cognizant of the hypotheses in this or any of the other studies." Comment: blinding of study participants attempted and unlikely that the blinding could have been broken. Very unlikely that key study personnel were blinded, but the review authors judge that the outcome is not likely to be influenced by lack of blinding of key study personnel
Blinding of outcome assessment (detection bias) Consumption outcome	Low risk	Comment: no blinding of outcome assessment, but the review authors judge that the outcome measurement is not likely to be influenced by lack of blinding
Incomplete outcome data (attrition bias) Consumption outcome	Low risk	Comment: no missing outcome data for consumption outcome
Selective reporting (reporting bias)	Unclear risk	Comment: search for record(s) containing details of study protocol conducted in ClinicalTrials.gov and the WHO International Clinical Trials Registry Platform (ICTRP). No records found. Insufficient information to permit judgement of 'low risk' or 'high risk'
Other bias #1 ‐ Baseline comparability of participant characteristics between groups	Unclear risk	Comment: study uses a between‐subjects design. Insufficient information to permit judgement of 'low risk' or 'high risk'
Other bias #2 ‐ Consistency in intervention delivery	Low risk	Quote: "The procedure was similar to that used in Study 1, except participants were told that they would be completing multiple surveys and that the first study involved memory. A common method used to demonstrate whether a particular process is cognitively effortful is a cognitive load task...Thus, following Shiv and Huber..., participants in the low‐load condition were asked to memorize a two‐digit number, whereas those in the high‐load condition were asked to memorize an eight‐digit number. Participants were then given the product (i.e., candy‐coated chocolate) to consume and the survey to complete." Comment: information provided to participants appears to have been standardised between the compared study conditions. No specific instructions were provided to participants and therefore monitoring of participants' compliance with instructions is not applicable
Summary of risk of bias Consumption outcome	Unclear risk	Unclear risk

**Table:** Burger 2011

Methods	Study design: within‐subjects randomised controlled trial	
Participants	Setting: laboratory setting Geographical region: Colorado State University, Fort Collins, Colorado, USA Number of enrolled participants: 30 adults Number (%) of enrolled participants completing the study: 27 (90%) Study completers ‐ mean age (SD): 37.4 (11.1) Study completers ‐ sex: male (44%) and female (56%) Study completers ‐ mean BMI kg/m^2^ (SD): 25.9 (4.5) Specific social or cultural characteristics: none Socio‐economic status context: low deprivation Inclusion criteria: aged between 18 and 60 years; willingness to eat the foods offered in the study; ability to read and understand English language at a 6th grade level Exclusion criteria: pregnancy; restrictive dietary practices (e.g. vegetarianism or food allergies); taste or visual impairment that could interfere with data collection	
Interventions	Manipulated product type: food Manipulation: portion size Duration of exposure to intervention: ≤ 1 day Social setting: consuming alone Study arms: small portion (410 ± 10 g Three Cheese Italiano pasta dish), participants blindfolded; small portion (410 ± 10 g Three Cheese Italiano pasta dish), food visible (participants not blindfolded); large portion (820 ± 10 g Three Cheese Italiano pasta dish), participants blindfolded; large portion ((820 ± 10 g Three Cheese Italiano pasta dish), food visible (participants not blindfolded) Number of comparisons analysed: 1 Comparisons analysed: Intervention 1: small portion (410 ± 10 g Three Cheese Italiano pasta dish); *versus* Intervention 2: large portion (820 ± 10g Three Cheese Italiano pasta dish) Concurrent intervention components: no	
Outcomes	Outcomes reported in study: energy intake from total meal (kcal); energy intake from entrée (kcal); energy intake from complementary foods (kcal); total meal duration (minutes); number of bites from total meal (N); bite size (grams) Selection outcome analysed: N/A Measurement of selection outcome: N/A Timing of selection outcome measurement: N/A Consumption outcome analysed: energy intake from total meal (kcal) Measurement of consumption outcome: objective Timing of consumption outcome measurement: immediate (≤ 1 day)	
Funding source	Helen F. McHugh Graduate Research Fellowship, Colorado State University; National Research Initiative of the USDA Cooperative State Research, Education and Extension Service (Grant number # 2006‐55215‐16726)	
Notes	Outcome data for blindfolded and food visible (not blindfolded) participant subgroups collapsed and analysed together (one comparison). Author contacted to request information missing from the study report ‐ requested information was supplied (February 2014)	
***Risk of bias***		
**Bias**	**Authors' judgement**	**Support for judgement**
Random sequence generation (selection bias)	Unclear risk	Comment: method of sequence generation for condition order is not described. Insufficient information about the sequence generation process to permit judgement of 'low risk' or 'high risk'
Allocation concealment (selection bias)	Unclear risk	Comment: method of concealment is not described. Insufficient information to permit judgement of 'low risk' or 'high risk'
Blinding of participants and personnel (performance bias) Consumption outcome	Unclear risk	Quote: "The participants were not told the purpose of the study, but were told that the aim was to investigate the effects of visibility on sensory aspects of food intake (i.e., taste and mouth feel)... Any comments made by the participant were recorded by research staff throughout the study session. An informal discharge interview was performed at the end of the last study session. Participants were queried regarding their thoughts about the purpose of the study [and] whether they noticed differences in the meal between study sessions... The majority of the participants noticed the difference in portion size, yet no participant was able to deduce the purpose of the study." Comment: blinding of study participants attempted but likely that blinding was broken in many cases and it is possible that the outcome may be influenced by lack of blinding (due to potential carry‐over effects between conditions). Very unlikely that key study personnel were blinded, but the review authors judge that the outcome is not likely to be influenced by lack of blinding of key study personnel
Blinding of outcome assessment (detection bias) Consumption outcome	Low risk	Comment: no blinding of outcome assessment, but the review authors judge that the outcome measurement is not likely to be influenced by lack of blinding
Incomplete outcome data (attrition bias) Consumption outcome	Low risk	Quote: "In testing the effect of portion size on intake, consuming all of the entrée (plate cleaning) can skew data, inflating the effect of the increase in portion. Our study included three steps to account for the effect of plate cleaning: pilot testing of the portion sizes, operationally defining a "plate cleaner" and completing an analysis to determine whether a plate cleaner × portion size interaction existed. Based on previous literature ... a participant was defined as plate cleaner if they left ≤ 20 g of the entrée in both of the small portion conditions (blindfolded and visible)...A total of 30 individuals (M = 15, F = 15) completed the study, and three men (BMI = 31.3 ± 4.4) were identified as plate cleaners. In addition to consuming all of the small portions, one of these men left ≤ 20 g of the large portion entrée in the blindfolded condition. No participant left ≤ 20 g of the large portion entrée in the visible condition. A plate cleaner × portion size interaction was observed (P < 0.001). The plate cleaners had a significantly larger response to the increase in portion size suggesting that they would have possibly continued to eat in the small portion condition if there was more food available. Because the plate cleaners were restricted by the amount of food presented in the small portion conditions and likely were not able to eat until full, their response to portion size was inflated, thus skewing the data and they were eliminated from further analyses." Comment: the reason for missing outcome data for consumption outcome is the study authors' decision to exclude participants who left ≤ 20 g of the entrée in both of the small portion conditions ('plate cleaners') from the analysis. The review authors judge that this decision is reasonable, as it produces a more conservative estimate of the effect of the intervention on consumption. Any attrition bias due to handling of incomplete outcome data produces a more conservative estimate of the effect of the intervention on consumption
Selective reporting (reporting bias)	Unclear risk	Comment: search for record(s) containing details of study protocol conducted in ClinicalTrials.gov and the WHO International Clinical Trials Registry Platform (ICTRP). No records found. Insufficient information to permit judgement of 'low risk' or 'high risk'
Other bias #1 ‐ Baseline comparability of participant characteristics between groups	Low risk	Quote: "After consent was completed and all questions regarding the study were answered, participants filled out a series of premeal visual analog scales (VAS). Pre and postmeal VAS were used to rate the participants' hunger, thirst, and fullness using a 0–100 mm scale, anchored by "not at all" and "extremely."...Additionally analyses were performed to test for possible effects of order independent of conditions, no significant effects were observed...[Pre‐meal] hunger, thirst, and fullness...did not vary across any of the...experimental conditions." Comment: study uses a within‐subjects design. No differences between conditions in terms of measured pre‐condition participant 'state' characteristics, but not reported whether there were differences between condition orders in terms of measured pre‐condition participant 'state' characteristics. However, a statistical analysis was conducted to test for the potential influence of condition order on measured outcomes and no influence was observed. It is therefore unlikely that any differences between condition orders in terms of measured pre‐condition participant 'state' characteristics influenced the measured outcomes. Risk of bias due to period effects is therefore judged low
Other bias #2 ‐ Consistency in intervention delivery	Unclear risk	Quote: "Participants were instructed to have a typical breakfast on study session days... The participants were then presented with a meal...and were instructed to eat ad libitum... One member of the research staff recorded number of bites of the entrée via direct observation behind a two‐way mirror at every session." Comment: information and instructions provided to participants appear to have been standardised between the compared study conditions. No information pertaining to monitoring of participants' compliance with the instruction to have a typical breakfast on study session days is reported. No monitoring results are reported with respect to this instruction
Summary of risk of bias Consumption outcome	Unclear risk	Unclear risk

**Table:** Cavanagh 2013

Methods	Study design: between‐subjects randomised controlled trial	
Participants	Setting: laboratory setting Geographical region: University of New South Wales, Sydney, Australia Number of enrolled participants: 96 female undergraduate students Number (%) of enrolled participants completing the study: 96 (100%) Study completers ‐ mean age (SD): 19.7 (4.7) Study completers ‐ sex: female only Study completers ‐ mean BMI kg/m^2^ (SD): 21.5 (3.1) Specific social or cultural characteristics: undergraduate students Socio‐economic status context: low deprivation Inclusion criteria: female; undergraduate student; enrolled in a first‐year psychology course Exclusion criteria: none reported	
Interventions	Manipulated product type: food Manipulation: portion size Duration of exposure to intervention: ≤ 1 day Social setting: consuming with others Study arms: small portion (350 g macaroni pasta with tomato sauce, plus approximately 750 g macaroni pasta with tomato sauce in a large serving bowl ‐ approximately 1100 g total available), education information leaflet and an associated 6‐minute activity intended to assist with the consolidation of the information that participants were provided with; small portion (350 g macaroni pasta with tomato sauce, plus approximately 750 g macaroni pasta with tomato sauce in a large serving bowl ‐ approximately 1100 g total available), mindfulness information leaflet and an associated 6‐minute activity intended to assist with the consolidation of the information that participants were provided with; small portion (350 g macaroni pasta with tomato sauce, plus approximately 750 g macaroni pasta with tomato sauce in a large serving bowl ‐ approximately 1100g total available), sleep hygiene information leaflet and an associated 6‐minute activity intended to assist with the consolidation of the information that participants were provided with (control); large portion (600 g macaroni pasta with tomato sauce, plus approximately 500 g macaroni pasta with tomato sauce in a large serving bowl ‐ approximately 1100g total available), education information leaflet and an associated 6‐minute activity intended to assist with the consolidation of the information that participants were provided with; large portion (600 g macaroni pasta with tomato sauce, plus approximately 500 g macaroni pasta with tomato sauce in a large serving bowl ‐ approximately 1100 g total available), mindfulness information leaflet and an associated 6‐minute activity intended to assist with the consolidation of the information that participants were provided with; large portion (600 g macaroni pasta with tomato sauce, plus approximately 500 g macaroni pasta with tomato sauce in a large serving bowl ‐ approximately 1100 g total available), sleep hygiene information leaflet and an associated 6‐minute activity intended to assist with the consolidation of the information that participants were provided with (control) Number of comparisons analysed: 1 Comparisons analysed: Intervention 1: small portion (350 g macaroni pasta with tomato sauce, plus approximately 750 g macaroni pasta with tomato sauce in a large serving bowl ‐ approximately 1100 g total available); *versus* Intervention 2: large portion (600 g macaroni pasta with tomato sauce, plus approximately 500 g macaroni pasta with tomato sauce in a large serving bowl ‐ approximately 1100 g total available). Concurrent intervention components: yes. Information leaflet (education versus mindfulness versus control) plus an associated 6‐minute activity ‐ provided to both the Intervention 1 and Intervention 2 groups	
Outcomes	Outcomes reported in study: energy intake from macaroni with tomato sauce (kcal); amount of macaroni with tomato sauce consumed (grams) Selection outcome analysed: N/A Measurement of selection outcome: N/A Timing of selection outcome measurement: N/A Consumption outcome analysed: energy intake from macaroni with tomato sauce (kcal) Measurement of consumption outcome: objective Timing of consumption outcome measurement: immediate (≤ 1 day)	
Funding source	Australian Research Council's Discovery Projects funding scheme (Project number DP110101124)	
Notes	Outcome data for education, mindfulness and control information leaflet and associated activity participant subgroups collapsed and analysed together (one comparison). Author contacted to request information missing from the study report ‐ requested information was supplied (February 2014)	
***Risk of bias***		
**Bias**	**Authors' judgement**	**Support for judgement**
Random sequence generation (selection bias)	Unclear risk	Comment: method of sequence generation for condition order is not described. Insufficient information about the sequence generation process to permit judgement of 'low risk' or 'high risk'
Allocation concealment (selection bias)	Unclear risk	Comment: method of concealment is not described. Insufficient information to permit judgement of 'low risk' or 'high risk'
Blinding of participants and personnel (performance bias) Consumption outcome	Low risk	Quote: "Upon arrival, participants were informed that the study consisted of two separate components: the first testing different types of health‐related information and the second examining individual aspects of taste sensitivity over the course of a meal... Participants were then probed for suspicion (no participant expressed suspicion about the hypotheses) and were debriefed about the true nature of the experiment." Comment: blinding of study participants attempted and unlikely that the blinding could have been broken. Participants were probed for suspicion of study purpose. Very unlikely that key study personnel were blinded, but the review authors judge that the outcome is not likely to be influenced by lack of blinding of key study personnel
Blinding of outcome assessment (detection bias) Consumption outcome	Low risk	Comment: no blinding of outcome assessment, but the review authors judge that the outcome measurement is not likely to be influenced by lack of blinding
Incomplete outcome data (attrition bias) Consumption outcome	Low risk	Comment: no missing outcome data for consumption outcome
Selective reporting (reporting bias)	Unclear risk	Comment: search for record(s) containing details of study protocol conducted in ClinicalTrials.gov and the WHO International Clinical Trials Registry Platform (ICTRP). No records found. Insufficient information to permit judgement of 'low risk' or 'high risk'
Other bias #1 ‐ Baseline comparability of participant characteristics between groups	Low risk	Quote: "A 10‐item taste‐rating scale was included to... control for any possible confounding influence of liking of the food on consumption... Prior to eating the pasta, participants were asked to rate their current hunger level along a 10‐cm visual analog scale, with not at all hungry and extremely hungry as the anchors... We also measured dietary restraint...and positive and negative affect to include as potential covariates. Those variables had no impact on the results of the study and are therefore not discussed further... After [the experiment]...participants were asked to...provide some basic demographic information (age, height, and weight, which were used to calculate their BMI)...Prior to the main analyses, correlational analyses were conducted to identify potential covariates. Ratings of initial hunger... and liking of the food...were significantly associated with total food consumed, but BMI was unrelated to food intake...Thus, only hunger and liking were included as covariates in all subsequent analyses relating to total food consumed." Comment: study uses a between‐subjects design. Difference between comparison groups in terms of baseline ratings of hunger and liking of the manipulated foods. The statistical analysis of outcome data controls for these differences. No information pertaining to differences between comparison groups in terms of age is reported
Other bias #2 ‐ Consistency in intervention delivery	Low risk	Quote: "Next, participants completed an initial hunger questionnaire and took part in the tasting component of the study. They were told that they could eat as much as they wanted of the meal and were asked to complete the taste‐rating forms after their first and last mouthfuls." Comment: information and instructions provided to participants appear to have been standardised between the compared study conditions. No specific instructions, other than the instruction that they could eat as much as they wanted of the meal, were provided to participants and therefore monitoring of participants' compliance with instructions is not applicable
Summary of risk of bias Consumption outcome	Unclear risk	Unclear risk

**Table:** Coelho do Vale 2008 (S2)

Methods	Study design: between‐subjects randomised controlled trial	
Participants	Setting: laboratory setting Geographical region: Tilburg University, Tilburg, Netherlands Number of enrolled participants: 140 undergraduate students Number (%) of enrolled participants completing the study: 73 (52%) Study completers ‐ mean age (SD): 21.3 (2.0) Study completers ‐ sex: male (70%) and female (30%) Study completers ‐ mean BMI kg/m^2^ (SD): not reported (neither BMI nor other body weight or body weight status) Specific social or cultural characteristics: undergraduate students Socio‐economic status context: low deprivation Inclusion criteria: undergraduate student Exclusion criteria: none reported	
Interventions	Manipulated product type: food Manipulation: package size (potato chips) Duration of exposure to intervention: ≤ 1 day Social setting: consuming alone Study arms: small package format (9 x 45 g packages potato chips ‐ 405 g total), self regulatory concerns not activated; small package format (9 x 45 g packages potato chips ‐ 405 g total), self regulatory concerns activated; large package format (2 x 200 g packages potato chips ‐ 400 g total), self regulatory concerns not activated; large package format (2 x 200 g packages potato chips ‐ 400 g total), self regulatory concerns activated Number of comparisons analysed: 1 Comparisons analysed: Intervention 1: small package format (9 x 45 g packages potato chips ‐ 405 g total); *versus* Intervention 2: large package format (2 x 200 g packages potato chips ‐ 400 g total) Concurrent intervention components: yes. Regulatory concerns (not activated versus activated) ‐ provided to both the Intervention 1 and Intervention 2 groups	
Outcomes	Outcomes reported in study: amount of potato chips consumed (grams); any potato chips consumed? (dichotomous) Selection outcome analysed: N/A Measurement of selection outcome: N/A Timing of selection outcome measurement: N/A Consumption outcome analysed: amount of potato chips consumed (grams) Measurement of consumption outcome: objective Timing of consumption outcome measurement: immediate (≤ 1 day)	
Funding source	Portuguese Foundation for Science and Technology	
Notes	Outcome data for regulatory concerns not activated and regulatory concerns activated participant subgroups collapsed and analysed together (one comparison). Author contacted to request information missing from the study report ‐ requested information was supplied (February 2014)	
***Risk of bias***		
**Bias**	**Authors' judgement**	**Support for judgement**
Random sequence generation (selection bias)	Unclear risk	Comment: method of sequence generation for condition order is not described. Insufficient information about the sequence generation process to permit judgement of 'low risk' or 'high risk'
Allocation concealment (selection bias)	Unclear risk	Comment: method of concealment is not described. Insufficient information to permit judgement of 'low risk' or 'high risk'
Blinding of participants and personnel (performance bias) Consumption outcome	Low risk	Quote: "Participants first read that the purpose of the study was to assess and understand their reactions and opinions about TV commercials. Then, to increase the believability of the cover story, participants were asked to indicate on 7‐point scales their general opinion about TV commercials (e.g., "TV commercials are amusing to watch": not at all–very much), followed by an example of the main task that they were going to perform: the ad evaluation task. Then, participants read "During the next 20 minutes you will perform an 'ad evaluation' task. Since most commercials are usually watched at home, we want to recreate as much as possible a normal home environment while you watch the commercials. Therefore, we also included an extract from a 'Friends' episode (sitcom) to mimic regular TV viewing. Moreover, since previous studies have shown that 70% of the snacks are consumed while watching TV, you'll find next to the computer a bowl with potato chips that you can eat while doing this study."... At the end, participants answered questions about their consumption decision and debriefing questions... Upon completion of the experiment, a funneled debriefing methodology was used...to assess suspicion and hypothesis guessing. Participants were asked to indicate what they thought the purpose of the study was, what it was trying to assess, if there was something unusual in the study, and if they had any specific goal while participating. None of the participants showed suspicion or identified the true purpose of the study." Comment: blinding of study participants attempted and unlikely that the blinding could have been broken. Participants were probed for suspicion of study purpose. Very unlikely that key study personnel were blinded, but the review authors judge that the outcome is not likely to be influenced by lack of blinding of key study personnel
Blinding of outcome assessment (detection bias) Consumption outcome	Low risk	Comment: no blinding of outcome assessment, but the review authors judge that the outcome measurement is not likely to be influenced by lack of blinding
Incomplete outcome data (attrition bias) Consumption outcome	High risk	Comment: the reason for missing outcome data for consumption outcome is the study authors' decision to exclude participants with zero consumption from the analysis. The substantial proportion (67 participants, 55% of study sample) of exclusions due to zero consumption and the differential distribution between arms means that the review authors judge that it is plausible that the effect size among these missing data is enough to have had an important impact on the observed effect size
Selective reporting (reporting bias)	Unclear risk	Comment: search for record(s) containing details of study protocol conducted in ClinicalTrials.gov and the WHO International Clinical Trials Registry Platform (ICTRP). No records found. Insufficient information to permit judgement of 'low risk' or 'high risk'
Other bias #1 ‐ Baseline comparability of participant characteristics between groups	Unclear risk	Comment: study uses a between‐subjects design. Insufficient information to permit judgement of 'low risk' or 'high risk'
Other bias #2 ‐ Consistency in intervention delivery	Low risk	Quote: "...participants [in each condition] read "During the next 20 minutes you will perform an 'ad evaluation' task. Since most commercials are usually watched at home, we want to recreate as much as possible a normal home environment while you watch the commercials. Therefore, we also included an extract from a 'Friends' episode (sitcom) to mimic regular TV viewing. Moreover, since previous studies have shown that 70% of the snacks are consumed while watching TV, you'll find next to the computer a bowl with potato chips that you can eat while doing this study." Comment: information provided to participants appears to have been standardised between the compared study conditions. No specific instructions were provided to participants and therefore monitoring of participants' fidelity to protocol is not applicable
Summary of risk of bias Consumption outcome	Unclear risk	Unclear risk

**Table:** Devitt 2004

Methods	Study design: within‐subjects randomised controlled trial	
Participants	Setting: laboratory setting Geographical region: Purdue University, West Lafayette, Illinois, USA Number of enrolled participants: 26 adults Number (%) of enrolled participants completing the study: 20 (77%) Study completers ‐ mean age (SD): 22.6 (5.8) Study completers ‐ sex: male (55%) and female (45%) Study completers ‐ mean BMI kg/m^2^ (SD): 25.3 (4.3) Specific social or cultural characteristics: none Socio‐economic status context: low deprivation Inclusion criteria: score of ≥ 5 on a 9‐point hedonic scale for the foods used in study; aged between 18 and 50 years; BMI between 18 and 33; typical meal pattern of 3 meals per day Exclusion criteria: none reported	
Interventions	Manipulated product type: food Manipulation: individual unit size (various foods) Duration of exposure to intervention: ≤ 1 day Social setting: consuming alone Study arms: small food unit size (96 x 13 g omelettes ‐ 1244 g total; 48 x 24 g wraps ‐ 1158 g total; 92 x 12 g pizzas ‐ 1110 g total), low energy density; small food unit size (96 x 13 g omelettes ‐1244 g total; 48 x 24 g wraps ‐ 1158 g total; 92 x 12 g pizzas ‐ 1110g total), high energy density; customary (larger) food unit size (4 x 311 g omelettes ‐ 1244 g total; 6 x 193 g wraps ‐ 1158 g total; 2 x 555 g pizzas ‐ 1110 g total), low energy density; customary (larger) food unit size (4 x 311 g omelettes ‐ 1244 g total; 6 x 193 g wraps ‐ 1158 g total; 2 x 555 g pizzas ‐ 1110g total), high energy density Number of comparisons analysed: 1 Comparisons analysed: Intervention 1: small food unit size (96 x 13 g omelettes ‐1244 g total; 48 x 24 g wraps ‐ 1158 g total; 92 x 12 g pizzas ‐ 1110 g total); *versus* Intervention 2: customary (larger) food unit size (4 x 311 g omelettes ‐ 1244 g total; 6 x 193 g wraps ‐ 1158 g total; 2 x 555 g pizzas ‐ 1110 g total) Concurrent intervention components: yes. Energy density (low versus high) ‐ provided to both the Intervention 1 and Intervention 2 groups	
Outcomes	Outcomes reported in study: total daily energy intake (kcal); total amount of food consumed during day from breakfast, lunch and dinner (grams); energy intake from breakfast (kcal); amount of food consumed from breakfast (grams); energy intake from lunch (kcal); amount of food consumed from lunch (grams); energy intake from dinner (kcal); amount of food consumed from dinner (grams) Selection outcome analysed: N/A Measurement of selection outcome: N/A Timing of selection outcome measurement: N/A Consumption outcome analysed: total daily energy intake (kcal) Measurement of consumption outcome: objective Timing of consumption outcome measurement: immediate (≤ 1 day)	
Funding source	Not reported	
Notes	Outcome data for low energy density and high energy density participant subgroups collapsed and analysed together (one comparison)	
***Risk of bias***		
**Bias**	**Authors' judgement**	**Support for judgement**
Random sequence generation (selection bias)	Unclear risk	Comment: method of sequence generation for condition order is not described. Insufficient information about the sequence generation process to permit judgement of 'low risk' or 'high risk'
Allocation concealment (selection bias)	Unclear risk	Comment: method of concealment is not described. Insufficient information to permit judgement of 'low risk' or 'high risk'
Blinding of participants and personnel (performance bias) Consumption outcome	Unclear risk	Quote: "Each meal occasion required a 1.5 h stay in the laboratory during which, the participant completed a hunger questionnaire, and tests of cognitive ability and manual dexterity at time zero (prior to the meal), and 45 and 90 min post‐meal. The latter two tests were included to distract participants from the study's purpose." Comment: blinding of study participants attempted. Not reported whether participants were probed for suspicion of study purpose or awareness of size manipulation between study conditions. It is possible that blinding of study participants was broken in some cases and it is possible that the outcome may be influenced by this lack of blinding (due to potential carry‐over effects between conditions). Very unlikely that key study personnel were blinded, but the review authors judge that the outcome is not likely to be influenced by lack of blinding of key study personnel
Blinding of outcome assessment (detection bias) Consumption outcome	Low risk	Comment: no blinding of outcome assessment, but the review authors judge that the outcome measurement is not likely to be influenced by lack of blinding
Incomplete outcome data (attrition bias) Consumption outcome	Low risk	Quote: "Six (two male and four female) participants did not complete all sessions of the study due to insufficient time to devote toward the study. They were not different from those who did complete the study on baseline characteristics. Eleven males and nine females completed the study. Data reported includes only those 20 persons completing all study sessions." Comment: reason for missing outcome data is unlikely to be related to consumption outcome and study authors state that participants who did not complete the study are not different from those who did in terms of baseline characteristics
Selective reporting (reporting bias)	Unclear risk	Comment: search for record(s) containing details of study protocol conducted in ClinicalTrials.gov and the WHO International Clinical Trials Registry Platform (ICTRP). No records found. Insufficient information to permit judgement of 'low risk' or 'high risk'
Other bias #1 ‐ Baseline comparability of participant characteristics between groups	Unclear risk	Quote: "Participants were asked to answer appetitive questions using a nine‐point category scale. They were asked to choose the number that best reflected their response for each question. The question "How hungry do you feel right now?" was anchored with "not at all hungry" at 1 and "as hungry as I've ever felt" at 9. "How strong is your desire to eat right now?" was anchored with "very weak" and "very strong" and "How much food do you think you could eat right now?" was anchored with "nothing at all" and "a large amount". The question regarding fullness ("How full does your stomach feel right now?") was anchored with "not at all full" and "very full"... Breakfast, lunch and dinner mean ratings for hunger and fullness were not different across treatments at 0... min (Table 4)." Comment: study uses a within‐subjects design. Differences between conditions in terms of measured pre‐condition participant 'state' characteristics are reported, but not reported whether there were differences between condition orders in terms of measured pre‐condition participant 'state' characteristics. No analysis of potential differences in measured outcomes between condition orders appears to have been conducted and the statistical analysis of outcome data does not appear to control for condition order. Risk of bias due to period effects is therefore unclear. Insufficient information to permit judgement of 'low risk' or 'high risk'
Other bias #2 ‐ Consistency in intervention delivery	Unclear risk	Quote: "Subjects were instructed to follow a 10 [hour] overnight fast on the evening before each study day... Upon arrival for each meal they were instructed to eat as much as they wanted and, if they desired, more food would be provided... Participants were permitted to leave the laboratory between meals and were instructed not to consume foods or beverages outside of the laboratory." Comment: information and instructions provided to participants appear to have been standardised between the compared study conditions. No information pertaining to monitoring of participants' compliance with the instruction to follow a 10‐hour overnight fast on the evening before each study day is reported and no further specific instructions were provided, other than the instruction to eat as much as they wanted. Insufficient information to permit judgement of 'low risk' or 'high risk'
Summary of risk of bias Consumption outcome	Unclear risk	Unclear risk

**Table:** Diliberti 2004

Methods	Study design: between‐subjects cluster‐randomised controlled trial Unit of allocation: day of the week Unit of analysis: individual Number of clusters: 4 Number of participants per cluster: not reported Analysis does not appear to account for cluster allocation, as the statistical model does not appear to include any covariate related to cluster assignment	
Participants	Setting: field setting, public cafeteria‐style restaurant Geographical region: Pennsylvania State University campus, University Park, Pennsylvania, USA Number of enrolled participants: 180 adults Number (%) of enrolled participants completing the study: 180 (100%) Study completers ‐ mean age (SD): 22.6 (5.8) Study completers ‐ sex: male (55%) and female (45%) Study completers ‐ mean BMI kg/m^2^ (SD): 25.3 (4.3) Specific social or cultural characteristics: customers of a university campus public cafeteria‐style restaurant Socio‐economic status context: low deprivation Inclusion criteria: purchaser of the pasta entrée on a study day; willing to complete a short survey Exclusion criteria: had purchased the pasta entrée on a previous study day; has shared meal with another person	
Interventions	Manipulated product type: food Manipulation: portion size Duration of exposure to intervention: ≤ 1 day Social setting: consuming with others Study arms: 100% portion size pasta entrée (ziti pasta, canned diced tomatoes, four cheeses ‐ ricotta, mozzarella, provolone and Romano ‐ heavy cream, fresh basil, garlic, salt and pepper ‐ mean cooked weight of 248.4 +/‐ 0.4 g), with standard size one‐half a tomato topped with pesto and standard size white bread roll with a butter packet; 150% portion size pasta entrée (ziti pasta, canned diced tomatoes, four cheeses ‐ ricotta, mozzarella, provolone and Romano ‐ heavy cream, fresh basil, garlic, salt and pepper mean cooked weight of 376.6 +/‐ 0.6 g), with standard size one‐half a tomato topped with pesto and standard size white bread roll with a butter packet Number of comparisons analysed: 1 Comparisons analysed: Intervention 1: 100% portion size pasta entrée (ziti pasta, canned diced tomatoes, four cheeses ‐ ricotta, mozzarella, provolone and Romano ‐ heavy cream, fresh basil, garlic, salt and pepper ‐ mean cooked weight of 248.4 +/‐ 0.4 g), with standard size one‐half a tomato topped with pesto and standard size white bread roll with a butter packet; *versus* Intervention 2: 150% portion size pasta entrée (ziti pasta, canned diced tomatoes, four cheeses ‐ ricotta, mozzarella, provolone and Romano ‐ heavy cream, fresh basil, garlic, salt and pepper mean cooked weight of 376.6 +/‐ 0.6 g), with standard size one‐half a tomato topped with pesto and standard size white bread roll with a butter packet Concurrent intervention components: no	
Outcomes	Outcomes reported in study: energy intake from total lunch meal (kcal); energy intake from pasta entrée (kcal); energy intake from standard portion accompaniments ‐ half tomato, bread roll and butter portion (kcal); energy intake from any side dishes (kcal); energy intake from any desserts (kcal); energy intake from any beverages (kcal) Selection outcome analysed: N/A Measurement of selection outcome: N/A Timing of selection outcome measurement: N/A Consumption outcome analysed: energy intake from total lunch meal (kcal) Measurement of consumption outcome: objective Timing of consumption outcome measurement: immediate (≤ 1 day)	
Funding source	US National Institutes of Health Grant (DK59853).	
Notes	—	
***Risk of bias***		
**Bias**	**Authors' judgement**	**Support for judgement**
Random sequence generation (selection bias)	Unclear risk	Quote: "On 10 days over 5 months, we covertly recorded the food intake of customers who purchased a baked pasta entrée from a serving line at lunch. On 5 of the days, the portion size of the entrée was the standard (100%) portion, and on 5 different days, the size was increased to 150% of the standard portion. The same portion size of the entrée was sold on two consecutive days of a given study week (Monday to Thursday). Study weeks were separated by at least 2 weeks, and the portion size sold in a given week was randomly determined."
Allocation concealment (selection bias)	High risk	Quote: "On 10 days over 5 months, we covertly recorded the food intake of customers who purchased a baked pasta entrée from a serving line at lunch. On 5 of the days, the portion size of the entrée was the standard (100%) portion, and on 5 different days, the size was increased to 150% of the standard portion. The same portion size of the entrée was sold on two consecutive days of a given study week (Monday to Thursday). Study weeks were separated by at least 2 weeks, and the portion size sold in a given week was randomly determined." Comment: explicitly unconcealed procedure
Blinding of participants and personnel (performance bias) Consumption outcome	Unclear risk	Quote: "...the customers in this study ate significantly more when the portion was increased, and their responses to the survey indicated that many were unaware that the portion was larger than normal or that they had eaten more food." Comment: no blinding or incomplete blinding of study participants and it is possible that the outcome may be influenced by lack of blinding. Very unlikely that key study personnel were blinded, but the review authors judge that the outcome is not likely to be influenced by lack of blinding of key study personnel
Blinding of outcome assessment (detection bias) Consumption outcome	Low risk	Comment: no blinding of outcome assessment, but the review authors judge that the outcome measurement is not likely to be influenced by lack of blinding
Incomplete outcome data (attrition bias) Consumption outcome	Low risk	Comment: no missing outcome data for consumption outcome.
Selective reporting (reporting bias)	Unclear risk	Comment: search for record(s) containing details of study protocol conducted in ClinicalTrials.gov and the WHO International Clinical Trials Registry Platform (ICTRP). No records found. Insufficient information to permit judgement of 'low risk' or 'high risk'
Other bias #1 ‐ Baseline comparability of participant characteristics between groups	Unclear risk	Comment: study uses a between‐subjects design. Insufficient information to permit judgement of 'low risk' or 'high risk'
Other bias #2 ‐ Consistency in intervention delivery	Low risk	Comment: information provided to participants appears to have been standardised between the compared study conditions. No specific instructions were provided to participants and therefore participants' compliance with instructions is not applicable
Summary of risk of bias Consumption outcome	High risk	High risk

**Table:** DiSantis 2013

Methods	Study design: within‐subjects cluster‐randomised controlled trial Unit of allocation: classroom Unit of analysis: individual Number of clusters: 2 Number of participants per cluster: not reported Analysis appears to account for cluster allocation, as generalised estimating equations were used to evaluate effects	
Participants	Setting: field setting, privately funded urban elementary school Geographical region: Philadelphia, Pennsylvania, USA Number of enrolled participants: 43 children Number (%) of enrolled participants completing the study: 41 (98%) Study completers ‐ mean age (SD): not reported Study completers ‐ sex: male (39%) and female (61%) Study completers ‐ mean BMI kg/m^2^ (SD): 45% overweight or obese (neither BMI z score nor BMI percentile were reported). Specific social or cultural characteristics: Participants in the US National School Lunch Program Socio‐economic status context: high deprivation Inclusion criteria: child in first‐grade (USA); participating in the US Department of Agriculture National School Lunch Program (NSLP) Exclusion criteria: parental report of a chronic medical condition or medication use affecting food intake; reported allergies to foods on the experimental menu	
Interventions	Manipulated product type: food Manipulation: tableware size Duration of exposure to intervention: ≤ 1 day Social setting: selecting and consuming with others Study arms: child size dishware (7.25 inch diameter plate with a surface area of 41.26 inches^2^ and an 8 ounce bowl); adult size dishware (10.25 inch diameter plate with a surface area of 82.47 inches^2^ and a 16 ounce bowl) Number of comparisons analysed: 1 Comparisons analysed: Intervention 1: child size dishware (7.25 inch diameter plate with a surface area of 41.26 inches^2^ and an 8 ounce bowl); *versus* Intervention 2: adult size dishware (10.25 inch diameter plate with a surface area of 82.47 inches^2^ and a 16 ounce bowl) Concurrent intervention components: no	
Outcomes	Outcomes reported in study: total energy self served at lunch meal (kcal); energy self served from unit (chicken nuggets) entrée (kcal); energy self served from amorphous (penne with meat sauce) entrée (kcal); energy self served from vegetable side dish (kcal); energy self served from fruit side dish (kcal); energy intake from total lunch meal (kcal); energy intake from unit (chicken nuggets) entrée (kcal); energy intake from amorphous (penne with meat sauce) entrée (kcal); energy intake from vegetable side dish (kcal); energy intake from fruit side dish (kcal) Selection outcome analysed: total energy self served at lunch meal (kcal) Measurement of selection outcome: objective Timing of selection outcome measurement: immediate (≤ 1 day) Consumption outcome analysed: energy intake from total lunch meal (kcal) Measurement of consumption outcome: objective Timing of consumption outcome measurement: immediate (≤ 1 day)	
Funding source	US Department of Agriculture National Research Initiative (USDA NRI 2006‐55215‐05938)	
Notes	Author contacted to request information missing from the study report ‐ requested information was supplied (February 2014)	
***Risk of bias***		
**Bias**	**Authors' judgement**	**Support for judgement**
Random sequence generation (selection bias)	Unclear risk	Comment: method of sequence generation for condition order is not described. Insufficient information about the sequence generation process to permit judgement of 'low risk' or 'high risk'
Allocation concealment (selection bias)	Low risk	Comment: participating classrooms appear to have been randomised to condition order concurrently, after consent for individuals' participation had been obtained. The review authors therefore judge that any lack of concealment of allocation sequence is unlikely to be an issue for risk of bias
Blinding of participants and personnel (performance bias) Selection outcome	Unclear risk	Comment: no blinding or incomplete blinding of study participants. Not reported whether participants were probed for suspicion of study purpose or awareness of size manipulation between study conditions. It is possible that the outcome may be influenced by lack of blinding of study participants (due to potential carry‐over effects between conditions). Very unlikely that key study personnel were blinded, but the review authors judge that the outcome is not likely to be influenced by lack of blinding of key study personnel
Blinding of participants and personnel (performance bias) Consumption outcome	Unclear risk	Comment: no blinding or incomplete blinding of study participants. Not reported whether participants were probed for suspicion of study purpose or awareness of size manipulation between study conditions. It is possible that the outcome may be influenced by lack of blinding of study participants (due to potential carry‐over effects between conditions). Very unlikely that key study personnel were blinded, but the review authors judge that the outcome is not likely to be influenced by lack of blinding of key study personnel
Blinding of outcome assessment (detection bias) Selection outcome	Low risk	Comment: no blinding of outcome assessment, but the review authors judge that the outcome measurement is not likely to be influenced by lack of blinding
Blinding of outcome assessment (detection bias) Consumption outcome	Low risk	Comment: no blinding of outcome assessment, but the review authors judge that the outcome measurement is not likely to be influenced by lack of blinding
Incomplete outcome data (attrition bias) Selection outcome	Low risk	Quote: "Of the 43 child participants, 1 left the school and did not complete the study." Comment: reason for missing outcome data is unlikely to be related to selection outcome
Incomplete outcome data (attrition bias) Consumption outcome	Low risk	Quote: "Of the 43 child participants, 1 left the school and did not complete the study." Comment: reason for missing outcome data is unlikely to be related to consumption outcome
Selective reporting (reporting bias)	Unclear risk	Comment: search for record(s) containing details of study protocol conducted in ClinicalTrials.gov and the WHO International Clinical Trials Registry Platform (ICTRP). No records found. Insufficient information to permit judgement of 'low risk' or 'high risk'
Other bias #1 ‐ Baseline comparability of participant characteristics between groups	Unclear risk	Comment: study uses a within‐subjects design. No measurement of participant pre‐condition 'state' characteristics is reported. No analysis of potential differences in measured outcomes between condition orders appears to have been conducted and the statistical analysis of outcome data does not appear to control for condition order. Risk of bias due to period effects is therefore unclear. Insufficient information to permit judgement of 'low risk' or 'high risk'
Other bias #2 ‐ Consistency in intervention delivery	Unclear risk	Quote: "[In each study condition] Children were told that they could make 1 trip through the buffet line, that they could serve themselves and eat as much or as little as they wanted, and they were not allowed to share food with other children. Children ate at their desks in their classrooms during a 15‐minute timed meal. Research assistants were present to ensure that foods were not shared and to note any spilled or dropped foods." Comment: information and instructions provided to participants appear to have been standardised between the compared study conditions. No information pertaining to monitoring of participants' compliance with the instruction to make 1 trip through the buffet line is reported. Participants' compliance with the instruction to not share food with other children was monitored by research assistants present for the duration of each timed meal; however, no monitoring results are reported with respect to this instruction. No further specific instructions were provided to participants, other than the instruction that participants could serve themselves and eat as much or as little as they wanted
Summary of risk of bias Selection outcome	Unclear risk	Unclear risk
Summary of risk of bias Consumption outcome	Unclear risk	Unclear risk

**Table:** Ebbeling 2007

Methods	Study design: within‐subjects randomised controlled trial	
Participants	Setting: field setting, national fast food chain in a food court Geographical region: Boston, Massachusetts, USA Number of enrolled participants: 20 adolescents Number (%) of enrolled participants completing the study: 18 (90%) Study completers ‐ mean age (SD): 15.3 (1.3) Study completers ‐ sex: male (22%) and female (78%) Study completers ‐ mean BMI kg/m^2^ (SD): 93.9 (5.9) (BMI percentile) Specific social or cultural characteristics: none Socio‐economic status context: low deprivation Inclusion criteria: aged between 13 and 17 years; self reported consumer of fast food at least once per week; BMI values exceeding gender and age‐specific 80th percentile values Exclusion criteria: self reported diagnosis of major medical illness; self reported diagnosis of eating disorder; self reported smoking ≥ 1 cigarette in the past week; self report taking any prescription medication that may affect food intake	
Interventions	Manipulated product type: food Manipulation: package size Duration of exposure to intervention: ≤ 1 day Social setting: consuming with others Study arms: fast food meal presented as 1 large serving (on a tray) at a single time point; fast food meal presented as portioned into 4 smaller servings (divided equally among a tray and 3 lunch boxes) presented at a single time point; fast food meal presented as portioned into 4 smaller servings (divided equally among a tray and 3 lunch boxes) presented at 15‐minute intervals (with the tray being delivered at time 0 and the boxes being delivered at regular intervals ‐ 15 min, 30 min and 45 min) Number of comparisons analysed: 1 Comparisons analysed: Intervention 1: fast food meal presented as portioned into 4 smaller servings (divided equally among a tray and 3 lunch boxes) presented at a single time point; *versus* Intervention 2: fast food meal presented as 1 large serving (on a tray) at a single time point Concurrent intervention components: no	
Outcomes	Outcomes reported in study: energy intake from total meal (kilojoules); amount of food consumed from total meal (grams); energy intake from total meal, as a proportion of total one day energy expenditure (%) Selection outcome analysed: N/A Measurement of selection outcome: N/A Timing of selection outcome measurement: N/A Consumption outcome analysed: energy intake from total meal (kilojoules) Measurement of consumption outcome: objective Timing of consumption outcome measurement: immediate (≤ 1 day)	
Funding source	US National Institutes of Health (Grant P30 DK40561); Charles H. Hood Foundation; National Institutes of Health (Grant M01 RR02172); National Institute of Diabetes and Digestive and Kidney Diseases (Grant R01 DK59240)	
Notes	—	
***Risk of bias***		
**Bias**	**Authors' judgement**	**Support for judgement**
Random sequence generation (selection bias)	Low risk	Quote: "By using a crossover design for visits 2 to 4, we assigned each subject randomly to 1 of 6 possible sequences of 3 feeding conditions. The random assignment was stratified according to gender. Identification numbers for male participants were matched randomly to a single block of 12 assignments (i.e. with each possible feeding sequence represented twice) and those for female participants to 2 blocks of 12 and 6 assignments."
Allocation concealment (selection bias)	Low risk	Quote: "The assignments were prepared on index cards by the study statistician and were delivered in opaque envelopes to the principal investigator, to be opened after each participant's baseline assessment visit."
Blinding of participants and personnel (performance bias) Consumption outcome	Unclear risk	Quote: "At the time of recruitment...We did not mention strategies for altering portion sizes and eating rate." Comment: no blinding or incomplete blinding of study participants. Not reported whether participants were probed for suspicion of study purpose or awareness of size manipulation between study conditions. It is possible that the outcome may be influenced by lack of blinding of study participants (due to potential carry‐over effects between conditions). Very unlikely that key study personnel were blinded, but the review authors judge that the outcome is not likely to be influenced by lack of blinding of key study personnel
Blinding of outcome assessment (detection bias) Consumption outcome	Low risk	Comment: no blinding of outcome assessment, but the review authors judge that the outcome measurement is not likely to be influenced by lack of blinding
Incomplete outcome data (attrition bias) Consumption outcome	Low risk	Quote: "Eighteen of the 20 subjects (4 male subjects and 14 female subjects) enrolled in the study completed all of the study visits." Comment: no reasons for participants not completing all study visits provided. The low proportion (two participants, 10% of study sample) of exclusions means that the review authors judge that the plausible effect size among missing outcomes is unlikely to be enough to have an important impact on the observed effect size
Selective reporting (reporting bias)	Low risk	Comment: search for record(s) containing details of study protocol conducted in ClinicalTrials.gov and the WHO International Clinical Trials Registry Platform (ICTRP). Record found in ClinicalTrials.gov (Identifier: NCT00121706). Comparison of ClinicalTrials.gov record with published study report indicates no selective outcome reporting
Other bias #1 ‐ Baseline comparability of participant characteristics between groups	Low risk	Quote: "Before each meal, we asked each subject to rate his or her level of hunger by using a 10‐cm visual analog scale, anchored with the descriptors "not at all hungry" and "extremely hungry."... The analysis of variance included a fixed effect to test for systematic variation across the 3 successive visits (order effects) and an interaction term to test whether differences among feeding conditions depended on the position in the sequence (effect modification)...Position in the visit sequence had no systematic effect on intake...and there was no significant interaction between feeding condition and visit number...[Ratings] of hunger... did not differ across conditions." Comment: study uses a within‐subjects design. No difference between conditions in terms of measured pre‐condition participant 'state' characteristic, but not reported whether there were differences between condition orders in terms of measured pre‐condition participant 'state' characteristics. However, a statistical analysis was conducted to test for the potential influence of condition order on measured outcomes and no influence was observed. It is therefore unlikely that any differences between condition orders in terms of measured pre‐condition participant 'state' characteristics influenced the measured outcomes. Risk of bias due to period effects is therefore judged low
Other bias #2 ‐ Consistency in intervention delivery	Unclear risk	Quote: "We instructed subjects to eat a standard breakfast of cold cereal and milk at 9:00 AM on the day of each visit and then not to eat or to drink anything, except water, until after the visit... The following standard instructions were read to the group of subjects before the meal: "We will bring each of you a meal. Eat as much or as little as you like, until you have had enough. There is more food available, and you may eat as much as you want. Please do not share your food with others in the group. If you need more of anything, just ask. Keep your packaging on your tray." Research staff members monitored food intake discreetly... We collected dietary and physical activity data during telephone‐administered, 24‐hour recall interviews, by calling each subject on the 2 days after each of the 3 test visits to assess behaviors during the day of the visit and the day after the visit." Comment: information and instructions to participants appear to have been standardised between the compared study conditions. Whilst not explicitly stated, it is likely that participants' compliance with the instruction not to share food with others in the group was monitored by research staff present for the duration of each study visit; however no monitoring results are reported with respect to this instruction. Monitoring of compliance with the instruction regarding eating prior to each study visit appears to have been encompassed in the telephone‐administered interview that assessed dietary behaviour during the day of the visit and the day after the visit; however no monitoring results are reported with respect to this instruction. No other specific instructions were provided to participants, other than the instruction that they may eat as much as they want
Summary of risk of bias Consumption outcome	Unclear risk	Unclear risk

**Table:** Fisher 2003

Methods	Study design: within‐subjects randomised controlled trial	
Participants	Setting: laboratory setting Geographical region: Pennsylvania State University, University Park, Pennsylvania, USA Number of enrolled participants: 35 children Number (%) of enrolled participants completing the study: 35 (100%) Study completers ‐ mean age (SD): 4.0 (0.5) Study completers ‐ sex: male (49%) and female (51%) Study completers ‐ mean BMI kg/m^2^ (SD): not reported (neither BMI z score nor BMI percentile) Specific social or cultural characteristics: parents tended to be highly educated and currently employed: 81% of mothers and 90% of fathers reported having a 4‐y university degree, and 84% of mothers and 90% of fathers reported current employment. Most of the families (68%) reported combined family incomes of > USD 50,000 Socio‐economic status context: low deprivation Inclusion criteria: pre‐school child attending full‐day day care programmes at The Pennsylvania State University Exclusion criteria: none reported	
Interventions	Manipulated product type: food. Manipulation: portion size Duration of exposure to intervention: > 1 day Social setting: selecting and consuming with others Study arms: age‐appropriate size reference portion of macaroni and cheese entrée (125 g for younger children; 175 g for older children); large size portion of macaroni and cheese entrée (250 g for younger children; 350 g for older children) Number of comparisons analysed: 1 Comparisons analysed: Intervention 1: age‐appropriate size reference portion of macaroni and cheese entrée (125 g for younger children; 175 g for older children); *versus* Intervention 2: large size portion of macaroni and cheese entrée (250 g for younger children; 350 g for older children) Concurrent intervention components: no	
Outcomes	Outcomes reported in study: average (mean) amount of entrée self served at 2 lunches during weeks following reference/large sized meal weeks (grams); average (mean) energy intake from lunch meal (kilojoules); average (mean) energy intake from entrée (kilojoules); average (mean) number of bites from entrée (N); average (mean) bite size from entrée (grams per bite) Selection outcome analysed: average (mean) amount of entrée self served at 2 lunches during weeks following reference/large sized meal weeks (grams) Measurement of selection outcome: objective Timing of selection outcome measurement: longer‐term (> 1 day) Consumption outcome analysed: average (mean) energy intake from lunch meals (kilojoules) Measurement of consumption outcome: objective Timing of consumption outcome measurement: immediate (≤ 1 day)	
Funding source	US Department of Agriculture Grant (NRI 00001322)	
Notes	—	
***Risk of bias***		
**Bias**	**Authors' judgement**	**Support for judgement**
Random sequence generation (selection bias)	Unclear risk	Quote: "The order in which the children received the reference and large portions was balanced for age and sex." Comment: author contact confirmed condition order was randomised but no further details (13/3/13)
Allocation concealment (selection bias)	Unclear risk	Comment: method of concealment is not described. Insufficient information to permit judgement of 'low risk' or 'high risk'
Blinding of participants and personnel (performance bias) Selection outcome	Unclear risk	Quote: "To decrease visual comparisons of portion size by children receiving different portion sizes, a portable room divider was used to separate the tables... The children's comments about portion size during the lunches were recorded at one‐half of both the reference‐portion and the large‐portion lunch sessions. A staff member sat with each table of 4–5 children. The frequency of any evaluative comments regarding the size of the main entrée as being "small," "okay," or "big" was tallied. Coders were trained by using written descriptions and examples of comments to be coded in each category. Any questionable comment was recorded verbatim and coded at the end of the session... The children's comments about portion size were measured to determine the extent to which any changes in intake might reflect changes in awareness of portion size. Few comments were made regarding portion size throughout the experiment. During 2 reference‐portion lunches and 2 large‐portion lunches at which behavioral observations were made, none of the children described the portion sizes as "small" or "okay." The reference portion size was described as being "big" by 1 child during a reference‐portion lunch, and the large portion size was described as being "big" by 6 children during the large‐portion lunches... In the present study, few children made comments about portion size, and the children's self‐selected portions of the entrée did not change with repeated exposure to large portions. It is possible that changes in portion size may have been visually difficult to discern because of the use of an amorphous entrée. In any case, these findings indicate that increases in children's entrée bite size and intake occurred without appreciable awareness of changes in portion size." Comment: blinding of study participants attempted but likely that blinding was broken in some cases and it is possible that the outcome may be influenced by lack of blinding (due to potential carry‐over effects between conditions). Very unlikely that key study personnel were blinded, but the review authors judge that the outcome is not likely to be influenced by lack of blinding of key study personnel
Blinding of participants and personnel (performance bias) Consumption outcome	Unclear risk	Quote: "To decrease visual comparisons of portion size by children receiving different portion sizes, a portable room divider was used to separate the tables... The children's comments about portion size during the lunches were recorded at one‐half of both the reference‐portion and the large‐portion lunch sessions. A staff member sat with each table of 4–5 children. The frequency of any evaluative comments regarding the size of the main entrée as being "small," "okay," or "big" was tallied. Coders were trained by using written descriptions and examples of comments to be coded in each category. Any questionable comment was recorded verbatim and coded at the end of the session... The children's comments about portion size were measured to determine the extent to which any changes in intake might reflect changes in awareness of portion size. Few comments were made regarding portion size throughout the experiment. During 2 reference‐portion lunches and 2 large‐portion lunches at which behavioral observations were made, none of the children described the portion sizes as "small" or "okay." The reference portion size was described as being "big" by 1 child during a reference‐portion lunch, and the large portion size was described as being "big" by 6 children during the large‐portion lunches... In the present study, few children made comments about portion size, and the children's self‐selected portions of the entrée did not change with repeated exposure to large portions. It is possible that changes in portion size may have been visually difficult to discern because of the use of an amorphous entrée. In any case, these findings indicate that increases in children's entrée bite size and intake occurred without appreciable awareness of changes in portion size." Comment: blinding of study participants attempted but likely that blinding was broken in some cases and it is possible that the outcome may be influenced by lack of blinding (due to potential carry‐over effects between conditions). Very unlikely that key study personnel were blinded, but the review authors judge that the outcome is not likely to be influenced by lack of blinding of key study personnel
Blinding of outcome assessment (detection bias) Selection outcome	Low risk	Comment: no blinding of outcome assessment, but the review authors judge that the outcome measurement is not likely to be influenced by lack of blinding
Blinding of outcome assessment (detection bias) Consumption outcome	Low risk	Comment: no blinding of outcome assessment, but the review authors judge that the outcome measurement is not likely to be influenced by lack of blinding
Incomplete outcome data (attrition bias) Selection outcome	High risk	Quote: "Data are reported for 30 of the 35 children; the data from 5 children were excluded from analyses because their mean intake of the main entrée was < 10 g across the 4 lunches in which the reference portion was served. The children whose data were excluded were not significantly different from all others in terms of age (P = 0.74) or body mass index (BMI)–for–age z score (P = 0.44). Missing data or children identified as outliers (> 2 SDs) are reflected in the sample size for each change variable." Comment: the reason for missing outcome data for selection outcome is the study authors' decision to exclude participants with < 10 g consumption across the 4 lunches in which the reference portion was served and outliers (> 2 standard deviations from mean consumption) from the analysis. The substantial proportion (6 participants, 17% of study sample) of exclusions due to low consumption and outliers means that the review authors judge that it is plausible that the effect size among these missing data is enough to have had an important impact on the observed effect size
Incomplete outcome data (attrition bias) Consumption outcome	High risk	Quote: "Data are reported for 30 of the 35 children; the data from 5 children were excluded from analyses because their mean intake of the main entrée was < 10 g across the 4 lunches in which the reference portion was served. The children whose data were excluded were not significantly different from all others in terms of age (P = 0.74) or body mass index (BMI)–for–age z score (P = 0.44)." Comment: the reason for missing outcome data for consumption outcome is the study authors' decision to exclude participants with < 10 g consumption across the 4 lunches in which the reference portion was served from the analysis. The substantial proportion (5 participants, 14% of study sample) of exclusions due to low consumption means that the review authors judge that it is plausible that the effect size among these missing data is enough to have had an important impact on the observed effect size
Selective reporting (reporting bias)	Unclear risk	Comment: search for record(s) containing details of study protocol conducted in ClinicalTrials.gov and the WHO International Clinical Trials Registry Platform (ICTRP). No records found. Insufficient information to permit judgement of 'low risk' or 'high risk'
Other bias #1 ‐ Baseline comparability of participant characteristics between groups	Low risk	Comment: study uses a within‐subjects design. No measurement of participant pre‐condition 'state' characteristics is reported. However, the statistical analysis appears to control for the potential influence of condition order on measured outcomes. It is therefore unlikely that any differences between condition orders in terms of unmeasured pre‐condition participant 'state' characteristics influenced the measured outcomes. Risk of bias due to period effects is therefore judged low
Other bias #2 ‐ Consistency in intervention delivery	Unclear risk	Quote: "The children were instructed not to share any foods, to eat as much or as little as they desired, and to remain seated for the duration of the lunch period...A staff member [behavioural coder] sat with each table of 4–5 children." Comment: information and instructions to participants appear to have been standardised between the compared study conditions. Whilst not explicitly reported, it is likely that participants' compliance with the instructions not to share any foods and to remain seated for the duration of the lunch period was monitored by a behavioural coder seated with each group of participants for the duration of each study session; however, no monitoring results are reported with respect to these instructions. No other specific instructions were provided to participants, other than the instruction to eat as much or as little as they desired
Summary of risk of bias Selection outcome	Unclear risk	Unclear risk
Summary of risk of bias Consumption outcome	Unclear risk	Unclear risk

**Table:** Fisher 2007a

Methods	Study design: within‐subjects randomised controlled trial	
Participants	Setting: laboratory setting Geographical region: Head Start Programs in the greater metropolitan area of Houston, TX, USA Number of enrolled participants: 59 children and their 59 mothers Number (%) of enrolled participants completing the study: children = 58 (98%); mothers = 58 (98%) Study completers ‐ mean age (SD): children = 5.0 (missing); mothers = 30.0 (5.0) Study completers ‐ sex: male (40%) and female (60%) children and their mothers Study completers ‐ mean BMI kg/m^2^ (SD): children = 60.0 (29.0) (BMI percentile); mothers = 34.0 (9.0) Specific social or cultural characteristics: low‐income Hispanic and African American children and their mothers Socio‐economic status context: high deprivation Inclusion criteria: attending Head Start Program in the greater metropolitan area of Houston, TX, USA; 5‐year old child; Hispanic or non‐Hispanic African American ethnicity Exclusion criteria: presence of severe food allergies or chronic illnesses affecting food intake (child or mother); dislike of ≥ 2 of the foods for which portion size was manipulated (child or mother); self reported previous diagnosis of maternal depression (mother) or eating disorders (child or mother)	
Interventions	Manipulated product type: food Manipulation: portion size Duration of exposure to intervention: ≤ 1 day Social setting: consuming with others Study arms: reference size portions; large size portions Number of comparisons analysed: 2 (children =1; mothers =1) Comparisons analysed: children = Intervention 1: reference portions of macaroni and cheese (453 kcal) at lunch, apple juice (113 kcal) and Graham crackers (185 kcal) at afternoon snack, chicken nuggets (368 kcal) at dinner, Oat ring cereal (160 kcal) at breakfast; *versus* Intervention 2: large portions of macaroni and cheese (906 kcal) at lunch, apple juice (226 kcal) and Graham crackers (370 kcal) at afternoon snack, chicken nuggets (736 kcal) at dinner, Oat ring cereal (320 kcal) at breakfast. Mothers = Intervention 1: reference portions of macaroni and cheese (604 kcal) at lunch, apple juice (158 kcal) and Graham crackers (277 kcal) at afternoon snack, chicken strips (346 kcal) and rice (160 kcal) at dinner, Oat ring cereal (320 kcal) at breakfast; *versus* Intervention 2: large portions of macaroni and cheese (1208 kcal) at lunch, apple juice (316 kcal) and Graham crackers (544 kcal) at afternoon snack, chicken strips (692 kcal) and rice (320 kcal) at dinner, Oat ring cereal (640 kcal) at breakfast Concurrent intervention components: no	
Outcomes	Outcomes reported in study: children and mothers: total daily energy intake (kcal); total daily energy intake from all portion‐manipulated foods (kcal); total daily energy intake from all other (non‐portion‐manipulated) foods (kcal);energy intake from (non‐portion‐manipulated) foods at morning snack (kcal); energy intake from (portion‐manipulated) macaroni and cheese at lunch (kcal); energy intake from other (non‐portion‐manipulated) foods at lunch (kcal); energy intake from (portion‐manipulated) apple juice at afternoon snack (kcal); energy intake from (portion‐manipulated) Graham crackers at afternoon snack (kcal); energy intake from (portion‐manipulated) chicken strips at dinner (kcal); energy intake from (portion‐manipulated) rice at dinner (kcal); energy intake from other (non‐portion‐manipulated) foods at dinner (kcal); energy intake from (non‐portion‐manipulated) foods at evening snack (kcal); energy intake from (portion‐manipulated) Oat ring cereal at breakfast (kcal); energy intake from other (non‐portion‐manipulated) foods at breakfast (kcal) Selection outcome analysed: N/A Measurement of selection outcome: N/A Timing of selection outcome measurement: N/A Consumption outcome analysed: total daily energy intake (kcal) Measurement of consumption outcome: objective Timing of consumption outcome measurement: immediate (≤ 1 day)	
Funding source	US Department of Agriculture CRIS funds and the National Research Initiative of the US Department of Agriculture Cooperative State Research, Education and Extension Service (Grant number 2002‐35200‐12264)	
Notes	Outcome data for children and mothers analysed separately (one comparison each) because the absolute difference in portion size between reference size and large size portion conditions varied between children and mothers	
***Risk of bias***		
**Bias**	**Authors' judgement**	**Support for judgement**
Random sequence generation (selection bias)	Unclear risk	Comment: method of sequence generation for condition order is not described. Insufficient information about the sequence generation process to permit judgement of 'low risk' or 'high risk'
Allocation concealment (selection bias)	Unclear risk	Comment: method of concealment is not described. Insufficient information to permit judgement of 'low risk' or 'high risk'
Blinding of participants and personnel (performance bias) Consumption outcome	Unclear risk	Quote: "The mothers were told that the purpose of the study was to evaluate their children's food preferences and intake patterns and that their own intake patterns would be measured to provide background information. Data collected at the end of the study indicate that mothers generally perceived the child to be the focus of study: less than half of the mothers (28 of 59) made reference to their own eating in describing the study purpose (ie, "to study the eating patterns of children of different ethnicity"), and almost one‐third (9 of 28) of those who did believed the study to involve parent‐child similarities in food preference (ie, "to observe food preference in children in comparison to the mothers"). The staff did not inform the participating children that their food intakes were being measured." Comment: no blinding or incomplete blinding of study participants and it is possible that the outcome may be influenced by lack of blinding (due to potential carry‐over effects between conditions). Very unlikely that key study personnel were blinded, but the review authors judge that the outcome is not likely to be influenced by lack of blinding of key study personnel
Blinding of outcome assessment (detection bias) Consumption outcome	Low risk	Comment: no blinding of outcome assessment, but the review authors judge that the outcome measurement is not likely to be influenced by lack of blinding
Incomplete outcome data (attrition bias) Consumption outcome	Low risk	Quote: "Data from one mother‐child pair were excluded from the analyses because the child complained of a toothache and was observed to have a loose tooth for the duration of one of the visits. Data from 58 children and 58 mothers were analyzed." Comment: reason for missing outcome data is likely to be related to consumption outcome but inclusion could plausibly have biased the estimate of the effect of the intervention on consumption. The review authors judge that the decision to exclude this participant is reasonable, as it is likely to protect against bias in the estimate of the effect of the intervention on consumption
Selective reporting (reporting bias)	Unclear risk	Comment: search for record(s) containing details of study protocol conducted in ClinicalTrials.gov and the WHO International Clinical Trials Registry Platform (ICTRP). No records found. Insufficient information to permit judgement of 'low risk' or 'high risk'
Other bias #1 ‐ Baseline comparability of participant characteristics between groups	Low risk	Quote: "Potential correlates of changes in food and total energy intake were tested cojointly by analysis of variance: sex, ethnicity, condition order, BMI (z scores used for children), and food insecurity." Comment: study uses a within‐subjects design. No measurement of participant pre‐condition 'state' characteristics is reported. However, the statistical analysis appears to control for the potential influence of condition order on measured outcomes. It is therefore unlikely that any differences between condition orders in terms of unmeasured pre‐condition participant 'state' characteristics influenced the measured outcomes. Risk of bias due to period effects is therefore judged low
Other bias #2 ‐ Consistency in intervention delivery	Low risk	Children: Quote: "Three to 4 children who did not know one another were seated together with a research staff member who facilitated non‐food related conversation, ensured that foods were not shared, and accounted for dropped or spilled food. Participants were informed that they could eat as much or as little as desired during each meal and snack." Comment: information and instructions to participants appear to have been standardised between the compared study conditions. Participants' compliance with the instruction not to share food was monitored by a member of research staff seated with children for the duration of meals during each 24‐h study visit; it is explicitly stated that the member of research staff ensured participants were compliant with this instruction. No other specific instructions were provided to participants, other than the instruction that they could eat as much or as little as desired during each meal and snack Mothers: Quote: "Participants were informed that they could eat as much or as little as desired during each meal and snack." Comment: information and instructions provided to participants appear to have been standardised between the compared study conditions. No specific instructions were provided to participants, other than the instruction to eat as much or as little as desired, and therefore monitoring of participants' compliance with instructions is not applicable
Summary of risk of bias Consumption outcome	Unclear risk	Unclear risk

**Table:** Fisher 2007b

Methods	Study design: within‐subjects cluster‐randomised controlled trial Unit of allocation: group Unit of analysis: individual Number of clusters: not reported Number of participants per cluster: 3 to 4 Analysis does not appear to account for cluster allocation, as the statistical model does not appear to include any covariate related to cluster assignment	
Participants	Setting: laboratory setting Geographical region: greater metropolitan area of Houston, Texas, USA Number of enrolled participants: 53 children Number (%) of enrolled participants completing the study: 53 (100%) Study completers ‐ mean age (SD): not reported Study completers ‐ sex: male (47%) and female (53%) Study completers ‐ mean BMI kg/m^2^ (SD): 0.45 (1.08) (BMI z score); 61.4 (28.4) (BMI percentile) Specific social or cultural characteristics: none Socio‐economic status context: low deprivation Inclusion criteria: aged between 5 and 6 years Exclusion criteria: presence of chronic medical conditions or medication affecting food intake; food allergies; BMI for age < 5th percentile; dislike of the study entrée	
Interventions	Manipulated product type: food Manipulation: portion size Duration of exposure to intervention: ≤ 1 day Social setting: consuming with others Study arms: small portion size macaroni and cheese entrée (250 g), low energy density; small portion size macaroni and cheese entrée (250 g), high energy density; large portion size macaroni and cheese entrée (500 g), low energy density; large portion size macaroni and cheese entrée (500 g), high energy density Number of comparisons analysed: 1 Comparisons analysed: Intervention 1: small portion size macaroni and cheese entrée (250 g); *versus* Intervention 2: large portion size macaroni and cheese entrée (500 g) Concurrent intervention components: no	
Outcomes	Outcomes reported in study: energy intake from total dinner meal (kcal); energy intake from macaroni and cheese entrée (kcal); amount of macaroni and cheese entrée consumed (grams); energy intake from other (non‐entrée) meal components (foods) (kcal); amount of other (non‐entrée) meal components (foods) consumed Selection outcome analysed: N/A Measurement of selection outcome: N/A Timing of selection outcome measurement: N/A Consumption outcome analysed: energy intake from total dinner meal (kcal) Measurement of consumption outcome: objective Timing of consumption outcome measurement: immediate (≤ 1 day)	
Funding source	National Institutes of Health (Grant R01 DK071095); US Department of Agriculture CRIS funds; Baylor College of Medicine General Clinical Research Center	
Notes	Outcome data for low energy density and high energy density participant subgroups collapsed and analysed together (one comparison)	
***Risk of bias***		
**Bias**	**Authors' judgement**	**Support for judgement**
Random sequence generation (selection bias)	Unclear risk	Comment: method of sequence generation for condition order is not described. Insufficient information about the sequence generation process to permit judgement of 'low risk' or 'high risk'
Allocation concealment (selection bias)	Unclear risk	Comment: method of concealment is not described. Insufficient information to permit judgement of 'low risk' or 'high risk'
Blinding of participants and personnel (performance bias) Consumption outcome	Unclear risk	Quote: "To minimize visual comparisons of portion sizes, each child was assigned to eat with children in the same portion size condition." Comment: blinding of study participants attempted but it is possible that blinding was broken in some cases. Not reported whether participants were probed for suspicion of study purpose or awareness of size manipulation between study conditions. It is possible that the outcome may be influenced by lack of blinding of study participants (due to potential carry‐over effects between conditions). Very unlikely that key study personnel were blinded, but the review authors judge that the outcome is not likely to be influenced by lack of blinding of key study personnel
Blinding of outcome assessment (detection bias) Consumption outcome	Low risk	Comment: no blinding of outcome assessment, but the review authors judge that the outcome measurement is not likely to be influenced by lack of blinding
Incomplete outcome data (attrition bias) Consumption outcome	Low risk	Comment: no missing outcome data for consumption outcome
Selective reporting (reporting bias)	Low risk	Comment: search for record(s) containing details of study protocol conducted in ClinicalTrials.gov and the WHO International Clinical Trials Registry Platform (ICTRP). Record found in ClinicalTrials.gov (Identifier: NCT00436878; Experiment 3). Comparison of ClinicalTrials.gov record with published study report indicates no selective outcome reporting
Other bias #1 ‐ Baseline comparability of participant characteristics between groups	Unclear risk	Comment: study uses a within‐subjects design. No measurement of participant pre‐condition 'state' characteristics is reported. No analysis of potential differences in measured outcomes between condition orders appears to have been conducted and the statistical analysis of outcome data does not appear to control for condition order. Risk of bias due to period effects is therefore unclear. Insufficient information to permit judgement of 'low risk' or 'high risk'
Other bias #2 ‐ Consistency in intervention delivery	Unclear risk	Quote: "Parents were instructed to refrain from giving their child any foods or beverages 2 hours before the visit. On arrival, a research member interviewed the parent to confirm that those instructions had been followed... At all visits, 3 to 4 children were served dinner together in the presence of a research staff member. The group of children to which each child was assigned and the staff member to whom each group was assigned did not vary across visits. Children were instructed not to share food and to eat as little or as much as desired during the 20‐min timed dinner." Comment: information and instructions provided to participants appear to have been standardised between the compared study conditions. Compliance with the instruction for parents to refrain from giving their child any foods or beverages for 2 hours before each study visit was monitored via parent interview; however no monitoring results are reported with respect to this instruction. Although not explicitly stated, it is likely that compliance with the instruction for children not to share food was monitored by the research staff member present with each group of children for the duration of each dinner visit; however no monitoring results are reported with respect to this instruction. No further specific instructions were provided to participants, other than the instruction to eat as little or as much as desired during each 20‐min timed dinner
Summary of risk of bias Consumption outcome	Unclear risk	Unclear risk

**Table:** Fisher 2007c

Methods	Study design: within‐subjects randomised controlled trial	
Participants	Setting: laboratory setting Geographical region: greater metropolitan area of Houston, TX, USA Number of enrolled participants: 25 children aged 2 to 3 years; 25 children aged 5 to 6 years; 25 children aged 8 to 9 years Number (%) of enrolled participants completing the study: children aged 2 to 3 years = 25 (100%); children aged 5 to 6 years = 25 (100%); children aged 8 to 9 years = 25 (100%) Study completers ‐ mean age (SD): children aged 2 to 3 years = 2.6 (0.5); children aged 5 to 6 years = 5.6 (0.5); children aged 8 to 9 years = 8.7 (0.4) Study completers ‐ sex: children aged 2 to 3 years = male (68%) and female (32%); children aged 5 to 6 years = male (68%) and female (32%); children aged 8 to 9 years = male (40%) and female (60%) Study completers ‐ mean BMI kg/m^2^ (SD): children aged 2 to 3 years = 76.0 (33.0) (BMI percentile); children aged 5 to 6 years = 61.0 (31.0) (BMI percentile); children aged 8 to 9 years = 75.0 (25.0) (BMI percentile) Specific social or cultural characteristics: non‐Hispanic White children Socio‐economic status context: low deprivation Inclusion criteria: aged 2 to 3, 5 to 6, or 8 to 9 years; non‐Hispanic white ethnicity Exclusion criteria: presence of chronic medical conditions or medication affecting food intake; food allergies; BMI for age < 5th percentile; dislike of ≥ 2 foods on the experimental menu	
Interventions	Manipulated product type: food Manipulation: portion size Duration of exposure to intervention: ≤ 1 day Social setting: consuming with others Study arms: small portion size; large portion size; large portion size self served from an individual serving dish Number of comparisons analysed: 3 (children aged 2 to 3 years = 1; children aged 5 to 6 years = 1; children aged 8 to 9 years = 1) Comparisons analysed: children aged 2 to 3 years = Intervention 1: small size portion (200 g) macaroni and cheese entrée; *versus* Intervention 2: large size portion (400 g) macaroni and cheese entrée; children aged 5 to 6 years = Intervention 1: small size portion (250 g) macaroni and cheese entrée; *versus* Intervention2: large size portion (500 g) macaroni and cheese entrée; children aged 8 to 9 years = Intervention 1: small size portion (450 g) macaroni and cheese entrée; *versus* Intervention 2: large size portion (900 g) macaroni and cheese entrée Concurrent intervention components: no	
Outcomes	Outcomes reported in study: all age groups: energy intake from total dinner meal (kcal); energy intake from macaroni and cheese entrée (kcal); energy intake from other (non‐entrée) dinner meal components (foods) (kcal); bite frequency from total dinner meal (n); average (mean) bite size from total dinner meal (grams per bite) Selection outcome analysed: N/A Measurement of selection outcome: N/A Timing of selection outcome measurement: N/A Consumption outcome analysed: energy intake from total dinner meal (kcal) Measurement of consumption outcome: objective Timing of consumption outcome measurement: immediate (≤ 1 day)	
Funding source	North American International Life Sciences Association Committee on Lifestyle and Weight Management	
Notes	Outcome data for children aged 2 to 3 years, children aged 5 to 6 years and children aged 8 to 9 years analysed separately (one comparison each) because the absolute difference in portion size between reference size and large size portion conditions varied between age groups	
***Risk of bias***		
**Bias**	**Authors' judgement**	**Support for judgement**
Random sequence generation (selection bias)	Unclear risk	Comment: method of sequence generation for condition order is not described. Insufficient information about the sequence generation process to permit judgement of 'low risk' or 'high risk'
Allocation concealment (selection bias)	Unclear risk	Comment: method of concealment is not described. Insufficient information to permit judgement of 'low risk' or 'high risk'
Blinding of participants and personnel (performance bias) Consumption outcome	Unclear risk	Quote: "Children's...comments about portion size, were measured using behavioral observations... To minimize visual comparisons of portion size, each child was assigned to eat with children of similar age in the same portion size condition... Children's comments regarding entrée portion size were recorded in each condition by a research staff member... Children made few comments about portion size. Seven of 75 children made comments in the large portion condition (e.g., "This is a lot of mac and cheese"; "This is a lot of food"; "This is more food than we get to eat at home"), whereas only one child made similar comments in the reference portion condition... The capacity of large portions to promote intake in both male and female children of varying ages and body weights raises the question of potential mechanism. Some have argued that large food packaging, food vessels, and portion sizes promote selection and consumption in adults by conveying greater expected consumption norms. In this case, visual cues provided by larger food portions are believed to implicitly reinforce greater consumption as being normative or appropriate. Behavioral observations made in the present study, however, suggest that children were unlikely to be affected by such norms because they were relatively unaware of the increases to entrée portion size." Comment: blinding of study participants attempted but it is possible that blinding was broken in some cases and that the outcome may be influenced by lack of blinding (due to potential carry‐over effects between conditions). Very unlikely that key study personnel were blinded, but the review authors judge that the outcome is not likely to be influenced by lack of blinding of key study personnel
Blinding of outcome assessment (detection bias) Consumption outcome	Low risk	Comment: no blinding of outcome assessment, but the review authors judge that the outcome measurement is not likely to be influenced by lack of blinding
Incomplete outcome data (attrition bias) Consumption outcome	High risk	Quote: "Complete intake data were obtained from 75 children. Because relative change in entrée consumption across conditions was of primary interest, cases in which entrée intake was 0 grams were not included in analyses: eight in the reference condition, two in the large portion condition, and four in the self‐selection condition. Also excluded from data analyses were two cases in which change scores were >3 SD above the mean: one case comparing entrée intake in the reference and large portion conditions (339% increase) and one case comparing entrée intake in the large and self‐selection conditions (226% increase). Analyses of relative change in entrée intake from the reference to large portion condition were performed on 65 cases. Those 10 excluded cases tended to be boys...from the two youngest age groups...but did not differ from those retained on the basis of child overweight." Comment: the reason for missing outcome data for consumption outcome is the study authors' decision to exclude participants with zero consumption and outliers (> 3 standard deviations above mean consumption) from the analysis. For the 2 to 3 years age group, the substantial proportion (7 participants, 28% of study sample) of exclusions due to zero consumption and outliers means that the review authors judge that it is plausible that the effect size among these missing data is enough to have had an important impact on the observed effect size. Similarly, for the 5 to 6 years age group, there was a substantial proportion (3 participants, 12% of study sample) of exclusions due to zero consumption and outliers
Selective reporting (reporting bias)	Unclear risk	Comment: search for record(s) containing details of study protocol conducted in ClinicalTrials.gov and the WHO International Clinical Trials Registry Platform (ICTRP). No records found. Insufficient information to permit judgement of 'low risk' or 'high risk'
Other bias #1 ‐ Baseline comparability of participant characteristics between groups	Low risk	Quote: "...the main analyses controlled for...condition order...". Comment: study uses a within‐subjects design. No measurement of participant pre‐condition 'state' characteristics is reported. However, the statistical analysis appears to control for the potential influence of condition order on measured outcomes. It is therefore unlikely that any differences between condition orders in terms of unmeasured pre‐condition participant 'state' characteristics influenced the measured outcomes. Risk of bias due to period effects is therefore judged low
Other bias #2 ‐ Consistency in intervention delivery	Unclear risk	Quote: "Parents were instructed to refrain from giving their child any foods or beverages for 2 hours before the visit. On arrival, a research member interviewed the parent to confirm that those instructions had been followed... three to four children were served dinner together in the presence of a research staff member...Children were instructed not to share food and to eat as little or as much as desired during the 20 minutes allotted for dinner." Comment: information and instructions provided to participants appear to have been standardised between the compared study conditions. Compliance with the instruction for parents to refrain from giving their child any foods or beverages 2 hours before each study visit was monitored via parent interview; however no monitoring results are reported with respect to this instruction. Although not explicitly stated, it is likely that compliance with the instruction for children not to share food was monitored by the research staff member present with each group of children for the duration of each dinner visit; however no monitoring results are reported with respect to this instruction. No further specific instructions were provided to participants, other than the instruction to eat as little or as much as desired during each 20‐min timed dinner
Summary of risk of bias Consumption outcome	Unclear risk	Unclear risk

**Table:** Fisher 2013

Methods	Study design: within‐subjects cluster‐randomised controlled trial Unit of allocation: group Unit of analysis: individual Number of clusters: not reported Number of participants per cluster: 3 to 4 Analysis appears to account for cluster allocation, as the group of children with whom each child ate during the experiment was modelled in each analysis	
Participants	Setting: laboratory setting Geographical region: not reported Number of enrolled participants: 77 children Number (%) of enrolled participants completing the study: 60 (78%) Study completers ‐ mean age (SD): 5.0 (0.6) Study completers ‐ sex: male (45%) and female (55%) Study completers ‐ mean BMI kg/m^2^ (SD): 0.39 (1.11) (BMI z score); 59.9 (29.4) (BMI percentile) Specific social or cultural characteristics: none Socio‐economic status context: low deprivation Inclusion criteria: aged between 4 and 6 years; English speaking Exclusion criteria: highly restrictive diet; severe food allergies; chronic illnesses affecting food intake; anticipated discomfort being separated from the parent during the experiment; perceived dislike of the study entrée or other study foods (> 2 of 4 accompanying foods); stated dislike of the study entrée, evaluated in an individual taste assessment interview before the experimental conditions; served 0 g of the study entrée at 2 or more of the experimental meals	
Interventions	Manipulated product type: food. Manipulation: Comparison 1: portion size; Comparison 2: tableware size Duration of exposure to intervention: ≤ 1 day Social setting: selecting and consuming with others Study arms: small portion (275 g) macaroni and cheese entrée, teaspoon; small portion (275 g) macaroni and cheese entrée, tablespoon; large portion (550 g) macaroni and cheese entrée, teaspoon; large portion (550 g) macaroni and cheese entrée, tablespoon Number of comparisons analysed: 2 Comparisons analysed: comparison 1: Intervention 1: small portion (275 g) macaroni and cheese entrée; *versus* Intervention 2: large portion (550 g) macaroni and cheese entrée; Comparison 2: Intervention 1: teaspoon; *versus* Intervention 2: tablespoon Concurrent intervention components: no	
Outcomes	Outcomes reported in study: amount of entrée self served (grams); number of spoonfuls of entrée self served (N); average (mean) grams per spoonful self served (grams); energy intake from total dinner meal (kcal); amount of food consumed from total dinner meal (grams); energy intake from macaroni and cheese entrée (kcal); amount of macaroni and cheese entrée consumed (grams); energy intake from other (non‐entrée) meal components (foods) (kcal); amount of other (non‐entrée) meal components (foods) consumed (grams) Selection outcome analysed: amount of entrée self served (grams) Measurement of selection outcome: objective Timing of selection outcome measurement: immediate (≤ 1 day) Consumption outcome analysed: N/A – no usable outcome data (energy intake from total dinner meal (kcal)) Measurement of consumption outcome: N/A – no usable outcome data (energy intake from total dinner meal (kcal)) Timing of consumption outcome measurement: N/A – no usable outcome data (energy intake from total dinner meal (kcal))	
Funding source	US Department of Agriculture Grant (NRI 2006‐55215‐16694); US Department of Agriculture CRIS funds	
Notes	Outcome data (selection) relating to portion size manipulation and tableware size manipulation analysed separately (one comparison each). No usable outcome data in published study report for consumption outcome. Author contacted to request information missing from the study report ‐ requested information was not supplied	
***Risk of bias***		
**Bias**	**Authors' judgement**	**Support for judgement**
Random sequence generation (selection bias)	Unclear risk	Comment: method of sequence generation for condition order is not described. Insufficient information about the sequence generation process to permit judgement of 'low risk' or 'high risk'
Allocation concealment (selection bias)	Unclear risk	Comment: method of concealment is not described. Insufficient information to permit judgement of 'low risk' or 'high risk'
Blinding of participants and personnel (performance bias) Selection outcome	Unclear risk	Quote: "To avoid visual comparisons of differences across conditions, each child was assigned to eat with 3–4 children in the same condition sequence." Comment: blinding of study participants attempted but it is possible that blinding was broken in some cases and that the outcome may be influenced by lack of blinding (due to potential carry‐over effects between conditions). Very unlikely that key study personnel were blinded, but the review authors judge that the outcome is not likely to be influenced by lack of blinding of key study personnel
Blinding of participants and personnel (performance bias) Consumption outcome	Unclear risk	Quote: "To avoid visual comparisons of differences across conditions, each child was assigned to eat with 3–4 children in the same condition sequence." Comment: blinding of study participants attempted but it is possible that blinding was broken in some cases and that the outcome may be influenced by lack of blinding (due to potential carry‐over effects between conditions). Very unlikely that key study personnel were blinded, but the review authors judge that the outcome is not likely to be influenced by lack of blinding of key study personnel
Blinding of outcome assessment (detection bias) Selection outcome	Low risk	Comment: no blinding of outcome assessment, but the review authors judge that the outcome measurement is not likely to be influenced by lack of blinding
Blinding of outcome assessment (detection bias) Consumption outcome	Low risk	Comment: no blinding of outcome assessment, but the review authors judge that the outcome measurement is not likely to be influenced by lack of blinding
Incomplete outcome data (attrition bias) Selection outcome	High risk	Quote: "Six children for whom consent was obtained were seen in 1 or fewer trials due to drop out, child refusal to participate, or child dislike of the entree (based on tasting assessment). Additionally, 11 children did not serve any of the entree in ≥two of the four conditions and were excluded from the analyses." Comment: 3 reasons for missing outcome data are dropout, child refusal to participate, or child dislike of the entree (based on tasting assessment). The latter 2 reasons are per protocol. Reasons for dropout are not provided. A fourth reason for missing outcome data is the study authors' decision to exclude participants with zero consumption in ≥ 2 of the 4 conditions from the analysis. The substantial proportion (11 participants, 14% of study sample) of exclusions due to zero consumption means that the review authors judge that it is plausible that the effect size among these missing data is enough to have had an important impact on the observed effect size
Incomplete outcome data (attrition bias) Consumption outcome	High risk	Quote: "Six children for whom consent was obtained were seen in 1 or fewer trials due to drop out, child refusal to participate, or child dislike of the entree (based on tasting assessment). Additionally, 11 children did not serve any of the entree in ≥two of the four conditions and were excluded from the analyses." Comment: 3 reasons for missing outcome data are dropout, child refusal to participate, or child dislike of the entree (based on tasting assessment). The latter 2 reasons are per protocol. Reasons for dropout are not provided. A fourth reason for missing outcome data is the study authors' decision to exclude participants with zero consumption in ≥ 2 of the 4 conditions from the analysis. The substantial proportion (11 participants, 14% of study sample) of exclusions due to zero consumption means that the review authors judge that it is plausible that the effect size among these missing data is enough to have had an important impact on the observed effect size
Selective reporting (reporting bias)	Unclear risk	Comment: search for record(s) containing details of study protocol conducted in ClinicalTrials.gov and the WHO International Clinical Trials Registry Platform (ICTRP). No records found. Insufficient information to permit judgement of 'low risk' or 'high risk'
Other bias #1 ‐ Baseline comparability of participant characteristics between groups	Low risk	Quote: "Time by condition interactions were estimated as random effects." Comment: study uses a within‐subjects design. No measurement of participant pre‐condition 'state' characteristics is reported. Analysis of potential differences in measured outcomes between condition orders appears to have been conducted and the statistical analysis appears to control for any influence of condition order on measured outcomes (condition by time interaction terms). It is therefore unlikely that any differences between condition orders in terms of unmeasured pre‐condition participant 'state' characteristics influenced the measured outcomes. Risk of bias due to period effects is therefore judged low
Other bias #2 ‐ Consistency in intervention delivery	Unclear risk	Quote: "Parents were instructed to refrain from giving their child any foods or beverages 2 h before the visit. Upon arrival, a research member interviewed the parent to confirm that those instructions were followed; any deviations were noted in the research record... At all visits, children ate dinner together in the presence of a research staff member... Children were instructed to serve themselves the entree using the serving spoon placed in each individual serving dish... Children were also told to serve themselves and eat as much as desired during the 20 min timed meal." Comment: information and instructions provided to participants appear to have been standardised between the compared study conditions. Compliance with the instruction for parents to refrain from giving their child any foods or beverages for 2 hours before each study visit was monitored via parent interview; however no monitoring results are reported with respect to this instruction. Whilst not explicitly stated, it is likely that compliance with the instruction for children to serve themselves the entree using the serving spoon placed in each individual serving dish was monitored by the member of research staff present for the duration of each dinner visit; however, no monitoring results are reported with respect to this instruction. No further specific instructions were provided to participants, other than the instruction to eat as much as desired during the 20 min timed meal
Summary of risk of bias Selection outcome	Unclear risk	Unclear risk
Summary of risk of bias Consumption outcome	Unclear risk	Unclear risk

**Table:** Flood 2006

Methods	Study design: within‐subjects randomised controlled trial	
Participants	Setting: laboratory setting Geographical region: Pennsylvania State University, University Park, Pennsylvania, USA Number of enrolled participants: 40 adults Number (%) of enrolled participants completing the study: 33 (83%) Study completers ‐ mean age (SD): 22.6 (1.2) Study completers ‐ sex: male (45%) and female (55%) Study completers ‐ mean BMI kg/m^2^ (SD): 23.5 (1.16) Specific social or cultural characteristics: none Socio‐economic status context: low deprivation Inclusion criteria: aged between18 and 45 years; regularly consumes 3 meals a day; reported liking of both regular and diet soda; BMI 18 to 40; scored < 40 on the Zung Questionnaire (measure of depression); scored < 20 on the Eating Attitudes Test (measures attitudes toward food and eating) Exclusion criteria: taking medications that are known to affect appetite or food intake; smoker; dieting to gain or lose weight; athlete in training; pregnant or breastfeeding; food allergies; food restrictions	
Interventions	Manipulated product type: food Manipulation: portion size Duration of exposure to intervention: ≤ 1 day Social setting: consuming alone Study arms: small size regular cola (360 g, PepsiCo Inc.), or diet cola (360 g, PepsiCo Inc.), or tap water (360 g) as part of a lunch meal also comprising an entrée of rotini pasta (450 g for females, 650 g for males) and tomato sauce (250 g for females, 375 g for males), a salad of romaine lettuce (50 g), cherry tomatoes (6 each) and parmesan cheese (15 g), a choice of salad dressings (43 g each), a roll (38 g) with butter spread (20 g) and chocolate chip cookies (80 g); large size regular cola (540 g, PepsiCo Inc.), or diet cola (540 g, PepsiCo Inc.), or tap water (360 g) as part of a lunch meal also comprising an entrée of rotini pasta (450 g for females, 650 g for males) and tomato sauce (250 g for females, 375 g for males), a salad of romaine lettuce (50 g), cherry tomatoes (6 each) and parmesan cheese (15 g), a choice of salad dressings (43 g each), a roll (38 g) with butter spread (20 g) and chocolate chip cookies (80 g) Number of comparisons analysed: 1 Comparisons analysed: Intervention 1: small size regular cola (360 g, PepsiCo Inc.), or diet cola (360 g, PepsiCo Inc.), or tap water (360g) as part of a lunch meal also comprising an entrée of rotini pasta (450 g for females, 650 g for males) and tomato sauce (250 g for females, 375 g for males), a salad of romaine lettuce (50 g), cherry tomatoes (6 each) and parmesan cheese (15 g), a choice of salad dressings (43 g each), a roll (38 g) with butter spread (20 g) and chocolate chip cookies (80 g); *versus* Intervention 2: large size regular cola (540 g, PepsiCo Inc.), or diet cola (540 g, PepsiCo Inc.), or tap water (360 g) as part of a lunch meal also comprising an entrée of rotini pasta (450 g for females, 650 g for males) and tomato sauce (250 g for females, 375 g for males), a salad of romaine lettuce (50 g), cherry tomatoes (6 each) and parmesan cheese (15 g), a choice of salad dressings (43 g each), a roll (38 g) with butter spread (20 g) and chocolate chip cookies (80 g) Concurrent intervention components: no	
Outcomes	Outcomes reported in study: energy intake from total lunch meal (kcal); energy intake from beverage at lunch (kcal); amount of beverage consumed at lunch (grams); energy intake from foods at lunch (kcal); energy intake from fat from foods at lunch (kcal); energy intake from carbohydrate from foods at lunch (kcal); energy intake from protein from foods at lunch (kcal); amount of foods consumed at lunch (grams) Selection outcome analysed: N/A Measurement of selection outcome: N/A Timing of selection outcome measurement: N/A Consumption outcome analysed: energy intake from total lunch meal (kcal) Measurement of consumption outcome: objective Timing of consumption outcome measurement: immediate (≤ 1 day)	
Funding source	National Institutes of Health (Grant DK59853)	
Notes	Outcome data relating to regular cola, diet cola and tap water analysed together (one comparison) because disaggregation was not possible. Author contacted to request information missing from the study report ‐ requested information was supplied (February 2014)	
***Risk of bias***		
**Bias**	**Authors' judgement**	**Support for judgement**
Random sequence generation (selection bias)	Unclear risk	Comment: method of sequence generation for condition order is not described. Insufficient information about the sequence generation process to permit judgement of 'low risk' or 'high risk'
Allocation concealment (selection bias)	Unclear risk	Comment: method of concealment is not described. Insufficient information to permit judgement of 'low risk' or 'high risk'
Blinding of participants and personnel (performance bias) Consumption outcome	Unclear risk	Quote: "Subjects gave signed consent and were told that the purpose of the study was to examine the effects of consumption of various foods and beverages... Subjects were not given information about the beverage type or portion size that they were served... On the discharge questionnaire... seven subjects (21%) noticed a change in beverage portion size during the study. Two subjects (6%) correctly reported that the purpose of the study was to examine the effect of changing beverage portion size on beverage intake, one subject (3%) correctly reported that the purpose of the study was to examine the effects of changing beverage portion size on food intake, and 13 subjects (39%) correctly reported that the purpose of the study was to examine the impact of changing beverage type on food intake. No subjects correctly reported all three study purposes. The mixed linear analysis showed that the primary study outcomes were not significantly influenced by whether subjects had correctly or incorrectly ascertained any purposes of the study (data not shown)." Comment: blinding of study participants attempted but blinding was broken in some cases and it is possible that the outcome may be influenced by lack of blinding (due to potential carry‐over effects between conditions). Very unlikely that key study personnel were blinded, but the review authors judge that the outcome is not likely to be influenced by lack of blinding of key study personnel
Blinding of outcome assessment (detection bias) Consumption outcome	Low risk	Comment: no blinding of outcome assessment, but the review authors judge that the outcome measurement is not likely to be influenced by lack of blinding
Incomplete outcome data (attrition bias) Consumption outcome	Low risk	Quote: "Forty subjects were enrolled in the study: 20 women and 20 men. Of these subjects, one woman and three men were excluded because they consumed the entire entrée served during a test meal. In addition, one woman and two men were excluded because of noncompliance with study protocol or inability to attend scheduled meals. Therefore, a total of 33 subjects completed the study (18 women and 15 men)." Comment: 2 reasons for missing outcome data for consumption outcome are noncompliance with study protocol or inability to attend scheduled meals. These reasons for missing outcome data are unlikely to be related to consumption outcome. The third reason for missing outcome data for consumption outcome is the study authors' decision to exclude participants who consumed the entire entrée ('plate cleaners') from the analysis. The review authors judge that this decision is reasonable, as it produces a more conservative estimate of the effect of the intervention on consumption. Any attrition bias due to handling of incomplete outcome data produces a more conservative estimate of the effect of the intervention on consumption
Selective reporting (reporting bias)	Unclear risk	Comment: search for record(s) containing details of study protocol conducted in ClinicalTrials.gov and the WHO International Clinical Trials Registry Platform (ICTRP). No records found. Insufficient information to permit judgement of 'low risk' or 'high risk'
Other bias #1 ‐ Baseline comparability of participant characteristics between groups	Unclear risk	Quote: "Before...each meal, subjects filled out a series of 100‐mm visual analog scales...to assess hunger, thirst, fullness, prospective consumption, and nausea...There were no significant differences across experimental conditions in ratings of hunger, fullness, thirst, prospective consumption, or nausea before lunch was served (data not shown)." Comment: study uses a within‐subjects design. No differences between conditions in terms of measured pre‐condition participant 'state' characteristics, but not reported whether there were differences between condition orders in terms of measured pre‐condition participant 'state' characteristics. No analysis of potential differences in measured outcomes between condition orders appears to have been conducted and the statistical analysis of outcome data does not appear to control for condition order. Risk of bias due to period effects is therefore unclear. Insufficient information to permit judgement of 'low risk' or 'high risk'
Other bias #2 ‐ Consistency in intervention delivery	Low risk	Quote: "On test days, subjects were instructed to consume only foods and beverages provided by the laboratory from the time they woke up in the morning until after the lunch session...Subjects were instructed not to drink alcohol in the 24 hours prior to coming to the laboratory, and not to consume dinner in a restaurant the evening before the test session. Subjects were also told to keep the amount of food eaten and physical activity performed the day before coming to the laboratory as consistent as possible across sessions, and completed a food and activity diary the day before each test session to encourage compliance with this protocol...Before each meal, subjects filled out a report to evaluate their compliance with study protocol...After completing the report, lunch was served, and subjects were instructed to eat and drink as much or as little of the foods and beverages as they wanted... One woman and two men were excluded because of noncompliance with study protocol or inability to attend scheduled meals." Comment: information and instructions provided to participants appear to have been standardised between the compared study conditions. Compliance with the instructions for participants regarding pre‐visit food and beverage consumption and physical activity was monitored via food and activity diary and a pre‐meal written self report. It is reported that a small number of participants were excluded from the analysis for not complying with these instructions. No further specific instructions were provided to participants, other than the instruction to eat and drink as much or as little of the foods and beverages as they wanted
Summary of risk of bias Consumption outcome	Unclear risk	Unclear risk

**Table:** Goldstein 2006

Methods	Study design: between‐subjects randomised controlled trial	
Participants	Setting: laboratory setting Geographical region: Lehigh University, Bethlehem, Pennsylvania, USA Number of enrolled participants: 40 undergraduate students Number (%) of enrolled participants completing the study: 40 (100%) Study completers ‐ mean age (SD): not reported Study completers ‐ sex: male (47%) and female (53%) Study completers ‐ mean BMI kg/m^2^ (SD): not reported Specific social or cultural characteristics: undergraduate university students Socio‐economic status context: low deprivation Inclusion criteria: undergraduate student; member of the 'Introduction to Psychology participant pool' Exclusion criteria: none reported	
Interventions	Manipulated product type: food Manipulation: portion size Duration of exposure to intervention: ≤ 1 day Social setting: consuming with others Study arms: small portion (80 g bag) of packaged, prepared popcorn; large portion (160 g bag) of packaged, prepared popcorn Number of comparisons analysed: 0 ‐ no usable outcome data Comparisons analysed: N/A ‐ no usable outcome data Concurrent intervention components: yes. 2 Tom & Jerry cartoon clips totalling 15 minutes ‐ provided to both Intervention 1 and Intervention 2 groups	
Outcomes	Outcomes reported in study: amount of popcorn consumed (grams) Selection outcome analysed: N/A Measurement of selection outcome: N/A Timing of selection outcome measurement: N/A Consumption outcome analysed: N/A ‐ no usable outcome data Measurement of consumption outcome: N/A ‐ no usable outcome data Timing of consumption outcome measurement: N/A ‐ no usable outcome data	
Funding source	Not reported	
Notes	No usable outcome data in published study report (amount of popcorn consumed (grams)). Attempts made to contact study authors via e‐mail, but no contact established	
***Risk of bias***		
**Bias**	**Authors' judgement**	**Support for judgement**
Random sequence generation (selection bias)	High risk	Quote: "We randomly assigned participants to conditions and kept a tally of the number of participants per condition in order to balance the number of participants in each condition." Comment: method of sequence generation appears likely to have been open to the influence of the researcher(s)
Allocation concealment (selection bias)	High risk	Quote: "We randomly assigned participants to conditions and kept a tally of the number of participants per condition in order to balance the number of participants in each condition." Comment: explicitly unconcealed procedure and investigators enrolling participants could possibly foresee assignments and thus introduce risk of selection bias
Blinding of participants and personnel (performance bias) Consumption outcome	Low risk	Quote: "Participants tested in the same group were all given the same amount of popcorn." Comment: blinding of study participants attempted and unlikely that the blinding could have been broken. Very unlikely that key study personnel were blinded, but the review authors judge that the outcome is not likely to be influenced by lack of blinding of key study personnel
Blinding of outcome assessment (detection bias) Consumption outcome	Low risk	Comment: no blinding of outcome assessment, but the review authors judge that the outcome measurement is not likely to be influenced by lack of blinding
Incomplete outcome data (attrition bias) Consumption outcome	Low risk	Comment: no missing outcome data for consumption outcome
Selective reporting (reporting bias)	Unclear risk	Comment: search for record(s) containing details of study protocol conducted in ClinicalTrials.gov and the WHO International Clinical Trials Registry Platform (ICTRP). No records found. Insufficient information to permit judgement of 'low risk' or 'high risk'
Other bias #1 ‐ Baseline comparability of participant characteristics between groups	Unclear risk	Comment: study uses a between‐subjects design. Insufficient information to permit judgement of 'low risk' or 'high risk'
Other bias #2 ‐ Consistency in intervention delivery	Low risk	Quote: "The experimenters verbally instructed participants that they would be watching a short 15 minute cartoon clip. They were told that they could enjoy some popcorn during the movie if they wished. Lastly, the experimenters told participants to wait patiently when the cartoon clip ended for further instructions. The experimenters distributed the bags of popcorn. Then, two randomly assigned cartoon clips of Tom & Jerry totaling 15 minutes were shown. Next, the experimenters instructed participants to remain seated while the bags of popcorn were being collected." Comment: information and instructions provided to participants appear to have been standardised between the compared study conditions. No further specific instructions were provided to participants, other than those described above and therefore monitoring of participants' compliance with instructions is not applicable
Summary of risk of bias Consumption outcome	High risk	High risk

**Table:** Hermans 2012

Methods	Study design: between‐subjects randomised controlled trial	
Participants	Setting: laboratory setting Geographical region: Netherlands Number of enrolled participants: 85 female undergraduate students Number (%) of enrolled participants completing the study: 85 (100%) Study completers ‐ mean age (SD): 20.8 (3.6) Study completers ‐ sex: female only Study completers ‐ mean BMI kg/m^2^ (SD): 22.4 (2.3) Specific social or cultural characteristics: undergraduate university students Socio‐economic status context: low deprivation Inclusion criteria: female Exclusion criteria: none reported	
Interventions	Manipulated product type: food Manipulation: portion size Duration of exposure to intervention: ≤ 1 day Social setting: consuming with others Study arms: small portion (250 g) macaroni Bolognese or spaghetti with cheese sauce or mash potato or lasagne*, eating companion's food intake small; small portion (250 g) macaroni Bolognese or spaghetti with cheese sauce or mash potato or lasagne*, eating companion's food intake standard; small portion (250 g) macaroni Bolognese or spaghetti with cheese sauce or mash potato or lasagne*, eating companion's food intake large; standard portion (500 g) macaroni Bolognese or spaghetti with cheese sauce or mash potato or lasagne*, eating companion's food intake small; standard portion (500 g) macaroni Bolognese or spaghetti with cheese sauce or mash potato or lasagne*, eating companion's food intake standard; standard portion (500 g) macaroni Bolognese or spaghetti with cheese sauce or mash potato or lasagne*, eating companion's food intake large * Each participant was asked to choose among 4 different meals before registering for the study in order to ensure that they liked the test food offered Number of comparisons analysed: 1 Comparisons analysed: Intervention 1: small portion (250 g) macaroni Bolognese or spaghetti with cheese sauce or mash potato or lasagne; *versus* Intervention 2: standard portion (500 g) macaroni Bolognese or spaghetti with cheese sauce or mash potato or lasagne Concurrent intervention components: yes. Confederate instructed to eat x% of a same‐size portion (50% versus 100% versus 150%) – provided to both Intervention 1 and Intervention 2 groups	
Outcomes	Outcomes reported in study: amount of food consumed from entrée (grams); energy intake from entrée (kilojoules) Selection outcome analysed: N/A Measurement of selection outcome: N/A Timing of selection outcome measurement: N/A Consumption outcome analysed: amount of food consumed from entrée (grams) Measurement of consumption outcome: objective Timing of consumption outcome measurement: immediate (≤ 1 day)	
Funding source	Fellowship grant from the Netherlands Organization for Scientific Research	
Notes	Outcome data for 'eating companion's food intake small', 'eating companion's food intake standard' and 'eating companion's food intake large' participant subgroups collapsed and analysed together (one comparison)	
***Risk of bias***		
**Bias**	**Authors' judgement**	**Support for judgement**
Random sequence generation (selection bias)	Unclear risk	Comment: method of sequence generation is not described. Insufficient information about the sequence generation process to permit judgement of 'low risk' or 'high risk'
Allocation concealment (selection bias)	Unclear risk	Comment: method of concealment is not described. Insufficient information to permit judgement of 'low risk' or 'high risk'
Blinding of participants and personnel (performance bias) Consumption outcome	Low risk	Quote: "Upon arriving at the front office of the research facility, both participants were informed that the purpose of the study was to examine the effects of nutrition on cognitive test performance. Participants were asked to read and provide written consent and were then asked to stand in front of the television screen and the Nintendo Wii. They were asked to individually play a Wii game in which their cognitive performance both before and after meal consumption was tested. In the meanwhile, the confederate completed three paper‐and‐pencil tasks involving concentration and spatial insight. These tasks took approximately 15 min. Because the true purpose of the study was to examine the effects of portion size and the intake of others on actual intake (and not cognitive performance), the cognitive tasks were bogus tests and the second set of cognitive tests never occurred... After the participant had completed the questionnaire, her height and weight were measured, and she received a short debriefing about the purpose of the study. After all data were collected, participants were fully debriefed about the study by email... Participants' ratings of portion size varied significantly as a function of the portion‐size manipulation. Participants perceived the portion as smaller in the small portion conditions...than in the standard‐ size portion conditions...confirming that the portion‐size manipulation was successful." Comment: blinding of study participants attempted and unlikely that the blinding could have been broken. Very unlikely that key study personnel were blinded, but the review authors judge that the outcome is not likely to be influenced by lack of blinding of key study personnel
Blinding of outcome assessment (detection bias) Consumption outcome	Low risk	Comment: no blinding of outcome assessment, but the review authors judge that the outcome measurement is not likely to be influenced by lack of blinding
Incomplete outcome data (attrition bias) Consumption outcome	Low risk	Comment: no missing outcome data for consumption outcome
Selective reporting (reporting bias)	Unclear risk	Comment: search for record(s) containing details of study protocol conducted in ClinicalTrials.gov and the WHO International Clinical Trials Registry Platform (ICTRP). No records found. Insufficient information to permit judgement of 'low risk' or 'high risk'
Other bias #1 ‐ Baseline comparability of participant characteristics between groups	Low risk	Quote: "BMI, measured as weight.../height2 (m2) was calculated based on measured height and weight. Participants' weight and height were measured following standard procedures......Restrained eating was measured by the dietary restraint subscale of the Dutch Eating Behaviour Questionnaire...External eating was measured by the external eating subscale of the Dutch Eating Behaviour Questionnaire...The results of ANOVA indicated no significant differences in age, BMI, hunger level, dietary restraint and external eating across conditions." Comment: study uses a between‐subjects design. Method for measuring pre‐meal hunger is not reported. No differences between comparison groups in terms of measured baseline participant characteristics
Other bias #2 ‐ Consistency in intervention delivery	Unclear risk	Quote: "All participants were asked to refrain from eating for 3 [hours] before their scheduled session to control for individual variations in hunger... Upon arriving at the front office of the research facility, both participants were informed that the purpose of the study was to examine the effects of nutrition on cognitive test performance... Participants...were then asked to stand in front of the television screen and the Nintendo Wii. They were asked to individually play a Wii game in which their cognitive performance both before and after meal consumption was tested... After performing the cover tasks, the confederate and the participant were asked to sit down at the table that was especially set for them. They would have 20 min to eat a complete meal. During this time, participants were free to talk and interact as they would during a normal meal... After approximately 5 min, the experimenter came back and served the meal (described below) while informing the participants that they could eat as much or as little as they liked and that more food was available on the hot plate if they wanted to eat more. At this point, the experimenter told the participants to 'enjoy their meal' and left the room. These instructions were used during all sessions." Comment: information and instructions provided to participants appear to have been standardised between the compared study conditions. No information pertaining to monitoring of participants' compliance with the instruction to refrain from eating for 3 hours before their scheduled session is reported. Insufficient information to permit judgement of 'low risk' or 'high risk'
Summary of risk of bias Consumption outcome	Unclear risk	Unclear risk

**Table:** Huss 2013

Methods	Study design: within‐subjects cluster‐randomised controlled trial Unit of allocation: classroom Unit of analysis: individual Number of clusters: 4 Number of participants per cluster: not reported Analysis appears to account for cluster allocation, as the statistical model accounted for between‐subjects variation in classroom and the classroom variable was used to determine main effects and interactions	
Participants	Setting: field setting, university childcare centre Geographical region: West Lafayette, IN, USA Number of enrolled participants: 23 children Number (%) of enrolled participants completing the study: 23 (100%) Study completers ‐ mean age (SD): not reported Study completers ‐ sex: male (74%) and female (26%) Study completers ‐ mean BMI kg/m^2^ (SD): not reported (BMI z score/ BMI percentile) Specific social or cultural characteristics: no Socio‐economic status context: low deprivation Inclusion criteria: aged 2 to 5 years; attending childcare centre for full day Exclusion criteria: food restrictions; food allergies; digestive diseases (e.g. Crohn's disease, cystic fibrosis)	
Interventions	Manipulated product type: food Manipulation: portion size Duration of exposure to intervention: > 1 day Social setting: consuming with others Study arms: reference size portions, dessert served concurrently with entrée; reference size portions, dessert served after entrée; large size portions, dessert served concurrently with entrée; large size portions, dessert served after entrée. Number of comparisons analysed: 1 Comparisons analysed: Intervention 1: reference size portions (1 ounce baked freshwater fish, 1/4 cup mixed vegetables, 1/4 cup orange, 1/4 cup rice at 2 lunch meals for 2‐year olds OR 1.5 ounces baked freshwater fish, 1/2 cup mixed vegetables, 1/2 cup orange, 1/4 cup rice at 2 lunch meals for 3‐ to 5‐year olds; 1/4 cup pasta, 1 ounce meat sauce, 1/4 cup mixed vegetables, 1/4 cup mixed fruit at 2 lunch meals for 2‐year olds OR 1/4 cup pasta, 1.5 ounces meat sauce, 1/2 cup mixed vegetables, 1/2 cup mixed fruit at 2 lunch meals for 3‐ to 5‐year olds); *versus* Intervention 2: large size portions (1.5 ounces baked freshwater fish, 1/3 cup mixed vegetables, 1/3 cup orange, 1/3 cup rice at 2 lunch meals for 2 year olds OR 2.25 ounces baked freshwater fish, 3/4 cup mixed vegetables, 3/4 cup orange, 1/3 cup rice at 2 lunch meals for 3‐ to 5‐year olds; 1/3 cup pasta, 1.5 ounces meat sauce, 1/3 cup mixed vegetables, 1/3 cup mixed fruit at 2 lunch meals for 2‐year olds OR 1/3 cup pasta, 2.25 ounces meat sauce, 3/4 cup mixed vegetables, 3/4 cup mixed fruit at 2 lunch meals for 3‐ to 5‐year olds) Concurrent intervention components: no	
Outcomes	Outcomes reported in study: average (mean) energy intake from total lunch meal (kcal); average (mean) energy intake from main course at lunch (kcal); average (mean) energy intake from dessert at lunch (kcal) Selection outcome analysed: N/A Measurement of selection outcome: N/A Timing of selection outcome measurement: N/A Consumption outcome analysed: average (mean) energy intake from total lunch meal (kcal) Measurement of consumption outcome: objective Timing of consumption outcome measurement: longer‐term (> 1 day)	
Funding source	Not reported	
Notes	Outcome data for children aged 2 years and children aged 3 to 5 years analysed together (one comparison) because these data could not be disaggregated by age group. Absolute and relative differences in portion size between reference size and large size portion conditions varied between age groups	
***Risk of bias***		
**Bias**	**Authors' judgement**	**Support for judgement**
Random sequence generation (selection bias)	High risk	Quote: "The researchers randomly assigned the classrooms to one of the four possible combinations of portion size and timing of dessert... on each day. In one given day, the children in one classroom were undergoing the same treatment. For 12 weeks (4 week baseline and 8 week intervention), the children received fish on Thursdays and pasta on Fridays. Randomization was not conducted for all weeks of the study to assure that each classroom had equal amounts of repeated exposures."
Allocation concealment (selection bias)	Low risk	Comment: participating classrooms appear to have been randomised to condition order concurrently, after consent for individuals' participation had been obtained. The review authors therefore judge that any lack of concealment of allocation sequence is unlikely to be an issue for risk of bias
Blinding of participants and personnel (performance bias) Consumption outcome	Unclear risk	Comment: no blinding of study participants and not reported whether participants were probed for suspicion of study purpose or awareness of size manipulation between study conditions. It is possible that the outcome may be influenced by lack of blinding of study participants (due to potential carry‐over effects between conditions). Very unlikely that key study personnel were blinded, but the review authors judge that the outcome is not likely to be influenced by lack of blinding of key study personnel
Blinding of outcome assessment (detection bias) Consumption outcome	Low risk	Comment: no blinding of outcome assessment, but the review authors judge that the outcome measurement is not likely to be influenced by lack of blinding
Incomplete outcome data (attrition bias) Consumption outcome	Low risk	Comment: no missing outcome data for consumption outcome
Selective reporting (reporting bias)	Unclear risk	Comment: search for record(s) containing details of study protocol conducted in ClinicalTrials.gov and the WHO International Clinical Trials Registry Platform (ICTRP). No records found. Insufficient information to permit judgement of 'low risk' or 'high risk'
Other bias #1 ‐ Baseline comparability of participant characteristics between groups	Low risk	Quote: "The between‐subject factors were...4‐week menu rotation...". Comment: study uses a within‐subjects design. No measurement of participant pre‐condition 'state' characteristics is reported. Analysis of potential differences in measured outcomes between condition orders appears to have been conducted and the statistical analysis appears to control for any influence of condition order on measured outcomes ("4‐week menu rotation"). It is therefore unlikely that any differences between condition orders in terms of unmeasured pre‐condition participant 'state' characteristics influenced the measured outcomes. Risk of bias due to period effects is therefore judged low
Other bias #2 ‐ Consistency in intervention delivery	Unclear risk	Quote: "Teachers in participating classrooms were instructed to follow standard mealtime procedures for mid‐morning snack and lunch. In each classroom the participating children would sit at a table together and were served lunch by a research assistant. Children were not encouraged to eat more or less than usual and were instructed not to share food." Comment: information and instructions provided to participants appear to have been standardised between the compared study conditions. No information pertaining to monitoring of teachers' compliance with the instruction to follow standard mealtime procedures for mid‐morning snack and lunch is reported. Whilst not explicitly reported, it is likely that participants' compliance with the instruction not to share food was monitored by a research assistant who was present for the duration of each lunch session; however, no monitoring results are reported with respect to this instruction. No further specific instructions were provided to participants or providers
Summary of risk of bias Consumption outcome	High risk	High risk

**Table:** Jarvik 1978 (E1)

Methods	Study design: within‐subjects randomised controlled trial	
Participants	Setting: laboratory setting Geographical region: Brentwood Veterans Administration Hospital, West Los Angeles, CA, USA Number of enrolled participants: 9 adult males Number (%) of enrolled participants completing the study: 9 (100%) Study completers ‐ mean age (SD): not reported Study completers ‐ sex: male only Study completers ‐ mean BMI kg/m^2^ (SD): not reported (neither BMI nor other body weight or body weight status) Specific social or cultural characteristics: patients at a Veterans Administration hospital Socio‐economic status context: low deprivation Inclusion criteria: current smoker; patient at the Brentwood Veterans Administration Hospital, West Los Angeles, CA, USA Exclusion criteria: none reported	
Interventions	Manipulated product type: tobacco Manipulation: individual unit size Duration of exposure to intervention: ≤ 1 day Social setting: consuming alone Study arms: one eighth‐length cigarettes; one quarter‐length cigarettes; half‐length cigarettes; full‐length cigarettes Number of comparisons analysed: 3 Comparisons analysed: comparison 1 ‐ Intervention 1: one eighth‐length cigarettes; *versus* Intervention 2: one quarter‐length cigarettes. Comparison 2 ‐ Intervention 1: one quarter‐length cigarettes;*versus* Intervention 2: half‐length cigarettes. Comparison 3 ‐ Intervention 1: half‐length cigarettes;*versus* Intervention 2: full‐length cigarettes Concurrent intervention components: no	
Outcomes	Outcomes reported in study: total number of puffs from all cigarettes consumed (N); total number of cigarettes consumed (N) Selection outcome analysed: N/A Measurement of selection outcome: N/A Timing of selection outcome measurement: N/A Consumption outcome analysed: total number of puffs from all cigarettes consumed (N) Measurement of consumption outcome: objective Timing of consumption outcome measurement: immediate (≤ 1 day)	
Funding source	Not reported	
Notes	Incremental comparisons only analysed	
***Risk of bias***		
**Bias**	**Authors' judgement**	**Support for judgement**
Random sequence generation (selection bias)	Unclear risk	Comment: method of sequence generation for condition order is not described. Insufficient information about the sequence generation process to permit judgement of 'low risk' or 'high risk'
Allocation concealment (selection bias)	Unclear risk	Comment: method of concealment is not described. Insufficient information to permit judgement of 'low risk' or 'high risk'
Blinding of participants and personnel (performance bias) Consumption outcome	Unclear risk	Quote: "On the first day, subjects were asked to read and sign the consent form, after which they were informed that they would be smoking different sizes of cigarettes on different days." Comment: no blinding of study participants and it is possible that the outcome may be influenced by lack of blinding (due to potential carry‐over effects between conditions). Very unlikely that key study personnel were blinded, but the review authors judge that the outcome is not likely to be influenced by lack of blinding of key study personnel
Blinding of outcome assessment (detection bias) Consumption outcome	Low risk	Comment: no blinding of outcome assessment, but the review authors judge that the outcome measurement is not likely to be influenced by lack of blinding
Incomplete outcome data (attrition bias) Consumption outcome	Low risk	Comment: no missing outcome data for consumption outcome
Selective reporting (reporting bias)	Unclear risk	Comment: search for record(s) containing details of study protocol conducted in ClinicalTrials.gov and the WHO International Clinical Trials Registry Platform (ICTRP). No records found. Insufficient information to permit judgement of 'low risk' or 'high risk'
Other bias #1 ‐ Baseline comparability of participant characteristics between groups	Unclear risk	Comment: study uses a within‐subjects design. No measurement of participant pre‐condition 'state' characteristics is reported. No analysis of potential differences in measured outcomes between condition orders appears to have been conducted and the statistical analysis of outcome data does not appear to control for condition order. Risk of bias due to period effects is therefore unclear. Insufficient information to permit judgement of 'low risk' or 'high risk'
Other bias #2 ‐ Consistency in intervention delivery	Low risk	Comment: information provided to participants appears to have been standardised between the compared study conditions. No specific instructions were provided to participants and therefore participants' compliance with instructions is not applicable
Summary of risk of bias Consumption outcome	Unclear risk	Unclear risk

**Table:** Jarvik 1978 (E2)

Methods	Study design: within‐subjects randomised controlled trial	
Participants	Setting: laboratory setting Geographical region: Brentwood Veterans Administration Hospital, West Los Angeles, CA, USA Number of enrolled participants: 28 adults Number (%) of enrolled participants completing the study: 9 (100%) Study completers ‐ mean age (SD): not reported Study completers ‐ sex: male (95%) and female (5%) Study completers ‐ mean BMI kg/m^2^ (SD): not reported (neither BMI nor other body weight or body weight status) Specific social or cultural characteristics: patients at a Veterans Administration hospital Socio‐economic status context: low deprivation Inclusion criteria: current smoker; current smoker of at least 1 pack per day; patient at the Brentwood Veterans Administration Hospital, West Los Angeles, CA, USA Exclusion criteria: none reported	
Interventions	Manipulated product type: tobacco Manipulation: individual unit size Duration of exposure to intervention: ≤ 1 day Social setting: consuming alone Study arms: one quarter‐length cigarettes, low nicotine content; one quarter‐length cigarettes, high nicotine content; full‐length cigarettes, low nicotine content; full‐length cigarettes, high nicotine content Number of comparisons analysed: 1 Comparisons analysed: Intervention 1: one quarter‐length cigarettes;*versus* Intervention 2: full‐length cigarettes Concurrent intervention components: no	
Outcomes	Outcomes reported in study: total number of puffs from all cigarettes consumed (N); total number of cigarettes consumed (N) Selection outcome analysed: N/A Measurement of selection outcome: N/A Timing of selection outcome measurement: N/A Consumption outcome analysed: total number of puffs from all cigarettes consumed (N) Measurement of consumption outcome: objective Timing of consumption outcome measurement: immediate (≤ 1 day)	
Funding source	Not reported	
Notes	Outcome data for 'low nicotine content' and 'high nicotine content' participant subgroups collapsed and analysed together (one comparison)	
***Risk of bias***		
**Bias**	**Authors' judgement**	**Support for judgement**
Random sequence generation (selection bias)	Unclear risk	Comment: method of sequence generation for condition order is not described. Insufficient information about the sequence generation process to permit judgement of 'low risk' or 'high risk'
Allocation concealment (selection bias)	Unclear risk	Comment: method of concealment is not described. Insufficient information to permit judgement of 'low risk' or 'high risk'
Blinding of participants and personnel (performance bias) Consumption outcome	Unclear risk	Comment: no blinding of study participants and it is possible that the outcome may be influenced by lack of blinding (due to potential carry‐over effects between conditions). Very unlikely that key study personnel were blinded, but the review authors judge that the outcome is not likely to be influenced by lack of blinding of key study personnel
Blinding of outcome assessment (detection bias) Consumption outcome	Low risk	Comment: no blinding of outcome assessment, but the review authors judge that the outcome measurement is not likely to be influenced by lack of blinding
Incomplete outcome data (attrition bias) Consumption outcome	Low risk	Quote: "Data from six of the male subjects was excluded because of machine failures." Comment: the reason for missing outcome data is unlikely to be related to consumption outcome
Selective reporting (reporting bias)	Unclear risk	Comment: search for record(s) containing details of study protocol conducted in ClinicalTrials.gov and the WHO International Clinical Trials Registry Platform (ICTRP). No records found. Insufficient information to permit judgement of 'low risk' or 'high risk'
Other bias #1 ‐ Baseline comparability of participant characteristics between groups	Unclear risk	Comment: study uses a within‐subjects design. No measurement of participant pre‐condition 'state' characteristics is reported. No analysis of potential differences in measured outcomes between condition orders appears to have been conducted and the statistical analysis of outcome data does not appear to control for condition order. Risk of bias due to period effects is therefore unclear. Insufficient information to permit judgement of 'low risk' or 'high risk'
Other bias #2 ‐ Consistency in intervention delivery	Low risk	Comment: information provided to participants appears to have been standardised between the compared study conditions. No specific instructions were provided to participants and therefore participants' compliance with instructions is not applicable
Summary of risk of bias Consumption outcome	Unclear risk	Unclear risk

**Table:** Jeffery 2007

Methods	Study design: within‐subjects randomised controlled trial	
Participants	Setting: field setting, community medical centre Geographical region: USA Number of enrolled participants: 20 adult females Number (%) of enrolled participants completing the study: 19 (95%) Study completers ‐ mean age (SD): not reported Study completers ‐ sex: female only Study completers ‐ mean BMI kg/m^2^ (SD): not reported (neither BMI nor other body weight or body weight status) Specific social or cultural characteristics: no Socio‐economic status context: low deprivation Inclusion criteria: female; aged between 18 and 40 years; employee of a community medical centre; self reported BMI 18.5 to 40.0; willing to consent to the conditions of study participation Exclusion criteria: pregnancy; recently given birth; actively dieting to control weight; more than 3 days a week moderate physical activity	
Interventions	Manipulated product type: food Manipulation: portion size Duration of exposure to intervention: > 1 day Social setting: consuming alone and with others Study arms: small size box lunch*, provided 5 days per week for 4 weeks; large size box lunch*, provided 5 days per week for 4 weeks * Box lunches comprised various foods and non‐alcoholic beverages (rotation of 7 different lunches). The contents were typical lunch items that included a main course, side dish, dessert and a drink. Main courses were sandwiches or salads. Side dishes were fruit or vegetable salad, chips or bread depending on the main course. Desserts were cookies or bars. Drinks were water, Coke or Sprite Number of comparisons analysed: 1 Comparisons analysed: Intervention 1: small size box lunch, provided 5 days per week for 4 weeks;*versus* Intervention 2: large size box lunch, provided 5 days per week for 4 weeks Concurrent intervention components: no	
Outcomes	Outcomes reported in study: average (mean) total energy intake per day (kcal); average (mean) percentage energy intake from fat per day (%) Selection outcome analysed: N/A Measurement of selection outcome: N/A Timing of selection outcome measurement: N/A Consumption outcome analysed: average (mean) total energy intake per day (kcal) Measurement of consumption outcome: self report Timing of consumption outcome measurement: longer‐term (> 1 day)	
Funding source	University of Minnesota Obesity Prevention Center; National Institute of Diabetes and Digestive and Kidney Diseases (Grant No. DK50456)	
Notes	Author contacted to request information missing from the study report ‐ requested information was not supplied	
***Risk of bias***		
**Bias**	**Authors' judgement**	**Support for judgement**
Random sequence generation (selection bias)	Unclear risk	Comment: method of sequence generation for condition order is not described. Insufficient information about the sequence generation process to permit judgement of 'low risk' or 'high risk'
Allocation concealment (selection bias)	Unclear risk	Comment: method of concealment is not described. Insufficient information to permit judgement of 'low risk' or 'high risk'
Blinding of participants and personnel (performance bias) Consumption outcome	Unclear risk	Quote: "Candidates were told that the study was being conducted to assess factors influencing eating habits and the feasibility of providing daily box lunches. No specific mention was made of portion size or energy intake as study objectives until the final follow‐up visit at which time the study purpose was disclosed. Because all participants received both sets of lunches, and because individuals receiving different portion size lunches were not prevented from interacting during the study, many became aware of the portion size manipulation as the study progressed, but most remained unaware of the study's intent. Although blinding to the portion size manipulation was considered, it was not attempted, in part because we thought it could be difficult to do while keeping the study exposures naturalistic, and in part because we thought that any bias related to knowledge of portion size would probably work against rather than for observing a portion size effect on intake." Comment: no blinding of study participants and it is possible that the outcome may be influenced by lack of blinding (due to potential carry‐over effects between conditions). Very unlikely that key study personnel were blinded, but the review authors judge that the outcome is very unlikely to be influenced by lack of blinding of key study personnel
Blinding of outcome assessment (detection bias) Consumption outcome	Unclear risk	Quote: "First, dietary intake at lunch was assessed by having study participants complete a self‐administered questionnaire after each lunch in which they estimated the proportion of each food item eaten using a visual analogue scale... They also reported any food items eaten at lunch that were not from their lunch box... The second diet assessment method was to conduct two 24‐hour dietary recalls by telephone on randomly selected days for each participant during each of the lunch intervention weeks." Comment: no blinding of outcome assessment and it is possible that the outcome measurement may be influenced by lack of blinding
Incomplete outcome data (attrition bias) Consumption outcome	Low risk	Quote: "One participant had to withdraw from the study very early due to a health problem." Comment: the reason for missing outcome data is unlikely to be related to consumption outcome
Selective reporting (reporting bias)	Unclear risk	Comment: search for record(s) containing details of study protocol conducted in ClinicalTrials.gov and the WHO International Clinical Trials Registry Platform (ICTRP). No records found. Insufficient information to permit judgement of 'low risk' or 'high risk'
Other bias #1 ‐ Baseline comparability of participant characteristics between groups	Low risk	Quote: "The analyses of the meal size manipulation on kilocalories consumed and on percent calories from fat at the lunch meal and per day were carried out using a general linear mixed model analysis, controlling for order of lunch presentation and physical activity as fixed effects and participant as a random effect." Comment: study uses a within‐subjects design. No measurement of participant pre‐condition 'state' characteristics is reported. However, the statistical analysis appears to control for the potential influence of condition order on measured outcomes. It is therefore unlikely that any differences between condition orders in terms of unmeasured pre‐condition participant 'state' characteristics influenced the measured outcomes. Risk of bias due to period effects is therefore judged low
Other bias #2 ‐ Consistency in intervention delivery	Low risk	Comment: information provided to participants appears to have been standardised between the compared study conditions. No specific instructions were provided to participants and therefore participants' compliance with instructions is not applicable
Summary of risk of bias Consumption outcome	Unclear risk	Unclear risk

**Table:** Kelly 2009

Methods	Study design: within‐subjects randomised controlled trial	
Participants	Setting: laboratory setting Geographical region: Belfast, Northern Ireland Number of enrolled participants: 44 adults Number (%) of enrolled participants completing the study: 43 (98%) Study completers ‐ mean age (SD): 30.7 (7.5) Study completers ‐ sex: male (49%) and female (51%) Study completers ‐ mean BMI kg/m^2^ (SD): 24.5 (3.2) Specific social or cultural characteristics: no Socio‐economic status context: low deprivation Inclusion criteria: aged between 18 and 65 years Exclusion criteria: current smoker; vegetarian; taking prescription medications or any drugs that might interfere with normal food intake; food allergies or dietary restrictions; chronic disease; BMI < 18.5 or > 30 kg/m^2^; unwilling to participate in fully residential study	
Interventions	Manipulated product type: food Manipulation: portion size Duration of exposure to intervention: > 1 day Social setting: consuming alone and with others Study arms: standard portions of breakfast, lunch, dinner meals and snacks* provided for 4 consecutive days; large portions of breakfast, lunch, dinner meals and snacks* provided for 4 consecutive days * Various foods and non‐alcoholic beverages Number of comparisons analysed: 1 Comparisons analysed: Intervention 1: standard portions of breakfast, lunch, dinner meals and snacks provided for 4 consecutive days;*versus* Intervention 2: large portions of breakfast, lunch, dinner meals and snacks provided for 4 consecutive days Concurrent intervention components: no	
Outcomes	Outcomes reported in study: total energy intake over 4 days from all meals and snacks (megajoules); average (mean) daily energy intake from all meals and snacks (megajoules); energy intake from breakfast on day 1 (megajoules); energy intake from breakfast on day 2 (megajoules); energy intake from breakfast on day 3 (megajoules); energy intake from breakfast on day 4 (megajoules); energy intake from lunch on day 1 (megajoules); energy intake from lunch on day 2 (megajoules); energy intake from lunch on day 3 (megajoules); energy intake from lunch on day 4 (megajoules); energy intake from dinner on day 1 (megajoules); energy intake from dinner on day 2 (megajoules); energy intake from dinner on day 3 (megajoules); energy intake from dinner on day 4 (megajoules); energy intake from all snacks on day 1 (megajoules); energy intake from all snacks on day 2 (megajoules); energy intake from all snacks on day 3 (megajoules); energy intake from all snacks on day 4 (megajoules); percentage energy intake from fat over 4 days (%); percentage energy intake from carbohydrate over 4 days (%); percentage energy intake from protein over 4 days (%); percentage of total foods provided that were consumed over 4 days (%) Selection outcome analysed: N/A Measurement of selection outcome: N/A Timing of selection outcome measurement: N/A Consumption outcome analysed: total energy intake over 4 days from all meals and snacks (megajoules) Measurement of consumption outcome: objective Timing of consumption outcome measurement: longer‐term (> 1 day)	
Funding source	Food Standards Agency (Project N09021)	
Notes	—	
***Risk of bias***		
**Bias**	**Authors' judgement**	**Support for judgement**
Random sequence generation (selection bias)	Unclear risk	Comment: method of sequence generation for condition order is not described. Insufficient information about the sequence generation process to permit judgement of 'low risk' or 'high risk'
Allocation concealment (selection bias)	Unclear risk	Comment: method of concealment is not described. Insufficient information to permit judgement of 'low risk' or 'high risk'
Blinding of participants and personnel (performance bias) Consumption outcome	Unclear risk	Quote: "Differing sizes of serving dishes were used for the two portion treatments so that visually the portions would not seem different to the subjects... To ensure that subjects remained blind as to the true nature of the study, the consent form stated that the purpose of the study was to investigate the effect of mood on food choice... At the end of each 4 d study period subjects completed an end‐of‐study questionnaire designed to rate their perceptions of the portion sizes offered. In order to avoid drawing the subjects' attention to these questions, the food portion questions were embedded in a range of more general questions about mood and surroundings... The end‐of‐study questionnaire revealed that 55% of men felt that the portions were 'just about right' on both the standard and large portion conditions for all meals. In the women 62% reported the portions were 'just about right' on the standard portion condition but 74% reported that they would have been 'satisfied with smaller' on the large portion condition. Despite this, the women still consumed more food and increased their EI by 10% under the large portion condition." Comment: blinding of study participants attempted but it is possible that blinding was broken in some cases. Not reported whether participants were probed for suspicion of study purpose or awareness of size manipulation between study conditions. It is possible that the outcome may be influenced by lack of blinding of study participants (due to potential carry‐over effects between conditions). Very unlikely that key study personnel were blinded, but the review authors judge that the outcome is not likely to be influenced by lack of blinding of key study personnel
Blinding of outcome assessment (detection bias) Consumption outcome	Low risk	Comment: no blinding of outcome assessment, but the review authors judge that the outcome measurement is not likely to be influenced by lack of blinding
Incomplete outcome data (attrition bias) Consumption outcome	Low risk	Quote: "One subject did not comply with the study protocol and was excluded." Comment: the nature of the participant's failure to comply with the study protocol is not provided, so it is unclear whether the reason for this exclusion is likely to be related to the study outcome or not. The low proportion (one participant, 2% of study sample) of exclusions due to outliers means that the review authors judge that the plausible effect size among missing outcomes is unlikely to be enough to have an important impact on the observed effect size
Selective reporting (reporting bias)	Unclear risk	Comment: search for record(s) containing details of study protocol conducted in ClinicalTrials.gov and the WHO International Clinical Trials Registry Platform (ICTRP). No records found. Insufficient information to permit judgement of 'low risk' or 'high risk'
Other bias #1 ‐ Baseline comparability of participant characteristics between groups	Low risk	Quote: "Subjects completed visual analogue scales immediately before...each meal to rate their feelings of hunger, fullness, desire to eat and prospective consumption... Covariates in the main model were sex, age (years), BMI (kg/m2) and treatment order...When ratings on the large portion study period were compared with the standard portion study period, subjects reported that before eating, they were less hungry...more full...had less of a desire to eat...and thought they could eat a smaller amount." Comment: differences between conditions in terms of measured pre‐condition participant 'state' characteristics are reported, but not reported whether there were differences between condition orders in terms of measured pre‐condition participant 'state' characteristics. However, the statistical analysis appears to control for the potential influence of condition order on measured outcomes. It is therefore unlikely that any differences between condition orders in terms of unmeasured pre‐condition participant 'state' characteristics influenced the measured outcomes. Risk of bias due to period effects is therefore judged low
Other bias #2 ‐ Consistency in intervention delivery	Low risk	Quote: "Subjects were asked to refrain from eating and drinking from 21.00 hours on the evening prior to each study period... Subjects were instructed to consume only the foods and beverages that were provided for them in the Human Intervention Studies Unit and not to share food items with others. Subjects were advised that they could consume as much of the foods and beverages as desired on both the standard and large portion conditions and were aware that more food was always available on request... One subject did not comply with the study protocol and was excluded." Comment: information and instructions provided to participants appear to have been standardised between the compared study conditions. No specific information pertaining to monitoring of compliance with the instruction for participants to refrain from eating and drinking from 21.00 hours on the evening prior to each study period is reported. No specific information pertaining to monitoring of compliance with the instruction to consume only the foods and beverages that were provided for them in the Human Intervention Studies Unit is reported. No specific information pertaining to monitoring of compliance with the instruction not to share foods with others is reported. However, it is judged likely that participants' compliance with one or more of these instructions was monitored, since it is reported that one participant were excluded from the analysis for non‐compliance with the study protocol. However, it is not reported which aspect of the protocol (instruction) was contravened. No further specific instructions were provided to participants, other than the instruction to consume as much of the foods and beverages as desired
Summary of risk of bias Consumption outcome	Unclear risk	Unclear risk

**Table:** Koh 2009

Methods	Study design: between‐subjects randomised controlled trial	
Participants	Setting: laboratory setting Geographical region: University of Toronto at Mississauga, Mississauga, Canada Number of enrolled participants: 57 female undergraduate student and friend or stranger dyads Number (%) of enrolled participants completing the study: 57(100%) Study completers ‐ mean age (SD): 19.2 (1.6) Study completers ‐ sex: female only Study completers ‐ mean BMI kg/m^2^ (SD): 21.6 (3.2) Specific social or cultural characteristics: undergraduate university students Socio‐economic status context: low deprivation Inclusion criteria: female; undergraduate student; enrolled in a first‐year psychology course Exclusion criteria: none reported (Query: "[Participants] were unselected for dietary restraint")	
Interventions	Manipulated product type: food Manipulation: tableware size Duration of exposure to intervention: ≤ 1 day Social setting: selecting and consuming with others Study arms: small plate size (18.2 cm diameter; 260.2 cm^2^ surface area), large serving bowl placed between participant and their partner, eating with friend (partner); small plate size (18.2 cm diameter; 260.2 cm^2^ surface area), 2 smaller serving bowls placed in front of (i) participant and (ii) their partner, eating with friend (partner); small plate size (18.2 cm diameter; 260.2 cm^2^ surface area), 2 smaller serving bowls placed in front of (i) participant and (ii) their partner, eating with stranger (partner); large plate size (23.5 cm diameter; 433.7 cm^2^ surface area), large serving bowl placed between participant and their partner, eating with friend (partner); large plate size (23.5 cm diameter; 433.7 cm^2^ surface area), 2 smaller serving bowls placed in front of (i) participant and (ii) their partner, eating with friend (partner); large plate size (23.5 cm diameter; 433.7 cm^2^ surface area), 2 smaller serving bowls placed in front of (i) participant and (ii) their partner, eating with stranger (partner) Number of comparisons analysed: 1 Comparisons analysed: Intervention 1: small plate size (18.2 cm diameter; 260.2 cm^2^ surface area);*versus* Intervention 2: large plate size (23.5 cm diameter; 433.7 cm^2^ surface area) Concurrent intervention components: no	
Outcomes	Outcomes reported in study: average (mean) amount of pasta self served per person within pair (grams); average (mean) amount of pasta consumed per person within pair (grams) Selection outcome analysed: average (mean) amount of pasta self served per person within pair (grams) Measurement of selection outcome: objective Timing of selection outcome measurement: immediate (≤ 1 day) Consumption outcome analysed: average (mean) amount of pasta consumed per person within pair (grams) Measurement of consumption outcome: objective Timing of consumption outcome measurement: immediate (≤ 1 day)	
Funding source	Not reported	
Notes	Outcome data for 'large serving bowl placed between participant and their partner' and 'two smaller serving bowls placed in front of (i) participant and (ii) their partner', and for 'eating with friend (partner)' and 'eating with stranger (partner)' participant subgroups collapsed and analysed together (one comparison)	
***Risk of bias***		
**Bias**	**Authors' judgement**	**Support for judgement**
Random sequence generation (selection bias)	Unclear risk	Comment: method of sequence generation is not described. Insufficient information about the sequence generation process to permit judgement of 'low risk' or 'high risk'
Allocation concealment (selection bias)	Unclear risk	Comment: method of concealment is not described. Insufficient information to permit judgement of 'low risk' or 'high risk'
Blinding of participants and personnel (performance bias) Selection outcome	Low risk	Quote: "Upon arriving at the laboratory, all four participants were informed that the purpose of the study was to examine the effects of two factors on cognitive test performance. The first factor was described as ''having a proper meal'' (i.e. one that produced ''comfortable satiation''); thus, cognitive test performance would be compared before and after a meal. The second factor was described as ''intimacy level;'' thus, the cognitive performance of those who completed the tests in the presence of a friend would be compared with that of those who completed the tests in the presence of a stranger... Following their assignment to the friend or stranger condition, participants were informed that they would first complete a Pre‐ Meal Questionnaire, followed by the first section of a cognitive test in their food‐deprived states. After the test, they would have a meal of pasta. Following the meal, they would complete a Post‐Meal Questionnaire. Finally, they would complete a second version of the same cognitive test. They were also told that this entire process would be conducted with the friend/stranger with whom they had been paired. Because the true purpose of the study was to examine eating behavior and not cognitive performance, the first cognitive test was a bogus test and the second cognitive test never occurred...Once [participants] had completed [the Post‐Meal Questionnaire, the experiment was over, and they were fully debriefed... One of the items required the participant to rate the ''total amount of food available for both participants'' (1: very small to 5: very big) on a 5‐point Likert Scale. This question was designed primarily to ensure that participants in the sharing and nonsharing conditions perceived the total amount of food to be the same even though the serving bowls were of different sizes. If this is the case, then any effect obtained can be attributed to the manipulation of sharing, which is confounded with serving bowl size... the F‐value for the main effect of sharing was less than one, suggesting that even though the pasta was presented in one large bowlful in the sharing condition and two smaller bowlfuls in the non‐sharing condition, participants in the two conditions perceived the same total amount available." Comment: blinding of study participants attempted and unlikely that the blinding was broken. Very unlikely that key study personnel were blinded, but the review authors judge that the outcome is not likely to be influenced by lack of blinding of key study personnel
Blinding of participants and personnel (performance bias) Consumption outcome	Low risk	Quote: "Upon arriving at the laboratory, all four participants were informed that the purpose of the study was to examine the effects of two factors on cognitive test performance. The first factor was described as ''having a proper meal'' (i.e. one that produced ''comfortable satiation''); thus, cognitive test performance would be compared before and after a meal. The second factor was described as ''intimacy level;'' thus, the cognitive performance of those who completed the tests in the presence of a friend would be compared with that of those who completed the tests in the presence of a stranger... Following their assignment to the friend or stranger condition, participants were informed that they would first complete a Pre‐ Meal Questionnaire, followed by the first section of a cognitive test in their food‐deprived states. After the test, they would have a meal of pasta. Following the meal, they would complete a Post‐Meal Questionnaire. Finally, they would complete a second version of the same cognitive test. They were also told that this entire process would be conducted with the friend/stranger with whom they had been paired. Because the true purpose of the study was to examine eating behavior and not cognitive performance, the first cognitive test was a bogus test and the second cognitive test never occurred...Once [participants] had completed [the Post‐Meal Questionnaire, the experiment was over, and they were fully debriefed... One of the items required the participant to rate the ''total amount of food available for both participants'' (1: very small to 5: very big) on a 5‐point Likert Scale. This question was designed primarily to ensure that participants in the sharing and nonsharing conditions perceived the total amount of food to be the same even though the serving bowls were of different sizes. If this is the case, then any effect obtained can be attributed to the manipulation of sharing, which is confounded with serving bowl size... the F‐value for the main effect of sharing was less than one, suggesting that even though the pasta was presented in one large bowlful in the sharing condition and two smaller bowlfuls in the non‐sharing condition, participants in the two conditions perceived the same total amount available." Comment: blinding of study participants attempted and unlikely that the blinding could have been broken. Very unlikely that key study personnel were blinded, but the review authors judge that the outcome is not likely to be influenced by lack of blinding of key study personnel
Blinding of outcome assessment (detection bias) Selection outcome	Low risk	Comment: no blinding of outcome assessment, but the review authors judge that the outcome measurement is not likely to be influenced by lack of blinding
Blinding of outcome assessment (detection bias) Consumption outcome	Low risk	Comment: no blinding of outcome assessment, but the review authors judge that the outcome measurement is not likely to be influenced by lack of blinding
Incomplete outcome data (attrition bias) Selection outcome	Low risk	Comment: no missing outcome data for selection outcome
Incomplete outcome data (attrition bias) Consumption outcome	Low risk	Comment: no missing outcome data for consumption outcome
Selective reporting (reporting bias)	Unclear risk	Comment: search for record(s) containing details of study protocol conducted in ClinicalTrials.gov and the WHO International Clinical Trials Registry Platform (ICTRP). No records found. Insufficient information to permit judgement of 'low risk' or 'high risk'
Other bias #1 ‐ Baseline comparability of participant characteristics between groups	Low risk	Quote: "The Pre‐Meal Questionnaire contained items that required the participant to rate how well she knew the person with whom she had been paired and how hungry she felt on a 5‐point Likert Scale (1: not at all to 5: very)...Before analyzing the main dependent variables, we analyzed a number of the questionnaire items to ensure that participants assigned to the various conditions were equivalent and to check on the manipulation of some of the independent variables. A 2 (level of acquaintance) x 2 (plate size) x 2 (sharing condition) ANOVA on participants' ages, BMIs, and Restraint scores revealed no significant differences between groups...Next, we examined...[initial hunger score as a variable] that could possibly affect amounts consumed, independent of the variables manipulated in the study...The analysis revealed a significant effect of plate size...Participants who were...assigned to the large plate condition rated themselves as slightly hungrier than those assigned to the small plate condition...we will return to this later... We now return to an issue described in our preliminary analyses. Because there were significant differences between participants on [initial hunger score]... in these analyses... we did [an] additional permutation test, with [this] variable as [a covariate], to see whether differences in the amount served and amount consumed dependent variables could be accounted for by [this variable]. The results...can be easily summarized for the amount of food taken dependent variable; the data reveal the same pattern of significant and nearly significant effects as in the original analyses without covariates." Comment: study uses a between‐subjects design. Evidence of difference between comparison groups in terms of baseline hunger level. However, this difference did not influence measured outcomes (selection and consumption outcomes). No evidence of differences between comparison groups in terms of other measured baseline participant characteristics. Risk of bias due to baseline imbalances between comparison groups is therefore judged low
Other bias #2 ‐ Consistency in intervention delivery	Unclear risk	Quote: "All participants were asked to refrain from eating for 3 h before their scheduled session as they would be eating a meal during the study... [The] experimenter served the meal...informing participants that they would have 20 min to eat the meal and that, during this time, they were free to talk and interact as they would during a normal meal. They were also told that, since there was more than enough food, they were free to help themselves to as much as they wanted. The experimenter reminded them that the goal was to be ''...comfortably full (that is, have a 'proper meal').'' At this point, the experimenter told the participants to ''enjoy the meal.'' The same sequence of events occurred for the pair of participants in each room." Comment: information and instructions provided to participants appear to have been standardised between the compared study conditions. No specific information pertaining to monitoring of compliance with the instruction for participants to refrain from eating for 3 h before their scheduled session is reported. No further specific instructions were provided to participants, other than the instructions that they were free to talk and interact as they would during a normal meal, that they were free to help themselves to as much food as they wanted, and that their goal was to be 'comfortably full'
Summary of risk of bias Selection outcome	Unclear risk	Unclear risk
Summary of risk of bias Consumption outcome	Unclear risk	Unclear risk

**Table:** Kral 2004a

Methods	Study design: within‐subjects randomised controlled trial	
Participants	Setting: laboratory setting Geographical region: local university community, Pennsylvania State University, Pennsylvania, USA Number of enrolled participants: 41 adult females Number (%) of enrolled participants completing the study: 39 (95%) Study completers ‐ mean age (SD): 23.4 (6.2) Study completers ‐ sex: female only Study completers ‐ mean BMI kg/m^2^ (SD): 23.1 (2.6) Specific social or cultural characteristics: none Socio‐economic status context: low deprivation Inclusion criteria: female; aged between 20 and 45 years; in good health; consumes meals at regular intervals; normal weight or overweight (BMI 19 to 29.9 kg/m2); < 20 on Eating Attitudes Test; ≤ 40 on the Zung Self‐Rating Depression Scale; unaware of the purpose of the research Exclusion criteria: current smoker; currently dieting; in athletic training; pregnant or lactating; using medications known to affect food intake or appetite; change in body weight +/‐ 4.5 kg in the previous 6 months; food allergies; food restrictions	
Interventions	Manipulated product type: food Manipulation: portion size Duration of exposure to intervention: ≤ 1 day Social setting: consuming alone Study arms: 500 g portion Italian pasta bake lunch entrée, low energy density (5.23 kJ/g); 500 g portion Italian pasta bake lunch entrée, high energy density (57.32 kJ/g); 700 g portion Italian pasta bake lunch entrée, low energy density (5.23 kJ/g); 700 g portion Italian pasta bake lunch entrée, high energy density (57.32 kJ/g); 900 g portion Italian pasta bake lunch entrée, low energy density (5.23 kJ/g); 900 g portion Italian pasta bake lunch entrée, high energy density (57.32 kJ/g) Number of comparisons analysed: 2 Comparisons analysed: Comparison 1 ‐ Intervention 1: 500 g portion Italian pasta bake lunch entrée;*versus* Intervention 2: 700 g portion Italian pasta bake lunch entrée. Comparison 2 ‐ Intervention 1: 700 g portion Italian pasta bake lunch entrée; *versus* Intervention 2: 900 g portion Italian pasta bake lunch entrée Concurrent intervention components: no	
Outcomes	Outcomes reported in study: total energy intake from breakfast, lunch and dinner meals (kilojoules); energy intake from breakfast meal (kilojoules); energy intake from lunch meal (kilojoules); energy intake from dinner meal (kilojoules); total amount of food consumed from breakfast, lunch and dinner meals (grams); amount of food consumed from breakfast meal (grams); amount of food consumed from lunch meal (grams); amount of food consumed from dinner meal (grams); total amount of beverages consumed from breakfast, lunch and dinner meals (grams); amount of beverages consumed from breakfast meal (grams); amount of beverages consumed from lunch meal (grams); amount of beverages consumed from dinner meal (grams) Selection outcome analysed: N/A Measurement of selection outcome: N/A Timing of selection outcome measurement: N/A Consumption outcome analysed: total energy intake from breakfast, lunch and dinner meals (kilojoules) Measurement of consumption outcome: objective Timing of consumption outcome measurement: immediate (≤ 1 day)	
Funding source	US National Institutes of Health (Grant DK 59853)	
Notes	Incremental comparisons only analysed. Outcome data for low energy density and high energy density participant subgroups collapsed and analysed together (2 comparisons). Author contacted to request information missing from the study report ‐ requested information was not supplied	
***Risk of bias***		
**Bias**	**Authors' judgement**	**Support for judgement**
Random sequence generation (selection bias)	Unclear risk	Comment: method of sequence generation for condition order is not described. Insufficient information about the sequence generation process to permit judgement of 'low risk' or 'high risk'
Allocation concealment (selection bias)	Unclear risk	Comment: method of concealment is not described. Insufficient information to permit judgement of 'low risk' or 'high risk'
Blinding of participants and personnel (performance bias) Consumption outcome	Unclear risk	Quote: "We accepted in the study...women who were unaware of the purpose of the research conducted in the laboratory... To prevent experimental bias, the consent form indicated that the aim of the study was to investigate the effects of food on taste... At the end of their last test day, the women completed a discharge questionnaire. This questionnaire asked the subjects what they thought was the purpose of the study and whether they had noticed any differences between the test days...Only one subject correctly identified that a purpose of the study was to investigate whether the portion size of the lunch entrée affected food intake. Nine subjects (23%) related the purpose of the study either to ratings of hunger and fullness or to ratings of taste or food intake in general. Twenty‐nine subjects (74%) had no knowledge or incorrect knowledge about the purpose of the study. When asked whether they were aware of differences between any of the sessions, 21 subjects (54%) mentioned that they noticed changes in portion size of the lunch entrée; 2 subjects thought incorrectly that the portion sizes at dinner had also changed. Eight subjects reported noticing changes in the composition of the pasta bake, and 3 subjects reported noticing differences in the taste and flavoring of the pasta bake. Ten subjects (26%) did not report noticing any differences between their test days. The effect of portion size and energy density on energy intake was the same regardless of whether the subjects noticed portion‐size differences in the lunch entrée... The subjects' ratings of portion size in relation to their usual portion indicated that they did notice differences in the size of the entrées." Comment: blinding of study participants attempted but blinding was broken in some cases and it is possible that the outcome may be influenced by lack of blinding of study participants (due to potential carry‐over effects between conditions). Participants were probed for suspicion of study purpose or awareness of size manipulation between study conditions. Very unlikely that key study personnel were blinded, but the review authors judge that the outcome is not likely to be influenced by lack of blinding of key study personnel
Blinding of outcome assessment (detection bias) Consumption outcome	Low risk	Comment: no blinding of outcome assessment, but the review authors judge that the outcome measurement is not likely to be influenced by lack of blinding
Incomplete outcome data (attrition bias) Consumption outcome	Low risk	Quote: "Forty five women were recruited for participation in the study. Three subjects withdrew from the study before it started, for personal reasons; one subject did so after her second session. Two subjects were excluded from the analysis because they did not meet the minimum requirements for intake (≥100 g) and ratings of pleasantness of taste (≥35 mm) of the manipulated entrée. Thus, a total of 39 women completed the study". Comment: the second reason for missing outcome data for consumption outcome is the study authors' decision to exclude participants who did not rate pleasantness of taste of the manipulated entrée ≥ 35 mm on a 100 mm visual analogue scale. This reason for missing outcome data is likely to be related to consumption outcome but inclusion could plausibly have biased the estimate of the effect of the intervention on consumption. The review authors judge that the decision to exclude this participant is reasonable, as it is likely to protect against bias in the estimate of the effect of the intervention on consumption. The first reason for missing outcome data for consumption outcome is the study authors' decision to exclude participants with consumption ≤ 99 g from the analysis. The low proportion (1 participant, 2% of study sample) of exclusions due to low consumption means that the review authors judge that the plausible effect size among missing outcomes is unlikely to be enough to have an important impact on the observed effect size
Selective reporting (reporting bias)	Unclear risk	Comment: search for record(s) containing details of study protocol conducted in ClinicalTrials.gov and the WHO International Clinical Trials Registry Platform (ICTRP). No records found. Insufficient information to permit judgement of 'low risk' or 'high risk'
Other bias #1 ‐ Baseline comparability of participant characteristics between groups	Unclear risk	Quote: "Before each meal was served...the subjects completed a series of 100‐mm visual analogue scales (VAS), rating their degree of hunger, thirst, perception of how much they could eat (prospective consumption), nausea, and fullness...There were no significant differences in subjects' ratings of hunger, thirst, prospective consumption, nausea, and fullness across conditions...before ...consumption of breakfast, lunch, and dinner." Comment: study uses a within‐subjects design. Differences between conditions in terms of measured pre‐condition participant 'state' characteristics are reported, but not reported whether there were differences between condition orders in terms of measured pre‐condition participant 'state' characteristics. No analysis of potential differences in measured outcomes between condition orders appears to have been conducted and the statistical analysis of outcome data does not appear to control for condition order. Risk of bias due to period effects is therefore unclear. Insufficient information to permit judgement of 'low risk' or 'high risk'
Other bias #2 ‐ Consistency in intervention delivery	Unclear risk	Quote: "The women were instructed to refrain from eating and drinking (except for water) after 2200 the night before each test day, not to consume alcoholic beverages during the 24 h preceding and throughout their test day, and to maintain similar exercise levels throughout the day...On arrival at the laboratory before each meal, the subjects...completed a questionnaire about...intake of...alcohol in the previous 24 h and any food intake since their last meal. The questionnaire was reviewed for compliance with the study protocol; the women who failed to comply had their test day rescheduled... The subjects were instructed to consume only foods and beverages provided by the laboratory on test days." Comment: information and instructions provided to participants appear to have been standardised between the compared study conditions. Participants' compliance with the instructions to refrain from eating and drinking (except for water) after 22:00 the night before each test day and not to consume alcoholic beverages during the 24 h preceding and throughout their test day was monitored via questionnaire (self report). While no monitoring results are reported with respect to these 2 instructions, it is reported that women who failed to comply had their test day rescheduled and that rescheduling for this reason was infrequent. No specific information pertaining to monitoring of compliance with the instructions for participants to maintain similar exercise levels throughout the day and to consume only foods and beverages provided by the laboratory on test days is reported. No further specific instructions were provided to participants
Summary of risk of bias Consumption outcome	Unclear risk	Unclear risk

**Table:** Kral 2010

Methods	Study design: within‐subjects randomised controlled trial	
Participants	Setting: laboratory setting Geographical region: greater metropolitan area of Philadelphia, PA, USA Number of enrolled participants: 43 children Number (%) of enrolled participants completing the study: 43 (100%) Study completers ‐ mean age (SD): 5.9 (0.6) Study completers ‐ sex: male (51%) and female (49%) Study completers ‐ mean BMI kg/m^2^ (SD): 17.0 (2.5) (BMI); 0.73 (1.10) (BMI z score); 21% overweight; 16% obese Specific social or cultural characteristics: none Socio‐economic status context: low deprivation Inclusion criteria: aged between 5 and 6 years; resident in Greater metropolitan area of Philadelphia; BMI‐for‐age > 5th percentile; likes most foods served in the study (children who rated the majority of the foods with a neutral ("Just okay") or smiling ("Yummy") face at screening visit assessment were invited to participate in the study) Exclusion criteria: serious medical conditions known to affect food intake and body weight; any developmental, medical or psychiatric conditions that might impact study compliance; any food allergies; taking medications known to affect food intake or body weight	
Interventions	Manipulated product type: food Manipulation: portion size Duration of exposure to intervention: ≤ 1 day Social setting: consuming with others Study arms: small size fruit and vegetable portions (75 g broccoli served plain without any butter or seasoning, 75 g carrots served plain without any butter or seasoning and 310 g pasta with tomato sauce, served on a 10¼‐inch diameter 3‐compartment plate; 122 g unsweetened applesauce served in a 12 oz bowl; and 244 g 2% fat milk served in a 300 ml transparent cup with a lid and straw); large size fruit and vegetable portions (150 g broccoli served plain without any butter or seasoning, 150 g carrots served plain without any butter or seasoning and 310 g pasta with tomato sauce, served on a 10¼‐inch diameter 3‐compartment plate; 244 g unsweetened applesauce served in a 12 oz bowl; and 244 g 2% fat milk served in a 300 ml transparent cup with a lid and straw) Number of comparisons analysed: 1 Comparisons analysed: Intervention 1: small size fruit and vegetable portions (75 g broccoli served plain without any butter or seasoning, 75 g carrots served plain without any butter or seasoning and 310 g pasta with tomato sauce, served on a 10¼‐inch diameter 3‐compartment plate; 122 g unsweetened applesauce served in a 12 oz bowl; and 244 g 2% fat milk served in a 300 ml transparent cup with a lid and straw);*versus* Intervention 2: large size fruit and vegetable portions (150 g broccoli served plain without any butter or seasoning, 150 g carrots served plain without any butter or seasoning and 310 g pasta with tomato sauce, served on a 10¼‐inch diameter 3‐compartment plate; 244 g unsweetened applesauce served in a 12‐oz bowl; and 244 g 2% fat milk served in a 300 ml transparent cup with a lid and straw) Concurrent intervention components: no	
Outcomes	Outcomes reported in study: energy intake from 3 fruit and vegetable side dishes (kcal); energy intake from broccoli (kcal); energy intake from carrots (kcal); energy intake from applesauce (kcal); energy intake from pasta entrée (kcal); energy intake from 2% fat milk (kcal); total energy intake from dinner meal (kcal); amount of 3 fruit and vegetable side dishes consumed (grams); amount of broccoli consumed (grams); amount of carrots consumed (grams); amount of applesauce consumed (grams); amount of pasta entrée consumed (grams); amount of 2% milk consumed (grams); total amount consumed from dinner meal (grams); overall energy density of foods consumed at dinner meal (kcal per gram) Selection outcome analysed: N/A Measurement of selection outcome: N/A Timing of selection outcome measurement: N/A Consumption outcome analysed: energy intake from 3 fruit and vegetable side dishes (kcal) Measurement of consumption outcome: objective Timing of consumption outcome measurement: immediate (≤ 1 day)	
Funding source	The Obesity Society (USA)	
Notes	Author contacted to request information missing from the study report ‐ requested information was supplied (January 2014)	
***Risk of bias***		
**Bias**	**Authors' judgement**	**Support for judgement**
Random sequence generation (selection bias)	Unclear risk	Comment: method of sequence generation for condition order is not described. Insufficient information about the sequence generation process to permit judgement of 'low risk' or 'high risk'
Allocation concealment (selection bias)	Unclear risk	Comment: method of concealment is not described. Insufficient information to permit judgement of 'low risk' or 'high risk'
Blinding of participants and personnel (performance bias) Consumption outcome	Unclear risk	Quote: "Details about the purpose of the study were disclosed to families at the end of the study." Comment: blinding of study participants attempted. Not reported whether participants were probed for suspicion of study purpose or awareness of size manipulation between study conditions. It is possible that blinding was broken in some cases and it is possible that the outcome may be influenced by lack of blinding of study participants (due to potential carry‐over effects between conditions). Very unlikely that key study personnel were blinded, but the review authors judge that the outcome is not likely to be influenced by lack of blinding of key study personnel
Blinding of outcome assessment (detection bias) Consumption outcome	Low risk	Comment: no blinding of outcome assessment, but the review authors judge that the outcome measurement is not likely to be influenced by lack of blinding
Incomplete outcome data (attrition bias) Consumption outcome	Low risk	Comment: no missing outcome data for consumption outcome
Selective reporting (reporting bias)	Unclear risk	Comment: search for record(s) containing details of study protocol conducted in ClinicalTrials.gov and the WHO International Clinical Trials Registry Platform (ICTRP). No records found. Insufficient information to permit judgement of 'low risk' or 'high risk'
Other bias #1 ‐ Baseline comparability of participant characteristics between groups	Low risk	Quote: "The fixed factor effects used in all models were portion size condition and time (week). The interaction between portion size condition and time was tested for significance in all models and removed if not significant." Comment: study uses a within‐subjects design. No measurement of participant pre‐condition 'state' characteristics is reported. Analysis of potential differences in measured outcomes between condition orders appears to have been conducted and the statistical analysis appears to control for the potential influence of condition order on measured outcomes ("interaction between portion size condition and time"). It is therefore unlikely that any differences between condition orders in terms of unmeasured pre‐condition participant 'state' characteristics influenced the measured outcomes. Risk of bias due to period effects is therefore judged low
Other bias #2 ‐ Consistency in intervention delivery	Unclear risk	Quote: "On the day of their test session, parents/caretakers were instructed to have their child consume a typical lunch and an afternoon snack (if desired) and not consume any foods or beverages (except water) after 3:00 pm. Upon arrival at the Center at 5:00 pm, parents/caretakers were asked to complete a meal/snack report to ensure that they had complied with the study procedures. At 5:30 pm, dinner was served. Children ate in groups of two to four children in the presence of a research assistant. Children were instructed not to share foods, to remain in their seats once they finished eating, and that they could eat as much or as little as they desired. Children were given 20 min to eat their dinner. The research assistant remained in the room during dinner to ensure that children adhered to the instructions." Comment: information and instructions provided to participants appear to have been standardised between the compared study conditions. Parents'/caretakers' compliance with the instruction to have their child consume a typical lunch and an afternoon snack (if desired) and not consume any foods or beverages (except water) after 3:00 pm on each study visit day was monitored via questionnaire (self report); however, no monitoring results are reported with respect to this instruction. Participants' compliance with the instructions not to share foods and to remain in their seats once they finished eating were monitored by a research assistant present for the duration of the dinner meal time; whilst not explicitly stated, it is likely that compliance with these instructions was maintained by enforcement. No further specific instructions were provided to participants, other than the instruction that they could eat as much or as little as they desired
Summary of risk of bias Consumption outcome	Unclear risk	Unclear risk

**Table:** Leahy 2008

Methods	Study design: within‐subjects randomised controlled trial	
Participants	Setting: field setting Geographical region: USA Number of enrolled participants: 75 children Number (%) of enrolled participants completing the study: 61 (81%) Study completers ‐ mean age (SD): 4.4 (0.6) Study completers ‐ sex: male (49%) and female (51%) Study completers ‐ mean BMI kg/m^2^ (SD): 62.5 (24.6) (BMI percentile); 18.0 (2.7) (body weight, kg) Specific social or cultural characteristics: none Socio‐economic status context: low deprivation Inclusion criteria: aged ≥ 3 years at start of study Exclusion criteria: none reported	
Interventions	Manipulated product type: food Manipulation: portion size Duration of exposure to intervention: ≤ 1 day Social setting: consuming with others Study arms: smaller portion (300 g) of lower energy density (1.2 kcal/g) pasta with cheese and a tomato‐based vegetable sauce entrée served as part of a lunch meal; smaller portion (300 g) of higher energy density (1.6 kcal/g) pasta with cheese and a tomato‐based vegetable sauce entrée served as part of a lunch meal; larger portion (400 g) of lower energy density (1.2 kcal/g) pasta with cheese and a tomato‐based vegetable sauce entrée served as part of a lunch meal; larger portion (400 g) of higher energy density (1.6 kcal/g) pasta with cheese and a tomato‐based vegetable sauce entrée served as part of a lunch meal Number of comparisons analysed: 1 Comparisons analysed: Intervention 1: smaller portion (300 g) of pasta with cheese and a tomato‐based vegetable sauce entrée served as part of a lunch meal;*versus* Intervention 2: larger portion (400 g) of pasta with cheese and a tomato‐based vegetable sauce entrée served as part of a lunch meal Concurrent intervention components: no	
Outcomes	Outcomes reported in study: energy intake from total lunch meal (kcal); energy intake from pasta entrée (kcal); energy intake from vegetables (kcal); energy intake from milk (kcal); energy intake from carrots (kcal); energy intake from applesauce (kcal); total amount consumed from lunch meal (grams); amount consumed from pasta entrée (grams); amount consumed from vegetables (grams); amount consumed from milk (grams); amount consumed from carrots (grams); amount consumed from applesauce (grams) Selection outcome analysed: N/A Measurement of selection outcome: N/A Timing of selection outcome measurement: N/A Consumption outcome analysed: energy intake from total lunch meal (kcal) Measurement of consumption outcome: objective Timing of consumption outcome measurement: immediate (≤ 1 day)	
Funding source	Robert Wood Johnson Foundation (USA)	
Notes	Outcome data for lower energy density and higher energy density participant subgroups collapsed and analysed together (one comparison)	
***Risk of bias***		
**Bias**	**Authors' judgement**	**Support for judgement**
Random sequence generation (selection bias)	Unclear risk	Comment: method of sequence generation for condition order is not described. Insufficient information about the sequence generation process to permit judgement of 'low risk' or 'high risk'
Allocation concealment (selection bias)	Unclear risk	Comment: method of concealment is not described. Insufficient information to permit judgement of 'low risk' or 'high risk'
Blinding of participants and personnel (performance bias) Consumption outcome	Unclear risk	Quote: "During each preference assessment... the child was simultaneously shown two plated portions (400 and 300 g) of the entrée and was asked, "Does one of these plates have more pasta than the other or do they have the same amount of pasta?" The child's responses were recorded... Of the 51 children who participated in the portion size comparisons for the entrée, 27 children (53%) thought that there was no size difference between the 300 and 400 g portions, three children (6%) thought the 300 g portion was >400 g portion, and 21 children (41%) correctly identified the 400 g portion as >300 g portion. The children's ability to recognize the 400 g portion as >300 g portion did not significantly affect the weight of pasta that they consumed." Comment: no blinding or incomplete blinding. Participants were probed for awareness of size manipulation between study conditions. It is possible that the outcome may be influenced by lack of blinding of study participants (due to potential carry‐over effects between conditions). Very unlikely that key study personnel were blinded, but the review authors judge that the outcome is not likely to be influenced by lack of blinding of key study personnel
Blinding of outcome assessment (detection bias) Consumption outcome	Low risk	Comment: no blinding of outcome assessment, but the review authors judge that the outcome measurement is not likely to be influenced by lack of blinding
Incomplete outcome data (attrition bias) Consumption outcome	High risk	Quote: "Twelve children were excluded from the analyses because they failed to meet the predefined minimum consumption criteria: these children ate <25 g of the entrée on three or more occasions. Two children were excluded because of absenteeism." Comment: the second reason for missing outcome data for consumption outcome is the participant absenteeism. This reason for missing outcome data is unlikely to be related to consumption outcome. The first reason for missing outcome data for consumption outcome is the study authors' decision to exclude participants with consumption < 25 g of the entrée on 3 or more occasions from the analysis. The substantial proportion (12 participants, 16% of study sample) of exclusions due to low consumption means that the review authors judge that it is plausible that the effect size among these missing data is enough to have had an important impact on the observed effect size
Selective reporting (reporting bias)	Unclear risk	Comment: search for record(s) containing details of study protocol conducted in ClinicalTrials.gov and the WHO International Clinical Trials Registry Platform (ICTRP). No records found. Insufficient information to permit judgement of 'low risk' or 'high risk'
Other bias #1 ‐ Baseline comparability of participant characteristics between groups	Unclear risk	Comment: study uses a within‐subjects design. No measurement of participant pre‐condition 'state' characteristics is reported. No analysis of potential differences in measured outcomes between condition orders appears to have been conducted and the statistical analysis of outcome data does not appear to control for condition order. Risk of bias due to period effects is therefore unclear. Insufficient information to permit judgement of 'low risk' or 'high risk'
Other bias #2 ‐ Consistency in intervention delivery	Low risk	Quote: "Teachers were instructed not to encourage children to eat and not to discuss food. Food and drink spillage and any comments made by children or teachers pertaining to food were recorded by trained observers. Conversations about food‐related topics were redirected to minimize the influence of teachers' and peers' comments on children's lunch intake." Comment: information and instructions provided to participants appear to have been standardised between the compared study conditions. Teachers' compliance with the instruction not to encourage children to eat and not to discuss food was monitored by trained observers; whilst no monitoring results are reported with respect to this instruction, it is likely that any potential effect‐modifying influences of non‐compliance were minimised by trained observers redirecting conversations about food‐related topics that followed teachers' or peers' comments. No further specific instructions were provided to participants or providers
Summary of risk of bias Consumption outcome	Unclear risk	Unclear risk

**Table:** Levitsky 2004

Methods	Study design: within‐subjects randomised controlled trial	
Participants	Setting: laboratory setting Geographical region: Cornell University, Ithaca, NY, USA Number of enrolled participants: 13 undergraduate students Number (%) of enrolled participants completing the study: 13 (100%) Study completers ‐ mean age (SD): 23.0 (8.6) Study completers ‐ sex: male (69%) and female (31%) Study completers ‐ mean BMI kg/m^2^ (SD): 23.2 (2.9) Specific social or cultural characteristics: undergraduate university students Socio‐economic status context: low deprivation Inclusion criteria: undergraduate student Exclusion criteria: allergies to study foods; dietary restraint score < 30	
Interventions	Manipulated product type: food Manipulation: portion size Duration of exposure to intervention: ≤ 1 day Social setting: unclear Study arms: 100% portion size (vegetable soup, rigatoni pasta and tomato sauce, breadsticks and ice cream); 125% Portion size (vegetable soup, rigatoni pasta and tomato sauce, breadsticks and ice cream); 150% portion size (vegetable soup, rigatoni pasta and tomato sauce, breadsticks and ice cream) Number of comparisons analysed: 2 Comparisons analysed: comparison 1 ‐ Intervention 1: 100% portion size (vegetable soup, rigatoni pasta and tomato sauce, breadsticks and ice cream);*versus* Intervention 2: 125% portion size (vegetable soup, rigatoni pasta and tomato sauce, breadsticks and ice cream). Comparison 2 ‐ Intervention 1: 125% portion size (vegetable soup, rigatoni pasta and tomato sauce, breadsticks and ice cream);*versus* Intervention 2: 150% portion size (vegetable soup, rigatoni pasta and tomato sauce, breadsticks and ice cream) Concurrent intervention components: no	
Outcomes	Outcomes reported in study: energy intake from total lunch meal (kcal); energy intake from vegetable soup (kcal); energy intake from rigatoni pasta and tomato sauce (kcal); energy intake from breadsticks (kcal); energy intake from ice cream (kcal); amount of lunch meal consumed (grams) Selection outcome analysed: N/A Measurement of selection outcome: N/A Timing of selection outcome measurement: N/A Consumption outcome analysed: energy intake from total lunch meal (kcal) Measurement of consumption outcome: objective Timing of consumption outcome measurement: immediate (≤ 1 day)	
Funding source	Not reported	
Notes	Outcome data for lower energy density and higher energy density participant subgroups collapsed and analysed together (one comparison). Increments only analysed. Author contacted to request information missing from the study report ‐ requested information was supplied (February 2014)	
***Risk of bias***		
**Bias**	**Authors' judgement**	**Support for judgement**
Random sequence generation (selection bias)	Unclear risk	Comment: method of sequence generation for condition order is not described. Insufficient information about the sequence generation process to permit judgement of 'low risk' or 'high risk'
Allocation concealment (selection bias)	Unclear risk	Comment: method of concealment is not described. Insufficient information to permit judgement of 'low risk' or 'high risk'
Blinding of participants and personnel (performance bias) Consumption outcome	Unclear risk	Quote: "The subjects were deceived into thinking that the study was about taste enhancers and the perception of certain foods. They received a debriefing session after the study." Comment: no blinding or incomplete blinding. Not reported whether participants were probed for suspicion of study purpose or awareness of size manipulation between study conditions. It is possible that the outcome may be influenced by lack of blinding of study participants (due to potential carry‐over effects between conditions). Very unlikely that key study personnel were blinded, but the review authors judge that the outcome is not likely to be influenced by lack of blinding of key study personnel
Blinding of outcome assessment (detection bias) Consumption outcome	Low risk	Comment: no blinding of outcome assessment, but the review authors judge that the outcome measurement is not likely to be influenced by lack of blinding
Incomplete outcome data (attrition bias) Consumption outcome	Low risk	Comment: no missing outcome data for consumption outcome
Selective reporting (reporting bias)	Unclear risk	Comment: search for record(s) containing details of study protocol conducted in ClinicalTrials.gov and the WHO International Clinical Trials Registry Platform (ICTRP). No records found. Insufficient information to permit judgement of 'low risk' or 'high risk'
Other bias #1 ‐ Baseline comparability of participant characteristics between groups	Low risk	Quote: "...[Subjects] completed a 7‐point hunger rating scale before and after eating...No interactions between portion size and test day were observed." Comment: not reported whether there were differences between condition orders in terms of measured baseline participant 'state' characteristic. No analysis of potential differences in measured outcomes between condition orders appears to have been conducted but the statistical analysis appears to control for the potential influence of condition order on measured outcomes ("interaction between portion size and test day"). It is therefore unlikely that any differences between condition orders in terms of unmeasured pre‐condition participant 'state' characteristics influenced the measured outcomes. Risk of bias due to period effects is therefore judged low
Other bias #2 ‐ Consistency in intervention delivery	Unclear risk	Quote: "Subjects were asked to eat the same foods and maintain the same level of activity they exhibited in wk 1 throughout wk 2 of testing." Comment: information and instructions provided to participants appear to have been standardised between the compared study conditions. No information pertaining to monitoring of participants' compliance with the instruction to eat the same foods and maintain the same level of activity they exhibited in week 1 throughout week 2 of testing is reported. No further specific instructions were provided to participants with respect to week 2 of testing
Summary of risk of bias Consumption outcome	Unclear risk	Unclear risk

**Table:** Looney 2011

Methods	Study design: within‐subjects cluster‐randomised controlled trial Unit of allocation: classroom Unit of analysis: individual Number of clusters: 2 Number of participants per cluster: not reported Analysis appears to include a covariate to account for cluster allocation. Repeated measures analyses of covariance with the within‐subject factors of portion size and energy density and order as a covariate. Only 2 classes, so 'order' is equivalent to 'classroom'	
Participants	Setting: field setting, Early Learning Center on the University of Tennessee Knoxville campus Geographical region: University of Tennessee Knoxville campus, Tennessee, USA Number of enrolled participants: 21 children Number (%) of enrolled participants completing the study: 17 (81%) Study completers ‐ mean age (SD): 3.8 (0.6) Study completers ‐ sex: male (41%) and female (59%) Study completers ‐ mean BMI kg/m^2^ (SD): 0.01 (1.06) (BMI z score); 50.2 (32.4) (BMI percentile); 29% overweight Specific social or cultural characteristics: none Socio‐economic status context: low deprivation Inclusion criteria: aged 2 to 5 years; attending full day pre‐school Exclusion criteria: unable to use a spoon (caregiver report); lactose intolerant; allergies to study foods; dislike of study foods	
Interventions	Manipulated product type: food Manipulation: portion size Duration of exposure to intervention: ≤ 1 day Social setting: consuming with others Study arms: small portion snack ‐ 150 g unsweetened apple sauce and chocolate pudding made with 2% fat milk; large portion snack ‐ 300 g unsweetened apple sauce and chocolate pudding made with 2% fat milk Number of comparisons analysed: 1 Comparisons analysed: Intervention 1: small portion snack ‐ 150 g unsweetened apple sauce and chocolate pudding made with 2% fat milk;*versus* Intervention 2: large portion snack ‐ 300 g unsweetened apple sauce and chocolate pudding made with 2% fat milk Concurrent intervention components: no	
Outcomes	Outcomes reported in study: total energy intake from snack foods (kcal); energy intake from applesauce (kcal); energy intake from chocolate pudding made with 2% fat milk (kcal); amount of snack foods consumed (grams); amount of applesauce consumed (grams); amount of chocolate pudding made with 2% fat milk consumed (grams) Selection outcome analysed: N/A Measurement of selection outcome: N/A Timing of selection outcome measurement: N/A Consumption outcome analysed: total energy intake from snack foods (kcal) Measurement of consumption outcome: objective Timing of consumption outcome measurement: immediate (≤ 1 day)	
Funding source	No funding to disclose	
Notes	—	
***Risk of bias***		
**Bias**	**Authors' judgement**	**Support for judgement**
Random sequence generation (selection bias)	Low risk	Comment: author contact: (13/3/13) "Yes the orders were randomized. We simply flipped a coin to assign order to the classroom one (head = order 1, tails = order 2). The second Classroom by default was the order not assigned to classroom one."
Allocation concealment (selection bias)	Unclear risk	Comment: participating classrooms appear to have been randomised to condition order concurrently. However, it is unclear whether randomised to condition order occurred before or after consent for individuals' participation had been obtained. The review authors therefore judge that there is insufficient information to permit judgement of 'low risk' or 'high risk'
Blinding of participants and personnel (performance bias) Consumption outcome	Unclear risk	Comment: no blinding or incomplete blinding. Not reported whether participants were probed for suspicion of study purpose or awareness of size manipulation between study conditions. It is possible that the outcome may be influenced by lack of blinding of study participants (due to potential carry‐over effects between conditions). Very unlikely that key study personnel were blinded, but the review authors judge that the outcome is not likely to be influenced by lack of blinding of key study personnel
Blinding of outcome assessment (detection bias) Consumption outcome	Low risk	Comment: no blinding of outcome assessment, but the review authors judge that the outcome measurement is not likely to be influenced by lack of blinding
Incomplete outcome data (attrition bias) Consumption outcome	High risk	Quote: "Although 21 children completed all sessions of the study, 4 children were excluded from the analyses because they consumed <5 kcal in at least one session." Comment: the reason for missing outcome data for consumption outcome is the study authors' decision to exclude participants with consumption < 5 kcal in at least one session from the analysis. The substantial proportion (4 participants, 19% of study sample) of exclusions due to low consumption means that the review authors judge that it is plausible that the effect size among these missing data is enough to have had an important impact on the observed effect size
Selective reporting (reporting bias)	Low risk	Comment: search for record(s) containing details of study protocol conducted in ClinicalTrials.gov and the WHO International Clinical Trials Registry Platform (ICTRP). Record found in ClinicalTrials.gov and duplicate record found in ICTRP (Identifier: NCT00936507). Comparison of ClinicalTrials.gov/ICTRP records with published study report indicates no selective outcome reporting
Other bias #1 ‐ Baseline comparability of participant characteristics between groups	Low risk	Quote: "Liking of each food was assessed with the aid of a trained research assistant before each snack was served at each session, using a three‐point Likert‐type scale... The hunger of children was assessed with the aid of trained research assistants before each snack was served at each session with a tool developed by Birch...and used in previous studies......Repeated measures analyses of covariance with the within‐subject factors of portion size and energy density and order as a covariate were also used to assess the dependent variables grams/energy of food consumed." Comment: study uses a within‐subjects design. Not reported whether there were differences between condition orders in terms of measured pre‐condition participant 'state' characteristic. However, the statistical analysis appears to control for the potential influence of condition order on measured outcomes. It is therefore unlikely that any differences between condition orders in terms of unmeasured pre‐condition participant 'state' characteristics influenced the measured outcomes. Risk of bias due to period effects is therefore judged low
Other bias #2 ‐ Consistency in intervention delivery	Unclear risk	Quote: "Preportioned snacks, as typically served at the Early Learning Center, were passed out and children were asked not to share their snack and to eat as much or as little of their snack as desired. Children sat at the table with a classroom attendant, which was standard procedures at the Early Learning Center, and a research assistant while they consumed their snack until reported being done." Comment: information and instructions provided to participants appear to have been standardised between the compared study conditions. Whilst not explicitly stated, it is likely that compliance with the instruction for children not to share their snack was monitored by the research assistant seated at the table for the duration of each study session; however, no monitoring results are reported with respect to this instruction. No further specific instructions were provided to participants, other than the instruction to eat as much or as little of their snack as desired
Summary of risk of bias Consumption outcome	Unclear risk	Unclear risk

**Table:** Marchiori 2011

Methods	Study design: between‐subjects randomised controlled trial	
Participants	Setting: laboratory setting Geographical region: Université Libre de Bruxelles, Brussels, Belgium Number of enrolled participants: 54 undergraduate students Number (%) of enrolled participants completing the study: 33 (61%) Study completers ‐ mean age (SD): 20.3 (2.0) Study completers ‐ sex: male (12%) and female (88%) Study completers ‐ mean BMI kg/m^2^ (SD): 21.7 (3.7) Specific social or cultural characteristics: undergraduate university psychology students Socio‐economic status context: low deprivation Inclusion criteria: undergraduate psychology student Exclusion criteria: presence of food allergies; weight problems; overweight (BMI > 25); dieting behaviour; personal food intake control in order to gain or lose weight	
Interventions	Manipulated product type: food Manipulation: individual unit size Duration of exposure to intervention: ≤ 1 day Social setting: consuming alone Study arms: 90 g half‐size candies (sweets), comprising 20 half‐size (2 g) cherry‐shaped gummy candies and 20 half‐size (2.5 g) sweet‐sour red gummy ribbons; 90 g full‐size candies (sweets), comprising 10 full‐size (4 g) cherry‐shaped gummy candies and 10 full‐size (5 g) sweet‐sour red gummy ribbons Number of comparisons analysed: 1 Comparisons analysed: Intervention 1: 90 g half‐size candies (sweets), comprising 20 half‐size (2 g) cherry‐shaped gummy candies and 20 half‐size (2.5 g) sweet‐sour red gummy ribbons;*versus* Intervention 2: 90 g full‐size candies (sweets), comprising 10 full‐size (4 g) cherry‐shaped gummy candies and 10 full‐size (5 g) sweet‐sour red gummy ribbons Concurrent intervention components: no	
Outcomes	Outcomes reported in study: energy intake from snack (kcal); amount of candies consumed (grams); number of candies consumed (N) Selection outcome analysed: N/A Measurement of selection outcome: N/A Timing of selection outcome measurement: N/A Consumption outcome analysed: energy intake from snack (kcal) Measurement of consumption outcome: objective Timing of consumption outcome measurement: immediate (≤ 1 day)	
Funding source	Ministère luxembourgeois de la Culture, de l'Enseignement Supérieur et de la Recherche Grant (AFR 07/052)	
Notes	—	
***Risk of bias***		
**Bias**	**Authors' judgement**	**Support for judgement**
Random sequence generation (selection bias)	Unclear risk	Comment: method of sequence generation is not described. Insufficient information about the sequence generation process to permit judgement of 'low risk' or 'high risk'
Allocation concealment (selection bias)	Unclear risk	Comment: method of concealment is not described. Insufficient information to permit judgement of 'low risk' or 'high risk'
Blinding of participants and personnel (performance bias) Consumption outcome	Low risk	Quote: "The experiment was conducted during an unrelated computerized experiment (decision‐making task about four objects after sequential information presentation). Participants were seated in individual cubicles...Participants were told that the candies were offered for free consumption in recognition for their participation and that they could eat as much as they wanted... After the conclusion of the experiment, participants were given a questionnaire in which they were told that the candies were actually part of an experiment about eating habits. To avoid cueing participants to the issue of food intake, consumption was not experimentally induced nor were pre‐meal hunger ratings assessed." Comment: blinding of study participants attempted and unlikely that the blinding could have been broken. Very unlikely that key study personnel were blinded, but the review authors judge that the outcome is not likely to be influenced by lack of blinding of key study personnel
Blinding of outcome assessment (detection bias) Consumption outcome	Low risk	Comment: no blinding of outcome assessment, but the review authors judge that the outcome measurement is not likely to be influenced by lack of blinding
Incomplete outcome data (attrition bias) Consumption outcome	High risk	Comment: the reason for missing outcome data for consumption outcome is the study authors' decision to exclude participants with zero consumption from the analysis. The substantial proportion (21 participants, 39% of study sample) of exclusions due to zero consumption and the differential distribution between arms means that the review authors judge that it is plausible that the effect size among these missing data is enough to have had an important impact on the observed effect size
Selective reporting (reporting bias)	Unclear risk	Comment: search for record(s) containing details of study protocol conducted in ClinicalTrials.gov and the WHO International Clinical Trials Registry Platform (ICTRP). No records found. Insufficient information to permit judgement of 'low risk' or 'high risk'
Other bias #1 ‐ Baseline comparability of participant characteristics between groups	Low risk	Quote: "To avoid cueing participants to the issue of food intake, consumption was not experimentally induced nor were premeal hunger ratings assessed. However, a retrospective measure of prestudy hunger was taken and used as a covariate in the analyses ... Using 7‐point Likert scales, participants rated their prestudy hunger, their liking of the candies, the extent to which they consumed candies on a regular basis, and the extent to which they controlled their food intake...Finally, they reported exercise frequency (hours/week)...Demographic measures were: age, sex, nationality, weight, height, primary language, and dieting behavior... Analysis of variance was used to examine differences between food‐item size conditions [in terms of all measured baseline participant characteristics]. No statistically significant differences were observed between conditions...There were no significant differences across conditions of food‐item size in ratings of hunger, liking of the candies, eating candies on a regular basis, and estimates of the price and energy content (kcal) of the entire plate...which suggests that random assignment was successful (see Table)." Comment: study uses a between‐subjects design. No differences between comparison groups in terms of measured baseline participant characteristics
Other bias #2 ‐ Consistency in intervention delivery	Low risk	Quote: "Participants were told that the candies were offered for free consumption in recognition for their participation and that they could eat as much as they wanted. Participants were asked to not take any food out, which was further ensured by the experimenter." Comment: information and instructions provided to participants appear to have been standardised between the compared study conditions. Participants' compliance with the instruction not to take any food out was monitored and enforced by the experimenter. No further specific instructions were provided to participants, other than the instruction that they could eat as much as they wanted
Summary of risk of bias Consumption outcome	Unclear risk	Unclear risk

**Table:** Marchiori 2012a

Methods	Study design: between‐subjects randomised controlled trial	
Participants	Setting: laboratory setting Geographical region: Université Libre de Bruxelles, Brussels, Belgium Number of enrolled participants: 58 undergraduate students Number (%) of enrolled participants completing the study: 58 (100%) Study completers ‐ mean age (SD): 19.9 (1.9) Study completers ‐ sex: male (29%) and female (71%) Study completers ‐ mean BMI kg/m^2^ (SD): 22.5 (4.3) Specific social or cultural characteristics: undergraduate university students Socio‐economic status context: low deprivation Inclusion criteria: undergraduate student Exclusion criteria: none reported	
Interventions	Manipulated product type: food Manipulation: comparison 1 ‐ portion size; comparison 2 ‐ package size Duration of exposure to intervention: ≤ 1 day Social setting: consuming alone Study arms: medium portion of M&Ms (200 g) served in a small container (250 ml ‐ 6.5 cm wide, 9 cm long and 3.5 cm deep); medium portion of M&Ms (200 g) served in a large container (750 ml ‐ 9.9 cm wide, 16.3 cm long and 4.3 cm deep); large portion of M&Ms (600 g) served in a large container (750 ml ‐ 9.9 cm wide, 16.3 cm long and 4.3 cm deep) Number of comparisons analysed: 2 Comparisons analysed: comparison 1 ‐ Intervention 1: medium portion of M&Ms (200 g) served in a large container (750 ml ‐ 9.9 cm wide, 16.3 cm long and 4.3 cm deep);*versus* Intervention 2: large portion of M&Ms (600 g) served in a large container (750 ml ‐ 9.9 cm wide, 16.3 cm long and 4.3 cm deep). Comparison 2 ‐ Intervention 1: medium portion of M&Ms (200 g) served in a small container (250 ml ‐ 6.5 cm wide, 9 cm long and 3.5 cm deep); *versus* Intervention 2: medium portion of M&Ms (200 g) served in a large container (750 ml ‐ 9.9 cm wide, 16.3 cm long and 4.3 cm deep) Concurrent intervention components: yes. 22‐minute TV show (Scrubs, Season 1, Episode 1) ‐ provided to both the intervention and comparator groups	
Outcomes	Outcomes reported in study: energy intake from M&Ms (kcal); energy intake from M&Ms (MJ); amount of M&Ms consumed (grams) Selection outcome analysed: N/A Measurement of selection outcome: N/A Timing of selection outcome measurement: N/A Consumption outcome analysed: energy intake from M&Ms (kcal) Measurement of consumption outcome: objective Timing of consumption outcome measurement: immediate (≤ 1 day)	
Funding source	National Research Fund (Luxembourg)	
Notes	Author contacted to request information missing from the study report ‐ requested information was supplied (February 2014)	
***Risk of bias***		
**Bias**	**Authors' judgement**	**Support for judgement**
Random sequence generation (selection bias)	Unclear risk	Comment: method of sequence generation is not described. Insufficient information about the sequence generation process to permit judgement of 'low risk' or 'high risk'
Allocation concealment (selection bias)	Unclear risk	Comment: method of concealment is not described. Insufficient information to permit judgement of 'low risk' or 'high risk'
Blinding of participants and personnel (performance bias) Consumption outcome	Low risk	Quote: "The study was advertised as examining the effects of snack food consumption on information processing. It was run from 2 pm to 6 pm in individual cubicles in a psychology laboratory." Comment: blinding of study participants attempted and unlikely that the blinding could have been broken. Very unlikely that key study personnel were blinded, but the review authors judge that the outcome is not likely to be influenced by lack of blinding of key study personnel
Blinding of outcome assessment (detection bias) Consumption outcome	Low risk	Comment: no blinding of outcome assessment, but the review authors judge that the outcome measurement is not likely to be influenced by lack of blinding
Incomplete outcome data (attrition bias) Consumption outcome	Low risk	Comment: no missing outcome data for consumption outcome
Selective reporting (reporting bias)	Unclear risk	Comment: search for record(s) containing details of study protocol conducted in ClinicalTrials.gov and the WHO International Clinical Trials Registry Platform (ICTRP). No records found. Insufficient information to permit judgement of 'low risk' or 'high risk'
Other bias #1 ‐ Baseline comparability of participant characteristics between groups	Low risk	Quote: "Before...consumption, participants used visual analog scales (VAS) to rate their hunger, prospective consumption (how much food they thought they could eat) and fullness... Liking of foods was also assessed before...consumption with VAS by having participants take one M&M and rate pleasantness of taste, appearance and quality... Plate cleaning tendency was assessed with the same question used by Rolls, Roe, Kral, Meengs, and Wall...and the two questions used by Wansink and colleagues... Mood was measured with the two items used by Wansink and Kim...and the four items used by Reinbach, Martinussen, and Møller...Plate cleaning tendency, consumption monitoring and mood were translated into French and assessed on agreement scales anchored (‐3) strongly disagree and (+3) strongly agree. Dieting behavior was assessed with the French translation...of the Eating Attitude Test ... Binge eating was assessed by a question from the Eating Disorders Examination...: ''Have there been any times when you have eaten a large amount of food in a short amount of time and you had a sense of loss of control about your eating?'' Demographics measured were: age, weight [and] height...There were no significant differences across conditions in ratings of participant characteristics (...see Table 1)." Comment: study uses a between‐subjects design. No differences between comparison groups in terms of measured baseline participant characteristics
Other bias #2 ‐ Consistency in intervention delivery	Low risk	Comment: information provided to participants appears to have been standardised between the compared study conditions. No specific instructions were provided to participants and therefore participants' compliance with instructions is not applicable
Summary of risk of bias Consumption outcome	Unclear risk	Unclear risk

**Table:** Marchiori 2012c

Methods	Study design: between‐subjects randomised controlled trial	
Participants	Setting: field setting, elementary school Geographical region: Brussels, Belgium Number of enrolled participants: 85 children Number (%) of enrolled participants completing the study: 77 (91%) Study completers ‐ mean age (SD): 9.2 (2.5) Study completers ‐ sex: male (45%) and female (55%) Study completers ‐ mean BMI kg/m^2^ (SD): 41.1 (20.9) (BMI percentile); 29.9 (8.9) (body weight, kg); 0% overweight; 0% obese Specific social or cultural characteristics: none Socio‐economic status context: low deprivation Inclusion criteria: first or sixth grade elementary school student Exclusion criteria: presence of food allergies; overweight (BMI ≥ 85th percentile); weight problems; dieting behaviour; food intake control in order to gain or lose weight; lack of hunger	
Interventions	Manipulated product type: food Manipulation: individual unit size Duration of exposure to intervention: ≤ 1 day Social setting: consuming with others Study arms: small size cookies ‐ 36 half‐sized cookies, 126 g total, 3.5 g each, 3.7 cm long (1.45 in), 2.3 cm wide (0.9 in) and 1.1 cm high (0.4 in), rectangular and consisting of several layers of wafers filled with milk chocolate topping; large size cookies ‐ 18 full‐sized cookies, 126 g total, 7.0 g each, 7.4 cm long (2.9 in), 2.3 cm wide (0.9 in) and 1.1 cm high (0.4 in), rectangular and consisting of several layers of wafers filled with milk chocolate topping Number of comparisons analysed: 1 Comparisons analysed: Intervention 1: small size cookies ‐ 36 half‐sized cookies, 126 g total, 3.5 g each, 3.7 cm long (1.45 in), 2.3 cm wide (0.9 in) and 1.1 cm high (0.4 in), rectangular and consisting of several layers of wafers filled with milk chocolate topping;*versus* Intervention 2: large size cookies ‐ 18 full‐sized cookies, 126 g total, 7.0 g each, 7.4 cm long (2.9 in), 2.3 cm wide (0.9 in) and 1.1 cm high (0.4 in), rectangular and consisting of several layers of wafers filled with milk chocolate topping Concurrent intervention components: no	
Outcomes	Outcomes reported in study: energy intake from cookies (kcal); amount of cookies consumed (grams); number of cookies consumed (N) Selection outcome analysed: N/A Measurement of selection outcome: N/A Timing of selection outcome measurement: N/A Consumption outcome analysed: energy intake from cookies (kcal) Measurement of consumption outcome: objective Timing of consumption outcome measurement: immediate (≤ 1 day)	
Funding source	National Research Fund (Luxembourg)	
Notes	Author contacted to request information missing from the study report ‐ requested information was supplied (February 2014)	
***Risk of bias***		
**Bias**	**Authors' judgement**	**Support for judgement**
Random sequence generation (selection bias)	Unclear risk	Comment: method of sequence generation is not described. Insufficient information about the sequence generation process to permit judgement of 'low risk' or 'high risk'
Allocation concealment (selection bias)	Unclear risk	Comment: method of concealment is not described. Insufficient information to permit judgement of 'low risk' or 'high risk'
Blinding of participants and personnel (performance bias) Consumption outcome	Low risk	Quote: "The purpose of the study was referred to guardians as examining their children's food preferences and eating habits with no mention of assessing food intake...Children were called up in alphabetical order...and were randomly assigned to a room and table." Comment: blinding of study participants attempted and unlikely that the blinding could have been broken. Very unlikely that key study personnel were blinded, but the review authors judge that the outcome is not likely to be influenced by lack of blinding of key study personnel
Blinding of outcome assessment (detection bias) Consumption outcome	Low risk	Comment: no blinding of outcome assessment, but the review authors judge that the outcome measurement is not likely to be influenced by lack of blinding
Incomplete outcome data (attrition bias) Consumption outcome	Low risk	Quote: "Exclusion criteria were determined in view of the moderating effect of these variables: presence of food allergies, overweight, weight problems, dieting behavior, food intake control in order to gain or lose weight, and lack of hunger. As a result, data from 77 children (out of 85) were analysed." Comment: reasons for exclusion from analysis are per protocol and therefore do not raise concerns about risk of attrition bias due to handing of exclusions
Selective reporting (reporting bias)	Unclear risk	Comment: search for record(s) containing details of study protocol conducted in ClinicalTrials.gov and the WHO International Clinical Trials Registry Platform (ICTRP). No records found. Insufficient information to permit judgement of 'low risk' or 'high risk'
Other bias #1 ‐ Baseline comparability of participant characteristics between groups	Low risk	Quote: "Children ...reported prestudy hunger (4‐point scale labelled ''not at all,'' ''a little,'' ''fairly,'' and ''a lot'')...Questionnaires were sent home to guardians, where they reported on the following variables regarding their children: sex, birth date, nationality, weight, height, dieting behavior (''Is your child currently on a diet to lose weight? (Y/N)''), food intake control, possible food allergies or weight problems, and child's preferred afternoon snack. Body mass index (BMI) percentile was calculated with age‐ and sex‐specific reference data. Overweight was defined according to United States Centers for Disease Control and Prevention guidelines as BMI ≥85th percentile... Exclusion criteria were determined in view of the moderating effect of these variables: presence of food allergies, overweight, weight problems, dieting behavior, food intake control in order to gain or lose weight, and lack of hunger... On‐site, children rated liking of the cookies (3‐point scale labeled ''not good'' ''ok,'' ''good''), habit of eating cookies as afternoon snack (Y/N), and exercise frequency (hours/week).Fixed factors in the model were...sex and age... There were no significant differences across conditions of [food intake size], sex, and age...in ratings of hunger, liking of the cookies, and habit of eating cookies as an afternoon snack." Comment: no differences between comparison groups in terms of measured baseline participant characteristics. The statistical analysis of outcome data controls for any differences between comparison groups in terms age and sex
Other bias #2 ‐ Consistency in intervention delivery	Low risk	Quote: "Children were told they could eat as much or as little as desired and were informed they would be given a refill if they wanted. They were allowed to talk but not to share their food. Experimenters ensured that the food was not shared, and if it was not consumed, it was left on the table." Comment: information and instructions provided to participants appear to have been standardised between the compared study conditions. Participants' compliance with the instruction not to share their food was monitored and enforced by experimenters. No further specific instructions were provided to participants, other than the instruction that they could eat as much or as little as desired and would be given a refill if they wanted
Summary of risk of bias Consumption outcome	Unclear risk	Unclear risk

**Table:** Mathias 2012

Methods	Study design: within‐subjects cluster‐randomised controlled trial Unit of allocation: classroom Unit of analysis: individual Number of clusters: not reported Number of participants per cluster: 2 to 3 Analysis does not appear to account for cluster allocation, as the statistical model does not appear to include any covariate related to cluster assignment	
Participants	Setting: laboratory setting Geographical region: greater metropolitan area of Philadelphia, PA, USA Number of enrolled participants: 38 children Number (%) of enrolled participants completing the study: 30 (79%) Study completers ‐ mean age (SD): 5.4 (1.1) Study completers ‐ sex: male (40%) and female (60%) Study completers ‐ mean BMI kg/m^2^ (SD): 72.3 (29.6) (BMI percentile); 50% overweight or obese Specific social or cultural characteristics: none Socio‐economic status context: low deprivation Inclusion criteria: aged between 4 and 6 years; rated the main entrée as tasting "yummy" or "just okay" Exclusion criteria: dislike of the study main entrée; dislike of both the study fruit and the study vegetable side dishes; severe food allergies; chronic illnesses; conditions affecting food intake; receiving a special diet	
Interventions	Manipulated product type: food Manipulation: portion size Duration of exposure to intervention: ≤ 1 day Social setting: consuming with others Study arms: small size fruit portion (75 g drained canned peaches in light syrup), small size vegetable portion (75 g cooked broccoli with 3 g added butter for every 72 g cooked broccoli) served as part of a dinner meal; small size fruit portion (75 g drained canned peaches in light syrup), large size vegetable portion (150 g cooked broccoli with 3 g added butter for every 72 g cooked broccoli) served as part of a dinner meal; large size fruit portion (150 g drained canned peaches in light syrup), small size vegetable portion (75 g cooked broccoli with 3 g added butter for every 72 g cooked broccoli) served as part of a dinner meal; large size fruit portion (150 g drained canned peaches in light syrup), large size vegetable portion (75 g cooked broccoli with 3 g added butter for every 72 g cooked broccoli) served as part of a dinner meal Number of comparisons analysed: 1 Comparisons analysed: Intervention 1: small size fruit portion (75g drained canned peaches in light syrup) with either small (75 g) or large (150 g) size vegetable portion (cooked broccoli with 3 g added butter for every 72 g cooked broccoli) served as part of a dinner meal;*versus* Intervention 2: large size fruit portion (150 g drained canned peaches in light syrup) with either small (75 g) or large (150 g) size vegetable portion (cooked broccoli with 3 g added butter for every 72 g cooked broccoli) served as part of a dinner meal Concurrent intervention components: no	
Outcomes	Outcomes reported in study: energy intake from total dinner meal (kcal); energy intake from fruit side dish (kcal); energy intake from vegetable side dish (kcal); amount of food consumed from total dinner meal (grams); amount of fruit side dish consumed (grams); amount of vegetable side dish consumed (grams) Selection outcome analysed: N/A Measurement of selection outcome: N/A Timing of selection outcome measurement: N/A Consumption outcome analysed: energy intake from total dinner meal (kcal) Measurement of consumption outcome: objective Timing of consumption outcome measurement: immediate (≤ 1 day)	
Funding source	US National Institutes of Health (Grant R01 DK071095)	
Notes	Outcome data for small (75 g) and large (150 g) size vegetable portion participant subgroups collapsed and analysed together (one comparison)	
***Risk of bias***		
**Bias**	**Authors' judgement**	**Support for judgement**
Random sequence generation (selection bias)	Unclear risk	Comment: method of sequence generation for condition order is not described. Insufficient information about the sequence generation process to permit judgement of 'low risk' or 'high risk'
Allocation concealment (selection bias)	Low risk	Comment: participating small groups of children appear to have been randomised to condition order concurrently, after consent for individuals' participation had been obtained. The review authors therefore judge that any lack of concealment of allocation sequence is unlikely to be an issue for risk of bias.
Blinding of participants and personnel (performance bias) Consumption outcome	Unclear risk	Quote: "Test visits were spaced 1 week apart to minimize carryover effects...To minimize visual comparisons of portion sizes, all children in the same group were served the same experimental condition." Comment: no blinding or incomplete blinding. Not reported whether participants were probed for suspicion of study purpose or awareness of size manipulation between study conditions. It is possible that the outcome may be influenced by lack of blinding of study participants (due to potential carry‐over effects between conditions). Very unlikely that key study personnel were blinded, but the review authors judge that the outcome is not likely to be influenced by lack of blinding of key study personnel
Blinding of outcome assessment (detection bias) Consumption outcome	Low risk	Comment: no blinding of outcome assessment, but the review authors judge that the outcome measurement is not likely to be influenced by lack of blinding
Incomplete outcome data (attrition bias) Consumption outcome	High risk	Quote: "Three children were excluded at the beginning of the study due to disliking the main entrée. To examine the role of liking in F&V portion size effects, children had to like either the fruit or vegetable used in the experiment, but not necessarily both. One child disliked both the F&V and was excluded from the study. Four children ate negligible amounts of both foods (<10 g fruit and <10 g vegetable) at more than half of the visits and were, therefore, excluded from the analysis." Comment: the first reason for missing outcome data for consumption outcome is the study authors' decision to exclude participants who disliked both the fruit and the vegetable side dish from the analysis. This reason for exclusion is likely to be related to consumption outcome but inclusion could plausibly have biased the estimate of the effect of the intervention on consumption. The review authors judge that the decision to exclude participants for this reason is reasonable, as it is likely to protect against bias in the estimate of the effect of the intervention on consumption. The second reason for missing outcome data for consumption outcome is the study authors' decision to exclude participants those with consumption < 10 g fruit and < 10 g vegetables at more than half of the visits from the analysis. The substantial proportion (4 participants, 11% of study sample) of exclusions due to low consumption means that the review authors judge that it is plausible that the effect size among these missing data is enough to have had an important impact on the observed effect size
Selective reporting (reporting bias)	Unclear risk	Comment: search for record(s) containing details of study protocol conducted in ClinicalTrials.gov and the WHO International Clinical Trials Registry Platform (ICTRP). No records found. Insufficient information to permit judgement of 'low risk' or 'high risk'
Other bias #1 ‐ Baseline comparability of participant characteristics between groups	Unclear risk	Comment: study uses a within‐subjects design. No measurement of participant pre‐condition 'state' characteristics is reported. No analysis of potential differences in measured outcomes between condition orders appears to have been conducted and the statistical analysis of outcome data does not appear to control for condition order. Risk of bias due to period effects is therefore unclear. Insufficient information to permit judgement of 'low risk' or 'high risk'
Other bias #2 ‐ Consistency in intervention delivery	Unclear risk	Quote: "Parents were asked to refrain from giving any food or beverages to their child 2 hours before arrival and to report any deviations from these instructions. A trained staff member sat at the table during the meal to ensure that procedures were followed, including preventing children from sharing foods, noting dropped foods, and redirecting food‐related conversation... Children were instructed to eat as little or as much as they liked." Comment: parents' compliance with the instruction to refrain from giving any food or beverages to their child 2 hours before arrival at each study dinner meal was monitored by parent self report; however, no monitoring results are reported with respect to this instruction. Children's compliance with an instruction not to share foods was monitored and enforced by a trained staff member. No further specific instructions were provided to participants, other than the instruction to children to eat as little or as much as they liked
Summary of risk of bias Consumption outcome	Unclear risk	Unclear risk

**Table:** Mishra 2012 (S1)

Methods	Study design: between‐subjects cluster‐randomised controlled trial Unit of allocation: restaurant table Unit of analysis: individual Number of clusters: not reported Number of participants per cluster: not reported Analysis does not appear to account for cluster allocation, as the statistical model does not appear to include any covariate related to cluster assignment	
Participants	Setting: field setting, Italian restaurant Geographical region: south‐western United States Number of enrolled participants: 99 adults Number (%) of enrolled participants completing the study: 99 (100%) Study completers ‐ mean age (SD): not reported Study completers ‐ sex: not reported Study completers ‐ mean BMI kg/m^2^ (SD): not reported (neither BMI nor other body weight or body weight status) Specific social or cultural characteristics: none Socio‐economic status context: low deprivation Inclusion criteria: none reported Exclusion criteria: none reported	
Interventions	Manipulated product type: food Manipulation: tableware size Duration of exposure to intervention: ≤ 1 day Social setting: consuming with others Study arms: small fork (fork volume 20% less than the regular (standard) restaurant fork); large fork (fork volume 20% more than the regular (standard) restaurant fork) Number of comparisons analysed: 1 Comparisons analysed: Intervention 1: small fork (fork volume 20% less than the regular (standard) restaurant fork);*versus* Intervention 2: large fork (fork volume 20% more than the regular (standard) restaurant fork) Concurrent intervention components: no	
Outcomes	Outcomes reported in study: amount of food left on the plate after meal (ounces) Selection outcome analysed: N/A Measurement of selection outcome: N/A Timing of selection outcome measurement: N/A Consumption outcome analysed: amount of food left on the plate after meal (ounces) Measurement of consumption outcome: objective Timing of consumption outcome measurement: immediate (≤ 1 day)	
Funding source	No funding to disclose; research support provided by the David Eccles School of Business	
Notes	Attempts to contact author to request information missing from the study report but no contact could be established	
***Risk of bias***		
**Bias**	**Authors' judgement**	**Support for judgement**
Random sequence generation (selection bias)	High risk	Quote: "For each meal, tables were assigned to be either "large fork" or "small fork" tables, and the fork assignments were rotated after every meal."
Allocation concealment (selection bias)	High risk	Quote: "For each meal, tables were assigned to be either "large fork" or "small fork" tables, and the fork assignments were rotated after every meal." Comment: explicitly unconcealed procedure and investigators enrolling participants could possibly foresee assignments and thus introduce risk of selection bias
Blinding of participants and personnel (performance bias) Consumption outcome	Unclear risk	Comment: no blinding or incomplete blinding of study participants (as study setting was a restaurant, but unclear whether 'small fork' and 'large fork' tables were adjacent to one another) and it is possible that the outcome may be influenced by lack of blinding (due to potential carry‐over effects between conditions). Very unlikely that key study personnel were blinded, but the review authors judge that the outcome is not likely to be influenced by lack of blinding of key study personnel
Blinding of outcome assessment (detection bias) Consumption outcome	Low risk	Comment: no blinding of outcome assessment, but the review authors judge that the outcome measurement is not likely to be influenced by lack of blinding
Incomplete outcome data (attrition bias) Consumption outcome	Unclear risk	Comment: attrition is not described. Insufficient information to permit judgement of 'low risk' or 'high risk'
Selective reporting (reporting bias)	Unclear risk	Comment: search for record(s) containing details of study protocol conducted in ClinicalTrials.gov and the WHO International Clinical Trials Registry Platform (ICTRP). No records found. Insufficient information to permit judgement of 'low risk' or 'high risk'
Other bias #1 ‐ Baseline comparability of participant characteristics between groups	Unclear risk	Comment: study uses a between‐subjects design. Insufficient information to permit judgement of 'low risk' or 'high risk'
Other bias #2 ‐ Consistency in intervention delivery	Low risk	Comment: no information or instructions appear to have been provided to participants; therefore no concerns about related risk of bias
Summary of risk of bias Consumption outcome	High risk	High risk

**Table:** Mishra 2012 (S2)

Methods	Study design: between‐subjects randomised controlled trial	
Participants	Setting: laboratory setting Geographical region: not reported Number of enrolled participants: 81 adults Number (%) of enrolled participants completing the study: 81 (100%) Study completers ‐ mean age (SD): not reported Study completers ‐ sex: not reported Study completers ‐ mean BMI kg/m^2^ (SD): not reported (neither BMI nor other body weight or body weight status) Specific social or cultural characteristics: none Socio‐economic status context: low deprivation Inclusion criteria: none reported Exclusion criteria: none reported	
Interventions	Manipulated product type: food Manipulation: tableware size Duration of exposure to intervention: ≤ 1 day Social setting: consuming alone Study arms: small fork (fork volume 20% less than the regular (standard) restaurant fork); large fork (fork volume 20% more than the regular (standard) restaurant fork) Number of comparisons analysed: 1 Comparisons analysed: Intervention 1: small fork (fork volume 20% less than the regular (standard) restaurant fork);*versus* Intervention 2: large fork (fork volume 20% more than the regular (standard) restaurant fork) Concurrent intervention components: no	
Outcomes	Outcomes reported in study: amount of food left on the plate after meal (ounces) Selection outcome analysed: N/A Measurement of selection outcome: N/A Timing of selection outcome measurement: N/A Consumption outcome analysed: amount of food left on the plate after meal (ounces) Measurement of consumption outcome: objective Timing of consumption outcome measurement: immediate (≤ 1 day)	
Funding source	No funding to disclose; research support provided by the David Eccles School of Business	
Notes	Attempts to contact author to request information missing from the study report but no contact could be established	
***Risk of bias***		
**Bias**	**Authors' judgement**	**Support for judgement**
Random sequence generation (selection bias)	Unclear risk	Comment: method of sequence generation is not described. Insufficient information about the sequence generation process to permit judgement of 'low risk' or 'high risk'
Allocation concealment (selection bias)	Unclear risk	Comment: method of concealment is not described. Insufficient information to permit judgement of 'low risk' or 'high risk'
Blinding of participants and personnel (performance bias) Consumption outcome	Unclear risk	Comment: insufficient information to permit judgement of 'low risk' or 'high risk'
Blinding of outcome assessment (detection bias) Consumption outcome	Low risk	Comment: no blinding of outcome assessment, but the review authors judge that the outcome measurement is not likely to be influenced by lack of blinding
Incomplete outcome data (attrition bias) Consumption outcome	Unclear risk	Comment: attrition is not described. Insufficient information to permit judgement of 'low risk' or 'high risk'
Selective reporting (reporting bias)	Unclear risk	Comment: search for record(s) containing details of study protocol conducted in ClinicalTrials.gov and the WHO International Clinical Trials Registry Platform (ICTRP). No records found. Insufficient information to permit judgement of 'low risk' or 'high risk'
Other bias #1 ‐ Baseline comparability of participant characteristics between groups	Unclear risk	Comment: study uses a between‐subjects design. Insufficient information to permit judgement of 'low risk' or 'high risk'
Other bias #2 ‐ Consistency in intervention delivery	Unclear risk	Insufficient information to permit judgement of 'low risk' or 'high risk'
Summary of risk of bias Consumption outcome	Unclear risk	Unclear risk

**Table:** Raynor 2007

Methods	Study design: between‐subjects randomised controlled trial	
Participants	Setting: field setting; universities around Rhode Island, USA Geographical region: Rhode Island, USA Number of enrolled participants: 40 adults Number (%) of enrolled participants completing the study: 28 (70) Study completers – mean age (SD): 20 (1.6) Study completers ‐ sex: male (25%) and female (75%) Study completers ‐ mean BMI kg/m^2^ (SD): 23.45 (3.38) Specific social or cultural characteristics: university community Socio‐economic status context: low deprivation Inclusion criteria: healthy; do not have a health condition or use medication that affects eating or requires specialised diet therapy (e.g. diabetes); non‐smoker; not obese (self reported BMI < 30 kg/m^2^); aged between 18 and 30 years; unrestrained eater; not a binge eater; not following a weight loss diet; not an athlete in training; not pregnant or breastfeeding; consume snack foods 3 times per week; do not have allergies; do not have unfavourable preferences toward snack foods used in the study Exclusion criteria: not stated	
Interventions	Manipulated product type: food Manipulation: portion size (comparison 1); package size (comparison 2) Duration of exposure to intervention: > 1 day Social setting: selecting/consuming both alone and with others Study arms: small portion (portion being overall amount available)‐small package (5 1‐oz bags potato chips, 5 1.5‐oz bags crackers, 6 1.25‐oz bags cookies, 5 1.7‐oz bags candies); small portion‐large package (1 5‐oz bag potato chips, 1 7.2‐oz bag crackers, 1 8‐oz bag cookies, 1 9.4‐oz bag candies); large portion‐small package (10 1‐oz bags potato chips, 9 1.5‐oz bags crackers, 12 1.25‐oz cookies, 11 1.7‐oz bags candies); large portion‐large package (2 5‐oz bags potato chips, 2 7.2‐oz bags crackers, 2 8‐oz bags cookies, 2 9.4‐oz bags candies) Number of comparisons analysed: 2 (portion size; package size) Comparisons analysed: comparison 1 (portion) = Intervention 1: small portion of 4 snack foods (5 1‐oz bags potato chips, 5 1.5‐oz bags crackers, 6 1.25‐oz bags cookies, 5 1.7‐oz bags candies OR 1 5‐oz bag potato chips, 1 7.2‐oz bag crackers, 1 8‐oz bag cookies, 1 9.4‐oz bag candies); *versus* Intervention 2: large portion of 4 snack foods (10 1‐oz bags potato chips, 9 1.5‐oz bags crackers, 12 1.25‐oz cookies, 11 1.7‐oz bags candies OR 2 5‐oz bags potato chips, 2 7.2‐oz bags crackers, 2 8‐oz bags cookies, 2 9.4‐oz bags candies) Comparison 2 (Package) = Intervention 1: small package of 4 snack foods (5 1‐oz bags potato chips, 5 1.5‐oz bags crackers, 6 1.25‐oz bags cookies, 5 1.7‐oz bags candies OR 10 1‐oz bags potato chips, 9 1.5‐oz bags crackers, 12 1.25‐oz cookies, 11 1.7‐oz bags candies); *versus* Intervention 2: large package of 4 snack foods (1 5‐oz bag potato chips, 1 7.2‐oz bag crackers, 1 8‐oz bag cookies, 1 9.4‐oz bag candies OR 2 5‐oz bags potato chips, 2 7.2‐oz bags crackers, 2 8‐oz bags cookies, 2 9.4‐oz bags candies) Concurrent intervention components: no	
Outcomes	Outcomes reported in study: total grams intake from snacks over 3 days (grams); total energy intake from snacks over 3 days (kilojoules) Selection outcome analysed: N/A Measurement of selection outcome: N/A Timing of selection outcome measurement: N/A Consumption outcome analysed: total energy intake from snacks over 3 days (kilojoules) Measurement of consumption outcome: objective Timing of consumption outcome measurement: longer‐term (> 1 day)	
Funding source	National Institute of Diabetes and Digestive and Kidney Diseases	
Notes	Manipulated both portion and package size. Comparisons were analysed for both portion size and package size	
***Risk of bias***		
**Bias**	**Authors' judgement**	**Support for judgement**
Random sequence generation (selection bias)	Unclear risk	Comment: method of sequence generation is not described. Insufficient information about the sequence generation process to permit judgement of 'low risk' or 'high risk'
Allocation concealment (selection bias)	Unclear risk	Comment: method of concealment is not described. Insufficient information to permit judgement of 'low risk' or 'high risk'
Blinding of participants and personnel (performance bias) Consumption outcome	Low risk	Quote: "A between‐subjects design was used because requiring participants to go through several different groups in the study might produce satiation to the foods used in the study, causing intake to decrease with each successive group that a participant completed. Also, food given to the participants looked very different in each group; thus, the manipulation of the study would be very apparent to participants participating in more than one group... Participants were men and women between the ages of 18 and 30 years recruited by flyers posted around local universities (Providence, RI) regarding a study investigating the effects of snack food consumption on liking of snack foods." Comment: blinding of study participants attempted and unlikely that the blinding could have been broken. Very unlikely that key study personnel were blinded, but the review authors judge that the outcome is not likely to be influenced by lack of blinding of key study personnel
Blinding of outcome assessment (detection bias) Consumption outcome	Low risk	Comment: no blinding of outcome assessment, but the review authors judge that the outcome measurement is not likely to be influenced by lack of blinding
Incomplete outcome data (attrition bias) Consumption outcome	Low risk	Quote: "Forty participants enrolled in the investigation, but 12 were excluded from the study [6 participants did not show for the second session, 4 participants rated the foods used in the study <50 on a 100‐mm visual analog scale (VAS) during the first session, and 2 participants measured BMI was ≥30]. Therefore, 28 participants, 12 men and 16 women, completed the investigation." Comment: reasons for exclusion from analysis are per protocol and therefore do not raise concerns about risk of attrition bias due to handing of exclusions
Selective reporting (reporting bias)	High risk	Comment: search for record(s) containing details of study protocol conducted in ClinicalTrials.gov and the WHO International Clinical Trials Registry Platform (ICTRP). Record found in ClinicalTrials.gov (Identifier: NCT00200213). Comparison of ClinicalTrials.gov record with published study report indicates selective outcome reporting. The ClinicalTrials.gov record states that the study dependent variables [outcomes] would be the amount of grams and kcal consumed from the provided junk [snack] foods over 3 days, while the published study report only reports results for kcal (and KJs) consumed from the provided [junk] snack foods over 3 days. A comparison between the Methods and Results sections of the published study report confirms this assessment. The review authors judge that this discrepancy elevates risk of bias due to selective outcome reporting, since it is possible that the study could have detected a significant main effect of portion size on the amount of kcal consumed but no significant main effect of portion size on the amount of grams consumed (or vice versa)
Other bias #1 ‐ Baseline comparability of participant characteristics between groups	Low risk	Quote: "Participant weight was assessed by use of an electric scale, and height was assessed using a stadiometer, using standard procedures...BMI was calculated as weight in kg/height in m2. VASs were used to assess hedonics of the foods. Participants rated each of the snack foods, with a 100‐mm scale, using anchors of "very unpleasant" and "very pleasant"... Baseline characteristics of the participants are presented in Table 2. There were no differences in age; restraint; hedonic ratings of the potato chips, crackers, or cookies; hours since last meal before the first session; or race/ethnicity between the four groups. For BMI, there was a significant interaction,...with the small unit/large amount group having a significantly...lower BMI...than the small unit/small amount group...and the large unit/large amount group...BMI [was] also significantly related to the primary dependent variable and [was] included as [a covariate] in the analyses of snack food intake." Comment: study uses a between‐subjects design. Differences between comparison groups in terms of BMI. The statistical analysis of outcome data controls for this difference. No differences between comparison groups in terms of other measured baseline participant characteristics
Other bias #2 ‐ Consistency in intervention delivery	Low risk	Quote: "Participants were given a box of the previously tested snack foods corresponding to their randomly assigned group and instructed to eat as much or as little as they wanted of these foods over the next 3 days. Participants were informed that during the 3‐day period they needed to at least taste each of the 4 snack foods and to not eat other snack foods. They were also instructed to not let anyone else in their household/dormitory eat any of the provided snack foods... At the second appointment, participants... wrote down everything they had eaten and drunk in the time period since the first session. This was to determine the number of snack foods consumed over the 3 days in which snack foods had been provided. Participants were asked if anyone other than themselves had consumed the provided snack foods over the 3 days, and all participants self‐reported that no one else had consumed any of the provided snack foods... Over the 3‐day period participants consumed 4.5 +/‐ 1.2 different types of snack foods (6 of the 28 participants consumed more than the four provided snack foods), with no difference in number of snack foods consumed occurring between the groups... and with all participants reporting eating the four provided snack foods." Comment: information and instructions provided to participants appear to have been standardised between the compared study conditions. Participants' compliance with the instruction not eat snack foods other than those provided was monitored by written self report. Although 6 of 28 participants failed to comply with the latter instruction, there was no difference between the compared study conditions in the number of different types of snack foods consumed during the 3‐day study period. Participants' compliance with the instruction that they needed to at least taste each of the 4 provided snack foods was monitored by written self report. All participants reported eating the 4 provided snack foods during the 3‐day study period. Participants' compliance with the instruction to not let anyone else in their household/dormitory eat any of the provided snack foods was monitored by self report. All participants reported that no one else had consumed any of the provided snack foods. No further specific instructions were provided to participants, other than the instruction to eat as much or as little as they wanted of the provided snack foods over the next 3 days
Summary of risk of bias Consumption outcome	Unclear risk	Unclear risk

**Table:** Raynor 2009

Methods	Study design: between‐subjects randomised controlled trial	
Participants	Setting: field setting Geographical region: not reported Number of enrolled participants: 24 adults Number (%) of enrolled participants completing the study: 19 (79.2) Study completers – mean age (SD): 50.6 (9.3) Study completers ‐ sex: 94.7% female Study completers ‐ mean BMI kg/m^2^ (SD): 31.8 (4) Specific social or cultural characteristics: participants were recruited during July 2005 through local newspaper advertisements and from a database of individuals interested in participating in weight‐loss interventions Socio‐economic status context: low deprivation Inclusion criteria: eligibility criteria for the study were age 21 to 65 years; body mass index (BMI; calculated as kg/m2) 25 to 40, and consumption of breakfast 4 days/week Exclusion criteria: participants were phone‐screened and excluded if they were lactose‐intolerant; allergic to or would not eat the provided foods; could not engage in physical activity; were participating in a weight‐loss programme and/or taking weight‐loss medication or lost 5% of body weight during the past 6 months; unavailable for meetings 1 week during the programme; or were either pregnant, lactating or 6 months postpartum, or planned to become pregnant during the investigation	
Interventions	Manipulated product type: food Manipulation: package size Duration of exposure to intervention: > 1 day Social setting: selecting/consuming both alone and with others Study arms: small package size (cereal: 22 0.68‐oz boxes, peaches: 12 4‐oz cans, applesauce: 12 4‐oz cans, cheese: 16 1‐oz blocks): large package size (cereal: 1 15‐oz box, peaches: 3 15‐oz cans, applesauce: 3 15‐oz cans, cheese: 2 10‐oz blocks) Number of comparisons analysed: 1 Comparisons analysed: Intervention 1: small package size (cereal: 22 0.68‐oz boxes, peaches: 12 4‐oz cans, applesauce: 12 4‐oz cans, cheese: 16 1‐oz blocks); *versus* Intervention 2: large package size (cereal: 1 15‐oz box, peaches: 3 15‐oz cans, applesauce: 3 15‐oz cans, cheese: 2 10‐oz blocks) Concurrent intervention components: yes. Behavioural intervention identical in both conditions. Separate 60‐minute weekly group sessions for each condition, led by interventionists with expertise in weight management and delivered with the aid of a treatment manual. Participants were instructed to consume a standard calorie‐ and fat‐restricted diet and were shown how to correctly measure and weigh all food consumed. Participants were instructed to gradually increase their physical activity by 5 minutes per day each week until they reached the intervention goal of 30 minutes of activity 5 days per week. Behavioural and cognitive skills intended to help implement changes in eating and activity behaviours were taught to participants at each session. Participants were encouraged to eat breakfast daily and keep track of the number of days each week the provided foods were consumed at breakfast in a daily food diary	
Outcomes	Outcomes reported in study: mean energy intake per day of the provided foods over the course of the intervention, also assessed by each of the four foods Selection outcome analysed: N/A Measurement of selection outcome: N/A Timing of selection outcome measurement: N/A Consumption outcome analysed: mean energy intake per day from all provided foods (kcal) Measurement of consumption outcome: objective Timing of consumption outcome measurement: longer‐term (> 1 day)	
Funding source	National Institutes of Health	
Notes	—	
***Risk of bias***		
**Bias**	**Authors' judgement**	**Support for judgement**
Random sequence generation (selection bias)	Low risk	Quote: "Participants were then randomized using a random number table into one of the two treatment groups (Single‐Serving or Standard)."
Allocation concealment (selection bias)	High risk	Quote: "Participants were then randomized using a random number table into one of the two treatment groups (Single‐Serving or Standard)." Comment: unconcealed procedure and investigators enrolling participants could possibly foresee assignments
Blinding of participants and personnel (performance bias) Consumption outcome	Low risk	Quote: "Interventionists were not blinded to study condition as they distributed food weekly to participants." Comment: blinding of study participants attempted and unlikely that the blinding could have been broken. Very unlikely that key study personnel were blinded, but the review authors judge that the outcome is not likely to be influenced by lack of blinding of key study personnel
Blinding of outcome assessment (detection bias) Consumption outcome	Low risk	Comment: no blinding of outcome assessment, but the review authors judge that the outcome measurement is not likely to be influenced by lack of blinding
Incomplete outcome data (attrition bias) Consumption outcome	Low risk	Quote: "Twenty‐four of the 35 eligible individuals attended an orientation session where informed consent/signed Health Insurance Portability and Accountability Act forms were obtained. These 24 individuals were randomized into a condition, but five participants developed scheduling conflicts and could not be given foods to consume each week. There were no significant...differences in age, BMI, sex, race, education, and marital status in the completers and noncompleters, but the noncompleters had a greater percentage of Hispanic individuals than the completers...Complete consumption data from provided foods was obtained from 19 participants." Comment: reason for missing outcome data is unlikely to be related to consumption outcome
Selective reporting (reporting bias)	Low risk	Comment: search for record(s) containing details of study protocol conducted in ClinicalTrials.gov and the WHO International Clinical Trials Registry Platform (ICTRP). Record found in ClinicalTrials.gov (Identifier: NCT00200239). Comparison of ClinicalTrials.gov/ICTRP records with published study report indicates no selective outcome reporting
Other bias #1 ‐ Baseline comparability of participant characteristics between groups	Low risk	Quote: "At the baseline assessment session, height was measured by a stadiometer...and weight was measured on a physician's digital scale...using standard procedures..., allowing for calculation of BMI. At the baseline assessment session a demographic questionnaire was also completed by participants...There were no differences in participant baseline characteristics between Single‐Serving and Standard...(Table)." Comment: study uses a between‐subjects design. No differences between comparison groups in terms of measured baseline participant characteristics
Other bias #2 ‐ Consistency in intervention delivery	Unclear risk	Quote: "A breakfast prescription, identical for both conditions, was given to all participants. This prescription was to eat a serving of each of the provided foods for breakfast daily, along with one serving of low‐fat or non‐fat milk with the cereal and one serving of bread with the cheese, providing an approximately 200‐ to 300‐kcal/breakfast within 2 hours of awakening. Participants were instructed not to consume the provided foods at other times of the day... Participants were instructed to gradually increase their physical activity by 5 minutes per day each week until they reached the intervention goal of 30 minutes of activity 5 days per week... Participants were encouraged to eat breakfast daily and keep track of the number of days each week the provided foods were consumed at breakfast in a daily food diary... Number of days per week in which breakfast was consumed during treatment was not significantly different between the conditions (6.7 +/‐ 0.4 day/week; P>0.10)." Comment: participants' compliance with the instruction to eat a serving of each of the provided foods for breakfast daily, along with one serving of low‐fat or non‐fat milk with the cereal and one serving of bread with the cheese, was monitored by self report using a daily food diary; however, no monitoring results specific to this instruction are reported. Participants' compliance with the instruction to eat breakfast daily was monitored by self report using a daily food diary. There was no difference between study conditions in the number of days on which breakfast was consumed during the study period. No information pertaining to monitoring of participants' compliance with the instructions to not to consume the provided foods at other times of the day or to gradually increase their physical activity by 5 minutes per day each week until they reached the intervention goal of 30 minutes of activity 5 days per week is reported. No further specific instructions were provided to participants
Summary of risk of bias Consumption outcome	High risk	High risk

**Table:** Rolls 2000

Methods	Study design: within‐subjects randomised controlled trial	
Participants	Setting: laboratory setting Geographical region: Pennsylvania, USA Number of enrolled participants: 16 3‐year‐old children; 16 5‐year‐old children Number (%) of enrolled participants completing the study: 3‐year‐old children = 16 (100%); 5‐year‐old children = 16 (100%) Study completers ‐ mean age (SD): 3‐year‐old children = 3.6 (not reported); 5‐year‐old children = 5 (not reported) Study completers ‐ sex: 3‐year‐old children = 50% female; 5‐year‐old children = 62.5% female Study completers ‐ mean BMI kg/m^2^ (SD): 3‐year‐old children = not reported; 5‐year‐old children = not reported. BMI percentile reported Specific social or cultural characteristics: preschool children enrolled in a daycare programme at the Pennsylvania State University Child Development Laboratory Socio‐economic status context: low deprivation Inclusion criteria: not stated Exclusion criteria: not stated	
Interventions	Manipulated product type: food Manipulation: portion size Duration of exposure to intervention: ≤ 1 day Social setting: selecting/consuming with others Study arms: small portion of macaroni cheese (for 3‐year‐olds = 150 g, for 5‐year‐olds = 225 g); medium portion of macaroni cheese (for 3‐year‐olds = 263 g, for 5‐year‐olds = 338 g); large portion of macaroni cheese (for 3‐year‐olds = 376 g, for 5‐year‐olds = 450 g) Number of comparisons analysed: 4 (3‐year‐olds = 2; 5‐year‐olds = 2) Comparisons analysed: 3‐year‐olds: Comparison 1 = Intervention 1: small portion size: 150 g macaroni cheese; *versus* Intervention 2: medium portion size: 263 g macaroni cheese Comparison 2 = Intervention 1: medium portion size: 263 g macaroni cheese; *versus* Intervention 2: large portion size: 376 g macaroni cheese 5‐year‐olds: Comparison 1 = Intervention 1: small portion size: 225 g macaroni cheese; *versus* Intervention 2: medium portion size: 338g macaroni cheese Comparison 2 = Intervention 1: medium portion size: 338 g macaroni cheese; *versus* Intervention 2: large portion size: 450 g macaroni cheese Concurrent intervention components: no	
Outcomes	Outcomes reported in study: total energy intake (kcal) (consumption); weight intake of manipulated macaroni and cheese Selection outcome analysed: N/A Measurement of selection outcome: N/A Timing of selection outcome measurement: N/A Consumption outcome analysed: energy intake from total lunch meal (kcal) Measurement of consumption outcome: objective. Timing of consumption outcome measurement: immediate (≤ 1 day)	
Funding source	United States National Institutes of Health. Food provided by Nestlé	
Notes	Outcome data for 3‐year‐old and 5‐year‐old children analysed separately (2 comparisons each) because the absolute difference in portion size between portion size conditions varied between age groups. Study authors contacted for missing data with additional data received March 2014	
***Risk of bias***		
**Bias**	**Authors' judgement**	**Support for judgement**
Random sequence generation (selection bias)	Unclear risk	Comment: method of sequence generation for condition order is not described. Author contact confirmed condition order was randomised but no further details (13/3/13). Insufficient information about the sequence generation process to permit judgement of 'low risk' or 'high risk'
Allocation concealment (selection bias)	Unclear risk	Comment: method of concealment is not described. Author contact confirmed condition order was randomised but no further details (13/3/13). Insufficient information to permit judgement of 'low risk' or 'high risk'
Blinding of participants and personnel (performance bias) Consumption outcome	Unclear risk	Comment: no blinding of study participants reported. Not reported whether participants were probed for suspicion of study purpose or awareness of size manipulation between study conditions. It is possible that the outcome may be influenced by lack of blinding of study participants (due to potential carry‐over effects between conditions). Very unlikely that key study personnel were blinded, but the review authors judge that the outcome is not likely to be influenced by lack of blinding of key study personnel
Blinding of outcome assessment (detection bias) Consumption outcome	Low risk	Comment: no blinding of outcome assessment, but the review authors judge that the outcome measurement is not likely to be influenced by lack of blinding
Incomplete outcome data (attrition bias) Consumption outcome	Low risk	Comment: no missing outcome data for consumption outcome
Selective reporting (reporting bias)	Unclear risk	Comment: search for record(s) containing details of study protocol conducted in ClinicalTrials.gov and the WHO International Clinical Trials Registry Platform (ICTRP). No records found. Insufficient information to permit judgement of 'low risk' or 'high risk'
Other bias #1 ‐ Baseline comparability of participant characteristics between groups	Unclear risk	Quote: "Before...each lunch, children's hunger was assessed using cartoon drawings of children with stomachs shaded to represent degree of fullness...Children's liking of the macaroni and cheese was also assessed using cartoons with different facial expressions...Hunger ratings before the meal did not differ by...condition." Comment: study uses a within‐subjects design. Differences between conditions in terms of measured pre‐condition participant 'state' characteristics are partially reported, but not reported whether there were differences between condition orders in terms of measured pre‐condition participant 'state' characteristics. No analysis of potential differences in measured outcomes between condition orders appears to have been conducted and the statistical analysis of outcome data does not appear to control for condition order. Risk of bias due to period effects is therefore unclear. Insufficient information to permit judgement of 'low risk' or 'high risk'
Other bias #2 ‐ Consistency in intervention delivery	Low risk	Comment: information provided to participants appears to have been standardised between the compared study conditions. No specific instructions were provided to participants and therefore participants' compliance with instructions is not applicable
Summary of risk of bias Consumption outcome	Unclear risk	Unclear risk

**Table:** Rolls 2002

Methods	Study design: within‐subjects randomised controlled trial	
Participants	Setting: laboratory setting Geographical region: Pennsylvania, USA Number of enrolled participants: 51 adults Number (%) of enrolled participants completing the study: 51 (100%) Study completers ‐ mean age (SD): 22.2 (2.5) Study completers ‐ sex: 49% female Study completers ‐ mean BMI kg/m^2^ (SD): 23.7 (2) Specific social or cultural characteristics: not stated Socio‐economic status context: low deprivation Inclusion criteria: aged 21 to 40 y, were in good health, were not currently following a weight‐loss diet or trying to gain weight, were not using medication known to affect food intake or appetite, were not athletes in training, were not pregnant or lactating, had no food allergies or food restrictions that would affect food intake, and regularly ate 3 meals/d; body mass index (BMI; in kg/m2) was 20 to 28 Exclusion criteria: scored ≥ 30 on the EAT–40 or ≥ 40 on the Zung Questionnaire or if they reported that they disliked any of the foods to be served at the test meal	
Interventions	Manipulated product type: food Manipulation: portion size Duration of exposure to intervention: ≤ 1 day Social setting: consuming alone Study arms: 500 g macaroni cheese ‐ received on plate; 500 g macaroni cheese ‐ received in dish to self serve; 625 g macaroni cheese ‐ received on plate; 625 g macaroni cheese ‐ received in dish to self serve; 750 g macaroni cheese ‐ received on plate; 750 g macaroni cheese ‐ received in dish to self serve; 1000 g macaroni cheese ‐ received on plate; 1000 g macaroni cheese ‐ received in dish to self serve Number of comparisons analysed: 3 Comparisons analysed: Comparison 1 = Intervention 1: 500 g macaroni cheese; *versus* Intervention 2: 625 g macaroni cheese; Comparison 2 = Intervention 1: 625 g macaroni cheese; *versus* Intervention 2: 750 g macaroni cheese; Comparison 3 = Intervention 1: 750 g macaroni cheese; versus Intervention 2: 1000 g macaroni cheese Concurrent intervention components: yes. Served portion on a plate or self served from a dish	
Outcomes	Outcomes reported in study: total energy intake from meal (kJ); weight intake of manipulated macaroni and cheese Selection outcome analysed: N/A Measurement of selection outcome: N/A Timing of selection outcome measurement: N/A Consumption outcome analysed: energy intake from total lunch meal (kJ) Measurement of consumption outcome: objective Timing of consumption outcome measurement: immediate (≤ 1 day)	
Funding source	United States National Institutes of Health	
Notes	Study authors contacted for missing data with additional data received March 2014	
***Risk of bias***		
**Bias**	**Authors' judgement**	**Support for judgement**
Random sequence generation (selection bias)	Unclear risk	Comment: method of sequence generation for condition order is not described. Author contact confirmed condition order was randomised but no further details (13/3/13). Insufficient information about the sequence generation process to permit judgement of 'low risk' or 'high risk'
Allocation concealment (selection bias)	Unclear risk	Comment: method of concealment is not described. Author contact confirmed condition order was randomised but no further details (13 March 2013). Insufficient information to permit judgement of 'low risk' or 'high risk'
Blinding of participants and personnel (performance bias) Consumption outcome	Unclear risk	Quote: "The subjects were not informed of the actual purpose of the study but were told that the purpose was to examine the effects of lunch on taste... Subjects completed a discharge questionnaire at the end of the study, which asked what they thought was the purpose of the study, whether there were any factors that affected their responses, and whether they noticed any differences between the test days... Most subjects (94%) did not correctly report the purpose of the study. Three subjects (2 from the plate group and 1 from the serving dish group), however, correctly reported that the purpose of the study was to investigate whether the amount of food that was offered affected the amount that they ate. Less than one‐half (45%) of the subjects reported that they noticed differences in the portion sizes of the macaroni and cheese that were presented to them." Comment: no blinding or incomplete blinding. Participants were probed for suspicion of study purpose and awareness of size manipulation between study conditions. Blinding of study participants was broken in at least some cases and it is possible that the outcome may be influenced by lack of blinding (due to potential carry‐over effects between conditions). Very unlikely that key study personnel were blinded, but the review authors judge that the outcome is not likely to be influenced by lack of blinding of key study personnel
Blinding of outcome assessment (detection bias) Consumption outcome	Low risk	Comment: no blinding of outcome assessment, but the review authors judge that the outcome measurement is not likely to be influenced by lack of blinding
Incomplete outcome data (attrition bias) Consumption outcome	Low risk	Comment: no missing outcome data for consumption outcome
Selective reporting (reporting bias)	Unclear risk	Comment: search for record(s) containing details of study protocol conducted in ClinicalTrials.gov and the WHO International Clinical Trials Registry Platform (ICTRP). No records found. Insufficient information to permit judgement of 'low risk' or 'high risk'
Other bias #1 ‐ Baseline comparability of participant characteristics between groups	Unclear risk	Quote: "Subjects completed ratings of hunger and satiety immediately before...lunch. Subjects rated their hunger, thirst, prospective consumption (how much food they thought they could eat), nausea, and fullness on visual analogue scales (VASs)... Immediately before...lunch, subjects were also presented with 10‐g samples of macaroni and cheese, which were rated for palatability (pleasantness of appearance, odor, taste, and texture) with the use of VASs...Across all conditions of portion size, no significant differences were found before lunch in ratings of hunger, prospective consumption, fullness, thirst, or nausea in either group (data not shown)... Across all conditions of portion size, no significant differences were found before lunch in ratings of appearance, odor, taste, or texture of the sample of macaroni and cheese in either group (data not shown)." Comment: no differences between conditions in terms of measured pre‐condition participant 'state' characteristics, but not reported whether there were differences between condition orders in terms of measured pre‐condition participant 'state' characteristics. No analysis of potential differences in measured outcomes between condition orders appears to have been conducted and the statistical analysis of outcome data does not appear to control for condition order. Risk of bias due to period effects is therefore unclear. Insufficient information to permit judgement of 'low risk' or 'high risk'
Other bias #2 ‐ Consistency in intervention delivery	Low risk	Quote: "Subjects were asked to keep their evening meal and their activity level as similar as possible on the day before each test day and to refrain from eating or drinking (except water) after 2200. Subjects were also asked to refrain from drinking alcohol on the day before and throughout each test day and to eat a similar breakfast on the morning of each test day. During each test day, subjects were instructed not to consume any food or energy‐containing beverages for 3 h before the test meal and not to drink water for 1 h before the test meal. On completion of each test meal, subjects were instructed not to consume any food or energy‐containing beverages for the next 3 h and to eat a similar dinner on the evening of each test day. Subjects kept a brief record of their food intake and activity patterns on the day before and the day of each test meal; the purpose of the record was to encourage compliance with the study protocol... On each test day, subjects reported to the laboratory at their designated lunchtime. At that time, the food and activity records were collected and subjects completed a brief questionnaire to determine whether they...had consumed alcohol in the previous 24 h... or had consumed any food or energy‐containing beverages in the 3 h preceding the test meal or water in the 1 h preceding the test meal. The experimenters reviewed the records and questionnaires to monitor compliance with the study protocol. Subjects who failed to comply with the protocol were scheduled for another test day. At the start of each test meal... [subjects] were instructed to eat as much or as little of the macaroni and cheese as desired and to drink as much or as little of the water as desired." Comment: information and instructions provided to participants appear to have been standardised between the compared study conditions. Participants' compliance with the instructions to keep their evening meal and their activity level as similar as possible on the day before each test day, to refrain from eating or drinking (except water) after 22:00, to refrain from drinking alcohol on the day before and throughout each test day, to eat a similar breakfast on the morning of each test day, not to consume any food or energy‐containing beverages for 3 hours before the test meal, not to drink water for 1 hour before the test meal, not to consume any food or energy‐containing beverages for 3 hours following the test meal, and to eat a similar dinner on the evening following each test meal was monitored via experimenter review of self report food and activity diary and self report questionnaire. Whilst no monitoring results are reported with respect to these instructions, it is reported that participants who failed to comply were rescheduled for another test day. No further specific instructions were provided to participants, other than the instructions to eat as much or as little of the macaroni and cheese as desired and to drink as much or as little of the water as desired
Summary of risk of bias Consumption outcome	Unclear risk	Unclear risk

**Table:** Rolls 2004a

Methods	Study design: within‐subjects randomised controlled trial	
Participants	Setting: laboratory setting Geographical region: Pennsylvania, USA Number of enrolled participants: 76 adults Number (%) of enrolled participants completing the study: 75 (98.7) Study completers ‐ mean age (SD): 25.0 (6.7) Study completers ‐ sex: 49.3% female Study completers ‐ mean BMI kg/m^2^ (SD) = 23.6 (3.2) Specific social or cultural characteristics: university community Socio‐economic status context: low deprivation Inclusion criteria: healthy non‐smoking individuals aged 20 to 45 years with a reported BMI less than 40, not dieting to gain or lose weight, not an athlete in training, not taking medications that affect appetite, who have no food restrictions or allergies, eat meals at regular times, and like the foods to be served in the study. Female subjects were also required to not be pregnant or lactating at the time of the study Exclusion criteria: score on the Eating Attitudes Test of 20 or more (indicating a potential eating disturbance) or their score on the Zung Self‐Rating Scale was 40 or more (indicating a likelihood of depression)	
Interventions	Manipulated product type: food Manipulation: portion size Duration of exposure to intervention: ≤ 1 day Social setting: consuming alone Study arms: 6‐inch sandwich (275 g); 8‐inch sandwich (376 g); 10‐inch sandwich (458 g); 12‐inch sandwich (550 g) Number of comparisons analysed: 3 Comparisons analysed: Comparison 1 = Intervention 1: 6‐inch sandwich (275 g); *versus* Intervention 2: 8‐inch sandwich (376 g); Comparison 2 = Intervention 1: 8‐inch sandwich (376 g); *versus* Intervention 2: 10‐inch sandwich (458 g); Comparison 3 = Intervention 1: 10‐inch sandwich (458 g); *versus* Intervention 2: 12‐inch sandwich (550 g) Concurrent intervention components: no	
Outcomes	Outcomes reported in study: total energy intake (kcal) from lunch meal; weight intake (g) Selection outcome analysed: N/A Measurement of selection outcome: N/A Timing of selection outcome measurement: N/A Consumption outcome analysed: energy intake from total lunch meal (kcal) Measurement of consumption outcome: objective Timing of consumption outcome measurement: immediate (≤ 1 day)	
Funding source	Not stated	
Notes	Study authors contacted for missing data with additional data received March 2014	
***Risk of bias***		
**Bias**	**Authors' judgement**	**Support for judgement**
Random sequence generation (selection bias)	Unclear risk	Comment: method of sequence generation for condition order is not described. Author contact confirmed condition order was randomised but no further details (13 March 2013). Insufficient information about the sequence generation process to permit judgement of 'low risk' or 'high risk'
Allocation concealment (selection bias)	Unclear risk	Comment: method of concealment is not described. Author contact confirmed condition order was randomised but no further details (13 March 2013). Insufficient information to permit judgement of 'low risk' or 'high risk'
Blinding of participants and personnel (performance bias) Consumption outcome	Unclear risk	Quote: "[Subjects] were not told the actual purpose of the study but were told that the purpose was to examine the perception of taste... At the end of the study, subjects also completed a discharge questionnaire, which asked what they thought the purpose of the study was... At discharge, the majority of subjects (83%) did not correctly discern the purpose of the study, but guessed that it related to perceptions of taste or hunger or to general nutrition. Only 13 subjects (17%) correctly reported that we were investigating the effect of portion size on food intake." Comment: no blinding or incomplete blinding. Participants were probed for suspicion of study purpose but not for awareness of size manipulation between study conditions. It appears that blinding of study participants was broken in at least some cases and it is possible that the outcome may be influenced by lack of blinding (due to potential carry‐over effects between conditions). Very unlikely that key study personnel were blinded, but the review authors judge that the outcome is not likely to be influenced by lack of blinding of key study personnel
Blinding of outcome assessment (detection bias) Consumption outcome	Low risk	Comment: no blinding of outcome assessment, but the review authors judge that the outcome measurement is not likely to be influenced by lack of blinding
Incomplete outcome data (attrition bias) Consumption outcome	Low risk	Quote: "Seventy‐six subjects began the study, but one female subject failed to return after the first test meal. Thus, 75 subjects completed the study: 37 females and 38 males." Comment: the reason for missing outcome data is unlikely to be related to consumption outcome
Selective reporting (reporting bias)	Unclear risk	Comment: search for record(s) containing details of study protocol conducted in ClinicalTrials.gov and the WHO International Clinical Trials Registry Platform (ICTRP). No records found. Insufficient information to permit judgement of 'low risk' or 'high risk'
Other bias #1 ‐ Baseline comparability of participant characteristics between groups	Unclear risk	Quote: "Subjects completed ratings of their hunger and satiety immediately before and after lunch. Subjects rated their hunger, thirst, prospective consumption (how much food they thought they could eat), nausea, and fullness on visual analog scales... Before lunch was served, ratings of hunger did not differ between experimental conditions... The pattern of results for ratings of prospective consumption was similar to that for hunger, and for ratings of fullness the pattern was similar but in the opposite direction." Comment: study uses a within‐subjects design. Differences between conditions in terms of measured pre‐condition participant 'state' characteristics are partially reported, but not reported whether there were differences between condition orders in terms of measured pre‐condition participant 'state' characteristics. No analysis of potential differences in measured outcomes between condition orders appears to have been conducted and the statistical analysis of outcome data does not appear to control for condition order. Risk of bias due to period effects is therefore unclear. Insufficient information to permit judgement of 'low risk' or 'high risk'
Other bias #2 ‐ Consistency in intervention delivery	Low risk	Quote: "Subjects were instructed to keep their meals and activity level consistent and to refrain from consuming alcohol on the evening before and the morning of each test day. They were also asked not to consume food or caloric beverages during the 3 hours before and after each test meal. Subjects completed a brief record of their physical activity on the evening before the test day and their food intake on the evening before and day of each test meal. At the beginning of each test meal, they also filled out a questionnaire that asked about...departures from the protocol. The food and activity records and the questionnaire were reviewed before the beginning of each test meal; subjects who ... did not comply with the protocol had their test meal rescheduled... Subjects were instructed to consume as much or as little of the sandwich and water as they desired..." Comment: information and instructions provided to participants appear to have been standardised between the compared study conditions. Participants' compliance with the instructions to keep their meals and activity level consistent, to refrain from consuming alcohol on the evening before and the morning of each test day, and not to consume food or caloric beverages during the 3 hours before and after each test meal was monitored via experimenter review of self report food and activity diary and self report questionnaire. Whilst no monitoring results are reported with respect to these instructions, it is reported that participants who failed to comply had their test meal rescheduled. No further specific instructions were provided to participants, other than the instructions to consume as much or as little of the sandwich and water as they desired
Summary of risk of bias Consumption outcome	Unclear risk	Unclear risk

**Table:** Rolls 2004b

Methods	Study design: within‐subjects randomised controlled trial	
Participants	Setting: laboratory setting Geographical region: Pennsylvania, USA Number of enrolled participants: 68 adults Number (%) of enrolled participants completing the study: 63 (92.6) Study completers ‐ mean age (SD): 22.8 (4.8) Study completers ‐ sex: 56.7% female Study completers ‐ mean BMI kg/m^2^ (SD): 23.2 (3.1) Specific social or cultural characteristics: no Socio‐economic status context: low deprivation Inclusion criteria: aged 20 to 45 y; regularly ate 3 meals per day; regularly snacked between meals and liked potato chips; were not dieting to gain or lose weight; were not using medication known to affect food intake or appetite, were not athletes in training; were not pregnant or lactating; had no food allergies or food restrictions that would affect food intake; were not smokers Exclusion criteria: BMI outside the range of 20 to 40 kg/m2; Scored 30 on the Eating Attitudes Test (EAT); scored 40 on the Zung Self‐Rating Questionnaire; disliked any of the foods to be served in the study	
Interventions	Manipulated product type: food Manipulation: portion with package size Duration of exposure to intervention: ≤ 1 day Social setting: consuming alone Study arms: 28 g pack of potato chips; 42 g pack of potato chips; 85 g pack of potato chips; 128 g pack of potato chips; 170 g pack of potato chips Number of comparisons analysed: 4 Comparisons analysed: Comparison 1 = Intervention 1: 28 g pack of potato chips; *versus* Intervention 2: 42 g pack of potato chips Comparison 2 = Intervention 1: 42 g pack of potato chips; *versus* Intervention 2: 85 g pack of potato chips Comparison 3 = Intervention 1: 85 g pack of potato chips; *versus* Intervention 2: 128 g pack of potato chips Comparison 4 = Intervention 1: 128 g pack of potato chips; *versus* Intervention 2: 170 g pack of potato chips Concurrent intervention components: no	
Outcomes	Outcomes reported in study: combined energy intake over snack and meal (kj) Selection outcome analysed: N/A Measurement of selection outcome: N/A Timing of selection outcome measurement: N/A Consumption outcome analysed: energy intake from snack and meal (kilojoules) Measurement of consumption outcome: objective Timing of consumption outcome measurement: immediate (≤ 1 day)	
Funding source	United States National Institutes of Health	
Notes	Study authors contacted for missing data with additional data received March 2014	
***Risk of bias***		
**Bias**	**Authors' judgement**	**Support for judgement**
Random sequence generation (selection bias)	Unclear risk	Comment: method of sequence generation for condition order is not described. Author contact confirmed condition order was randomised but no further details (13 March 2013). Insufficient information about the sequence generation process to permit judgement of 'low risk' or 'high risk'
Allocation concealment (selection bias)	Unclear risk	Comment: method of concealment is not described. Author contact confirmed condition order was randomised but no further details (13 March 2013). Insufficient information to permit judgement of 'low risk' or 'high risk'
Blinding of participants and personnel (performance bias) Consumption outcome	Unclear risk	Quote: "The subjects were informed that the purpose of the study was to examine the effects of consumption of snacks... At the end of their final session, subjects completed a discharge questionnaire, which asked what they thought was the purpose of the study, whether they noticed any differences between the test days, and whether potato chips were a usual snack food for them... Only one subject correctly discerned that the purpose of the study was to examine whether the size of the snack package affected snack intake. Forty subjects believed that the study investigated whether the amount of food consumed at the snack affected the amount eaten at dinner. Fifteen subjects reported more general purposes and four subjects reported that they did not know the aim of the study. All subjects except two reported that the package size of the snack varied across test days." Comment: no blinding or incomplete blinding. Participants were probed for suspicion of study purpose and awareness of size manipulation between study conditions. It appears that blinding of study participants was broken in the majority of cases and it is possible that the outcome may be influenced by lack of blinding (due to potential carry‐over effects between conditions). Very unlikely that key study personnel were blinded, but the review authors judge that the outcome is not likely to be influenced by lack of blinding of key study personnel
Blinding of outcome assessment (detection bias) Consumption outcome	Low risk	Comment: no blinding of outcome assessment, but the review authors judge that the outcome measurement is not likely to be influenced by lack of blinding
Incomplete outcome data (attrition bias) Consumption outcome	Low risk	Quote: "Sixty‐eight subjects were enrolled in the study. Five subjects withdrew from the study for personal reasons or because they could not attend according to schedule. Thus, 63 subjects completed the study. Three subjects were excluded from the analysis for repeatedly having low intakes at the snack (<10 g at three or more sessions)." Comment: the first reason for missing data for consumption outcome is participant withdrawal due to personal reasons or inability to attend study sessions. This reason for missing outcome data is unlikely to be related to consumption outcome. The second reason for missing outcome data for consumption outcome is the study authors' decision to exclude participants with consumption of the snack < 10 g at 3 or more sessions from the analysis. The low proportion (3 participants, 4% of study sample) of exclusions due to low consumption means that the review authors judge that the plausible effect size among missing outcomes is unlikely to be enough to have an important impact on the observed effect size
Selective reporting (reporting bias)	Unclear risk	Comment: search for record(s) containing details of study protocol conducted in ClinicalTrials.gov and the WHO International Clinical Trials Registry Platform (ICTRP). No records found. Insufficient information to permit judgement of 'low risk' or 'high risk'
Other bias #1 ‐ Baseline comparability of participant characteristics between groups	Unclear risk	Quote: "At the snack session, subjects rated characteristics of the potato chips using visual analog scales...Immediately after the snack was served, subjects were asked to open the package, take one bite of the snack, and complete ratings for pleasantness of taste...and how much of the food they felt they could consume (prospective consumption)...Subjects also completed ratings of hunger and fullness...immediately before...the snack...and before...dinner. Subjects rated their sensations of hunger and fullness on 100 mm visual analogue scales... Subject ratings of prospective consumption of the snack (how much of the food they thought they could consume) decreased significantly as the package size increased... Mean ratings of the pleasantness of taste of the snack prior to consumption did not differ by package size... Initial ratings of hunger before the snack was served did not differ across experimental conditions... Ratings of hunger between the snack and dinner decreased significantly with increasing package size." Comment: study uses a within‐subjects design. Differences between conditions in terms of measured pre‐condition participant 'state' characteristics, but not reported whether there were differences between condition orders in terms of measured pre‐condition participant 'state' characteristics. No analysis of potential differences in measured outcomes between condition orders appears to have been conducted and the statistical analysis of outcome data does not appear to control for condition order. Risk of bias due to period effects is therefore unclear. Insufficient information to permit judgement of 'low risk' or 'high risk'
Other bias #2 ‐ Consistency in intervention delivery	Low risk	Quote: "We asked subjects to eat a similar breakfast and lunch on test days, to eat lunch at least 2 h before the snack session, and to refrain from consuming any food or energy containing beverages for at least 3 h after the dinner session. Subjects were instructed not to drink anything except water between meals on test days, and to refrain from drinking water for 1 h before both the snack and dinner. We also instructed subjects to maintain a consistent activity level on the day before and the day of each test session. On each test day, subjects kept a brief record of the foods they had eaten and their physical activity, to assist them in following the protocol. Subjects reported to the laboratory at their designated snack time between 2 and 3 p.m. At this time, we collected the food and activity record and subjects completed a brief questionnaire about their...intake of... alcohol in the previous 24 hours, as well as any food intake since lunch. The records and questionnaire were reviewed in order to monitor compliance with the study protocol; subjects who failed to comply with the protocol had their test day rescheduled... We instructed subjects to consume as much or as little of the snack and water as they desired, and to eat the potato chips directly from the bag... Subjects returned to the laboratory for dinner between 5 and 6 p.m... Before dinner was served, subjects completed a second questionnaire about their physical well‐being and intake of food, medications and alcohol since the snack... Subjects were again instructed to eat and drink as much or as little of the food as they desired." Comment: information and instructions provided to participants appear to have been standardised between the compared study conditions. Participants' compliance with the instructions to eat a similar breakfast and lunch on test days, to eat lunch at least 2 h before the snack session, to refrain from consuming any food or energy containing beverages for at least 3 h after the dinner session, not to drink anything except water between meals on test days, to refrain from drinking water for 1 h before both the snack and dinner, and to maintain a consistent activity level on the day before and the day of each test session was monitored via experimenter review of self report food and activity diary and self report questionnaire. Whilst no monitoring results are reported with respect to these instructions, it is reported that participants who failed to comply had their test day rescheduled. No further specific instructions were provided to participants, other than the instructions to consume as much or as little of the snack and water as they desired, and to eat the potato chips directly from the bag
Summary of risk of bias Consumption outcome	Unclear risk	Unclear risk

**Table:** Rolls 2006a

Methods	Study design: within‐subjects randomised controlled trial	
Participants	Setting: laboratory setting Geographical region: Pennsylvania, USA Number of enrolled participants: 16 adult females; 16 adult males Number (%) of enrolled participants completing the study: adult females = 16 (100%); adult males = 16 (100%) Study completers ‐ mean age (SD): adult females = 21.2 (2.0); adult females = 24.4 (4.8). Study completers ‐ sex: adult females = female only (100%); adult males = male only (100%) Study completers ‐ mean BMI kg/m^2^ (SD): adult females = 22.2 (2.0); adult males = 24.7 (2.4) Specific social or cultural characteristics: no Socio‐economic status context: low deprivation Inclusion criteria: non‐smoking adults in good health; aged between 19 and 45 years; not dieting to gain or lose weight; not in athletic training; not pregnant or breastfeeding; not taking medications known to affect appetite; no food allergies or dislikes for the entrées and desserts served in the study; regularly consuming 3 meals per day Exclusion criteria: BMI < 19 or > 30; scored ≥ 40 on the Zung Self‐Rating Scale; scored ≥ 20 on the Eating Attitudes Test	
Interventions	Manipulated product type: food Manipulation: portion size Duration of exposure to intervention: > 1 day Social setting: consuming alone Study arms: total portion sizes of served foods and beverages over 2 days comprising 100% portion size; total portion sizes of served foods and beverages over 2 days comprising 150% portion size; total portion sizes of served foods and beverages over 2 days comprising 200% portion size Number of comparisons analysed: 4 (adult females = 2; adult males = 2) Comparisons analysed: adult females ‐ Comparison 1 = Intervention 1: total portion sizes of served foods and beverages over 2 days comprising 100% portion size; *versus* Intervention 2: total portion sizes of served foods and beverages over 2 days comprising 150% portion size; Comparison 2 = Intervention 1: total portion sizes of served foods and beverages over 2 days comprising 150% portion size; *versus* Intervention 2: total portion sizes of served foods and beverages over 2 days comprising 200% portion size Adult males ‐ Comparison 1 = Intervention 1: total portion sizes of served foods and beverages over 2 days comprising 100% portion size; *versus* Intervention 2: total portion sizes of served foods and beverages over 2 days comprising 150% portion size; Comparison 2 = Intervention 1: total portion sizes of served foods and beverages over 2 days comprising 150% portion size; *versus* Intervention 2: total portion sizes of served foods and beverages over 2 days comprising 200% portion size Concurrent intervention components: no	
Outcomes	Outcomes reported in study: males and females: total energy intake over 2 days (kcal) Selection outcome analysed: N/A Measurement of selection outcome: N/A Timing of selection outcome measurement: N/A Consumption outcome analysed: total energy intake over 2 days (kcal) Measurement of consumption outcome: objective Timing of consumption outcome measurement: longer‐term (> 1 day)	
Funding source	United States National Institutes of Health	
Notes	Outcome data for males and females analysed separately (2 comparisons each) because the absolute difference in portion size between reference size and large size portion conditions varied by sex. Study authors contacted for missing data with additional data received February 2014 and March 2014	
***Risk of bias***		
**Bias**	**Authors' judgement**	**Support for judgement**
Random sequence generation (selection bias)	Unclear risk	Comment: method of sequence generation for condition order is not described. Author contact confirmed condition order was randomised but no further details (13 March 2013). Insufficient information about the sequence generation process to permit judgement of 'low risk' or 'high risk'
Allocation concealment (selection bias)	Unclear risk	Comment: method of concealment is not described. Author contact confirmed condition order was randomised but no further details (13March 2013). Insufficient information to permit judgement of 'low risk' or 'high risk'
Blinding of participants and personnel (performance bias) Consumption outcome	Unclear risk	Quote: "...the consent form stated that the purpose of the experiment was to investigate the consumption of a variety of foods... At the end of the last study session, subjects completed a discharge questionnaire that asked them to report their ideas about the purpose of the study and any differences they noticed between study sessions. On the discharge questionnaire, 12 subjects (38%) correctly reported that the purpose of the study was to investigate the effect of the amount of food served on the amount eaten (among other purposes that were mentioned). The effect of portion size on intake was not influenced by whether or not subjects guessed the purpose of the study. When asked to describe differences between study weeks, 31 of the 32 subjects mentioned that the portion sizes of the foods changed, but only four subjects (13%) reported that the different portion sizes affected their food intake." Comment: no blinding or incomplete blinding. Participants were probed for suspicion of study purpose and awareness of size manipulation between study conditions. Blinding of study participants was broken in some cases and it is possible that the outcome may be influenced by lack of blinding (due to potential carry‐over effects between conditions). Very unlikely that key study personnel were blinded, but the review authors judge that the outcome is not likely to be influenced by lack of blinding of key study personnel
Blinding of outcome assessment (detection bias) Consumption outcome	Low risk	Comment: no blinding of outcome assessment, but the review authors judge that the outcome measurement is not likely to be influenced by lack of blinding
Incomplete outcome data (attrition bias) Consumption outcome	Low risk	Comment: no missing outcome data for consumption outcome
Selective reporting (reporting bias)	Unclear risk	Comment: search for record(s) containing details of study protocol conducted in ClinicalTrials.gov and the WHO International Clinical Trials Registry Platform (ICTRP). No records found. Insufficient information to permit judgement of 'low risk' or 'high risk'
Other bias #1 ‐ Baseline comparability of participant characteristics between groups	Unclear risk	Quote: "Subjects used visual analog scales to rate their hunger, prospective consumption (how much food they thought they could eat), and fullness immediately before...each meal in the laboratory... At the beginning of each meal, subjects took one bite of the food and [used visual analog scales to rate]...the pleasantness of taste and appearance... There was no significant difference in ratings of hunger and satiety between the 150% and 200% portion conditions in either sex... There were no significant differences according to portion size in ratings of pleasantness of taste or appearance." Comment: study uses a within‐subjects design. Differences between conditions in terms of measured pre‐condition participant 'state' characteristics are partially reported, but not reported whether there were differences between condition orders in terms of measured pre‐condition participant 'state' characteristics. No analysis of potential differences in measured outcomes between condition orders appears to have been conducted and the statistical analysis of outcome data does not appear to control for condition order. Risk of bias due to period effects is therefore unclear. Insufficient information to permit judgement of 'low risk' or 'high risk'
Other bias #2 ‐ Consistency in intervention delivery	Unclear risk	Quote: "Subjects could consume as much of the foods and beverages as they wanted. Subjects were instructed not to consume any foods or beverages other than those provided by the researchers during each 2‐day session, with the exception of water, which they could consume up to 1 hour before each meal. Subjects were also asked not to share with anyone else the snacks that were provided for consumption away from the laboratory. Subjects were instructed to keep their activity level consistent and to refrain from drinking alcohol on the day before and during each 2‐day session; to encourage compliance with the protocol, they kept a brief record of their activity on each of these days. Before each meal, subjects completed a brief questionnaire that asked if...they had...consumed any foods or beverages other than those provided by the researchers. If subjects...did not comply with the protocol, their test session was rescheduled." Comment: information and instructions provided to participants appear to have been standardised between the compared study conditions. Participants' compliance with the instructions not to consume any foods or beverages other than those provided by the researchers during each 2‐day session, with the exception of water, which they could consume up to 1 hour before each meal, to keep their activity level consistent and to refrain from drinking alcohol on the day before and during each 2‐day session was monitored via experimenter review of self report food and activity diary and self report questionnaire. Whilst no monitoring results are reported with respect to these instructions, it is reported that participants who failed to comply had their test session rescheduled. No information pertaining to monitoring of participants' compliance with the instruction not to share with anyone else the snacks that were provided for consumption away from the laboratory is reported. No further specific instructions were provided to participants, other than the instruction that they could consume as much of the test foods and beverages as they wanted
Summary of risk of bias Consumption outcome	Unclear risk	Unclear risk

**Table:** Rolls 2006b

Methods	Study design: within‐subjects randomised controlled trial	
Participants	Setting: laboratory setting Geographical region: Pennsylvania, USA Number of enrolled participants: 25 adults Number (%) of enrolled participants completing the study: 24 (96%) Study completers ‐ mean age (SD): 21.9 (3.4) Study completers ‐ sex: 100% female Study completers ‐ mean BMI kg/m^2^ (SD): 22.6 (2.9) Specific social or cultural characteristics: no Socio‐economic status context: low deprivation Inclusion criteria: women 19 to 45 y not following a diet to lose or gain weight; not in athletic training; not pregnant or breastfeeding; not receiving medications known to affect appetite or food intake; did not smoke; regularly ate 3 meals daily; had no food allergies or restrictions Exclusion criteria: BMI below 18 or above 40; scored 40 on the Zung self rating scale or 20 on the Eating Attitudes Test; disliked any of the entrées to be served at the meals	
Interventions	Manipulated product type: food Manipulation: portion size Duration of exposure to intervention: > 1 day Social setting: consuming alone Study arms: daily menus of 75% portion size ‐ high energy density; daily menus of 75% portion size ‐ low energy density; daily menus of 100% portion size ‐ high energy density; daily menus of 100% portion size ‐ low energy density Number of comparisons analysed: 1 Comparisons analysed: Comparison 1 = Intervention 1: 75% portion size; *versus* Intervention 2: 100% portion size Concurrent intervention components: yes. Manipulation of energy density	
Outcomes	Outcomes reported in study: total energy intake over 2 days (kcal/2d); weight of food consumed (g/2d) Selection outcome analysed: N/A Measurement of selection outcome: N/A Timing of selection outcome measurement: N/A Consumption outcome analysed: total energy intake over 2 days (kcal) Measurement of consumption outcome: objective Timing of consumption outcome measurement: longer‐term (> 1 day)	
Funding source	United States National Institutes of Health	
Notes	—	
***Risk of bias***		
**Bias**	**Authors' judgement**	**Support for judgement**
Random sequence generation (selection bias)	Unclear risk	Comment: method of sequence generation for condition order is not described. Author contact confirmed condition order was randomised but no further details (13 March 2013). Insufficient information about the sequence generation process to permit judgement of 'low risk' or 'high risk'
Allocation concealment (selection bias)	Unclear risk	Comment: method of concealment is not described. Author contact confirmed condition order was randomised but no further details (13 March 2013). Insufficient information to permit judgement of 'low risk' or 'high risk'
Blinding of participants and personnel (performance bias) Consumption outcome	Unclear risk	Quote: "At the end of the study, the subjects completed a discharge questionnaire that asked whether they noticed any differences between the sessions and what they thought the purpose of the study was... When asked at discharge about differences between the study sessions, 14 of the 24 women (58%) reported that portion sizes changed across the weeks...Five women (21%) correctly discerned that a purpose of the study was to test the effect of portion size on food intake, and 3 women (13%) correctly discerned that a purpose was to test the effect of energy content on intake. Only one subject correctly discerned both of these purposes. The effect of food portion size and energy density on total energy intake was still significant (P < 0.0001) after excluding the subjects who discerned either of the purposes of the study." Comment: no blinding or incomplete blinding. Participants were probed for suspicion of study purpose and awareness of size manipulation between study conditions. Blinding of study participants was broken in some cases and it is possible that the outcome may be influenced by lack of blinding (due to potential carry‐over effects between conditions). Very unlikely that key study personnel were blinded, but the review authors judge that the outcome is not likely to be influenced by lack of blinding of key study personnel
Blinding of outcome assessment (detection bias) Consumption outcome	Low risk	Comment: no blinding of outcome assessment, but the review authors judge that the outcome measurement is not likely to be influenced by lack of blinding
Incomplete outcome data (attrition bias) Consumption outcome	Low risk	Quote: "Twenty‐five women were enrolled in the study, but one was excluded for not attending a scheduled meal. Thus, a total of 24 women completed the study..." Comment: the reason for missing outcome data is unlikely to be related to consumption outcome
Selective reporting (reporting bias)	Unclear risk	Comment: search for record(s) containing details of study protocol conducted in ClinicalTrials.gov and the WHO International Clinical Trials Registry Platform (ICTRP). No records found. Insufficient information to permit judgement of 'low risk' or 'high risk'
Other bias #1 ‐ Baseline comparability of participant characteristics between groups	Unclear risk	Quote: "Immediately before...each main meal in the laboratory, the subjects rated their hunger, fullness, and prospective consumption (how much food they thought they could eat) by using visual analog scales... A summary measure of the hunger and satiety ratings over time was produced by calculating the area under the curve for each rating across the 2 d...The factors of session order and menu order were also assessed...The summary measure (area under the curve) of the ratings of fullness, hunger, and prospective consumption over the 2‐d session did not differ significantly across conditions (data not shown)." Comment: no differences between conditions in terms of measured pre‐condition participant 'state' characteristics are reported, but not reported whether there were differences between condition orders in terms of measured pre‐condition participant 'state' characteristics. Whilst analysis of potential differences in measured outcomes between condition orders appears to have been conducted, the results are not reported and it is unclear whether the statistical analysis of outcome data controls for any influence of condition order if present. Risk of bias due to period effects is therefore unclear. Insufficient information to permit judgement of 'low risk' or 'high risk'
Other bias #2 ‐ Consistency in intervention delivery	Unclear risk	Quote: "During each of the 2‐d sessions, the subjects were instructed to eat only the foods provided by the laboratory and to drink nothing else except water or noncaloric beverages. The subjects were asked to keep their activity level similar across the 4 test sessions...At each main meal, the subjects completed a brief report that asked whether they had...consumed any foods or caloric beverages other than those provided by the laboratory since the previous meal. Any subject who answered in the affirmative had their 2‐d test session rescheduled (in practice, only one subject had a session rescheduled)." Comment: information and instructions provided to participants appear to have been standardised between the compared study conditions. Participants' compliance with the instructions to eat only the foods provided by the laboratory and to drink nothing else except water or noncaloric beverages was monitored via written self report. It is reported that participants who failed to comply with these instructions had their test session rescheduled and that in practice only one subject had a session rescheduled. No information pertaining to monitoring of the instruction for participants to keep their activity level similar across the 4 test sessions is reported. No further specific instructions were provided to participants
Summary of risk of bias Consumption outcome	Unclear risk	Unclear risk

**Table:** Rolls 2007a

Methods	Study design: within‐subjects randomised controlled trial	
Participants	Setting: laboratory setting Geographical region: Pennsylvania, USA Number of enrolled participants: 27 adults Number (%) of enrolled participants completing the study: 23 adults (85.2%) Study completers ‐ mean age (SD): adult females = 25.8 (8.5); adult males = 24.7 (3.6) Study completers ‐ sex: adult females = female only; adult males = male only Study completers ‐ mean BMI kg/m^2^ (SD): adult females = 22.9 (2.5); adult males = 24.6 (2.9) Specific social or cultural characteristics: no Socio‐economic status context: low deprivation Inclusion criteria: non‐smoking adults in good health between the ages of 20 and 40 years; reported BMI between 18 and 30 kg/m2; regularly ate 3 meals per day; were not dieting to gain or to lose weight; were not athletes in training; were not taking medications known to affect appetite; were not pregnant or breastfeeding; had no food allergies or restrictions; liked and were willing to eat the primary foods to be served in the study; were willing to refrain from drinking alcohol during each 11‐day period Exclusion criteria: scored 40 on the Zung Self‐Rating Scale; scored 20 on the Eating Attitudes Test	
Interventions	Manipulated product type: food Manipulation: portion size Duration of exposure to intervention: > 1 day Social setting: consuming alone Study arms: all foods and beverages over 11 days in standard portions (100%); all foods and beverages over 11 days in larger portions (150%) Number of comparisons analysed: 2 (adult females = 1; adult males = 1) Comparisons analysed: Adult females ‐ Comparison 1 = Intervention 1: all foods and beverages over 11 days in standard women's portions (100%); *versus* Intervention 2: all foods and beverages over 11 days in larger women's portions (150%) Adult males ‐ Comparison 1 = Intervention 1: all foods and beverages over 11 days in standard men's portions (100%); *versus* Intervention 2: all foods and beverages over 11 days in larger men's portions (150%) Concurrent intervention components: no	
Outcomes	Outcomes reported in study: males and females: daily energy intake (kcal/day); total food and beverage weight (g/d) Selection outcome analysed: N/A Measurement of selection outcome: N/A Timing of selection outcome measurement: N/A Consumption outcome analysed: average (mean) daily energy intake (kcal) Measurement of consumption outcome: objective Timing of consumption outcome measurement: longer‐term (> 1 day)	
Funding source	United States National Institutes of Health	
Notes	Outcome data for males and females analysed separately (one comparison each) because the absolute difference in portion sizes varied by sex	
***Risk of bias***		
**Bias**	**Authors' judgement**	**Support for judgement**
Random sequence generation (selection bias)	Unclear risk	Comment: method of sequence generation for condition order is not described. Insufficient information about the sequence generation process to permit judgement of 'low risk' or 'high risk'
Allocation concealment (selection bias)	Unclear risk	Comment: method of concealment is not described. Insufficient information to permit judgement of 'low risk' or 'high risk'
Blinding of participants and personnel (performance bias) Consumption outcome	Unclear risk	Quote: "...the consent form stated that the purpose of the study was to investigate the interaction of foods over 11 days... At the end of the last meal in the laboratory, participants completed a discharge questionnaire, which asked them to report their ideas about the purpose of the study and any differences they noticed between the experimental sessions... When asked on the discharge questionnaire to describe differences between the two 11‐day sessions, 15 of the 23 participants (65%) reported that portion sizes were larger during one session, and a further 3 participants (13%) reported an increase in portion size for a few specific foods. Five participants (22%) did not report any differences between sessions. Nine of the 23 participants (39%) correctly determined that the purpose of the study was to test the effect of portion size on food intake. The effect of portion size on intake was significant both for participants who did and did not report the correct purpose of the study." Comment: no blinding or incomplete blinding. Participants were probed for suspicion of study purpose and awareness of size manipulation between study conditions. It appears that blinding of study participants was broken in the majority of cases and it is possible that the outcome may be influenced by lack of blinding (due to potential carry‐over effects between conditions). Very unlikely that key study personnel were blinded, but the review authors judge that the outcome is not likely to be influenced by lack of blinding of key study personnel
Blinding of outcome assessment (detection bias) Consumption outcome	Low risk	Comment: no blinding of outcome assessment, but the review authors judge that the outcome measurement is not likely to be influenced by lack of blinding
Incomplete outcome data (attrition bias) Consumption outcome	Unclear risk	Quote: "Twenty‐seven participants (13 women and 14 men) were enrolled in the study. Three participants were excluded from the study for failing to comply with the study schedule or protocol, and one was excluded for consuming substantially less than her estimated daily energy requirements...on multiple days (<1000 kcal/d). A total of 23 participants completed the study (10 women and 13 men)..." Comment: the first reason for missing outcome data for consumption outcome is failure to comply with the study schedule or protocol. The nature of the participants' failure to comply with the study protocol is not provided, so it is unclear whether this reason for exclusion is likely to be related to the study outcome or not. The second reason for missing outcome data for consumption outcome is the study authors' decision to exclude one participant consuming substantially less than their estimated daily energy requirements on multiple days from the analysis. Exceeding a threshold of 10% of missing outcome data for reasons that may be related to the outcome suggests that it is plausible that the effect size among these missing data is enough to have had an important impact on the observed effect size. Therefore, the review authors judge that the study is not at low risk of bias. However, the low proportion (1 participant, 4% of study sample) of exclusions due to low consumption means that it is unclear that the outcome is at high risk of bias. Insufficient information to permit judgement of 'low risk' or 'high risk'
Selective reporting (reporting bias)	Unclear risk	Comment: search for record(s) containing details of study protocol conducted in ClinicalTrials.gov and the WHO International Clinical Trials Registry Platform (ICTRP). No records found. Insufficient information to permit judgement of 'low risk' or 'high risk'
Other bias #1 ‐ Baseline comparability of participant characteristics between groups	Unclear risk	Quote: "Participants used visual analog scales to rate their hunger, fullness, and prospective consumption (how much food they thought they could eat) immediately before...each meal consumed in the laboratory...[The] influence of study day and menu sequence was also investigated...Ratings of hunger and satiety were summarized for each study day by calculating the area under the curve for a given rating over time... Serving large portion sizes had a significant effect on daily ratings of hunger and satiety (summarized by area under the curve). When large portions were served, mean daily ratings of fullness increased by 11%, ratings of hunger decreased by 9%, and ratings of prospective consumption decreased by 11% for both sexes compared with the baseline portion condition." Comment: differences between conditions in terms of measured pre‐condition participant 'state' characteristics, but not reported whether there were differences between condition orders in terms of measured pre‐condition participant 'state' characteristics. Whilst analysis of potential differences in measured outcomes between condition orders appears to have been conducted, the results are not reported and it is unclear whether the statistical analysis of outcome data controls for any influence of condition order if present. Risk of bias due to period effects is therefore unclear. Insufficient information to permit judgement of 'low risk' or 'high risk'
Other bias #2 ‐ Consistency in intervention delivery	Unclear risk	Quote: "Participants were instructed not to consume any foods or caloric beverages other than those provided by the laboratory during each 11‐day session... Participants were instructed not to share with others any of the snacks or meals provided for consumption away from the laboratory and were asked to keep their activity level consistent during each 11‐day session. To encourage compliance with the protocol, participants completed a questionnaire before all meals served in the laboratory. Participants were asked to report if they had...consumed any foods or caloric beverages not provided by the laboratory since their last meal. In addition, at breakfast, participants completed a record of all physical activity performed in the previous 24 hours... Three participants were excluded from the study for failing to comply with the study schedule or protocol...Participants were instructed to consume as much or as little of each food and beverage as they desired." Comment: information and instructions provided to participants appear to have been standardised between the compared study conditions. Participants' compliance with the instructions not to consume any foods or caloric beverages other than those provided by the laboratory during each 11‐day session and to keep their activity level consistent during each 11‐day session was monitored via self report questionnaire. It is reported that participants who failed to comply with the study schedule or protocol were excluded from the study and that in practice 3 participants were excluded for this reason. No information pertaining to monitoring of participants' compliance with the instruction not to share with others any of the snacks or meals provided for consumption away from the laboratory is reported. No further specific instructions were provided to participants, other than the instruction to consume as much or as little of each test food and beverage as they desired
Summary of risk of bias Consumption outcome	Unclear risk	Unclear risk

**Table:** Rolls 2007b (S1)

Methods	Study design: within‐subjects randomised controlled trial	
Participants	Setting: laboratory setting Geographical region: Pennsylvania, USA Number of enrolled participants: 47 adults Number (%) of enrolled participants completing the study: 45 (95.7) Study completers ‐ mean age (SD): = 22.1 (3.5) Study completers ‐ sex: 48.9% female Study completers ‐ mean BMI kg/m^2^ (SD): 22.8 (2.7) Specific social or cultural characteristics: no Socio‐economic status context: low deprivation Inclusion criteria: were not dieting to lose or gain weight; were not in athletic training; were not pregnant or breastfeeding; were not taking medications known to affect appetite or food intake; had no food allergies or restrictions; regularly ate 3 meals daily; did not smoke Exclusion criteria: individuals were not included in the study if they had a body mass index of ≤ 18 or ≥ 40 kg/m2, if they scored ≥ 40 on the Zung Self‐rating Scale or ≥ 20 on the Eating Attitudes Test, or if they reported disliking the foods to be served	
Interventions	Manipulated product type: food Manipulation: tableware Duration of exposure to intervention: ≤ 1 day Social setting: consuming alone Study arms: 17 cm plate used to self serve from large dish; 22 cm plate used to self‐ serve from large dish; 26 cm plate used to self serve from large dish Number of comparisons analysed: 2 Comparisons analysed: Comparison 1 = Intervention 1: plate diameter 17 cm; *versus* Intervention 2: plate diameter 22 cm Comparison 2 = Intervention 1: plate diameter 22 cm; *versus* Intervention 2: plate diameter 26 cm Concurrent intervention components: no	
Outcomes	Outcomes reported in study: total energy intake at meal (kJ and kcal); total food intake (grams); main course intake (g) Selection outcome analysed: N/A Measurement of selection outcome: N/A Timing of selection outcome measurement: N/A Consumption outcome analysed: energy intake from total lunch meal (kilojoules) Measurement of consumption outcome: objective Timing of consumption outcome measurement: immediate (≤ 1 day)	
Funding source	United States National Institutes of Health (National Institute of Diabetes and Digestive and Kidney Diseases)	
Notes	Study authors contacted for missing data with additional data received March 2014	
***Risk of bias***		
**Bias**	**Authors' judgement**	**Support for judgement**
Random sequence generation (selection bias)	Unclear risk	Comment: method of sequence generation for condition order is not described. Insufficient information about the sequence generation process to permit judgement of 'low risk' or 'high risk'
Allocation concealment (selection bias)	Unclear risk	Comment: method of concealment is not described. Insufficient information to permit judgement of 'low risk' or 'high risk'
Blinding of participants and personnel (performance bias) Consumption outcome	Unclear risk	Quote: "At the end of each study, participants completed a discharge questionnaire, which asked them to report any differences they noticed between the meals and their conjecture about the purpose of the experiment... At discharge, 11 participants (24%) reported that the plate size changed across the meals. Only one participant correctly determined that the purpose of the experiment was to test the influence of plate size on intake. Neither awareness of the change in plate size nor knowledge of the study purpose had a significant influence on lunch energy intake." Comment: no blinding or incomplete blinding. Participants were probed for suspicion of study purpose and awareness of size manipulation between study conditions. Blinding of study participants was broken in some cases and it is possible that the outcome may be influenced by lack of blinding (due to potential carry‐over effects between conditions). Very unlikely that key study personnel were blinded, but the review authors judge that the outcome is not likely to be influenced by lack of blinding of key study personnel
Blinding of outcome assessment (detection bias) Consumption outcome	Low risk	Comment: no blinding of outcome assessment, but the review authors judge that the outcome measurement is not likely to be influenced by lack of blinding
Incomplete outcome data (attrition bias) Consumption outcome	Low risk	Quote: "Forty‐seven participants were enrolled, but two participants withdrew from the study after attending one meal." Comment: the reason(s) for participants' withdrawal after attending one meal not provided, so it is unclear whether this reason for exclusion is likely to be related to the study outcome or not. The low proportion (2 participants, 4% of study sample) of exclusions means that the review authors judge that the plausible effect size among missing outcomes is unlikely to be enough to have an important impact on the observed effect size
Selective reporting (reporting bias)	Unclear risk	Comment: search for record(s) containing details of study protocol conducted in ClinicalTrials.gov and the WHO International Clinical Trials Registry Platform (ICTRP). No records found. Insufficient information to permit judgement of 'low risk' or 'high risk'
Other bias #1 ‐ Baseline comparability of participant characteristics between groups	Unclear risk	Quote: "Immediately before and after each experimental meal, participants rated their hunger, fullness, and prospective consumption (how much they thought they could eat) using visual analog scales... There were no significant differences in ratings of hunger and satiety across conditions of plate size... before...lunch." Comment: no differences between conditions in terms of measured pre‐condition participant 'state' characteristics, but not reported whether there were differences between condition orders in terms of measured pre‐condition participant 'state' characteristics. No analysis of potential differences in measured outcomes between condition orders appears to have been conducted and the statistical analysis of outcome data does not appear to control for condition order. Risk of bias due to period effects is therefore unclear. Insufficient information to permit judgement of 'low risk' or 'high risk'
Other bias #2 ‐ Consistency in intervention delivery	Unclear risk	Quote: "Participants were instructed to keep their food and activity level similar and to refrain from consuming alcohol on the day before each study day. In order to encourage compliance with this protocol, participants completed a brief record of food intake and physical activity... Participants were instructed not to consume any foods or beverages other than water between breakfast and lunch, and not to consume water for 1 h before lunch. Before lunch, participants completed a short questionnaire that evaluated whether...they had...consumed any food or beverages outside the laboratory since breakfast... Participants were instructed to serve the food from the dish onto the plate as often as they wanted, and to eat as much as they wanted from the plate." Comment: information and instructions provided to participants appear to have been standardised between the compared study conditions. Participants' compliance with the instructions to keep their food and activity level similar on the day before each study day, to refrain from consuming alcohol on the day before each study day, not to consume any foods or beverages other than water between breakfast and lunch, not to consume water for 1 hour before lunch was monitored via self report food intake and activity record and self report questionnaire; however no monitoring results are explicitly reported with respect to these instructions. No further specific instructions were provided to participants, other than the instructions to serve the test food from the dish onto the plate as often as they wanted, and to eat as much as they wanted from the plate
Summary of risk of bias Consumption outcome	Unclear risk	Unclear risk

**Table:** Rolls 2007b (S2)

Methods	Study design: within‐subjects randomised controlled trial	
Participants	Setting: laboratory setting Geographical region: Pennsylvania, USA Number of enrolled participants: 30 adults Number (%) of enrolled participants completing the study: 30 (100%) Study completers ‐ mean age (SD): = 27.2 (7) Study completers ‐ sex: 50% female Study completers ‐ mean BMI kg/m^2^ (SD): 23.8 (3.4) Specific social or cultural characteristics: no Socio‐economic status context: low deprivation Inclusion criteria: were not dieting to lose or gain weight; were not in athletic training; were not pregnant or breastfeeding; were not taking medications known to affect appetite or food intake; had no food allergies or restrictions; regularly ate 3 meals daily; did not smoke Exclusion criteria: individuals were not included in the study if they had a body mass index of ≤ 18 or ≥ 40 kg/m2, if they scored ≥ 40 on the Zung Self‐rating Scale or ≥ 20 on the Eating Attitudes Test, or if they reported disliking the foods to be served	
Interventions	Manipulated product type: food Manipulation: tableware Duration of exposure to intervention: ≤ 1 day Social setting: consuming alone Study arms: food received on 22 cm plate with small spoon used; food received on 26 cm plate with large spoon used (50% larger spoon) Number of comparisons analysed: 1 Comparisons analysed: Comparison 1 = Intervention 1: food received on 22 cm plate with small spoon used; *versus* Intervention 2: food received on 26 cm plate with large spoon used (50% larger spoon) Concurrent intervention components: no	
Outcomes	Outcomes reported in study: total energy intake at meal (kJ and kcal); total food intake (grams); main course intake (g) Selection outcome analysed: N/A Measurement of selection outcome: N/A Timing of selection outcome measurement: N/A Consumption outcome analysed: energy intake from total lunch meal (kilojoules) Measurement of consumption outcome: objective Timing of consumption outcome measurement: immediate (≤ 1 day)	
Funding source	United States National Institutes of Health (National Institute of Diabetes and Digestive and Kidney Diseases)	
Notes	—	
***Risk of bias***		
**Bias**	**Authors' judgement**	**Support for judgement**
Random sequence generation (selection bias)	Unclear risk	Comment: method of sequence generation for condition order is not described. Insufficient information about the sequence generation process to permit judgement of 'low risk' or 'high risk'
Allocation concealment (selection bias)	Unclear risk	Comment: method of concealment is not described. Insufficient information to permit judgement of 'low risk' or 'high risk'
Blinding of participants and personnel (performance bias) Consumption outcome	Unclear risk	Quote: "At the end of each study, participants completed a discharge questionnaire, which asked them to report any differences they noticed between the meals and their conjecture about the purpose of the experiment... At discharge, five participants (17%) reported that the plate size changed between the meals; two of these participants also noted the change in spoon size. None of the participants correctly determined the purpose of the experiment. An awareness of the change in plate size did not have a significant effect on lunch energy intake." Comment: no blinding or incomplete blinding. Participants were probed for suspicion of study purpose and awareness of size manipulation between study conditions. Blinding of study participants was broken in some cases and it is possible that the outcome may be influenced by lack of blinding (due to potential carry‐over effects between conditions). Very unlikely that key study personnel were blinded, but the review authors judge that the outcome is not likely to be influenced by lack of blinding of key study personnel
Blinding of outcome assessment (detection bias) Consumption outcome	Low risk	Comment: no blinding of outcome assessment, but the review authors judge that the outcome measurement is not likely to be influenced by lack of blinding
Incomplete outcome data (attrition bias) Consumption outcome	Low risk	Comment: no missing outcome data for consumption outcome
Selective reporting (reporting bias)	Unclear risk	Comment: search for record(s) containing details of study protocol conducted in ClinicalTrials.gov and the WHO International Clinical Trials Registry Platform (ICTRP). No records found. Insufficient information to permit judgement of 'low risk' or 'high risk'
Other bias #1 ‐ Baseline comparability of participant characteristics between groups	Unclear risk	Quote: "Immediately before and after each experimental meal, participants rated their hunger, fullness, and prospective consumption (how much they thought they could eat) using visual analog scales... There were no significant differences in ratings of hunger and satiety between conditions of plate size... before...lunch." Comment: no differences between conditions in terms of measured pre‐condition participant 'state' characteristics, but not reported whether there were differences between condition orders in terms of measured pre‐condition participant 'state' characteristics. No analysis of potential differences in measured outcomes between condition orders appears to have been conducted and the statistical analysis of outcome data does not appear to control for condition order. Risk of bias due to period effects is therefore unclear. Insufficient information to permit judgement of 'low risk' or 'high risk'
Other bias #2 ‐ Consistency in intervention delivery	Unclear risk	Quote: "Participants were instructed to keep their food and activity level similar and to refrain from consuming alcohol on the day before each study day. In order to encourage compliance with this protocol, participants completed a brief record of food intake and physical activity... Participants were instructed not to consume any foods or beverages other than water between breakfast and lunch, and not to consume water for 1 h before lunch. Before lunch, participants completed a short questionnaire that evaluated whether...they had...consumed any food or beverages outside the laboratory since breakfast... Participants were instructed to consume as much of the food as they wanted using the provided eating utensil." Comment: information and instructions provided to participants appear to have been standardised between the compared study conditions. Participants' compliance with the instructions to keep their food and activity level similar on the day before each study day, to refrain from consuming alcohol on the day before each study day, not to consume any foods or beverages other than water between breakfast and lunch, not to consume water for 1 hour before lunch was monitored via self report food intake and activity record and self report questionnaire; however no monitoring results are explicitly reported with respect to these instructions. No further specific instructions were provided to participants, other than the instruction to consume as much of the food as they wanted using the provided eating utensil
Summary of risk of bias Consumption outcome	Unclear risk	Unclear risk

**Table:** Rolls 2007b (S3)

Methods	Study design: within‐subjects randomised controlled trial	
Participants	Setting: laboratory setting Geographical region: Pennsylvania, USA Number of enrolled participants: 44 adults Number (%) of enrolled participants completing the study: 44 (100%) Study completers ‐ mean age (SD): = 22.7 (2.6) Study completers ‐ sex: 50% female Study completers ‐ mean BMI kg/m^2^ (SD): 22.6 (2.2) Specific social or cultural characteristics: no Socio‐economic status context: low deprivation Inclusion criteria: were not dieting to lose or gain weight; were not in athletic training; were not pregnant or breastfeeding; were not taking medications known to affect appetite or food intake; had no food allergies or restrictions; regularly ate 3 meals daily; did not smoke Exclusion criteria: individuals were not included in the study if they had a body mass index of ≤ 18 or ≥ 40 kg/m2, if they scored ≥ 40 on the Zung Self‐rating Scale or ≥ 20 on the Eating Attitudes Test, or if they reported disliking the foods to be served	
Interventions	Manipulated product type: food Manipulation: tableware Duration of exposure to intervention: ≤ 1 day Social setting: consuming alone Study arms: 17 cm plate used to self serve from buffet; 22 cm plate used to self serve from buffet; 26 cm plate used to self serve from buffet Number of comparisons analysed: 2 Comparisons analysed: Comparison 1 = Intervention 1: plate diameter 17 cm; *versus* Intervention 2: plate diameter 22 cm Comparison 2 = Intervention 1: plate diameter 22 cm; *versus* Intervention 2: plate diameter 26 cm Concurrent intervention components: no	
Outcomes	Outcomes reported in study: total energy intake at meal (kJ), total food intake (g) Selection outcome analysed: N/A Measurement of selection outcome: N/A Timing of selection outcome measurement: N/A Consumption outcome analysed: energy intake from total lunch meal (kilojoules) Measurement of consumption outcome: objective Timing of consumption outcome measurement: immediate (≤ 1 day)	
Funding source	United States National Institutes of Health (National Institute of Diabetes and Digestive and Kidney Diseases)	
Notes	Study authors contacted for missing data with additional data received March 2014	
***Risk of bias***		
**Bias**	**Authors' judgement**	**Support for judgement**
Random sequence generation (selection bias)	Unclear risk	Comment: method of sequence generation for condition order is not described. Insufficient information about the sequence generation process to permit judgement of 'low risk' or 'high risk'
Allocation concealment (selection bias)	Unclear risk	Comment: method of concealment is not described. Insufficient information to permit judgement of 'low risk' or 'high risk'
Blinding of participants and personnel (performance bias) Consumption outcome	Unclear risk	Quote: "At the end of each study, participants completed a discharge questionnaire, which asked them to report any differences they noticed between the meals and their conjecture about the purpose of the experiment... At discharge, 38 (86%) of the participants reported noticing a difference in plate size, and 24 of these participants (55%) guessed the purpose of the study. Neither awareness of the change in plate size nor knowledge of the study purpose had a significant influence on lunch energy intake." Comment: no blinding or incomplete blinding. Participants were probed for suspicion of study purpose and awareness of size manipulation between study conditions. Blinding of study participants appears to have been broken in the majority of cases and it is possible that the outcome may be influenced by lack of blinding (due to potential carry‐over effects between conditions). Very unlikely that key study personnel were blinded, but the review authors judge that the outcome is not likely to be influenced by lack of blinding of key study personnel
Blinding of outcome assessment (detection bias) Consumption outcome	Low risk	Comment: no blinding of outcome assessment, but the review authors judge that the outcome measurement is not likely to be influenced by lack of blinding
Incomplete outcome data (attrition bias) Consumption outcome	Low risk	Comment: no missing outcome data for consumption outcome
Selective reporting (reporting bias)	Unclear risk	Comment: search for record(s) containing details of study protocol conducted in ClinicalTrials.gov and the WHO International Clinical Trials Registry Platform (ICTRP). No records found. Insufficient information to permit judgement of 'low risk' or 'high risk'
Other bias #1 ‐ Baseline comparability of participant characteristics between groups	Unclear risk	Quote: "Immediately before and after each experimental meal, participants rated their hunger, fullness, and prospective consumption (how much they thought they could eat) using visual analog scales... There were no significant differences in ratings of hunger and satiety across conditions of plate size... before...lunch." Comment: no differences between conditions in terms of measured pre‐condition participant 'state' characteristics, but not reported whether there were differences between condition orders in terms of measured pre‐condition participant 'state' characteristics. No analysis of potential differences in measured outcomes between condition orders appears to have been conducted and the statistical analysis of outcome data does not appear to control for condition order. Risk of bias due to period effects is therefore unclear. Insufficient information to permit judgement of 'low risk' or 'high risk'
Other bias #2 ‐ Consistency in intervention delivery	Unclear risk	Quote: "Participants were instructed to keep their food and activity level similar and to refrain from consuming alcohol on the day before each study day. In order to encourage compliance with this protocol, participants completed a brief record of food intake and physical activity... Participants were instructed not to consume any foods or beverages other than water between breakfast and lunch, and not to consume water for 1 h before lunch. Before lunch, participants completed a short questionnaire that evaluated whether...they had...consumed any food or beverages outside the laboratory since breakfast... Participants were instructed to walk to their personal buffet, serve their chosen foods onto the plate, and return to their dining cubicle to eat. Participants could return to their buffet as often as they wanted, and eat as much as they wanted." Comment: information and instructions provided to participants appear to have been standardised between the compared study conditions. Participants' compliance with the instructions to keep their food and activity level similar on the day before each study day, to refrain from consuming alcohol on the day before each study day, not to consume any foods or beverages other than water between breakfast and lunch, not to consume water for 1 hour before lunch was monitored via self report food intake and activity record and self report questionnaire; however no monitoring results are explicitly reported with respect to these instructions. No further specific instructions were provided to participants, other than the instructions that they could return to their buffet as often as they wanted, and eat as much as they wanted
Summary of risk of bias Consumption outcome	Unclear risk	Unclear risk

**Table:** Rolls 2010a (E1)

Methods	Study design: within‐subjects randomised controlled trial	
Participants	Setting: laboratory setting Geographical region: Pennsylvania, USA Number of enrolled participants: 52 adults Number (%) of enrolled participants completing the study: 49 (94.2%) Study completers ‐ mean age (SD): 26.8 (6.9) Study completers ‐ sex: 49% female Study completers ‐ mean BMI kg/m^2^ (SD): 24.1 (3.3) Specific social or cultural characteristics: no Socio‐economic status context: low deprivation Inclusion criteria: between the ages of 20 and 45 y; reported BMI between 18 and 40; regularly ate 3 meals/d; reported liking and being willing to eat all 3 foods to be served in the test meal Exclusion criteria: dieting to gain or lose weight; had food allergies or restrictions; taking medications known to affect appetite; were smokers; were athletes in training; were pregnant or breastfeeding	
Interventions	Manipulated product type: food Manipulation: portion size Duration of exposure to intervention: ≤ 1 day Social setting: consuming alone Study arms: vegetable portion size of 180 g (in addition to the meal) ‐ high energy density; vegetable portion of 180 g (in addition to the meal) ‐ low energy density; vegetable portion of 270 g (in addition to the meal) ‐ high energy density; vegetable portion of 270 g (in addition to the meal) ‐ low energy density; vegetable portion of 360 g (in addition to the meal) ‐ high energy density; vegetable portion of 360 g (in addition to the meal) ‐ low energy density Number of comparisons analysed: 2 Comparisons analysed: Comparison 1 = Intervention 1: vegetable portion of 180 g; *versus* Intervention 2: vegetable portion of 270 g Comparison 2 = Intervention 1: vegetable portion of 270 g; *versus* Intervention 2: vegetable portion of 360 g Concurrent intervention components: yes. Low versus high energy density vegetable portion	
Outcomes	Outcomes reported in study: total meal energy intake (kcal); total meal intake (g); overall energy density of the meal (kcal/g); intake of vegetable (kcal and g); intake of grain (kcal and g); intake of meat (kcal and g) Selection outcome analysed: N/A Measurement of selection outcome: N/A Timing of selection outcome measurement: N/A Consumption outcome analysed: energy intake from total lunch meal (kcal) Measurement of consumption outcome: objective Timing of consumption outcome measurement: immediate (≤ 1 day)	
Funding source	United States National Institutes of Health	
Notes	Study authors contacted for missing data with additional data received March 2014	
***Risk of bias***		
**Bias**	**Authors' judgement**	**Support for judgement**
Random sequence generation (selection bias)	Unclear risk	Comment: method of sequence generation for condition order is not described. Insufficient information about the sequence generation process to permit judgement of 'low risk' or 'high risk'
Allocation concealment (selection bias)	Unclear risk	Comment: method of concealment is not described. Insufficient information to permit judgement of 'low risk' or 'high risk'
Blinding of participants and personnel (performance bias) Consumption outcome	Unclear risk	Quote: "The consent form stated that the purpose of the study was to investigate the perceptions of different tastes at a meal... On the final test day, participants completed a discharge questionnaire after lunch in which they ...were...asked their opinion of the purpose of the study and whether they noticed any differences between the sessions... On the discharge questionnaire in the addition study, 22 participants (45%) noted that some portion sizes changed across the weeks... Only 13 participants (27%) in the addition study ... correctly stated that a purpose of the study was to examine the influence of portion size on intake. The effects of the experimental variables on meal energy intake did not differ significantly between participants who did and did not correctly determine the study purpose." Comment: no blinding or incomplete blinding. Participants were probed for suspicion of study purpose and awareness of size manipulation between study conditions. Blinding of study participants was broken in some cases and it is possible that the outcome may be influenced by lack of blinding (due to potential carry‐over effects between conditions). Very unlikely that key study personnel were blinded, but the review authors judge that the outcome is not likely to be influenced by lack of blinding of key study personnel
Blinding of outcome assessment (detection bias) Consumption outcome	Low risk	Comment: no blinding of outcome assessment, but the review authors judge that the outcome measurement is not likely to be influenced by lack of blinding
Incomplete outcome data (attrition bias) Consumption outcome	Low risk	Quote: "Three participants were excluded from the addition study for failure to arrive for scheduled meals. Thus, 49 participants completed the addition study..." Comment: reason for missing outcome data is unlikely to be related to consumption outcome
Selective reporting (reporting bias)	Unclear risk	Comment: search for record(s) containing details of study protocol conducted in ClinicalTrials.gov and the WHO International Clinical Trials Registry Platform (ICTRP). No records found. Insufficient information to permit judgement of 'low risk' or 'high risk'
Other bias #1 ‐ Baseline comparability of participant characteristics between groups	Low risk	Quote: "Participants rated their hunger, fullness, and prospective consumption (how much they thought they could eat) immediately before...each meal by using visual analog scales... the ratings of hunger and satiety measured after the meal were adjusted by including the before‐meal rating as a covariate in the model...Interactions of factors [inc. portion size and study week] were tested for significance before examining their main effects...[Ratings of hunger, fullness, and prospective consumption] did not differ significantly by...vegetable portion size...(data not shown)." Comment: no differences between conditions in terms of measured pre‐condition participant 'state' characteristics , but not reported whether there were differences between condition orders in terms of measured pre‐condition participant 'state' characteristics. Analysis of potential differences in measured outcomes between condition orders appears to have been conducted and it appears likely that the statistical analysis controls for any potential influence of condition order on measured outcomes ("Interactions of factors [inc. portion size and study week] were tested for significance before examining their main effects"). It is therefore unlikely that any differences between condition orders in terms of unmeasured pre‐condition participant 'state' characteristics influenced the measured outcomes. Risk of bias due to period effects is therefore judged low
Other bias #2 ‐ Consistency in intervention delivery	Low risk	Quote: "On the day before each test day, participants were instructed to keep their evening meal and their physical activity level consistent and to refrain from drinking alcoholic beverages during the evening. To encourage compliance with this protocol, participants kept a brief record of their food and beverage intake and activity on the day before each test day... Participants were instructed not to consume any foods or beverages, other than water, between breakfast and lunch and not to drink any water during the hour before lunch. Before being served breakfast, participants were given a brief questionnaire that asked whether they had consumed any foods or beverages since waking... A similar questionnaire was completed before lunch. If participants...did not comply with the study protocol, their test day was rescheduled. During all meals, participants...instructed to consume as much of the foods and beverages as they wanted." Comment: information and instructions provided to participants appear to have been standardised between the compared study conditions. Participants' compliance with the instructions to keep their evening meal and their physical activity level consistent on the day before each test day, to refrain from drinking alcoholic beverages during the evening on the day before each test day, not to consume any foods or beverages, other than water, between breakfast and lunch and not to drink any water during the hour before lunch was monitored via self report food and beverage intake and activity record and self report questionnaire. Whilst no monitoring results are reported with respect to these instructions, it is reported that participants who failed to comply with these instructions had their test day rescheduled. No further specific instructions were provided to participants, other than the instruction to consume as much of the test foods and beverages as they wanted
Summary of risk of bias Consumption outcome	Unclear risk	Unclear risk

**Table:** Rolls 2010b (E2)

Methods	Study design: within‐subjects randomised controlled trial	
Participants	Setting: laboratory setting Geographical region: Pennsylvania, USA Number of enrolled participants: 48 adults Number (%) of enrolled participants completing the study: 48 (100%) Study completers ‐ mean age (SD): 26.7 (7) Study completers ‐ sex: 49% female Study completers ‐ mean BMI kg/m^2^ (SD): 23.6 (3) Specific social or cultural characteristics: no Socio‐economic status context: low deprivation Inclusion criteria: between the ages of 20 and 45 y; reported BMI between 18 and 40; regularly ate 3 meals/d; reported liking and being willing to eat all 3 foods to be served in the test meal Exclusion criteria: dieting to gain or lose weight; had food allergies or restrictions; taking medications known to affect appetite; were smokers; were athletes in training; were pregnant or breastfeeding	
Interventions	Manipulated product type: food Manipulation: portion size Duration of exposure to intervention: ≤ 1 day Social setting: consuming alone Study arms: vegetable portion size of 180 g (in substitution) ‐ high energy density; vegetable portion of 180 g (in substitution) ‐ low energy density; vegetable portion of 270 g (in substitution) ‐ high energy density; vegetable portion of 270 g (in substitution) ‐ low energy density; vegetable portion of 360 g (in substitution) ‐ high energy density; vegetable portion of 360 g (in substitution) ‐ low energy density Number of comparisons analysed: 2 Comparisons analysed: Comparison 1 = Intervention 1: vegetable portion of 180 g; *versus* Intervention 2: vegetable portion of 270 g Comparison 2 = Intervention 1: vegetable portion of 270 g; *versus* Intervention 2: vegetable portion of 360 g Concurrent intervention components: yes. Low versus high energy density vegetable portion	
Outcomes	Outcomes reported in study: total meal energy intake (kcal); total meal intake (g); overall energy density of the meal (kcal/g); intake of vegetable (kcal and g); intake of grain (kcal and g); intake of meat (kcal and g) Selection outcome analysed: N/A Measurement of selection outcome: N/A Timing of selection outcome measurement: N/A Consumption outcome analysed: energy intake from total lunch meal (kcal) Measurement of consumption outcome: objective Timing of consumption outcome measurement: immediate (≤ 1 day)	
Funding source	United States National Institutes of Health	
Notes	Study authors contacted for missing data with additional data received March 2014	
***Risk of bias***		
**Bias**	**Authors' judgement**	**Support for judgement**
Random sequence generation (selection bias)	Unclear risk	Comment: method of sequence generation for condition order is not described. Insufficient information about the sequence generation process to permit judgement of 'low risk' or 'high risk'
Allocation concealment (selection bias)	Unclear risk	Comment: method of concealment is not described. Insufficient information to permit judgement of 'low risk' or 'high risk'
Blinding of participants and personnel (performance bias) Consumption outcome	Unclear risk	Quote: "The consent form stated that the purpose of the study was to investigate the perceptions of different tastes at a meal... On the final test day, participants completed a discharge questionnaire after lunch in which they ...were...asked their opinion of the purpose of the study and whether they noticed any differences between the sessions... In the substitution study, 41 participants (85%) noted some change in portion sizes, most often of the vegetable. Only... 8 participants (17%) in the substitution study correctly stated that a purpose of the study was to examine the influence of portion size on intake. The effects of the experimental variables on meal energy intake did not differ significantly between participants who did and did not correctly determine the study purpose." Comment: no blinding or incomplete blinding. Participants were probed for suspicion of study purpose and awareness of size manipulation between study conditions. Blinding of study participants was broken in some cases and it is possible that the outcome may be influenced by lack of blinding (due to potential carry‐over effects between conditions). Very unlikely that key study personnel were blinded, but the review authors judge that the outcome is not likely to be influenced by lack of blinding of key study personnel
Blinding of outcome assessment (detection bias) Consumption outcome	Low risk	Comment: no blinding of outcome assessment, but the review authors judge that the outcome measurement is not likely to be influenced by lack of blinding
Incomplete outcome data (attrition bias) Consumption outcome	Low risk	Comment: no missing outcome data for consumption outcome
Selective reporting (reporting bias)	Unclear risk	Comment: search for record(s) containing details of study protocol conducted in ClinicalTrials.gov and the WHO International Clinical Trials Registry Platform (ICTRP). No records found. Insufficient information to permit judgement of 'low risk' or 'high risk'
Other bias #1 ‐ Baseline comparability of participant characteristics between groups	Low risk	Quote: "Participants rated their hunger, fullness, and prospective consumption (how much they thought they could eat) immediately before...each meal by using visual analog scales... the ratings of hunger and satiety measured after the meal were adjusted by including the before‐meal rating as a covariate in the model...Interactions of factors [inc. portion size and study week] were tested for significance before examining their main effects...[Ratings of hunger, fullness, and prospective consumption] did not differ significantly by...vegetable portion size...(data not shown)." Comment: no differences between conditions in terms of measured pre‐condition participant 'state' characteristics , but not reported whether there were differences between condition orders in terms of measured pre‐condition participant 'state' characteristics. Analysis of potential differences in measured outcomes between condition orders appears to have been conducted and it appears likely that the statistical analysis controls for any potential influence of condition order on measured outcomes ("Interactions of factors [inc. portion size and study week] were tested for significance before examining their main effects"). It is therefore unlikely that any differences between condition orders in terms of unmeasured pre‐condition participant 'state' characteristics influenced the measured outcomes. Risk of bias due to period effects is therefore judged low
Other bias #2 ‐ Consistency in intervention delivery	Low risk	Quote: "On the day before each test day, participants were instructed to keep their evening meal and their physical activity level consistent and to refrain from drinking alcoholic beverages during the evening. To encourage compliance with this protocol, participants kept a brief record of their food and beverage intake and activity on the day before each test day... Participants were instructed not to consume any foods or beverages, other than water, between breakfast and lunch and not to drink any water during the hour before lunch. Before being served breakfast, participants were given a brief questionnaire that asked whether they had consumed any foods or beverages since waking... A similar questionnaire was completed before lunch. If participants...did not comply with the study protocol, their test day was rescheduled. During all meals, participants...instructed to consume as much of the foods and beverages as they wanted." Comment: information and instructions provided to participants appear to have been standardised between the compared study conditions. Participants' compliance with the instructions to keep their evening meal and their physical activity level consistent on the day before each test day, to refrain from drinking alcoholic beverages during the evening on the day before each test day, not to consume any foods or beverages, other than water, between breakfast and lunch and not to drink any water during the hour before lunch was monitored via self report food and beverage intake and activity record and self report questionnaire. Whilst no monitoring results are reported with respect to these instructions, it is reported that participants who failed to comply with these instructions had their test day rescheduled. No further specific instructions were provided to participants, other than the instruction to consume as much of the test foods and beverages as they wanted
Summary of risk of bias Consumption outcome	Unclear risk	Unclear risk

**Table:** Russell 1980

Methods	Study design: within‐subjects randomised controlled trial	
Participants	Setting: field setting. Community Geographical region: London, UK Number of enrolled participants: 14 adults Number (%) of enrolled participants completing the study: 10 (71.4%) Study completers ‐ mean age (SD): 41 (not reported) Study completers ‐ sex: 90% female Study completers ‐ mean BMI kg/m^2^ (SD): not reported Specific social or cultural characteristics: no Socio‐economic status context: low deprivation Inclusion criteria: cigarette smokers Exclusion criteria: not stated	
Interventions	Manipulated product type: tobacco Manipulation: individual unit size Duration of exposure to intervention: > 1 day Social setting: consuming alone and with others Study arms: full‐length cigarettes; 3/4 length cigarettes; 1/2 length cigarettes Number of comparisons analysed: 2 Comparisons analysed: Comparison 1 = Intervention 1: 1/2 length cigarette; *versus* Intervention 2: 3/4 length cigarette Comparison 2 = Intervention 1: 3/4 length cigarette; *versus* Intervention 2:– full‐length cigarette Concurrent intervention components: no	
Outcomes	Outcomes reported in study: cigarette consumption; puff rate; mouth‐level nicotine intake; intake to the lungs (plasma nicotine); intake to the lungs (% COHb level) Selection outcome analysed: N/A Measurement of selection outcome: N/A Timing of selection outcome measurement: N/A Consumption outcome analysed: intake to the lungs (% COHb level) Measurement of consumption outcome: objective Timing of consumption outcome measurement: longer‐term (> 1 day)	
Funding source	UK Medical Research Council	
Notes	Study authors contacted for missing data but data no longer available	
***Risk of bias***		
**Bias**	**Authors' judgement**	**Support for judgement**
Random sequence generation (selection bias)	Low risk	Comment: method of sequence generation for condition order is not described. Author contact confirmed condition order was randomised and author stated that sequence for condition order was generated using a "highly complex number pattern" (13 March 2013)
Allocation concealment (selection bias)	Unclear risk	Comment: method of concealment is not described. Author contact confirmed condition order was randomised and author stated that sequence for condition order was generated using a "highly complex number pattern" (13 March 2013). Insufficient information to permit judgement of 'low risk' or 'high risk'
Blinding of participants and personnel (performance bias) Consumption outcome	Unclear risk	Comment: no blinding of study participants and it is possible that the outcome may be influenced by lack of blinding (due to potential carry‐over effects between conditions). Very unlikely that key study personnel were blinded, but the review authors judge that the outcome is not likely to be influenced by lack of blinding of key study personnel
Blinding of outcome assessment (detection bias) Consumption outcome	Low risk	Comment: no blinding of outcome assessment, but the review authors judge that the outcome measurement is not likely to be influenced by lack of blinding
Incomplete outcome data (attrition bias) Consumption outcome	Unclear risk	Quote: "Fourteen cigarette smokers took part in the study but due to missing data, 4 were excluded from the final analysis. Three of the latter smoked untipped cigarettes so that nicotine deliveries could not be calculated from butt content." Comment: the first reason for missing data for consumption outcome is exclusion due to 3 participants' own brands being untipped cigarettes, which precluded measurement of some consumption outcomes. It is unclear whether this reason for exclusion is likely to be related to consumption outcome. No reason for exclusion is provided for a fourth participant with missing outcome data for consumption outcome. Exceeding a threshold of 10% of missing outcome data for reasons that may be related to the outcome suggests that it is plausible that the effect size among these missing data is enough to have had an important impact on the observed effect size. Therefore, the review authors judge that the study is not at low risk of bias. Insufficient information to permit judgement of 'low risk' or 'high risk'
Selective reporting (reporting bias)	Unclear risk	Comment: search for record(s) containing details of study protocol conducted in ClinicalTrials.gov and the WHO International Clinical Trials Registry Platform (ICTRP). No records found. Insufficient information to permit judgement of 'low risk' or 'high risk'
Other bias #1 ‐ Baseline comparability of participant characteristics between groups	Low risk	Quote: "These data were analysed by a series of analyses of variance, the factors being length of cigarette, days, and order of receiving the different lengths." Comment: differences between conditions in terms of measured pre‐condition participant 'state' characteristics are reported. However, the statistical analysis appears to control for condition order. It is therefore unlikely that any differences between condition orders in terms of measured pre‐condition participant 'state' characteristics influenced the measured outcomes. Risk of bias due to period effects is therefore judged low
Other bias #2 ‐ Consistency in intervention delivery	Low risk	Quote: "[Participants] were...instructed to smoke as much or as little as they felt inclined." Comment: information and instructions provided to participants appear to have been standardised between the compared study conditions. No specific instructions were provided to participants, other than the instruction to smoke as much or as little as they felt inclined
Summary of risk of bias Consumption outcome	Unclear risk	Unclear risk

**Table:** Scott 2008b (S2)

Methods	Study design: between‐subjects randomised controlled trial	
Participants	Setting: laboratory setting Geographical region: Arizona, USA Number of enrolled participants: 385 adults Number (%) of enrolled participants completing the study: 385 (100%) Study completers ‐ mean age (SD): not reported Study completers ‐ sex: not reported Study completers ‐ mean BMI kg/m^2^ (SD): not reported Specific social or cultural characteristics: yes. University students Socio‐economic status context: low deprivation Inclusion criteria: not stated Exclusion criteria: not stated	
Interventions	Manipulated product type: food Manipulation: package size Duration of exposure to intervention: ≤ 1 day Social setting: consuming alone Study arms: small food, small packages (200 calories of mini‐M&Ms evenly distributed across four small bags); large food, large package (200 calories of regular M&Ms in one large bag) Number of comparisons analysed: 1 Comparisons analysed: comparison 1 = Intervention 1: mini‐M&Ms in 4 small bags; *versus* Intervention 2: regular M&Ms in one large bag Concurrent intervention components: yes. Concurrent individual unit size manipulation	
Outcomes	Outcomes reported in study: energy intake from M&Ms; binary variable of consuming all the food presented or not Selection outcome analysed: N/A Measurement of selection outcome: N/A Timing of selection outcome measurement: N/A Consumption outcome analysed: energy intake from M&Ms (kcal) Measurement of consumption outcome: objective Timing of consumption outcome measurement: immediate (≤ 1 day)	
Funding source	Association for Consumer Research	
Notes	Study authors contacted for missing data but data no longer available Package size manipulation confounded with individual unit size manipulation and therefore coded as package size manipulation	
***Risk of bias***		
**Bias**	**Authors' judgement**	**Support for judgement**
Random sequence generation (selection bias)	Unclear risk	Comment: method of sequence generation is not described. Author contact confirmed group assignment was randomised but no further details relating to method of sequence generation for assignment to package/unit size groups (13 March 2013). Insufficient information about the sequence generation process to permit judgement of 'low risk' or 'high risk'
Allocation concealment (selection bias)	Unclear risk	Comment: method of concealment is not described. Author contact confirmed group assignment was randomised but no further details relating to method of sequence generation for assignment to package/unit size groups (13 March 2013). Insufficient information to permit judgement of 'low risk' or 'high risk'
Blinding of participants and personnel (performance bias) Consumption outcome	Low risk	Comment: blinding of study participants attempted and unlikely that the blinding could have been broken. Very unlikely that key study personnel were blinded, but the review authors judge that the outcome is not likely to be influenced by lack of blinding of key study personnel
Blinding of outcome assessment (detection bias) Consumption outcome	Low risk	Comment: no blinding of outcome assessment, but the review authors judge that the outcome measurement is not likely to be influenced by lack of blinding
Incomplete outcome data (attrition bias) Consumption outcome	Low risk	Comment: no missing outcome data for consumption outcome
Selective reporting (reporting bias)	Unclear risk	Comment: search for record(s) containing details of study protocol conducted in ClinicalTrials.gov and the WHO International Clinical Trials Registry Platform (ICTRP). No records found. Insufficient information to permit judgement of 'low risk' or 'high risk'
Other bias #1 ‐ Baseline comparability of participant characteristics between groups	Unclear risk	Comment: study uses a between‐subjects design. Insufficient information to permit judgement of 'low risk' or 'high risk'
Other bias #2 ‐ Consistency in intervention delivery	Unclear risk	Quote: "When participants arrived, they received the M&Ms and were told that they could eat as much as they wanted during the experimental session but that they would not be allowed to remove the food from the room after the session... At the end of the session, the participants were instructed to place any and all remaining food and food packages in an envelope...". Comment: information and instructions provided to participants appear to have been standardised between the compared study conditions. No information pertaining to monitoring of participants' compliance with the instructions that they would not be allowed to remove the food from the room after the session and to place any and all remaining food and food packages in an envelope is reported. No further specific instructions were provided to participants, other than the instruction that they could eat as much as they wanted during the experimental session
Summary of risk of bias Consumption outcome	Unclear risk	Unclear risk

**Table:** Scott 2008c (S3)

Methods	Study design: between‐subjects randomised controlled trial	
Participants	Setting: laboratory setting Geographical region: Arizona, USA Number of enrolled participants: 96 adults Number (%) of enrolled participants completing the study: 96 (100%) Study completers ‐ mean age (SD): not reported Study completers ‐ sex: not reported Study completers ‐ mean BMI kg/m^2^ (SD): not reported Specific social or cultural characteristics: yes. University students Socio‐economic status context: low deprivation Inclusion criteria: not stated Exclusion criteria: not stated	
Interventions	Manipulated product type: food Manipulation: package size Duration of exposure to intervention: ≤ 1 day Social setting: consuming alone Study arms: small food, small package (8 mini cookies equally distributed across 4 small bags (i.e. 2 cookies per bag); large food, large package (4 large cookies in one bag) Number of comparisons analysed: 1 Comparisons analysed: Comparison 1 = Intervention 1: 8 mini cookies in 4 small bags (2 per bag); *versus* Intervention 2: 4 large cookies in one bag Concurrent intervention components: yes. Concurrent individual unit size manipulation	
Outcomes	Outcomes reported in study: energy intake from cookies; binary variable of consuming all the food presented or not Selection outcome analysed: N/A Measurement of selection outcome: N/A Timing of selection outcome measurement: N/A Consumption outcome analysed: energy intake from cookies (kcal) Measurement of consumption outcome: objective Timing of consumption outcome measurement: immediate (≤ 1 day)	
Funding source	Association for Consumer Research	
Notes	Study authors contacted for missing data but data no longer available Package size manipulation confounded with individual unit size manipulation and therefore coded as package size manipulation	
***Risk of bias***		
**Bias**	**Authors' judgement**	**Support for judgement**
Random sequence generation (selection bias)	Unclear risk	Comment: method of sequence generation is not described. Author contact confirmed group assignment was randomised but no further details relating to method of sequence generation for assignment to package/unit size groups (13 March 2013). Insufficient information about the sequence generation process to permit judgement of 'low risk' or 'high risk'
Allocation concealment (selection bias)	Unclear risk	Comment: method of concealment is not described. Author contact confirmed group assignment was randomised but no further details relating to method of sequence generation for assignment to package/unit size groups (13 March 2013). Insufficient information to permit judgement of 'low risk' or 'high risk'
Blinding of participants and personnel (performance bias) Consumption outcome	Low risk	Comment: blinding of study participants attempted and unlikely that the blinding could have been broken. Very unlikely that key study personnel were blinded, but the review authors judge that the outcome is not likely to be influenced by lack of blinding of key study personnel
Blinding of outcome assessment (detection bias) Consumption outcome	Low risk	Comment: no blinding of outcome assessment, but the review authors judge that the outcome measurement is not likely to be influenced by lack of blinding
Incomplete outcome data (attrition bias) Consumption outcome	Low risk	Comment: no missing outcome data for consumption outcome
Selective reporting (reporting bias)	Unclear risk	Comment: search for record(s) containing details of study protocol conducted in ClinicalTrials.gov and the WHO International Clinical Trials Registry Platform (ICTRP). No records found. Insufficient information to permit judgement of 'low risk' or 'high risk'
Other bias #1 ‐ Baseline comparability of participant characteristics between groups	Unclear risk	Comment: study uses a between‐subjects design. Insufficient information to permit judgement of 'low risk' or 'high risk'
Other bias #2 ‐ Consistency in intervention delivery	Unclear risk	Comment: insufficient information to permit judgement of 'low risk' or 'high risk'
Summary of risk of bias Consumption outcome	Unclear risk	Unclear risk

**Table:** Scott 2008d (S4)

Methods	Study design: between‐subjects randomised controlled trial	
Participants	Setting: laboratory setting Geographical region: Arizona, USA Number of enrolled participants: 393 adults Number (%) of enrolled participants completing the study: 393 (100%) Study completers ‐ mean age (SD): not reported Study completers ‐ sex: not reported Study completers ‐ mean BMI kg/m^2^ (SD): not reported Specific social or cultural characteristics: yes. University students Socio‐economic status context: low deprivation Inclusion criteria: not stated Exclusion criteria: not stated	
Interventions	Manipulated product type: food Manipulation: package size; individual unit size Duration of exposure to intervention: ≤ 1 day Social setting: consuming alone Study arms: small food, small packages, manipulated control system focus (200 calories of mini‐M&Ms evenly distributed across 4 small bags); small food, small packages, manipulated cool system focus (200 calories of mini‐M&Ms evenly distributed across 4 small bags ; small food, small packages, manipulated hot system focus (200 calories of mini‐M&Ms evenly distributed across 4 small bags; large food, large package, manipulated control system focus (200 calories of regular M&Ms in one large bag); large food, large package, manipulated cool system focus (200 calories of regular M&Ms in one large bag); large food, large package, manipulated hot system focus (200 calories of regular M&Ms in one large bag) Number of comparisons analysed: 1 Comparisons analysed: comparison 1 = Intervention 1: mini‐M&Ms in 4 small bags; *versus* Intervention 2: regular M&Ms in one large bag Concurrent intervention components: yes. Concurrent individual unit size manipulation. System focus manipulation (hot, cool, control) via thinking and writing task	
Outcomes	Outcomes reported in study: energy intake from M&Ms; binary variable of consuming all the food presented or not Selection outcome analysed: N/A Measurement of selection outcome: N/A Timing of selection outcome measurement: N/A Consumption outcome analysed: energy intake from M&Ms (kcal) Measurement of consumption outcome: objective Timing of consumption outcome measurement: immediate (≤ 1 day)	
Funding source	Association for Consumer Research	
Notes	Study authors contacted for missing data but data no longer available Package size manipulation confounded with individual unit size manipulation and therefore coded as package size manipulation	
***Risk of bias***		
**Bias**	**Authors' judgement**	**Support for judgement**
Random sequence generation (selection bias)	Unclear risk	Comment: method of sequence generation is not described. Author contact confirmed group assignment was randomised but no further details relating to method of sequence generation for assignment to package/unit size groups (13 March 2013). Insufficient information about the sequence generation process to permit judgement of 'low risk' or 'high risk'
Allocation concealment (selection bias)	Unclear risk	Comment: method of concealment is not described. Author contact confirmed group assignment was randomised but no further details relating to method of sequence generation for assignment to package/unit size groups (13 March 2013). Insufficient information to permit judgement of 'low risk' or 'high risk'
Blinding of participants and personnel (performance bias) Consumption outcome	Low risk	Comment: blinding of study participants attempted and unlikely that the blinding could have been broken. Very unlikely that key study personnel were blinded, but the review authors judge that the outcome is not likely to be influenced by lack of blinding of key study personnel
Blinding of outcome assessment (detection bias) Consumption outcome	Low risk	Comment: the review authors assumed that consumption quantity is measured using the same procedure as in the other 2 included studies reported in the same article. No blinding of outcome assessment, but the review authors judge that the outcome measurement is not likely to be influenced by lack of blinding
Incomplete outcome data (attrition bias) Consumption outcome	Low risk	Comment: no missing outcome data for consumption outcome
Selective reporting (reporting bias)	Unclear risk	Comment: search for record(s) containing details of study protocol conducted in ClinicalTrials.gov and the WHO International Clinical Trials Registry Platform (ICTRP). No records found. Insufficient information to permit judgement of 'low risk' or 'high risk'
Other bias #1 ‐ Baseline comparability of participant characteristics between groups	Unclear risk	Comment: study uses a between‐subjects design. Insufficient information to permit judgement of 'low risk' or 'high risk'
Other bias #2 ‐ Consistency in intervention delivery	Unclear risk	Comment: insufficient information to permit judgement of 'low risk' or 'high risk'
Summary of risk of bias Consumption outcome	Unclear risk	Unclear risk

**Table:** Shah 2011

Methods	Study design: within‐subjects randomised controlled trial	
Participants	Setting: laboratory setting Geographical region: Texas, USA Number of enrolled participants: 20 adults Number (%) of enrolled participants completing the study: 20 (100%) Study completers ‐ mean age (SD): 40.6 (16.1) Study completers ‐ sex: 100% female Study completers ‐ mean BMI kg/m^2^ (SD): 26.7 (5.9) Specific social or cultural characteristics: yes. University community Socio‐economic status context: low deprivation Inclusion criteria: normal weight, overweight and obese women Exclusion criteria: current dieting; BMI ≥ 40; self reported eating disorders; taking medications that affect appetite; participation in vigorous physical activity; smoking	
Interventions	Manipulated product type: food Manipulation: tableware size Duration of exposure to intervention: ≤ 1 day Social setting: consuming alone Study arms: food self served on to a small diameter plate (diameter 21.6 cm); food self served on to a large diameter plate (diameter 27.4 cm) Number of comparisons analysed: 1 Comparisons analysed: Comparison 1 = Intervention 1: small plate (diameter 21.6 cm); *versus* Intervention 2: large plate (diameter 27.4 cm) Concurrent intervention components: no	
Outcomes	Outcomes reported in study: energy intake from total lunch meal (kilojoules) Selection outcome analysed: N/A Measurement of selection outcome: N/A Timing of selection outcome measurement: N/A Consumption outcome analysed: energy intake from total lunch meal (kilojoules) Measurement of consumption outcome: objective Timing of consumption outcome measurement: immediate (≤ 1 day)	
Funding source	Texas Christian University	
Notes	—	
***Risk of bias***		
**Bias**	**Authors' judgement**	**Support for judgement**
Random sequence generation (selection bias)	Unclear risk	Comment: method of sequence generation for condition order is not described. Insufficient information about the sequence generation process to permit judgement of 'low risk' or 'high risk'
Allocation concealment (selection bias)	Unclear risk	Comment: method of concealment is not described. Insufficient information to permit judgement of 'low risk' or 'high risk'
Blinding of participants and personnel (performance bias) Consumption outcome	Unclear risk	Quote: "The subjects were blinded to the study objective... Another concern is that the subject may have guessed the objective of the study because the study was conducted in a laboratory setting. This is unlikely to have occurred, however, because questioning the subjects after the study completion did not reveal any awareness of the study objective." Comment: no blinding or incomplete blinding. Participants were probed for suspicion of study purpose but not for awareness of size manipulation between study conditions. It is possible that blinding of study participants was broken in some cases and that the outcome may be influenced by lack of blinding (due to potential carry‐over effects between conditions). Very unlikely that key study personnel were blinded, but the review authors judge that the outcome is not likely to be influenced by lack of blinding of key study personnel
Blinding of outcome assessment (detection bias) Consumption outcome	Low risk	Comment: no blinding of outcome assessment, but the review authors judge that the outcome measurement is not likely to be influenced by lack of blinding
Incomplete outcome data (attrition bias) Consumption outcome	Low risk	Comment: no missing outcome data for consumption outcome
Selective reporting (reporting bias)	Unclear risk	Comment: search for record(s) containing details of study protocol conducted in ClinicalTrials.gov and the WHO International Clinical Trials Registry Platform (ICTRP). No records found. Insufficient information to permit judgement of 'low risk' or 'high risk'
Other bias #1 ‐ Baseline comparability of participant characteristics between groups	Unclear risk	Quote: "Immediately before...each meal, feelings of hunger, satiety, fullness and prospective consumption (i.e. how much one can eat) were assessed using a 100‐mm visual analogue scale..." Comment: not reported whether there were differences between condition orders in terms of measured baseline participant 'state' characteristics. No analysis of potential differences in measured outcomes between condition orders appears to have been conducted and the statistical analysis of outcome data does not appear to control for condition order. Risk of bias due to period effects is therefore unclear. Insufficient information to permit judgement of 'low risk' or 'high risk'
Other bias #2 ‐ Consistency in intervention delivery	Unclear risk	Quote: "Subjects were asked to consume the same food and drink and engage in the same level of physical activity the day before the study days...Subjects were also asked to eat the same breakfast on the two study days. No food or drink other than water was allowed between breakfast and lunch and no water was allowed for 1 h before lunch. Each subject was interviewed before lunch to ensure that the above requirements were met... Subjects were asked to drink 237 g of water when consuming the [test] meal." Comment: information and instructions provided to participants appear to have been standardised between the compared study conditions. Participants' compliance with the instructions to consume the same food and drink and engage in the same level of physical activity the day before the study days, to eat the same breakfast on the 2 study days, not to consume any food or drink between breakfast and lunch and not to consume water for 1 hour before lunch was monitored via verbal self report at interview; however no monitoring results are reported with respect to these instructions. No information pertaining to monitoring of participants' compliance with the instruction to drink 237 g of water when consuming the test meal is reported. No further specific instructions were provided to participants
Summary of risk of bias Consumption outcome	Unclear risk	Unclear risk

**Table:** Spill 2010

Methods	Study design: within‐subjects cluster‐randomised controlled trial Unit of allocation: classroom Unit of analysis: individual Number of clusters: 5 Number of participants per cluster: not reported Analysis does not appear to account for cluster allocation, as the classroom variable was not used to determine main effects and interactions	
Participants	Setting: field setting, daycare centre Geographical region: Pennsylvania, USA Number of enrolled participants: 51 children Number (%) of enrolled participants completing the study: 51 (100%) Study completers ‐ mean age (SD): 4.4 (0.7) Study completers ‐ sex: 56.9% female Study completers ‐ mean BMI kg/m^2^ (SD): not reported (BMI z score and BMI percentile are reported) Specific social or cultural characteristics: yes. Children enrolled in daycare centre of Pennsylvania State University Socio‐economic status context: low deprivation Inclusion criteria: preschool‐aged children enrolled in daycare at the Bennett Family Center at Pennsylvania State University. Exclusion criteria: not stated	
Interventions	Manipulated product type: food Manipulation: portion size Duration of exposure to intervention: ≤ 1 day Social setting: consuming with others Study arms: 30 g portion size of carrots in first course; 60 g portion size of carrots in first course; 90 g portion size of carrots in first course; no carrots given in first course (latter excluded from this analysis) Number of comparisons analysed: 2 Comparisons analysed: Comparison 1: Intervention 1: 30 g portion size of carrots served in the first course; *versus* Intervention 2: 60 g portion size of carrots served in the first course Comparison 2: Intervention 1: 60 g portion size of carrots served in the first course; *versus* Intervention 2: 90 g portion size of carrots served in the first course Concurrent intervention components: no	
Outcomes	Outcomes reported in study: total meal intake energy consumption (kcal); total meal intake (g); intake of carrots (kcal); intake of carrots (g); intake of other non‐manipulated meal components (kcal and g) Selection outcome analysed: N/A Measurement of selection outcome: N/A Timing of selection outcome measurement: N/A Consumption outcome analysed: energy intake from total lunch meal (kcal) Measurement of consumption outcome: objective Timing of consumption outcome measurement: immediate (≤ 1 day)	
Funding source	National Institute of Diabetes and Digestive and Kidney Diseases, and the Robert Wood Johnson Foundation	
Notes	Study authors contacted for missing data with additional data received March 2014	
***Risk of bias***		
**Bias**	**Authors' judgement**	**Support for judgement**
Random sequence generation (selection bias)	Low risk	Quote: "The experimental conditions across study weeks was assigned to classrooms by using a Latin square design."
Allocation concealment (selection bias)	Unclear risk	Comment: participating classrooms appear to have been randomised to condition order concurrently. However, it is unclear whether randomised to condition order occurred before or after consent for individuals' participation had been obtained. The review authors therefore judge that there is insufficient information to permit judgement of 'low risk' or 'high risk'
Blinding of participants and personnel (performance bias) Consumption outcome	Unclear risk	Comment: no blinding or incomplete blinding. Participants were not probed for suspicion of study purpose or awareness of size manipulation between study conditions. It is possible that blinding of study participants was broken in some cases and that the outcome may be influenced by lack of blinding (due to potential carry‐over effects between conditions). Very unlikely that key study personnel were blinded, but the review authors judge that the outcome is not likely to be influenced by lack of blinding of key study personnel
Blinding of outcome assessment (detection bias) Consumption outcome	Low risk	Comment: no blinding of outcome assessment, but the review authors judge that the outcome measurement is not likely to be influenced by lack of blinding
Incomplete outcome data (attrition bias) Consumption outcome	Low risk	Comment: no missing outcome data for consumption outcome
Selective reporting (reporting bias)	Unclear risk	Comment: search for record(s) containing details of study protocol conducted in ClinicalTrials.gov and the WHO International Clinical Trials Registry Platform (ICTRP). No records found. Insufficient information to permit judgement of 'low risk' or 'high risk'
Other bias #1 ‐ Baseline comparability of participant characteristics between groups	Unclear risk	Comment: study uses a within‐subjects design. No measurement of participant pre‐condition 'state' characteristics is reported. No analysis of potential differences in measured outcomes between condition orders appears to have been conducted and the statistical analysis of outcome data does not appear to control for condition order. Risk of bias due to period effects is therefore unclear. Insufficient information to permit judgement of 'low risk' or 'high risk'
Other bias #2 ‐ Consistency in intervention delivery	Unclear risk	Quote: "Teachers were instructed to redirect conversations pertaining to food to nonfood‐related topics to minimize the influence on lunch intake." Comment: information and instructions provided to participants appear to have been standardised between the compared study conditions. No information pertaining to monitoring of teachers' compliance with the instruction to redirect conversations pertaining to food to nonfood‐related topics is reported. No further specific instructions were provided to participants or providers
Summary of risk of bias Consumption outcome	Unclear risk	Unclear risk

**Table:** Spill 2011b

Methods	Study design: within‐subjects cluster‐randomised controlled trial Unit of allocation: classroom Unit of analysis: individual Number of clusters: 5 Number of participants per cluster: not reported Analysis appears to account for cluster allocation, as the statistical model accounted for between‐subjects variation in classroom and the classroom variable was used to determine main effects and interactions	
Participants	Setting: field setting, daycare centre Geographical region: Pennsylvania, USA Number of enrolled participants: 73 children Number (%) of enrolled participants completing the study: 72 (98.6%) Study completers ‐ mean age (SD): 4.7 (0.8) Study completers ‐ sex: 56.9% female Study completers ‐ mean BMI kg/m^2^ (SD): not reported (BMI percentile is reported) Specific social or cultural characteristics: yes. Children enrolled in daycare centre of Pennsylvania State University. Socio‐economic status context: low deprivation Inclusion criteria: children aged 3 to 6 years enrolled in daycare centres at the Pennsylvania State University Exclusion criteria: not stated	
Interventions	Manipulated product type: food Manipulation: portion size Duration of exposure to intervention: ≤ 1 day Social setting: consuming with others Study arms: 150 g portion size of tomato soup in first course of lunch; 225 g portion size of tomato soup in first course of lunch; 300 g portion size of tomato soup in first course of lunch; no soup given in first course of lunch (latter study arm excluded from this analysis) Number of comparisons analysed: 2 Comparisons analysed: Comparison 1: Intervention 1: 150 g portion of tomato soup; *versus* Intervention 2: 225 g portion of tomato soup Comparison 2: Intervention 1: 225 g portion of tomato soup; *versus* Intervention 2: 300 g portion of tomato soup Concurrent intervention components: no	
Outcomes	Outcomes reported in study: total lunch meal intake energy consumption (kcal); total lunch meal intake (g); intake of soup (kcal); intake of soup (g); intake of other non‐manipulated meal components (kcal and g) Selection outcome analysed: N/A Measurement of selection outcome: N/A Timing of selection outcome measurement: N/A Consumption outcome analysed: energy intake from total lunch meal (kcal) Measurement of consumption outcome: objective. Timing of consumption outcome measurement: immediate (≤ 1 day)	
Funding source	Not reported	
Notes	Study authors contacted for missing data with additional data received March 2014	
***Risk of bias***		
**Bias**	**Authors' judgement**	**Support for judgement**
Random sequence generation (selection bias)	Unclear risk	Comment: method of sequence generation for condition order is not described. Author contact confirmed condition order was randomised but no further details (13 March 13). Insufficient information about the sequence generation process to permit judgement of 'low risk' or 'high risk'
Allocation concealment (selection bias)	Unclear risk	Comment: participating classrooms appear to have been randomised to condition order concurrently. However, it is unclear whether randomised to condition order occurred before or after consent for individuals' participation had been obtained. The review authors therefore judge that there is insufficient information to permit judgement of 'low risk' or 'high risk'
Blinding of participants and personnel (performance bias) Consumption outcome	Unclear risk	Comment: no blinding or incomplete blinding. Participants were not probed for suspicion of study purpose or awareness of size manipulation between study conditions. It is possible that blinding of study participants was broken in some cases and that the outcome may be influenced by lack of blinding (due to potential carry‐over effects between conditions). Very unlikely that key study personnel were blinded, but the review authors judge that the outcome is not likely to be influenced by lack of blinding of key study personnel
Blinding of outcome assessment (detection bias) Consumption outcome	Low risk	Comment: no blinding of outcome assessment, but the review authors judge that the outcome measurement is not likely to be influenced by lack of blinding
Incomplete outcome data (attrition bias) Consumption outcome	Low risk	Quote: "A total of 73 children from five classrooms were recruited. Data from one child was identified as having an undue influence on the results because of high variability across meals, and the data was therefore excluded from the analysis." Comment: the reason for missing outcome data likely to be related to outcome. The low proportion (one participant, 1% of study sample) of exclusions means that the review authors judge that the plausible effect size among missing outcomes is unlikely to be enough to have an important impact on the observed effect size
Selective reporting (reporting bias)	Unclear risk	Comment: search for record(s) containing details of study protocol conducted in ClinicalTrials.gov and the WHO International Clinical Trials Registry Platform (ICTRP). No records found. Insufficient information to permit judgement of 'low risk' or 'high risk'
Other bias #1 ‐ Baseline comparability of participant characteristics between groups	Unclear risk	Comment: study uses a within‐subjects design. No measurement of participant pre‐condition 'state' characteristics is reported. No analysis of potential differences in measured outcomes between condition orders appears to have been conducted and the statistical analysis of outcome data does not appear to control for condition order. Risk of bias due to period effects is therefore unclear. Insufficient information to permit judgement of 'low risk' or 'high risk'
Other bias #2 ‐ Consistency in intervention delivery	Unclear risk	Quote: "Teachers were instructed to redirect conversations pertaining to food to other topics to minimize the influence on lunch intake." Comment: information and instructions provided to participants appear to have been standardised between the compared study conditions. No information pertaining to monitoring of teachers' compliance with the instruction to redirect conversations pertaining to food to other topics is reported. No further specific instructions were provided to participants or providers
Summary of risk of bias Consumption outcome	Unclear risk	Unclear risk

**Table:** Stroebele 2009

Methods	Study design: within‐subjects randomised controlled trial	
Participants	Setting: field setting. Community Geographical region: Colorado, USA Number of enrolled participants: 63 adults Number (%) of enrolled participants completing the study: 59 (93.7%) Study completers ‐ mean age (SD): 37.3 (12) Study completers ‐ sex: 69.5% female Study completers ‐ mean BMI kg/m^2^ (SD): 27.7 (3.9) Specific social or cultural characteristics: yes. University community Socio‐economic status context: low deprivation Inclusion criteria: between ages of 18 and 65 years; BMI between 23 and 40; frequent snacker (2+ snacks per day); living in a 1 to 2 person household (to reduce the likelihood of other individuals eating the provided food); currently taking no weight loss medications; no history of binge eating; being non‐diabetic and not pregnant or breastfeeding Exclusion criteria: not stated	
Interventions	Manipulated product type: food Manipulation: package size Duration of exposure to intervention: > 1 day Social setting: consuming alone and with others Study arms: small portion‐controlled 100 kcal packages of various snacks; large standard size packages of various snacks Number of comparisons analysed: 1 Comparisons analysed: Comparison 1: Intervention 1: small portion‐controlled 100 kcal packages of various snacks; *versus* Intervention 2: large standard size packages of various snacks Concurrent intervention components: no	
Outcomes	Outcomes reported in study: amount of allocated snack foods consumed over week (grams) Selection outcome analysed: N/A Measurement of selection outcome: N/A Timing of selection outcome measurement: N/A Consumption outcome analysed: amount of allocated snack foods consumed over week (grams) Measurement of consumption outcome: objective Timing of consumption outcome measurement: longer‐term (> 1 day)	
Funding source	United States National Institutes of Health. Foods provided by Kraft Foods and Frito‐Lay	
Notes	—	
***Risk of bias***		
**Bias**	**Authors' judgement**	**Support for judgement**
Random sequence generation (selection bias)	Unclear risk	Comment: method of sequence generation for condition order is not described. Insufficient information about the sequence generation process to permit judgement of 'low risk' or 'high risk'
Allocation concealment (selection bias)	Unclear risk	Comment: method of concealment is not described. Insufficient information to permit judgement of 'low risk' or 'high risk'
Blinding of participants and personnel (performance bias) Consumption outcome	Unclear risk	Quote: "Participants were men and women between the ages of 18 and 65 years recruited through an email distributed through the University of Colorado Denver to participate in a study investigating the differences in snack foods and food packaging on eating behavior in adults... [We] found that the order and week in which the packages were received also played a role in energy consumption. Receiving the 100 kcal snack packs first seemed to reduce the amount eaten from standard size packages later, suggesting that the portion‐controlled packages may increase awareness of portion size that lasted when the larger packages were available." Comment: no blinding or incomplete blinding. Participants were not probed for suspicion of study purpose or awareness of size manipulation between study conditions. It is likely that blinding of study participants was broken in some cases and possible that the outcome may be influenced by lack of blinding (due to potential carry‐over effects between conditions). Very unlikely that key study personnel were blinded, but the review authors judge that the outcome is not likely to be influenced by lack of blinding of key study personnel
Blinding of outcome assessment (detection bias) Consumption outcome	Unclear risk	Quote: "For both visits, participants were asked to record the amount of snacks remaining after each week. For the 100 kcal packages, they were asked to count the number of pouches left. For the standard size packages, participants were asked to count the remaining unopened snack bags and to return those bags that were opened. The opened bags were weighed by the research personnel These measures were used to assess the reliability of the snacking diaries." Comment: insufficient information to permit judgement of 'low risk' or 'high risk'
Incomplete outcome data (attrition bias) Consumption outcome	Low risk	Quote: "A total of 63 participants enrolled in the study, but 3 participants did not return after the first 7‐day period and one participant recorded both periods inaccurately. Therefore, 59 participants, 41 women and 18 men, completed the study." Comment: reasons for missing outcome data are unlikely to be related to consumption outcome
Selective reporting (reporting bias)	Unclear risk	Comment: search for record(s) containing details of study protocol conducted in ClinicalTrials.gov and the WHO International Clinical Trials Registry Platform (ICTRP). No records found. Insufficient information to permit judgement of 'low risk' or 'high risk'
Other bias #1 ‐ Baseline comparability of participant characteristics between groups	Low risk	Quote: "Repeated measures mixed models were used to analyse the data...[with] package size X study week interaction as [a fixed factor]...Estimate statements were used to ...perform post hoc tests for the package size X randomization order interaction... Post hoc comparisons revealed the effect of package size depended on...randomization order... Specifically, participants receiving standard size packages of snacks during week 2 (who had previously consumed 100 kcal snack packs) consumed an average of only 486.7 g of snacks from the standard size packages, compared to the 675.7 g of snacks consumed by the other randomization group when they received the standard size packages in week 1. Additionally, participants who received the standard size packages during week 1 ate significantly less when switching to the 100 kcal snack packs...There was no significant difference between the two randomization groups in the amounts consumed from the 100 kcal snack packs." Comment: study uses a within‐subjects design. No measurement of participant pre‐condition 'state' characteristics is reported. Whilst the study authors report differences in consumption outcome between condition orders, these differences appear to be controlled for in the statistical analysis of outcome data, as this appears to control for condition order. It is therefore unlikely that any differences between condition orders in terms of measured pre‐condition participant 'state' characteristics influenced the measured outcomes. Risk of bias due to period effects is therefore judged low
Other bias #2 ‐ Consistency in intervention delivery	Unclear risk	Quote: "...[Participants] were trained in using the 7‐day snacking diary. Participants were asked to record each snack occasion including the brand and amount of snack chosen, the consumption location, the time of day, whether the television was on or off, and the presence of other people. During the 100 kcal snack package week, participants were asked to simply record the number of 100 kcal pouches they were eating on each eating occasion. During the standard size package unit week, participants were provided with a digital food scale...and were asked to measure each food bag before and after consumption. Furthermore, participants were instructed to maintain their regular eating habits even if this would lead to days when no snacks were consumed to reflect real life conditions as accurate as possible....They were also instructed to not share their snacks with anyone else during the study period... At the second visit, participants were asked to return the snacking diary and the same food brands chosen during the first visit were provided in the other packaging size. Participants were asked not to eat any snack foods out of the previously provided boxes during the second week of recording...The same instructions about consumption and sharing were given. After recording their snacks again for 7 days, participants returned one last time to the research facility... For both visits, participants were asked to record the amount of snacks remaining after each week. For the 100 kcal packages, they were asked to count the number of pouches left. For the standard size packages, participants were asked to count the remaining unopened snack bags and to return those bags that were opened. The opened bags were weighed by the research personnel These measures were used to assess the reliability of the snacking diaries. The correlation between weights taken by the research personnel and intake derived from the food diaries was high (0.88 for standard size packages and 0.80 for 100 kcal packages)...[One] participant recorded both periods inaccurately [and was therefore excluded from the study]." Comment: information and instructions provided to participants appear to have been standardised between the compared study conditions. Participants' compliance with the instruction to record each snack occasion including the brand, the amount of snack chosen and the amount of snack consumed and remaining after each study week was monitored by comparison between weights of food measured by the research personnel at the end of each study week and intake derived from the food diaries. It is reported that the correlation between weights of food measured by the research personnel at the end of each study week and intake derived from the food diaries was high and also that one participant was excluded due to evidence of inaccuracy in their recording derived by this monitoring process. No information pertaining to monitoring of participants' compliance with the instructions to maintain their regular eating habits and to not share their snacks with anyone else during the study period is reported. No further specific instructions were provided to participants
Summary of risk of bias Consumption outcome	Unclear risk	Unclear risk

**Table:** van Kleef 2012

Methods	Study design: between‐subjects randomised controlled trial	
Participants	Setting: laboratory setting Geographical region: New York, USA Number of enrolled participants: 68 adults Number (%) of enrolled participants completing the study: 67 (98.5%) Study completers ‐ mean age (SD): 20.5 (2.4) Study completers ‐ sex: 47.6% female Study completers ‐ mean BMI kg/m^2^ (SD): 24.2 (4) Specific social or cultural characteristics: yes. University undergraduates Socio‐economic status context: low deprivation Inclusion criteria: not stated Exclusion criteria: not stated	
Interventions	Manipulated product type: food Manipulation: tableware size Duration of exposure to intervention: ≤ 1 day Social setting: selecting and consuming with others Study arms: serving self from 3.8 L capacity bowl, containing approximately 2000 g of pasta dish; serving self from 6.9 L capacity bowl, containing approximately 2000 g of pasta dish Number of comparisons analysed: 1 Comparisons analysed: Comparison 1: Intervention 1: serving self from 3.8 L bowl; *versus* Intervention 2: serving self from 6.9 L bowl Concurrent intervention components: no	
Outcomes	Outcomes reported in study: log transformed pasta served (grams); log transformed pasta consumed (kcal) Selection outcome analysed: log transformed pasta served (grams) Measurement of selection outcome: objective Timing of selection outcome measurement: immediate (≤ 1 day) Consumption outcome analysed: log transformed pasta consumed (kcal) Measurement of consumption outcome: objective Timing of consumption outcome measurement: immediate (≤ 1 day)	
Funding source	Marie Curie International Outgoing Fellowship within the 7^th^ European Community Framework Programme	
Notes	—	
***Risk of bias***		
**Bias**	**Authors' judgement**	**Support for judgement**
Random sequence generation (selection bias)	Unclear risk	Comment: method of sequence generation is not described. Insufficient information about the sequence generation process to permit judgement of 'low risk' or 'high risk'
Allocation concealment (selection bias)	Unclear risk	Comment: method of concealment is not described. Insufficient information to permit judgement of 'low risk' or 'high risk'
Blinding of participants and personnel (performance bias) Selection outcome	Low risk	Quote: "To prevent carryover effects and awareness of the study objective among participants, we chose a between‐subjects design instead of a within‐subjects design... Because participants in each experimental session were in only 1 of the 2 conditions, they were not biased by being able to observe the self‐serving of the food in the other condition." Comment: no blinding or incomplete blinding of study participants and key study personnel, but the review authors judge that the outcome is not likely to be influenced by lack of blinding
Blinding of participants and personnel (performance bias) Consumption outcome	Low risk	Quote: "To prevent carryover effects and awareness of the study objective among participants, we chose a between‐subjects design instead of a within‐subjects design... Because participants in each experimental session were in only 1 of the 2 conditions, they were not biased by being able to observe the self‐serving of the food in the other condition." Comment: blinding of study participants attempted and unlikely that the blinding could have been broken. Very unlikely that key study personnel were blinded, but the review authors judge that the outcome is not likely to be influenced by lack of blinding of key study personnel
Blinding of outcome assessment (detection bias) Selection outcome	Low risk	Comment: no blinding of outcome assessment, but the review authors judge that the outcome measurement is not likely to be influenced by lack of blinding
Blinding of outcome assessment (detection bias) Consumption outcome	Low risk	Comment: no blinding of outcome assessment, but the review authors judge that the outcome measurement is not likely to be influenced by lack of blinding
Incomplete outcome data (attrition bias) Selection outcome	Low risk	Quote: "...one outlier...was excluded because this participant deviated at least 3 SDs from the mean pasta consumption in her condition, leaving 67 participants in the dataset (32 women)." Comment: the reason for missing outcome data for selection outcome is the study authors' decision to exclude outliers (at least 3 SDs from mean consumption) from the analysis. The low proportion (1 participant, 1% of study sample) of exclusions due to outliers means that the review authors judge that the plausible effect size among missing outcomes is unlikely to be enough to have an important impact on the observed effect size
Incomplete outcome data (attrition bias) Consumption outcome	Low risk	Quote: "...one outlier...was excluded because this participant deviated at least 3 SDs from the mean pasta consumption in her condition, leaving 67 participants in the dataset (32 women)." Comment: the reason for missing outcome data for consumption outcome is the study authors' decision to exclude outliers (at least 3 SDs from mean consumption) from the analysis. The low proportion (1 participant, 1% of study sample) of exclusions due to outliers means that the review authors judge that the plausible effect size among missing outcomes is unlikely to be enough to have an important impact on the observed effect size
Selective reporting (reporting bias)	Unclear risk	Comment: search for record(s) containing details of study protocol conducted in ClinicalTrials.gov and the WHO International Clinical Trials Registry Platform (ICTRP). No records found. Insufficient information to permit judgement of 'low risk' or 'high risk'
Other bias #1 ‐ Baseline comparability of participant characteristics between groups	Low risk	Quote: "There were no significant differences in the time since participant received food most recently between the 2 conditions. However, there were trends toward a sex difference in BMI, and therefore in the analysis we included BMI as covariate to control for influence." Comment: study uses a between‐subjects design. Difference between comparison groups in terms of BMI. The statistical analysis of outcome data controls for this difference. No evidence of differences between comparison groups in terms of other measured baseline participant characteristics
Other bias #2 ‐ Consistency in intervention delivery	High risk	Quote: "The procedure followed was identical for both conditions, except that the bowl was at another place in the room. More specifically, in the condition of the large bowl, participants formed a line to serve themselves food from the bowl placed in front of the blackboard. This position was chosen because it was the most convenient and natural place for serving oneself out of a bowl containing a rather large amount of food. In the condition of the medium‐sized bowl, participants were instructed to serve themselves from the bowl placed in their station (bowls were placed in 8 kitchen stations). Placing them together in the same area in front of the blackboard (as in the large‐bowl condition) might have made the real purpose of the study apparent to participants... In both conditions, participants could serve themselves as much as they wanted and second servings were allowed." Comment: information provided to participants appears to have been standardised between the compared study conditions. Instructions provided to participants differed between the compared study conditions as described in the quote above. The rationale for providing instructions that differed between the compared study conditions was to attempt to preserve blinding of participants to the true study purpose and to the difference in bowl size between the compared study conditions. The review authors judge that it is feasible that measured selection and consumption outcomes may have been influenced by differences in instructions provided to participants in the 2 respective study conditions due to the potential moderating influence of the resulting difference in proximity of the respective serving bowls to the stations at which participants consumed the test meal. No further instructions were provided to participants, other than the instructions that participants could serve themselves as much as they wanted and second servings were allowed
Summary of risk of bias Selection outcome	Unclear risk	Unclear risk
Summary of risk of bias Consumption outcome	Unclear risk	Unclear risk

**Table:** van Kleef 2013

Methods	Study design: between‐subjects randomised controlled trial	
Participants	Setting: laboratory setting Geographical region: New York, USA Number of enrolled participants: 105 adults Number (%) of enrolled participants completing the study: 104 (99.1%) Study completers ‐ mean age (SD): 19.5 (3.1) Study completers ‐ sex: 49% female Study completers ‐ mean BMI kg/m^2^ (SD): 22.6 (1.8) Specific social or cultural characteristics: yes. University undergraduates Socio‐economic status context: low deprivation Inclusion criteria: not stated Exclusion criteria: not stated	
Interventions	Manipulated product type: food Manipulation: portion size Duration of exposure to intervention: ≤ 1 day Social setting: consuming with others Study arms: small portion condition containing 10 g of chocolate chips, 40 g of apple pie, and 10 g of potato chips (total calories = 195 calories); large portion condition containing 100 g of chocolate chips, 200 g of apple pie and 80 g of potato chips (total calories = 1370 calories) Number of comparisons analysed: 1 Comparisons analysed: Comparison 1: Intervention 1: 10 g of chocolate chips; 40 g of apple pie; 10 g of potato chips (total food = 60 g; total calories = 195 calories); *versus* Intervention 2: 100 g of chocolate chips; 200 g of apple pie; 80 g of potato chips (total food = 380g of total food; total calories = 1370 calories) Concurrent intervention components: no	
Outcomes	Outcomes reported in study: total energy intake (kcal); total food consumed (grams); chocolate consumed (grams); apple pie consumed (grams); potato chips consumed (grams) Selection outcome analysed: N/A Measurement of selection outcome: N/A Timing of selection outcome measurement: N/A Consumption outcome analysed: total energy intake (kcal) Measurement of consumption outcome: objective Timing of consumption outcome measurement: immediate (≤ 1 day)	
Funding source	Not stated	
Notes	—	
***Risk of bias***		
**Bias**	**Authors' judgement**	**Support for judgement**
Random sequence generation (selection bias)	Unclear risk	Comment: method of sequence generation is not described. Insufficient information about the sequence generation process to permit judgement of 'low risk' or 'high risk'
Allocation concealment (selection bias)	Unclear risk	Comment: method of concealment is not described. Insufficient information to permit judgement of 'low risk' or 'high risk'
Blinding of participants and personnel (performance bias) Consumption outcome	Low risk	Quote: "To prevent carry‐over effects and awareness of the study's objectives among participants, we chose a between subjects design instead of a within subjects design...Four different experimental sessions of 25 to 29 mixed‐gender participants were conducted, with two sessions involving a small portion size condition and two sessions involving the large portion size condition." Comment: blinding of study participants attempted and unlikely that the blinding could have been broken. Very unlikely that key study personnel were blinded, but the review authors judge that the outcome is not likely to be influenced by lack of blinding of key study personnel
Blinding of outcome assessment (detection bias) Consumption outcome	Low risk	Comment: no blinding of outcome assessment, but the review authors judge that the outcome measurement is not likely to be influenced by lack of blinding
Incomplete outcome data (attrition bias) Consumption outcome	Low risk	Quote: "One participant was excluded from the data based on unknown gender." Comment: reason for missing outcome data is unlikely to be related to consumption outcome
Selective reporting (reporting bias)	Unclear risk	Comment: search for record(s) containing details of study protocol conducted in ClinicalTrials.gov and the WHO International Clinical Trials Registry Platform (ICTRP). No records found. Insufficient information to permit judgement of 'low risk' or 'high risk'
Other bias #1 ‐ Baseline comparability of participant characteristics between groups	Low risk	Quote: "Measures of overall hunger and craving were assessed just before participants started with the taste test... As a manipulation check, we also measured the appeal of the three foods, their familiarity to participants and their expectation on how quickly the food would bore them (7‐point scales)...We conducted a mixed model ANCOVA with measurement time as within subjects factor and condition and gender as between subjects factors to assess differences in hunger and craving between conditions and measurement time. To control for influence, BMI (mean‐centered) and session time (2 and 3 pm) were included in all models as covariates...The mean age of the participants was 19.5 years...with participants having a mean BMI of 22.6 kg/m2...Of all participants, 14 were overweight (BMI > 25). These participants were distributed evenly across both portion size conditions...There were no significant differences in mean restrained score of participants...and the time since participant had last food...across conditions. There were also no differences across conditions in the appeal of the three foods ...their familiarity...and expectations on how quickly the food would bore participants...The mixed model ANCOVA demonstrated a significant main effect of time of measurement..., but no main effect of portion size condition...or interaction between portion size condition and time of measurement on hunger ratings." Comment: study uses a between‐subjects design. No differences between comparison groups in terms of measured baseline participant characteristics
Other bias #2 ‐ Consistency in intervention delivery	Low risk	Quote: "Participants were instructed to eat as much or as little as desired to evaluate the foods on several dimensions (e.g. aftertaste) and take as much time as needed." Comment: information and instructions provided to participants appear to have been standardised between the compared study conditions. No specific instructions were provided to participants, other than the instructions to eat as much or as little of the test foods as desired and to take as much time as needed, and therefore participants' compliance with instructions is not applicable
Summary of risk of bias Consumption outcome	Unclear risk	Unclear risk

**Table:** Wansink 1996a (S1)

Methods	Study design: between‐subjects randomised controlled trial	
Participants	Setting: laboratory setting Geographical region: New Hampshire and Vermont, USA Number of enrolled participants: 98 adults Number (%) of enrolled participants completing the study: 98 (100%) Study completers ‐ mean age (SD): not reported Study completers ‐ sex: 100% female Study completers ‐ mean BMI kg/m^2^ (SD): not reported Specific social or cultural characteristics: yes. Adults recruited via parent‐teacher associations Socio‐economic status context: low deprivation Inclusion criteria: none reported Exclusion criteria: none reported	
Interventions	Manipulated product type: food Manipulation: package size Duration of exposure to intervention: ≤ 1 day Social setting: selecting alone Study arms: small package of the Creamette spaghetti strands product (same amount of product to select presented, so package full); large package of the Creamette spaghetti strands package twice the size (same amount of product to select presented, so package half‐full) Number of comparisons analysed: 1 Comparisons analysed: Comparison 1: Intervention 1: small package of the product; *versus* Intervention 2: large package twice the size Concurrent intervention components: no	
Outcomes	Outcomes reported in study: strands of spaghetti selected by placing in pot (number) Selection outcome analysed: strands of spaghetti selected by placing in pot (number) Measurement of selection outcome: objective Timing of selection outcome measurement: immediate (≤ 1 day) Consumption outcome analysed: N/A Measurement of consumption outcome: N/A Timing of consumption outcome measurement: N/A	
Funding source	Marketing Science Institute, Tinbergen Institute (Amsterdam), Iowa State Extension Service, Procter & Gamble	
Notes	—	
***Risk of bias***		
**Bias**	**Authors' judgement**	**Support for judgement**
Random sequence generation (selection bias)	Low risk	Comment: method of sequence generation is not described. Author contact confirmed group assignment was randomised and author stated that sequence for group assignment was generated using a "random number generator" (13 March 2013)
Allocation concealment (selection bias)	High risk	Comment: method of concealment is not described. Author contact confirmed group assignment was randomised and author stated that sequence for group assignment was generated using a "random number generator" (13 March 2013). Investigators enrolling participants could possibly foresee assignments
Blinding of participants and personnel (performance bias) Selection outcome	Low risk	Quote: "In individual meetings, each subject was told that some basic home economics‐related information about two different types of products were being collected. The subject was then led to one of four isolated cubicles in which there was one of the two products in one of the two package size conditions. The research assistant assigned to each cubicle was blind to the purpose of the study." Comment: blinding of study participants attempted and unlikely that the blinding could have been broken. Very unlikely that key study personnel were blinded, but the review authors judge that the outcome is not likely to be influenced by lack of blinding of key study personnel
Blinding of outcome assessment (detection bias) Selection outcome	Low risk	Comment: no blinding of outcome assessment, but the review authors judge that the outcome measurement is not likely to be influenced by lack of blinding
Incomplete outcome data (attrition bias) Selection outcome	Low risk	Comment: no missing outcome data for selection outcome
Selective reporting (reporting bias)	Unclear risk	Comment: search for record(s) containing details of study protocol conducted in ClinicalTrials.gov and the WHO International Clinical Trials Registry Platform (ICTRP). No records found. Insufficient information to permit judgement of 'low risk' or 'high risk'
Other bias #1 ‐ Baseline comparability of participant characteristics between groups	Unclear risk	Comment: study uses a between‐subjects design. Insufficient information to permit judgement of 'low risk' or 'high risk'
Other bias #2 ‐ Consistency in intervention delivery	Low risk	Quote: "When the subject arrived, the research assistant read a scenario involving the use of the product (Crisco brand oil: "You are frying a chicken dinner for yourself and another adult"; Creamette brand spaghetti: "You are making spaghetti for yourself and another adult"). The subject was asked to show how much of the product she would use in this situation..." Comment: information and instructions provided to participants appear to have been standardised between the compared study conditions. No specific instructions were provided to participants other than those described in the quote above and therefore participants' compliance with instructions is not applicable
Summary of risk of bias Selection outcome	High risk	High risk

**Table:** Wansink 1996b (S2)

Methods	Study design: between‐subjects randomised controlled trial	
Participants	Setting: laboratory setting Geographical region: Pennsylvania, USA Number of enrolled participants: 126 adults Number (%) of enrolled participants completing the study: 126 (100%) Study completers ‐ mean age (SD): not reported Study completers ‐ sex: 100% female Study completers ‐ mean BMI kg/m^2^ (SD): not reported Specific social or cultural characteristics: yes. Adults recruited via parent‐teacher associations Socio‐economic status context: low deprivation Inclusion criteria: none reported Exclusion criteria: none reported	
Interventions	Manipulated product type: food Manipulation: portion with tableware (volume of serving pitcher) Duration of exposure to intervention: ≤ 1 day Social setting: selecting alone Study arms: 1000 ml pitcher of tap water to pour; 2000 ml pitcher of tap water to pour; 1000 ml pitcher of bottled water to pour; 2000 ml pitcher of bottled water to pour Number of comparisons analysed: 2 Comparisons analysed: Comparison 1: Intervention 1: 1000 ml pitcher of tap water to pour; *versus* Intervention 2: 2000 ml pitcher of tap water to pour Comparison 2: Intervention 1: 1000 ml pitcher of bottled water to pour; *versus* Intervention 2: 2000 ml pitcher of bottled water to pour Concurrent intervention components: no	
Outcomes	Outcomes reported in study: volume of water selected by pouring into glass (millilitres) Selection outcome analysed: volume of water selected by pouring into glass (millilitres) Measurement of selection outcome: objective Timing of selection outcome measurement: immediate (≤ 1 day) Consumption outcome analysed: N/A Measurement of consumption outcome: N/A Timing of consumption outcome measurement: N/A	
Funding source	Marketing Science Institute, Tinbergen Institute (Amsterdam), Iowa State Extension Service, Procter & Gamble	
Notes	—	
***Risk of bias***		
**Bias**	**Authors' judgement**	**Support for judgement**
Random sequence generation (selection bias)	Low risk	Comment: method of sequence generation is not described. Author contact confirmed group assignment was randomised and author stated that sequence for group assignment was generated using a "random number generator" (13 March 2013)
Allocation concealment (selection bias)	High risk	Comment: method of concealment is not described. Author contact confirmed group assignment was randomised and author stated that sequence for group assignment was generated using a "random number generator" (13 March 2013). Investigators enrolling participants could possibly foresee assignments
Blinding of participants and personnel (performance bias) Selection outcome	Low risk	Quote: "Subjects were told that some basic home economics‐related information about different topics were being collected." Comment: no blinding or incomplete blinding of study participants and key study personnel, but the review authors judge that the outcome is not likely to be influenced by lack of blinding
Blinding of outcome assessment (detection bias) Selection outcome	Low risk	Comment: no blinding of outcome assessment, but the review authors judge that the outcome measurement is not likely to be influenced by lack of blinding
Incomplete outcome data (attrition bias) Selection outcome	Low risk	Comment: no missing outcome data for selection outcome
Selective reporting (reporting bias)	Unclear risk	Comment: search for record(s) containing details of study protocol conducted in ClinicalTrials.gov and the WHO International Clinical Trials Registry Platform (ICTRP). No records found. Insufficient information to permit judgement of 'low risk' or 'high risk'
Other bias #1 ‐ Baseline comparability of participant characteristics between groups	Unclear risk	Comment: study uses a between‐subjects design. Insufficient information to permit judgement of 'low risk' or 'high risk'
Other bias #2 ‐ Consistency in intervention delivery	Low risk	Quote: "Each subject was randomly assigned to one of the four conditions noted previously and was told, "Imagine that when you get home this afternoon, you go to the refrigerator and take out a container of bottled water (tap water) to pour yourself a drink. To make it easier to pour we've put the water in a pitcher. This afternoon when you get home, how much will you pour?"" Comment: information and instructions provided to participants appear to have been standardised between the compared study conditions. No specific instructions were provided to participants other than those described in the quote above and therefore participants' compliance with instructions is not applicable
Summary of risk of bias Selection outcome	High risk	High risk

**Table:** Wansink 1996c (S4)

Methods	Study design: between‐subjects randomised controlled trial	
Participants	Setting: laboratory setting Geographical region: New Hampshire, USA Number of enrolled participants: 184 adults Number (%) of enrolled participants completing the study: 184 (100%) Study completers ‐ mean age (SD): not reported Study completers ‐ sex: 100% female Study completers ‐ mean BMI kg/m^2^ (SD): not reported Specific social or cultural characteristics: yes. Adults recruited via parent‐teacher associations Socio‐economic status context: low deprivation Inclusion criteria: none reported Exclusion criteria: none reported	
Interventions	Manipulated product type: food Manipulation: portion with package Duration of exposure to intervention: ≤ 1 day Social setting: selecting alone Study arms: 675 strand package of Creamette brand spaghetti plus 114 candy package of M&Ms; 1350 strand package of Creamette brand spaghetti plus 228 candy package of M&Ms; 2025 strand package of Creamette brand spaghetti plus 342 candy package of M&Ms Number of comparisons analysed: 2 Comparisons analysed: Intervention 1: 114 candy package of M&Ms; *versus* Intervention 2: 228 candy package of M&Ms Concurrent intervention components: no	
Outcomes	Outcomes reported in study: M&M candies selected by pouring into a bowl (number); average strands of spaghetti selected by placing in pot (number) Selection outcome analysed: M&M candies selected by pouring into a bowl (number) Measurement of selection outcome: objective Timing of selection outcome measurement: immediate (≤ 1 day) Consumption outcome analysed: N/A Measurement of consumption outcome: N/A Timing of consumption outcome measurement: N/A	
Funding source	Marketing Science Institute, Tinbergen Institute (Amsterdam), Iowa State Extension Service, Procter & Gamble	
Notes	Study authors contacted for missing data but data no longer available As study participants were exposed to 2 different products on separate occasions, we selected outcome data related to one product (M&Ms) for analysis based on its greater similarity with manipulated products in other included studies. No usable outcome data for the comparison 'Intervention 1: 228 candy package of M&Ms; versus Intervention 2: 2342 candy package of M&Ms' because associated standard deviations were not reported, could not be computed from reported test statistics and could not be obtained by contacting the study authors	
***Risk of bias***		
**Bias**	**Authors' judgement**	**Support for judgement**
Random sequence generation (selection bias)	Low risk	Comment: method of sequence generation is not described. Author contact confirmed group assignment was randomised and author stated that sequence for group assignment was generated using a "random number generator" (13 March 2013)
Allocation concealment (selection bias)	High risk	Comment: method of concealment is not described. Author contact confirmed group assignment was randomised and author stated that sequence for group assignment was generated using a "random number generator" (13 March 2013). Investigators enrolling participants could possibly foresee assignments
Blinding of participants and personnel (performance bias) Selection outcome	Low risk	Quote: "Each subject was met individually and told that some basic home economics‐related information about three different types of products were being collected. The subject then entered one of three isolated cubicles in which there was one product representing one of the three package size conditions." Comment: blinding of study participants attempted and unlikely that the blinding could have been broken. Very unlikely that key study personnel were blinded, but the review authors judge that the outcome is not likely to be influenced by lack of blinding of key study personnel
Blinding of outcome assessment (detection bias) Selection outcome	Low risk	Comment: no blinding of outcome assessment, but the review authors judge that the outcome measurement is not likely to be influenced by lack of blinding
Incomplete outcome data (attrition bias) Selection outcome	Low risk	Comment: no missing outcome data for selection outcome
Selective reporting (reporting bias)	Unclear risk	Comment: search for record(s) containing details of study protocol conducted in ClinicalTrials.gov and the WHO International Clinical Trials Registry Platform (ICTRP). No records found. Insufficient information to permit judgement of 'low risk' or 'high risk'
Other bias #1 ‐ Baseline comparability of participant characteristics between groups	Unclear risk	Comment: study uses a between‐subjects design. Insufficient information to permit judgement of 'low risk' or 'high risk'
Other bias #2 ‐ Consistency in intervention delivery	Low risk	Quote: "the research assistant assigned to each cubicle described a brief scenario that involved the use of the product (...M&M's brand candy: "You are watching a movie on television by yourself"). The research assistant then asked the subject to indicate how much of the product she would use in this situation." Comment: information and instructions provided to participants appear to have been standardised between the compared study conditions. No specific instructions were provided to participants other than those described in the quote above and therefore participants' compliance with instructions is not applicable
Summary of risk of bias Selection outcome	High risk	High risk

**Table:** Wansink 2001

Methods	Study design: between‐subjects randomised controlled trial	
Participants	Setting: field setting. Cinema Geographical region: Chicago, IL, USA Number of enrolled participants: 161 adults Number (%) of enrolled participants completing the study: 161 (100%) Study completers ‐ mean age (SD): not reported Study completers ‐ sex: 44% female Study completers ‐ mean BMI kg/m^2^ (SD): not reported Specific social or cultural characteristics: yes. Cinema‐goers Socio‐economic status context: low deprivation Inclusion criteria: none reported Exclusion criteria: none reported	
Interventions	Manipulated product type: food Manipulation: portion with package Duration of exposure to intervention: ≤ 1 day Social setting: consuming with others Study arms: medium (120 g) container of popcorn; large (240 g) container of popcorn Number of comparisons analysed: 1 Comparisons analysed: Comparison 1: Intervention 1: medium (120 g) container of popcorn; *versus* Intervention 2: large (240 g) container of popcorn Concurrent intervention components: no	
Outcomes	Outcomes reported in study: amount of popcorn consumed (grams) Selection outcome analysed: N/A Measurement of selection outcome: N/A Timing of selection outcome measurement: N/A Consumption outcome analysed: amount of popcorn consumed (grams) Measurement of consumption outcome: objective Timing of consumption outcome measurement: immediate (≤ 1 day)	
Funding source	Not reported	
Notes	Study authors contacted for missing data but data no longer available	
***Risk of bias***		
**Bias**	**Authors' judgement**	**Support for judgement**
Random sequence generation (selection bias)	Unclear risk	Comment: method of sequence generation is not described. Insufficient information about the sequence generation process to permit judgement of 'low risk' or 'high risk'
Allocation concealment (selection bias)	Unclear risk	Comment: method of concealment is not described. Insufficient information to permit judgement of 'low risk' or 'high risk'
Blinding of participants and personnel (performance bias) Consumption outcome	Unclear risk	Quote: "The subjects in this study were moviegoers who had independently elected to see the 1:30 and 2:15 screenings of "Payback'' (starring Mel Gibson) on its opening weekend at a large theatre near Chicago in April 1998. Upon purchasing their ticket, each of the 161 movie‐ goers were given a coupon that entitled them to a "free popcorn and a soft drink'' to purportedly celebrate the theatre's 1 year anniversary. When they arrived in the theatre they were given a soft drink and were randomly given either a medium (120 grams) or a large (240 grams) container of free popcorn." Comment: blinding of study participants attempted. However, it is possible that blinding of study participants could have been broken in some cases due to participants in one condition seeing ‐ and therefore becoming aware of ‐ the different sizes of popcorn container being handed to participants in the other condition on entry to the theatre, and it is possible that the outcome may be influenced by lack of blinding of study participants. Very unlikely that key study personnel were blinded, but the review authors judge that the outcome is not likely to be influenced by lack of blinding of key study personnel
Blinding of outcome assessment (detection bias) Consumption outcome	Low risk	Comment: no blinding of outcome assessment, but the review authors judge that the outcome measurement is not likely to be influenced by lack of blinding
Incomplete outcome data (attrition bias) Consumption outcome	Low risk	Comment: no missing outcome data for consumption outcome
Selective reporting (reporting bias)	Unclear risk	Comment: search for record(s) containing details of study protocol conducted in ClinicalTrials.gov and the WHO International Clinical Trials Registry Platform (ICTRP). No records found. Insufficient information to permit judgement of 'low risk' or 'high risk'
Other bias #1 ‐ Baseline comparability of participant characteristics between groups	Unclear risk	Comment: study uses a between‐subjects design. Insufficient information to permit judgement of 'low risk' or 'high risk'
Other bias #2 ‐ Consistency in intervention delivery	Low risk	Comment: information provided to participants appears to have been standardised between the compared study conditions. No specific instructions were provided to participants and therefore participants' compliance with instructions is not applicable
Summary of risk of bias Consumption outcome	Unclear risk	Unclear risk

**Table:** Wansink 2003 (S1)

Methods	Study design: between‐subjects randomised controlled trial	
Participants	Setting: field setting. Cafeteria at residential camp Geographical region: New Hampshire, USA Number of enrolled participants: 97 children Number (%) of enrolled participants completing the study: 97 (100%) Study completers ‐ mean age (SD): 15 (not reported) Study completers ‐ sex: 54.6% female Study completers ‐ mean BMI kg/m^2^ (SD): not reported Specific social or cultural characteristics: children involved in a 6‐week health and fitness camp Socio‐economic status context: low deprivation Inclusion criteria: children Exclusion criteria: none reported	
Interventions	Manipulated product type: food Manipulation: tableware shape Duration of exposure to intervention: ≤ 1 day Social setting: selecting with others Study arms: 22.3 oz juice glass with height of 18.9 cm; 22.3 oz juice glass with height of 10.6 cm Number of comparisons analysed: 1 Comparisons analysed: comparison 1: Intervention 1: 22.3 oz juice glass with height of 18.9 cm; *versus* Intervention 2: 22.3 oz juice glass with height of 10.6 cm Concurrent intervention components: no	
Outcomes	Outcomes reported in study: amount of juice poured (ounces) Selection outcome analysed: amount of juice poured (ounces) Measurement of selection outcome: objective Timing of selection outcome measurement: immediate (≤ 1 day) Consumption outcome analysed: N/A Measurement of consumption outcome: N/A Timing of consumption outcome measurement: N/A	
Funding source	Illinois Attorney General, Dartmouth College Scholars Fund	
Notes	Study authors contacted for missing data but data no longer available	
***Risk of bias***		
**Bias**	**Authors' judgement**	**Support for judgement**
Random sequence generation (selection bias)	Unclear risk	Comment: method of sequence generation is not described. Insufficient information about the sequence generation process to permit judgement of 'low risk' or 'high risk'
Allocation concealment (selection bias)	Unclear risk	Comment: method of concealment is not described. Insufficient information to permit judgement of 'low risk' or 'high risk'
Blinding of participants and personnel (performance bias) Selection outcome	Unclear risk	Quote: "Upon entering the cafeteria line for breakfast on the ninth day of the camp, the children were randomly given a 22.3 oz juice glass that was either relatively short or relatively tall. The height of the former was 10.6 cm, the latter 18.9 cm. As campers helped themselves to one of the juices in the cafeteria line, they were unaware of the use of different shaped glasses." Comment: blinding of study participants attempted. However, it is possible that blinding of study participants could have been broken in some cases due to participants in one condition seeing ‐ and therefore becoming aware of ‐ the different shapes of glasses being handed to participants in the other condition on entry to the cafeteria line, and it is possible that the outcome may be influenced by lack of blinding of study participants. Very unlikely that key study personnel were blinded, but the review authors judge that the outcome is not likely to be influenced by lack of blinding of key study personnel
Blinding of outcome assessment (detection bias) Selection outcome	Low risk	Comment: no blinding of outcome assessment, but the review authors judge that the outcome measurement is not likely to be influenced by lack of blinding
Incomplete outcome data (attrition bias) Selection outcome	Low risk	Comment: no missing outcome data for selection outcome
Selective reporting (reporting bias)	Unclear risk	Comment: search for record(s) containing details of study protocol conducted in ClinicalTrials.gov and the WHO International Clinical Trials Registry Platform (ICTRP). No records found. Insufficient information to permit judgement of 'low risk' or 'high risk'
Other bias #1 ‐ Baseline comparability of participant characteristics between groups	Unclear risk	Comment: study uses a between‐subjects design. Insufficient information to permit judgement of 'low risk' or 'high risk'
Other bias #2 ‐ Consistency in intervention delivery	Low risk	Comment: information provided to participants appears to have been standardised between the compared study conditions. No specific instructions were provided to participants and therefore participants' compliance with instructions is not applicable
Summary of risk of bias Selection outcome	Unclear risk	Unclear risk

**Table:** Wansink 2003 (S2)

Methods	Study design: between‐subjects randomised controlled trial	
Participants	Setting: field setting. Cafeteria at residential camp Geographical region: Massachusetts, USA Number of enrolled participants: 89 adults Number (%) of enrolled participants completing the study: 89 (100%) Study completers ‐ mean age (SD): 37.2 (not reported) Study completers ‐ sex: 22.5% female Study completers ‐ mean BMI kg/m^2^ (SD): not reported Specific social or cultural characteristics: adults involved in a weekend music camp Socio‐economic status context: low deprivation Inclusion criteria: not stated Exclusion criteria: none reported	
Interventions	Manipulated product type: food Manipulation: tableware shape Duration of exposure to intervention: ≤ 1 day Social setting: selecting with others Study arms: 22.3 oz juice glass with height of 18.9 cm; 22.3 oz juice glass with height of 10.6 cm Number of comparisons analysed: 1 Comparisons analysed: Comparison 1: Intervention 1: 22.3 oz juice glass with height of 18.9 cm; *versus* Intervention 2: 22.3 oz juice glass with height of 10.6 cm Concurrent intervention components: no	
Outcomes	Outcomes reported in study: amount of juice poured (ounces) Selection outcome analysed: amount of juice poured (ounces) Measurement of selection outcome: objective Timing of selection outcome measurement: immediate (≤ 1 day) Consumption outcome analysed: N/A Measurement of consumption outcome: N/A Timing of consumption outcome measurement: N/A	
Funding source	Illinois Attorney General, Dartmouth College Scholars Fund	
Notes	Study authors contacted for missing data but data no longer available	
***Risk of bias***		
**Bias**	**Authors' judgement**	**Support for judgement**
Random sequence generation (selection bias)	Unclear risk	Comment: method of sequence generation is not described. Insufficient information about the sequence generation process to permit judgement of 'low risk' or 'high risk'
Allocation concealment (selection bias)	Unclear risk	Comment: method of concealment is not described. Insufficient information to permit judgement of 'low risk' or 'high risk'
Blinding of participants and personnel (performance bias) Selection outcome	Unclear risk	Quote: "Upon entering the cafeteria line for breakfast on the second morning of the camp, these adults were randomly given a 22.3‐oz glass that was either relatively short or relatively tall. They were allowed to help themselves to one of five types of juice and were unaware of the use of different‐shaped glasses." Comment: blinding of study participants attempted. However, it is possible that blinding of study participants could have been broken in some cases due to participants in one condition seeing ‐ and therefore becoming aware of ‐ the different shapes of glasses being handed to participants in the other condition on entry to the cafeteria line, and it is possible that the outcome may be influenced by lack of blinding of study participants. Very unlikely that key study personnel were blinded, but the review authors judge that the outcome is not likely to be influenced by lack of blinding of key study personnel
Blinding of outcome assessment (detection bias) Selection outcome	Low risk	Comment: no blinding of outcome assessment, but the review authors judge that the outcome measurement is not likely to be influenced by lack of blinding
Incomplete outcome data (attrition bias) Selection outcome	Low risk	Comment: no missing outcome data for selection outcome
Selective reporting (reporting bias)	Unclear risk	Comment: search for record(s) containing details of study protocol conducted in ClinicalTrials.gov and the WHO International Clinical Trials Registry Platform (ICTRP). No records found. Insufficient information to permit judgement of 'low risk' or 'high risk'
Other bias #1 ‐ Baseline comparability of participant characteristics between groups	Unclear risk	Comment: study uses a between‐subjects design. Insufficient information to permit judgement of 'low risk' or 'high risk'
Other bias #2 ‐ Consistency in intervention delivery	Low risk	Comment: information provided to participants appears to have been standardised between the compared study conditions. No specific instructions were provided to participants and therefore participants' compliance with instructions is not applicable
Summary of risk of bias Selection outcome	Unclear risk	Unclear risk

**Table:** Wansink 2005b

Methods	Study design: between‐subjects randomised controlled trial	
Participants	Setting: field setting. Cinema Geographical region: Philadelphia, USA Number of enrolled participants: 158 adults Number (%) of enrolled participants completing the study: 157 (99.4%) Study completers ‐ mean age (SD): 28.9 (11.8) Study completers ‐ sex: 41.4% female Study completers ‐ mean BMI kg/m^2^ (SD): not reported Specific social or cultural characteristics: yes. Cinema‐goers Socio‐economic status context: low deprivation Inclusion criteria: none reported Exclusion criteria: none reported	
Interventions	Manipulated product type: food Manipulation: portion with package size Duration of exposure to intervention: ≤ 1 day Social setting: consuming with others Study arms: medium (120 g) container of fresh popcorn; large (240 g) container of fresh popcorn; medium (120 g) container of stale popcorn (14 days old) popcorn Number of comparisons analysed: 2 Comparisons analysed: Comparison 1: Intervention 1: medium (120 g) container of fresh popcorn; *versus* Intervention 2: large (240 g) container of fresh popcorn Comparison 2: Intervention 1: medium (120 g) container of stale popcorn (14 days old) popcorn; *versus* Intervention 2: large (240 g) container of stale (14 days old) popcorn Concurrent intervention components: no	
Outcomes	Outcomes reported in study: amount of popcorn consumed (grams) Selection outcome analysed: N/A Measurement of selection outcome: N/A Timing of selection outcome measurement: N/A Consumption outcome analysed: amount of popcorn consumed (grams) Measurement of consumption outcome: objective Timing of consumption outcome measurement: immediate (≤ 1 day)	
Funding source	University of Pennsylvania; Julian Simon Research Fellowship and Food and Brand Lab (University of Illinois)	
Notes	—	
***Risk of bias***		
**Bias**	**Authors' judgement**	**Support for judgement**
Random sequence generation (selection bias)	Unclear risk	Comment: method of sequence generation is not described. Insufficient information about the sequence generation process to permit judgement of 'low risk' or 'high risk'
Allocation concealment (selection bias)	Unclear risk	Comment: method of concealment is not described. Insufficient information to permit judgement of 'low risk' or 'high risk'
Blinding of participants and personnel (performance bias) Consumption outcome	Unclear risk	Quote: "This study investigated moviegoers who had independently elected to see 1 of 4 showings (2 consecutive shows on 2 consecutive evenings) of the film Stargate at a second‐run theatre in a northern Philadelphia suburb. On purchasing their ticket, all of the 177 adult moviegoers were asked if they would consent to answer a few questions related to the "theater and its concessions" following the movie... Because of their participation in the study, moviegoers were then told that they would be given free popcorn and a drink... The study employed a 2 × 2 between‐subjects design wherein each individual was randomly given a medium (120 g) or a large (240 g) container of popcorn that was either fresh or stale." Comment: insufficient information to permit judgement of 'low risk' or 'high risk' Unclear whether blinding of study participants was attempted and unclear whether blinding of study participants, if attempted, could have been broken in some cases. If broken, it is possible that the outcome may be influenced by lack of blinding of study participants. Very unlikely that key study personnel were blinded, but the review authors judge that the outcome is not likely to be influenced by lack of blinding of key study personnel
Blinding of outcome assessment (detection bias) Consumption outcome	Low risk	Comment: no blinding of outcome assessment, but the review authors judge that the outcome measurement is not likely to be influenced by lack of blinding
Incomplete outcome data (attrition bias) Consumption outcome	Low risk	Comment: no reason for missing outcome data for consumption outcome provided. The low proportion (1 participant, 1% of study sample) of exclusions means that the review authors judge that the plausible effect size among missing outcomes is unlikely to be enough to have an important impact on the observed effect size
Selective reporting (reporting bias)	Unclear risk	Comment: search for record(s) containing details of study protocol conducted in ClinicalTrials.gov and the WHO International Clinical Trials Registry Platform (ICTRP). No records found. Insufficient information to permit judgement of 'low risk' or 'high risk'
Other bias #1 ‐ Baseline comparability of participant characteristics between groups	Low risk	Quote: "As Table 1 indicates, the moviegoers in each randomized subsample were similar in terms of their age...and in terms of their gender mix..." Comment: study uses a between‐subjects design. No evidence of differences between comparison groups in terms of measured baseline participant characteristics
Other bias #2 ‐ Consistency in intervention delivery	Low risk	Comment: information provided to participants appears to have been standardised between the compared study conditions. No specific instructions were provided to participants and therefore participants' compliance with instructions is not applicable
Summary of risk of bias Consumption outcome	Unclear risk	Unclear risk

**Table:** Wansink 2005d

Methods	Study design: between‐subjects randomised controlled trial	
Participants	Setting: laboratory setting Geographical region: Urbana‐Champaign, IL, USA Number of enrolled participants: 50 adults Number (%) of enrolled participants completing the study: 50 (100%) Study completers ‐ mean age (SD): not reported Study completers ‐ sex: not reported Study completers ‐ mean BMI kg/m^2^ (SD): not reported Specific social or cultural characteristics: Army and Marine Reserve Officers' Training Corps students Socio‐economic status context: low deprivation Inclusion criteria: none reported Exclusion criteria: none reported	
Interventions	Manipulated product type: food Manipulation: tableware shape (shape of bottle); water, from 10‐gallon water container Duration of exposure to intervention: ≤ 1 day Social setting: selecting/consuming alone Study arms: taller, narrower 32 ounce clear plastic bottle to fill with water; shorter, wider 32 ounce clear plastic bottle to fill with water Number of comparisons analysed: 1 Comparisons analysed: Comparison 1: Intervention 1: taller, narrower 32 ounce clear plastic bottle to fill with water; *versus* Intervention 2: shorter, wider 32 ounce clear plastic bottle to fill with water Concurrent intervention components: no	
Outcomes	Outcomes reported in study: amount of water poured (ounces); self estimated amount of water poured (ounces); amount of water consumed (ounces) Selection outcome analysed: amount of water poured (ounces) Measurement of selection outcome: objective Timing of selection outcome measurement: immediate (≤ 1 day) Consumption outcome analysed: amount of water consumed (ounces) Measurement of consumption outcome: objective Timing of consumption outcome measurement: immediate (≤ 1 day)	
Funding source	Not reported	
Notes	—	
***Risk of bias***		
**Bias**	**Authors' judgement**	**Support for judgement**
Random sequence generation (selection bias)	Low risk	Comment: method of sequence generation is not described. Author contact confirmed group assignment was randomised and author stated that sequence for group assignment was generated using a "random number generator" (13 March 2013)
Allocation concealment (selection bias)	High risk	Comment: author contact confirmed group assignment was randomised and author stated that sequence for group assignment was generated using a "random number generator" (13 March 2013). Investigators enrolling participants could possibly foresee assignments
Blinding of participants and personnel (performance bias) Selection outcome	Unclear risk	Quote: "Upon entering the room where the study was to take place, the [participants] were told that they would be trying some different foods and that it was important that they not be thirsty before trying the foods. Two assistants then handed out empty (clear) plastic water bottles to the individuals assembled there. Both bottles held 32 ounces of water, but one‐half were tall and narrow and the other half were shorter and wider." Comment: blinding of study participants attempted. However, it is possible that blinding of study participants could have been broken in some cases due to participants in one condition seeing ‐ and therefore becoming aware of ‐ the different sizes of water bottles being handed to participants in the other condition in the room where the study took place, and it is possible that the outcome may be influenced by lack of blinding of study participants. Very unlikely that key study personnel were blinded, but the review authors judge that the outcome is not likely to be influenced by lack of blinding of key study personnel
Blinding of participants and personnel (performance bias) Consumption outcome	Unclear risk	Quote: "Upon entering the room where the study was to take place, the [participants] were told that they would be trying some different foods and that it was important that they not be thirsty before trying the foods. Two assistants then handed out empty (clear) plastic water bottles to the individuals assembled there. Both bottles held 32 ounces of water, but one‐half were tall and narrow and the other half were shorter and wider." Comment: blinding of study participants attempted. However, it is possible that blinding of study participants could have been broken in some cases due to participants in one condition seeing ‐ and therefore becoming aware of ‐ the different sizes of water bottles being handed to participants in the other condition in the room where the study took place, and it is possible that the outcome may be influenced by lack of blinding of study participants. Very unlikely that key study personnel were blinded, but the review authors judge that the outcome is not likely to be influenced by lack of blinding of key study personnel
Blinding of outcome assessment (detection bias) Selection outcome	Low risk	Comment: no blinding of outcome assessment, but the review authors judge that the outcome measurement is not likely to be influenced by lack of blinding
Blinding of outcome assessment (detection bias) Consumption outcome	Low risk	Comment: no blinding of outcome assessment, but the review authors judge that the outcome measurement is not likely to be influenced by lack of blinding
Incomplete outcome data (attrition bias) Selection outcome	Low risk	Comment: no missing outcome data for selection outcome
Incomplete outcome data (attrition bias) Consumption outcome	Low risk	Comment: no missing outcome data for consumption outcome
Selective reporting (reporting bias)	Unclear risk	Comment: search for record(s) containing details of study protocol conducted in ClinicalTrials.gov and the WHO International Clinical Trials Registry Platform (ICTRP). No records found. Insufficient information to permit judgement of 'low risk' or 'high risk'
Other bias #1 ‐ Baseline comparability of participant characteristics between groups	Unclear risk	Comment: study uses a between‐subjects design. Insufficient information to permit judgement of 'low risk' or 'high risk'
Other bias #2 ‐ Consistency in intervention delivery	Low risk	Comment: information provided to participants appears to have been standardised between the compared study conditions. No specific instructions were provided to participants and therefore participants' compliance with instructions is not applicable
Summary of risk of bias Selection outcome	High risk	High risk
Summary of risk of bias Consumption outcome	High risk	High risk

**Table:** Wansink 2006

Methods	Study design: between‐subjects randomised controlled trial	
Participants	Setting: field setting, ice cream social in a university department Geographical region: Urbana‐Champaign, IL, USA Number of enrolled participants: 85 adults Number (%) of enrolled participants completing the study: 85 (100%) Study completers ‐ mean age (SD): not reported Study completers ‐ sex: 32% female Study completers ‐ mean BMI kg/m^2^ (SD): not reported Specific social or cultural characteristics: yes. University faculty, graduate students and staff Socio‐economic status context: low deprivation Inclusion criteria: none reported Exclusion criteria: none reported	
Interventions	Manipulated product type: food Manipulation: tableware size (2 manipulations: serving bowl size; ice cream scoop size) Duration of exposure to intervention: ≤ 1 day Social setting: selecting alone Study arms: small (17 oz) bowl, small (2 oz) ice cream scoop; small (17 oz) bowl, large (3 oz) ice cream scoop; large (34 oz) bowl, small (2 oz) ice cream scoop; large (34 oz) bowl, large (3 oz) ice cream scoop Number of comparisons analysed: 2 Comparisons analysed: Comparison 1: Intervention 1: small (17 oz) bowl; *versus* Intervention 2: large (34 oz) bowl Comparison 2: Intervention 1: small (2 oz) ice cream scoop; *versus* Intervention 2: large (3oz) ice cream scoop Concurrent intervention components: no	
Outcomes	Outcomes reported in study: amount of ice cream self‐served (ounces); number of scoopfuls of ice cream self served (N); average amount of ice cream per scoopful (ounces) Selection outcome analysed: amount of ice cream self served (ounces) Measurement of selection outcome: objective Timing of selection outcome measurement: immediate (≤ 1 day) Consumption outcome analysed: N/A Measurement of consumption outcome: N/A Timing of consumption outcome measurement: N/A	
Funding source	Self funded	
Notes	Outcome data for each manipulation analysed separately (one comparison each)	
***Risk of bias***		
**Bias**	**Authors' judgement**	**Support for judgement**
Random sequence generation (selection bias)	Unclear risk	Comment: method of sequence generation is not described. Insufficient information about the sequence generation process to permit judgement of 'low risk' or 'high risk'
Allocation concealment (selection bias)	Unclear risk	Comment: method of concealment is not described. Insufficient information to permit judgement of 'low risk' or 'high risk'
Blinding of participants and personnel (performance bias) Selection outcome	Unclear risk	Quote: "[Participants] received an e‐mail invitation to attend an ice cream social to celebrate the success of a colleague... Participants were blind to the conditions. Upon individually entering the ice cream line, the participants were randomly given either a smaller (17 oz) or a larger (34 oz) bowl... In addition, participants were either given smaller (2 oz) or larger (3 oz) serving spoons with which to dish out their ice cream. Because participants individually helped themselves to the available ice cream in the cafeteria line, they were unaware that other participants had been given different‐sized bowls and serving spoons." Comment: blinding of study participants attempted. However, it is possible that blinding of study participants could have been broken in some cases due to participants in one condition seeing ‐ and therefore becoming aware of ‐ the different sizes of bowls and serving spoons being handed to participants in the other conditions on entry to the cafeteria line, and it is possible that the outcome may be influenced by lack of blinding of study participants. Very unlikely that key study personnel were blinded, but the review authors judge that the outcome is not likely to be influenced by lack of blinding of key study personnel
Blinding of outcome assessment (detection bias) Selection outcome	Low risk	Comment: no blinding of outcome assessment, but the review authors judge that the outcome measurement is not likely to be influenced by lack of blinding
Incomplete outcome data (attrition bias) Selection outcome	Low risk	Comment: no missing outcome data for selection outcome.
Selective reporting (reporting bias)	Unclear risk	Comment: search for record(s) containing details of study protocol conducted in ClinicalTrials.gov and the WHO International Clinical Trials Registry Platform (ICTRP). No records found. Insufficient information to permit judgement of 'low risk' or 'high risk'
Other bias #1 ‐ Baseline comparability of participant characteristics between groups	Unclear risk	Comment: study uses a between‐subjects design. Insufficient information to permit judgement of 'low risk' or 'high risk'
Other bias #2 ‐ Consistency in intervention delivery	Low risk	Comment: information provided to participants appears to have been standardised between the compared study conditions. No specific instructions were provided to participants and therefore participants' compliance with instructions is not applicable
Summary of risk of bias Selection outcome	Unclear risk	Unclear risk

**Table:** Wansink 2011a (S4)

Methods	Study design: between‐subjects randomised controlled trial	
Participants	Setting: field setting. Residential music camp Geographical region: Massachusetts, USA Number of enrolled participants: 81 adults Number (%) of enrolled participants completing the study: 81 (100%) Study completers ‐ mean age (SD): 40 (not reported) Study completers ‐ sex: 80.3% female Study completers ‐ mean BMI kg/m^2^ (SD): not reported Specific social or cultural characteristics: adults involved in a weekend music camp Socio‐economic status context: low deprivation Inclusion criteria: not stated Exclusion criteria: none reported	
Interventions	Manipulated product type: food Manipulation: tableware size Duration of exposure to intervention: ≤ 1 day Social setting: selecting alone Study arms: smaller (diameter of 15.2 cm) identically shaped bowl with a depth of 5.1 cm; larger (diameter of 30.5 cm) identically shaped bowl with a depth of 5.1 cm Number of comparisons analysed: 1 Comparisons analysed: Comparison 1: Intervention 1: smaller (diameter of 15.2 cm) bowl; *versus* Intervention 2: larger (diameter of 30.5 cm) bowl Concurrent intervention components: no	
Outcomes	Outcomes reported in study: amount of breakfast cereal self served (grams) Selection outcome analysed: amount of breakfast cereal self served (grams) Measurement of selection outcome: objective Timing of selection outcome measurement: immediate (≤ 1 day) Consumption outcome analysed: N/A Measurement of consumption outcome: N/A Timing of consumption outcome measurement: N/A	
Funding source	Illinois Attorney General	
Notes	Study authors contacted for missing data but data no longer available	
***Risk of bias***		
**Bias**	**Authors' judgement**	**Support for judgement**
Random sequence generation (selection bias)	Unclear risk	Comment: method of sequence generation is not described. Insufficient information about the sequence generation process to permit judgement of 'low risk' or 'high risk'
Allocation concealment (selection bias)	Unclear risk	Comment: method of concealment is not described. Insufficient information to permit judgement of 'low risk' or 'high risk'
Blinding of participants and personnel (performance bias) Selection outcome	Unclear risk	Quote: "Upon entering the cafeteria line for breakfast one morning, participants were randomly given either a smaller or larger (d = 15.2 cm vs. d = 30.5 cm), identically shaped bowl, both having a depth of 5.1 cm. Because participants arrived at staggered times, this could be done without them noticing that they had received a different‐sized bowl than other participants... None of the participants commented on the size of the bowls during debriefings." Comment: blinding of study participants attempted. However, it is possible that blinding of study participants could have been broken in some cases due to participants in one condition seeing ‐ and therefore becoming aware of ‐ the different sizes of bowls being handed to participants in the other condition on entry to the cafeteria line, and it is possible that the outcome may be influenced by lack of blinding of study participants. Very unlikely that key study personnel were blinded, but the review authors judge that the outcome is not likely to be influenced by lack of blinding of key study personnel
Blinding of outcome assessment (detection bias) Selection outcome	Low risk	Comment: no blinding of outcome assessment, but the review authors judge that the outcome measurement is not likely to be influenced by lack of blinding
Incomplete outcome data (attrition bias) Selection outcome	Low risk	Comment: no missing outcome data for selection outcome
Selective reporting (reporting bias)	Unclear risk	Comment: search for record(s) containing details of study protocol conducted in ClinicalTrials.gov and the WHO International Clinical Trials Registry Platform (ICTRP). No records found. Insufficient information to permit judgement of 'low risk' or 'high risk'
Other bias #1 ‐ Baseline comparability of participant characteristics between groups	Unclear risk	Comment: study uses a between‐subjects design. Insufficient information to permit judgement of 'low risk' or 'high risk'
Other bias #2 ‐ Consistency in intervention delivery	Low risk	Comment: information provided to participants appears to have been standardised between the compared study conditions. No specific instructions were provided to participants and therefore participants' compliance with instructions is not applicable
Summary of risk of bias Selection outcome	Unclear risk	Unclear risk

**Table:** Wansink 2011b

Methods	Study design: between‐subjects cluster‐randomised controlled trial	
Participants	Setting: laboratory setting Geographical region: USA Number of enrolled participants: 42 adults Number (%) of enrolled participants completing the study: 37 (88.1%) Study completers ‐ mean age (SD): 20.3 (1.1) Study completers ‐ sex: 40.5% female Study completers ‐ mean BMI kg/m^2^ (SD): 23.8 (3.9) Specific social or cultural characteristics: yes. Undergraduate students Socio‐economic status context: low deprivation Inclusion criteria: being a student was only criterion Exclusion criteria: none reported	
Interventions	Manipulated product type: food Manipulation: package size Duration of exposure to intervention: ≤ 1 day Social setting: consuming with others Study arms: package of crackers sub‐divided into 4 smaller 100‐calorie subpackaged crackers; one large 400‐calorie package of crackers Number of comparisons analysed: 1 Comparisons analysed: comparison 1: Intervention 1: package sub‐divided into 4 smaller 100‐calorie subpackaged crackers; *versus* Intervention 2: one large 400‐calorie package of crackers Concurrent intervention components: no	
Outcomes	Outcomes reported in study: energy intake from crackers (kcal) Selection outcome analysed: N/A Measurement of selection outcome: N/A Timing of selection outcome measurement: N/A Consumption outcome analysed: energy intake from crackers (kcal) Measurement of consumption outcome: objective Timing of consumption outcome measurement: immediate (≤ 1 day)	
Funding source	Not reported	
Notes	—	
***Risk of bias***		
**Bias**	**Authors' judgement**	**Support for judgement**
Random sequence generation (selection bias)	Unclear risk	Comment: method of sequence generation is not described. Insufficient information about the sequence generation process to permit judgement of 'low risk' or 'high risk'
Allocation concealment (selection bias)	Low risk	Comment: participating small groups of undergraduates appear to have been randomised to assignment group concurrently, after individuals had been recruited to the study. The review authors therefore judge that any lack of concealment of allocation sequence is unlikely to be an issue for risk of bias
Blinding of participants and personnel (performance bias) Consumption outcome	Low risk	Quote: "The 10 experimental sessions involved four to five participants, and each session was randomly assigned to a condition. Participants were either given one large 400‐calorie package of crackers or a similar‐sized package that had then been sub‐divided into four smaller 100‐calorie sub‐packaged crackers... Participants were told that they would watch a television comedy and would be asked questions about it. They were also told—in an offhanded manner—that there had been a reception the night before, and there were some leftover crackers they could eat if they wished. One half of the participants were given one 400 calorie bag of crackers, and the other half was given four 100 calorie bags of crackers." Comment: blinding of study participants attempted and unlikely that the blinding could have been broken. Very unlikely that key study personnel were blinded, but the review authors judge that the outcome is not likely to be influenced by lack of blinding of key study personnel
Blinding of outcome assessment (detection bias) Consumption outcome	Low risk	Comment: no blinding of outcome assessment, but the review authors judge that the outcome measurement is not likely to be influenced by lack of blinding
Incomplete outcome data (attrition bias) Consumption outcome	Low risk	Quote: "We excluded three participants who failed to report their weight and height and two outliers who consumed >2 s.d. from the mean intake scores, leaving 37 participants..." Comment: the first reason for missing data for consumption outcome is the study authors' decision to exclude participants who failed to report their weight and height from the analysis. This reason for missing outcome data is unlikely to be related to consumption outcome. The second reason for missing outcome data for consumption outcome is the study authors' decision to exclude outliers (> 2 SDs from mean consumption) from the analysis. The low proportion (2 participants, 5% of study sample) of exclusions due to outliers means that the review authors judge that the plausible effect size among missing outcomes is unlikely to be enough to have an important impact on the observed effect size
Selective reporting (reporting bias)	Unclear risk	Comment: search for record(s) containing details of study protocol conducted in ClinicalTrials.gov and the WHO International Clinical Trials Registry Platform (ICTRP). No records found. Insufficient information to permit judgement of 'low risk' or 'high risk'
Other bias #1 ‐ Baseline comparability of participant characteristics between groups	Low risk	Quote: "There was no difference between the BMI of those assigned to the large‐package condition...and those to the small condition..." Comment: study uses a between‐subjects design. No evidence of differences between comparison groups in terms of measured baseline participant characteristics
Other bias #2 ‐ Consistency in intervention delivery	Low risk	Comment: information provided to participants appears to have been standardised between the compared study conditions. No specific instructions were provided to participants and therefore participants' compliance with instructions is not applicable
Summary of risk of bias Consumption outcome	Unclear risk	Unclear risk

BMI: body mass index N/A: not applicable SD: standard deviation

### Characteristics of excluded studies [ordered by study ID]

**Table:**

Study	Reason for exclusion
Andrade 2008	No eligible interventions (within‐study comparisons)
Ashton 1978	No eligible interventions (within‐study comparisons)
Attwood 2012	No eligible interventions (within‐study comparisons)
Balagura 1974	Animal study (non‐human participants)
Bell 2003	No measurement (assessment) of selection or consumption outcomes
Blum 2007	Not an eligible study design
Bohnert 2011	No eligible interventions (within‐study comparisons)
Boyer 2012	No eligible interventions (within‐study comparisons)
Brown 2006	Not an empirical study
Caljouw 2014	No eligible interventions (within‐study comparisons)
Campbell 1996	No eligible interventions (within‐study comparisons)
Chait 1982a	No eligible interventions (within‐study comparisons)
Chait 1982b	No eligible interventions (within‐study comparisons)
Chandler 2009	No eligible interventions (within‐study comparisons)
Chandon 2009	No eligible interventions (within‐study comparisons)
Chang 2012	Not an eligible study design
Cleghorn 2010	No eligible interventions (within‐study comparisons)
Cluskey 1999	Not an eligible study design
Collings 2008	No eligible interventions (within‐study comparisons)
Cullen 2005	No eligible interventions (within‐study comparisons)
Cunningham 2011	Not an empirical study
Divert 2015	No eligible interventions (within‐study comparisons)
Edelman 1986	Not an eligible study design
Ello‐Martin 2005	Not an empirical primary study
Etten 1995	No eligible interventions (within‐study comparisons)
Farleigh 1990	Not an eligible study design
Faucher 2010	No eligible interventions (within‐study comparisons)
Freedman 2010	Not an eligible study design
French 2014	No eligible interventions (within‐study comparisons)
Garber 2008	No measurement (assessment) of selection or consumption outcomes
Geaney 2013	No eligible interventions (within‐study comparisons)
Geier 2006	Not an eligible study design
Gillis 2009	No eligible interventions (within‐study comparisons)
Goldfarb 1972	No eligible interventions (within‐study comparisons)
Gosnell 2001	No eligible interventions (within‐study comparisons)
Greenfield 1983	Not an eligible study design
Greenfield 1984	Not an eligible study design
Gritz 1976	No eligible interventions (within‐study comparisons)
Hackbart 2009	Not an eligible study design
Haisfield 2011	No eligible interventions (within‐study comparisons)
Hartstein 2008	Not an eligible study design
Head 1977	No eligible interventions (within‐study comparisons)
Healthy Study Group 2009	No eligible interventions (within‐study comparisons)
Healthy Study Group 2012	No eligible interventions (within‐study comparisons)
Higgins 1964	No eligible interventions (within‐study comparisons)
Huyghe 2013	No eligible interventions (within‐study comparisons)
Jaeger 2011	Not an eligible study design
Just 2014 (S1)	No eligible interventions (within‐study comparisons)
Just 2014 (S2)	Not an eligible study design
Kallbekken 2013	Not an eligible study design
Kesman 2011	No eligible interventions (within‐study comparisons)
Kildegaard 2011	Not an eligible study design
Kozlowski 1989	Not an eligible study design
Kral 2004b	Not an empirical primary study
Lawless 2003	No measurement (assessment) of selection or consumption outcomes
Leidy 2010	Not an eligible study design
Levitsky 2011	No eligible interventions (within‐study comparisons)
Lewis 2013	No eligible interventions (within‐study comparisons)
Libotte 2014	No eligible interventions (within‐study comparisons)
Liem 2009	Not an eligible study design
Lieux 1992	No eligible interventions (within‐study comparisons)
Lin 2013	No eligible interventions (within‐study comparisons)
Meguid 1998	Animal study (non‐human participants)
Mendoza 2010	No eligible interventions (within‐study comparisons)
Olsen 2012	No measurement (assessment) of selection or consumption outcomes
Pornpitakpan 2010	No eligible interventions (within‐study comparisons)
Raghubir 1999	Not an eligible study design
Rolls 1982	No eligible interventions (within‐study comparisons)
Rolls 1985	Not an empirical primary study
Rolls 1990	No eligible interventions (within‐study comparisons)
Rolls 2012	Not an empirical primary study
Savage 2012	Not an eligible study design
Saylor 1987	Not an eligible study design
Scheibehenne 2010	Not an eligible study design
Scisco 2012 (S1)	No eligible interventions (within‐study comparisons)
Scisco 2012 (S2)	No eligible interventions (within‐study comparisons)
Sharafi 2010	Not an eligible study design
Spanos 2015	No eligible interventions (within‐study comparisons)
Spiegel 1993	Not an eligible study design
Spill 2011a	No eligible interventions (within‐study comparisons)
Stepney 1977	No eligible interventions (within‐study comparisons)
Tapsell 2014	No eligible interventions (within‐study comparisons)
Ueland 2009	No eligible interventions (within‐study comparisons)
Van Ittersum 2012	No eligible interventions (within‐study comparisons)
Vermeer 2011	No eligible interventions (within‐study comparisons)
Vermeer 2012a	No eligible interventions (within‐study comparisons)
Walker 2014	No eligible interventions (within‐study comparisons)
Wansink 2005a	No eligible interventions (within‐study comparisons)
Wansink 2005c	No eligible interventions (within‐study comparisons)
Wansink 2005e	Not an eligible study design. NOTE: On the 19^th^ September 2018, JAMA, JAMA Internal Medicine and JAMA Pediatrics retracted six articles, including this article, on which Brian Wansink (John Dyson Professor of Marketing at Cornell University), was an author (https://media.jamanetwork.com/news‐item/jama‐network‐retracts‐6‐articles‐that‐included‐dr‐brian‐wansink‐as‐author/).
Wansink 2007a	Not an empirical primary study
Weijzen 2008	No eligible interventions (within‐study comparisons)
Weijzen 2009	No eligible interventions (within‐study comparisons)
White 2003	No eligible interventions (within‐study comparisons)
Williams 2013	No eligible interventions (within‐study comparisons)
Wilson 2013	No eligible interventions (within‐study comparisons)
Woodson 1992	No eligible interventions (within‐study comparisons)
Yamauchi 2014	No eligible interventions (within‐study comparisons)
Yang 2005	No eligible interventions (within‐study comparisons)
Yee 1979	Not an eligible study design
Yeomans 2009	No eligible interventions (within‐study comparisons)
Yip 2013	No eligible interventions (within‐study comparisons)
Zijlstra 2009	No eligible interventions (within‐study comparisons)

### Characteristics of studies awaiting assessment [ordered by study ID]

**Table:** Bajaj 2014

Methods	Between‐subjects randomised controlled trial
Participants	313 undergraduate psychology students. Laboratory setting, Arizona State University, Arizona, USA
Interventions	Manipulated product type: food Target of manipulation: individual unit size (bagel) Duration of exposure to intervention: ≤ 1 day Concurrent intervention components: no Eligible comparison(s): Intervention 1: exposure to quartered (multiple‐piece) bagel smeared with cream cheese; *versus* Intervention 2: exposure to uncut (single‐piece) bagel smeared with cream cheese
Outcomes	Selection outcome selectable for analysis: not measured Consumption outcome selectable for analysis: energy intake from bagel (kcal) Measurement of consumption outcome: objective Timing of consumption outcome measurement: immediate (≤ 1 day) Consumption outcome – effective sample size for meta‐analysis: 301 Consumption outcome – study‐level effect size: 0.23 (0.01 to 0.45) Consumption outcome ‐ direction of effect: food: larger size increased consumption
Notes	Eligible study identified by updated search (30 January 2015). Accepted into the review and awaiting full integration. See also Results of the search and Appendix 2

**Table:** Haire 2014

Methods	Between‐subjects randomised controlled trial
Participants	67 adults. Laboratory setting, University of Tennessee campus area, TN, USA
Interventions	Manipulated product type: food Target of manipulation: package size Duration of exposure to intervention: > 1 day Concurrent intervention components: no Eligible comparison(s): Intervention 1: exposure to a box containing 22 x 0.9 oz packages of Snyder's of Hanover salted minipretzels; *versus* Intervention 2: exposure to a box containing 2 x 10.0 oz packages of Snyder's of Hanover salted minipretzels
Outcomes	Selection outcome selectable for analysis: not measured Consumption outcome selectable for analysis: total amount of pretzels consumed over 4 days (grams) Measurement of consumption outcome: objective Timing of consumption outcome measurement: longer‐term (> 1 day) Consumption outcome – effective sample size for meta‐analysis: 64 Consumption outcome – study‐level effect size: 0.23 (‐0.26 to 0.72) Consumption outcome ‐ direction of effect: food: no difference
Notes	Eligible study identified by updated search (30 January 2015). Accepted into the review and awaiting full integration. See also Results of the search and Appendix 2

**Table:** Kral 2014

Methods	Within‐subjects randomised controlled trial
Participants	63 children. Laboratory setting, University of Pennsylvania campus area, USA
Interventions	Manipulated product type: food Target of manipulation: portion size Duration of exposure to intervention: ≤ 1 day Concurrent intervention components: no Eligible comparison(s): [1] Intervention 1: exposure to 100% sized portions of chicken nuggets, hash browns, green beans (w/small amount of butter), brownie and fruit punch; *versus* Intervention 2: exposure to 150% sized portions of chicken nuggets, hash browns, green beans (w/small amount of butter), brownie and fruit punch [2] Intervention 1: exposure to 150% sized portions of chicken nuggets, hash browns, green beans (w/small amount of butter), brownie and fruit punch; *versus* Intervention 2: exposure to 200% sized portions of chicken nuggets, hash browns, green beans (w/small amount of butter), brownie and fruit punch
Outcomes	Selection outcome selectable for analysis: not measured Consumption outcome selectable for analysis: energy intake from total lunch meal (kcal) Measurement of consumption outcome: objective Timing of consumption outcome measurement: immediate (≤ 1 day) Consumption outcome – effective sample size for meta‐analysis: [1] 75 [2] 75 Consumption outcome – study‐level effect size: [1] 0.43 (‐0.05 to 0.91) [2] ‐0.02 (‐0.50 to 0.46) Consumption outcome ‐ direction of effect: [1] Food: no difference [2] Food: no difference
Notes	Eligible study identified by updated search (30 January 2015). Accepted into the review and awaiting full integration. See also Results of the search and Appendix 2

**Table:** Loney 2010

Methods	Between‐subjects trial with participants allocated equally to 2 intervention groups
Participants	30 obese adolescents (aged 14 to 19) recruited from a UAE weekday residential school
Interventions	Intervention 1: 4 portion‐controlled meals daily Intervention 2: 4 meals daily where portion size was not regulated
Outcomes	Weight loss
Notes	Unclear based on study report (conference abstract) whether study has an eligible design, eligible intervention or eligible outcome

**Table:** Marchiori 2014

Methods	Between‐subjects randomised controlled trial
Participants	110 university students. Laboratory setting, Tilburg University. Netherlands
Interventions	Manipulated product type: food Target of manipulation: portion size Duration of exposure to intervention: ≤ 1 day Concurrent intervention components: yes. Participants either listened to the introduction of the audio book "The Digital Fortress" by Dan Brown (i.e. the first 14 min) or received a body scan mindfulness exercise ‐ provided to both Intervention 1 and Intervention 2 groups (groups combined) Eligible comparison(s): Intervention 1: small portion; *versus* Intervention 2: large portion
Outcomes	Selection outcome selectable for analysis: not measured Consumption outcome selectable for analysis: energy intake from cookies and water (kcal) Measurement of consumption outcome: objective Timing of consumption outcome measurement: immediate (≤ 1 day) Consumption outcome – effective sample size for meta‐analysis: 110 Consumption outcome – study‐level effect size: 0.81 (0.42 to 1.20) Consumption outcome ‐ direction of effect: food: larger size increased consumption
Notes	Eligible study identified by updated search (30 January 2015). Accepted into the review and awaiting full integration. See also Results of the search and Appendix 2

**Table:** Martinez 2010

Methods	Within‐subjects trial with participants receiving both interventions
Participants	24 college students (12 female, 12 male)
Interventions	Intervention 1: receive 10 small pies (50 g each) equivalent in taste and texture to one large size portion Intervention 2: receive large size pie (500 g) equivalent in taste and texture to small size portion
Outcomes	Consumption of food; perceptions of consumption of food
Notes	Unclear based on study report (conference abstract) whether study has an eligible design

**Table:** Rolls 2014a

Methods	Within‐subjects randomised controlled trial
Participants	41 adults. Laboratory setting, University of Pennsylvania campus area, USA
Interventions	Manipulated product type: food Target of manipulation: individual unit size Duration of exposure to intervention: ≤ 1 day Concurrent intervention components: no Eligible comparison(s): [1] Intervention 1: exposure to 40% sized wheat flakes cereal; *versus* Intervention 2: exposure to 60% sized wheat flakes cereal [2] Intervention 1: exposure to 60% sized wheat flakes cereal; *versus* Intervention 2: exposure to 80% sized wheat flakes cereal [3] Intervention 1: exposure to 80% sized wheat flakes cereal; *versus* Intervention 2: exposure to standard (100%) sized wheat flakes cereal
Outcomes	Selection outcome selectable for analysis: amount of cereal selected (grams) Measurement of selection outcome: objective Timing of selection outcome measurement: immediate (≤ 1 day) Selection outcome ‐ effective sample size for meta‐analysis: [1] 61 [2] 61 [3] 61 Selection outcome ‐ study‐level effect size: [1] ‐0.32 (‐0.86 to 0.22) [2] ‐0.36 (‐0.98 to 0.26) [3] ‐0.35 (‐0.88 to 0.18) Selection outcome ‐ direction of effect: [1] Food: no difference [2] Food: no difference [3] Food: no difference Consumption outcome selectable for analysis: energy intake from breakfast cereal (kcal) Measurement of consumption outcome: objective Timing of consumption outcome measurement: immediate (≤ 1 day) Consumption outcome – effective sample size for meta‐analysis: [1] 61 [2] 61 [3] 61 Consumption outcome – study‐level effect size: [1] ‐0.15 (‐0.68 to 0.38) [2] ‐0.35 (‐0.97 to 0.27) [3] ‐0.32 (‐0.85 to 0.21) Consumption outcome ‐ direction of effect: [1] Food: no difference [2] Food: no difference [3] Food: no difference
Notes	Eligible study identified by updated search (30 January 2015). Accepted into the review and awaiting full integration. See also Results of the search and Appendix 2

**Table:** Schmidt 2013

Methods	Between‐subjects trial with participants allocated to one of 2 interventions
Participants	Danish business leaders that took part in a congress in Copenhagen, Denmark (n = 220)
Interventions	Participants allocated to one of 2 floors in a building, which determined which intervention was received: Intervention 1: allocated to buffet table that used smaller‐sized plates (24 cm) Intervention 2: allocated to buffet table that used normal‐sized (larger) plates (27 cm)
Outcomes	Food waste at a single serving in a self service eating setting. Collected in designated rubbish bags and weighed
Notes	Unclear based on study report (conference abstract) whether study has an eligible design

**Table:** Skov 2013

Methods	Between‐subjects trial with participants allocated to one of 2 interventions
Participants	People attending a congress in Copenhagen, Denmark (n = 391)
Interventions	Participants allocated to one of 2 groups for snacking during breaks, which determined which intervention was received: Intervention 1: allocated to table for snacking with halved pieces of cake as well as apples served in quarter pieces Intervention 2: allocated to table for snacking with normal (full) sized pieces of cake as well as whole apples
Outcomes	Quantity of cake and apples consumed, measured by observation using electronic counting system
Notes	Unclear based on study report (conference abstract) whether study has an eligible design

**Table:** Smith 2013a

Methods	Within‐subjects, cluster‐randomised controlled trial
Participants	250 children aged 3 to 6 years. Field setting. DaGuan Kindergarten, Kunming, Yunnan Province, China
Interventions	Manipulated product type: food Target of manipulation: portion size Duration of exposure to intervention: ≤ 1 day Concurrent intervention components: no Eligible comparison(s): [1] Intervention 1: exposure to 105 g portion of rice/vegetable/protein mix and soup; *versus* Intervention 2: exposure to 150g portion of rice/vegetable/protein mix and soup [2] Intervention 1: exposure to 150 g portion of rice/vegetable/protein mix and soup; *versus* Intervention 2: exposure to 195g portion of rice/vegetable/protein mix and soup [3] Intervention 1: exposure to 182 g portion of rice/vegetable/protein mix and soup; *versus* Intervention 2: exposure to 261g portion of rice/vegetable/protein mix and soup. [4] Intervention 1: exposure to 261 g portion of rice/vegetable/protein mix and soup; *versus* Intervention 2: exposure to 389g portion of rice/vegetable/protein mix and soup
Outcomes	Selection outcome selectable for analysis: not measured Consumption outcome selectable for analysis: amount consumed from portion of rice/vegetable/protein mix and soup (grams) Measurement of consumption outcome: objective Timing of consumption outcome measurement: immediate (≤ 1 day) Consumption outcome – effective sample size for meta‐analysis: [1] 141 [2] 141 [3] 115 [4] 115 Consumption outcome – study‐level effect size: [1] 1.04 (0.67 to 1.41) [2] ‐0.96 (‐1.33 to ‐0.59) [3] 0.61 (0.22 to 1.00) [4] 0.67 (0.27 to 1.07) Consumption outcome ‐ direction of effect: [1] Food: larger size reduced consumption [2] Food: larger size increased consumption [3] Food: larger size increased consumption [4] Food: larger size increased consumption
Notes	Eligible study identified by updated search (30 January 2015). Accepted into the review and awaiting full integration. See also Results of the search and Appendix 2.

**Table:** van Ittersum 2013

Methods	Within‐subjects randomised controlled trial
Participants	18 elementary school children. Field setting, school cafeteria during 4‐week summer camp, USA
Interventions	Manipulated product type: food Target of manipulation: tableware size (cereal bowl) Duration of exposure to intervention: ≤ 1 day Concurrent intervention components: no Eligible comparison(s): Intervention 1: exposure to a 12 oz cereal bowl; *versus* Intervention 2: exposure to a 16 oz cereal bowl
Outcomes	Selection outcome selectable for analysis: amount of cereal and milk self served or served (grams) Measurement of selection outcome: objective. Timing of selection outcome measurement: immediate (≤ 1 day) Selection outcome ‐ effective sample size for meta‐analysis: 36 Selection outcome ‐ study‐level effect size: no useable data Selection outcome ‐ direction of effect: food: larger size increased selection (based on study authors' conclusion ‐ to be confirmed) Consumption outcome selectable for analysis: amount of cereal and milk consumed (grams) Measurement of consumption outcome: objective Timing of consumption outcome measurement: immediate (≤ 1 day) Consumption outcome – effective sample size for meta‐analysis: 36 Consumption outcome – study‐level effect size: no useable data Consumption outcome ‐ direction of effect: Food: larger size increased consumption (based on study authors' conclusion)
Notes	Eligible study identified by updated search (30 January 2015). Accepted into the review and awaiting full integration. See also Results of the search and Appendix 2

**Table:** van Kleef 2014

Methods	Between‐subjects randomised controlled trial
Participants	165 university students. Laboratory setting, Dutch university (unspecified). Netherlands
Interventions	Manipulated product type: food Target of manipulation: individual unit size Duration of exposure to intervention: ≤ 1 day Concurrent intervention components: yes. Computer‐based task that involved viewing and rating non‐food commercials on several aspects ("humoristic nature, attractiveness etc.") ‐ provided to both Intervention 1 and Intervention 2 groups. Eligible comparison(s): Intervention 1: exposure to 15 small Mars chocolate bars with a total weight of 150 g (45 calories each, resulting in 675 calories in total); *versus* Intervention 2: exposure to 3 Mars chocolate bars of 51 g (228 calories per bar, resulting in 684 calories in total)
Outcomes	Selection outcome selectable for analysis: not measured Consumption outcome selectable for analysis: energy intake from chocolate bars (kcal) Measurement of consumption outcome: objective Timing of consumption outcome measurement: immediate (≤ 1 day) Consumption outcome – effective sample size for meta‐analysis: 162 Consumption outcome – study‐level effect size: 0.48 (0.17 to 0.79) Consumption outcome ‐ direction of effect: food: larger size increased consumption
Notes	Eligible study identified by updated search (30 January 2015). Accepted into the review and awaiting full integration. See also Results of the search and Appendix 2

**Table:** Wansink 2013

Methods	Between‐subjects cluster‐randomised controlled trial
Participants	2150 middle school students. Field study, school lunchrooms, Wayne County, NY, USA
Interventions	Manipulated product type: food Target of manipulation: individual unit size Duration of exposure to intervention: > 1 day Concurrent intervention components: no Eligible comparison(s): Intervention 1: exposure to apples sliced into 6 symmetric pieces available for purchase in the school lunchroom; *versus* Intervention 2: exposure to whole apples available for purchase in the school lunchroom
Outcomes	Selection outcome selectable for analysis: purchased an apple/did not purchase an apple on study days (unclear ‐ subject to author confirmation) Measurement of selection outcome: objective Timing of selection outcome measurement: longer term (> 1 day) (unclear ‐ subject to author confirmation) Selection outcome ‐ effective sample size for meta‐analysis: 4300 Selection outcome ‐ study‐level effect size: No useable data Selection outcome ‐ direction of effect: food: larger size reduced selection (based on study authors' conclusion ‐ to be confirmed) Consumption outcome selectable for analysis: amount of apple consumed per student (grams) (unclear ‐ subject to author confirmation) Measurement of consumption outcome: objective Timing of consumption outcome measurement: longer‐term (> 1 day) (unclear ‐ subject to author confirmation) Consumption outcome – effective sample size for meta‐analysis: 4300 Consumption outcome – study‐level effect size: no useable data Consumption outcome ‐ direction of effect: food: larger size reduced consumption (based on study authors' conclusion ‐ to be confirmed)
Notes	Eligible study identified by updated search (30 January 2015). Accepted into the review and awaiting full integration. See also Results of the search and Appendix 2

**Table:** Wansink 2014

Methods	Between‐subjects randomised controlled trial
Participants	69 preschool aged children. Field setting, school lunchrooms, unspecified, USA
Interventions	Manipulated product type: food Target of manipulation: tableware size (cereal bowl) Duration of exposure to intervention: ≤ 1 day Concurrent intervention components: no Eligible comparison(s): Intervention 1: exposure to an 8 oz cereal bowl at breakfast; *versus* Intervention 2: exposure to a 16 oz cereal bowl at breakfast
Outcomes	Selection outcome selectable for analysis: amount of cereal and milk served for breakfast (grams) Measurement of selection outcome: objective Timing of selection outcome measurement: immediate (≤ 1 day) Selection outcome ‐ effective sample size for meta‐analysis: 69 Selection outcome ‐ study‐level effect size: 1.41 (0.88 to 1.94) Selection outcome ‐ direction of effect: food: larger size increased selection Consumption outcome selectable for analysis: not measured
Notes	Eligible study identified by updated search (30 January 2015). Accepted into the review and awaiting full integration. See also Results of the search and Appendix 2

**Table:** Williams 2014

Methods	Within‐subjects randomised controlled trial
Participants	54 adult women. Laboratory setting, Pennsylvania State University campus, USA
Interventions	Manipulated product type: food Target of manipulation: portion size Duration of exposure to intervention: ≤ 1 day Concurrent intervention components: no Eligible comparison(s): Intervention 1: exposure to salad preload followed by 450 g portion pasta entrée; *versus* Intervention 2: exposure to salad preload followed by 600 g portion pasta entrée
Outcomes	Selection outcome selectable for analysis: not measured Consumption outcome selectable for analysis: energy intake from entire lunch meal (kcal) Measurement of consumption outcome: objective Timing of consumption outcome measurement: immediate (≤ 1 day) Consumption outcome – effective sample size for meta‐analysis: 92 Consumption outcome – study‐level effect size: 0.46 (0.05 to 0.87) Consumption outcome ‐ direction of effect: Food: larger size increased consumption
Notes	Eligible study identified by updated search (30 January 2015). Accepted into the review and awaiting full integration. See also Results of the search and Appendix 2

## Differences between protocol and review

A difference between the protocol (Hollands 2014) and review is that the proposed search of the Cochrane Public Health Group Specialised Register was not, in practice, conducted. This omission is unlikely to have had any impact on the review. Study records on the Cochrane Public Health Group Specialised Register are submitted for inclusion in the Cochrane Central Register of Controlled Trials (CENTRAL) on a quarterly basis and we conducted searches of CENTRAL for this review up to 30 January 2015. Also, at the protocol stage, we intended to use the most commonly available measure of participants' socioeconomic status to construct the socioeconomic status context variable (see Data extraction and management). We were unable to do this in practice because no single proxy measure of participants' socioeconomic status, such as education or income, was commonly measured in and reported by included studies. Therefore we instead coded a binary study‐level covariate based on authors' explicit descriptors of the study sample and/or setting (e.g. "Low income Hispanic or non‐Hispanic African American children and their mothers", or "Faculty, graduate students, and staff members of the Department of Food Science and Nutritional Science of a large Midwestern university". Unless explicitly described as being of low socioeconomic status, we coded the context of included studies as high socioeconomic status.
